# Supplementary material for: Contribution to the Synthesis, Characterization, Separation and Quantification of New N-Acyl Thiourea Derivatives with Antimicrobial and Antioxidant Potential
Source: Pharmaceutics. 2023 Oct 20;15(10):2501. doi: 10.3390/pharmaceutics15102501 (PMC10609700; doi:10.3390/pharmaceutics15102501)
Supplement: Supplementary file 1 [file pharmaceutics-15-02501-s001.zip › pharmaceutics-2593306-supplementary.pdf]

pharmaceutics

MDPI

RP-HPLC Method Validation for Separation and Quantitative Determination of compounds **1h**, **1j**, **1i**, **1m**, **1g**, and **1n**

Analytical Report

1. SCOPE

The aim of the validation of an analytical procedure is to demonstrate that it is suitable for its intended purpose. A reversed-phase high-performance liquid chromatography (RP-HPLC) method used for separation and quantitative determination of compounds **1h, 1j, 1i, 1m, 1g, 1n** was developed and validated.

2. DESCRIPTION OF THE METHOD

Principle of the method: A reversed-phase high-performance liquid chromatography (RP-HPLC) method for separation and quantitative determination of **compounds 1h**, **1j**, **1i**, **1m**, **1g**, and **1n** has been developed and validated.

Reagents:

- Water for chromatographic use;
- Acetonitrile;
- Methanol;
- Triethylamine;
- Ortho-phosphoric acid;

Equipment:

- Waters Alliance HPLC system, or equivalent, comprised of the following modules:
  - 2695 + 2998 separation module;
  - 998 PDA detector;
  - PC equipped with “Empower 3, PDA Software”;
- Mettler Toledo analytical balance;
- pH meter inoLab pH7310P.

Chromatographic conditions:

- Column: stationary phase: octadecylsilica gel C18; length: 250 mm; internal diameter: 4.6 mm; particle size: 5µm (Inertsil ODS-3, 5 µm, 250 x 4.6 mm);
- Flow rate: 1.0 mL/ min;
- Detection: λ=275 nm;
- Injection volume: 10 µL;
- Run time: 35 minutes;
- Column temperature: room temperature;
- Sample temperature: room temperature;
- Elution: isocratic.
- *Mobile phase:* Solvent A: Solvent B (93: 7, V/V), where,
    - *Solvent A*: 200 mL mixture of water R and 800 mL acetonitrile R (20: 80, V/ V) are mixed together, 0.7 mL triethylamine R is added, and the pH is adjusted to 3.3 with ortho-phosphoric acid R. The obtained solution is filtered and sonicated for around one hour before use.
    - *Solvent B*: Water R (of chromatographic use).

Preparation of solutions:

Stock solutions of 500 µg/ mL are prepared from each of the tested compounds:

*Stock solution **1h** (500 µg/ mL):* 5 mg compound **1h** were transferred into a 10 mL volumetric flask, 5 mL acetonitrile R were added, the flask was sonicated for 5 minutes, cooled to room temperature and diluted to volume with acetonitrile R.

*Stock solution **1j** (500 µg/ mL):* 5 mg compound **1j** were transferred into a 10 mL volumetric flask, 5 mL acetonitrile R were added, the flask was sonicated for 5 minutes, cooled to room temperature and diluted to volume with acetonitrile R.

*Stock solution **1i** (500 µg/ mL):* 5 mg compound **1i** were transferred into a 10 mL volumetric flask, 5 mL acetonitrile R were added, the flask was sonicated for 5 minutes, cooled to room temperature and diluted to volume with acetonitrile R.

*Stock solution **1m** (500 µg/ mL):* 5 mg compound **1m** were transferred into a 10 mL volumetric flask, 5 mL acetonitrile R were added, the flask was sonicated for 5 minutes, cooled to room temperature and

diluted to volume with acetonitrile R.

Stock solution ***1g*** *(500 µg/ mL):* 5 mg compound **1g** were transferred into a 10 mL volumetric flask, 5 mL acetonitrile R were added, the flask was sonicated for 5 minutes, cooled to room temperature and diluted to volume with acetonitrile R.

Stock solution ***1n*** *(500 µg/ mL):* 5 mg compound **1n** were transferred into a 10 mL volumetric flask, 5 mL acetonitrile R were added, the flask was sonicated for 5 minutes, cooled to room temperature and diluted to volume with acetonitrile R.

From the stock solutions, suitable dilutions were made in order the perform the validation of the analytical procedure.

System suitability:

The system suitability is checked by investigating the retention time of the main peaks due to tested compounds, resolution, theoretical plates, and symmetry factors.

6. ACCEPTANCE CRITERIA AND VALIDATION STAGES DESCRIPTION

For the validation of the afore described analytical method, the following parameters were evaluated:

- Specificity;
- Precision;
- LOD – LOQ;
- Linearity and range;
- Accuracy;
- Robustness.

Table S1. Acceptance criteria for proposed analytical method

Validation characteristic	Experimental details	Acceptance criteria
**Specificity**	No interference between the analytic signal of tested compounds and the analytic signal of solvent	-
**Precision**	A minimum of 6 samples	RSD ≤ 2%
**LOD – LOQ**	Correlation coefficient	$R^2 \geq 0.99$
**Linearity**	Correlation coefficient	$R^2 \geq 0.99$
**Range**	LOQ – 6.0 µg/ mL of each validated compound	$R^2 \geq 0.99$
**Accuracy**	At least 9 determinations over 3 concentration levels	Mean recovery within 98 – 102%
**Robustness**	The influence of small variations in chromatographic conditions on compounds 1h, 1j, 1i, 1m, 1g, 1n for analytical method efficiency (retention time, resolution)	Resolution ≥ 2

Acceptance criteria

SPECIFICITY

Specificity is the ability to assess unequivocally the analyte in the presence of components which may be expected to be present.

In order to demonstrate the specificity of the method, the following solutions were prepared and analyzed:

- Solvent;
- Reference Solution;
- Identification solutions;

Preparation of solutions:

Stock solutions of 500 µg/ mL are prepared from each of the tested compounds:

*Stock solution **1h** (500 µg/ mL):* 5 mg compound **1h** were transferred into a 10 mL volumetric flask, 5 mL acetonitrile R were added, the flask was sonicated for 5 minutes, cooled to room temperature and diluted to volume with acetonitrile R.

*Stock solution **1j** (500 µg/ mL):* 5 mg compound **1j** were transferred into a 10 mL volumetric flask, 5 mL acetonitrile R were added, the flask was sonicated for 5 minutes, cooled to room temperature and diluted to volume with acetonitrile R.

*Stock solution **1i** (500 µg/ mL):* 5 mg compound **1i** were transferred into a 10 mL volumetric flask, 5 mL acetonitrile R were added, the flask was sonicated for 5 minutes, cooled to room temperature and diluted to volume with acetonitrile R.

*Stock solution **1m** (500 µg/ mL):* 5 mg compound **1m** were transferred into a 10 mL volumetric flask, 5 mL acetonitrile R were added, the flask was sonicated for 5 minutes, cooled to room temperature and diluted to volume with acetonitrile R.

*Stock solution **1g** (500 µg/ mL):* 5 mg compound **1g** were transferred into a 10 mL volumetric flask, 5 mL acetonitrile R were added, the flask was sonicated for 5 minutes, cooled to room temperature and diluted to volume with acetonitrile R.

*Stock solution **1n** (500 µg/ mL):* 5 mg compound **1n** were transferred into a 10 mL volumetric flask, 5 mL acetonitrile R were added, the flask was sonicated for 5 minutes, cooled to room temperature and diluted to volume with acetonitrile R.

From the stock solutions, 0.25 mL were diluted to 25 mL with methanol R in order the obtain identification solutions of 5 µg/ mL.

pharmaceutics

Procedure:

The following sequence is used: *MeOH*, S_{1h}, S_{1j}, S_{1i}, S_{1m}, S_{1g}, S_{1n}, where:

MeOH = Methanol – the solvent used for samples preparation;

S_{1h} = 5 µg/ mL solution **1h**;

S_{1j} = 5 µg/ mL solution **1j**;

S_{1i} = 5 µg/ mL solution **1i**;

S_{1m} = 5 µg/ mL solution **1m**;

S_{1g} = 5 µg/ mL solution **1g**;

S_{1n} = 5 µg/ mL solution **1n**.

Results:

There was no detectable influence of solvent – methanol (**Figure S1**) on the identification of compounds **1h**, **1j**, **1i**, **1m**, **1g**, and **1n**.

Purity angles of all peaks recorded in the solutions chromatograms are below their corresponding thresholds, indicating proper separation of the compounds (**Figures S2 – S9**).

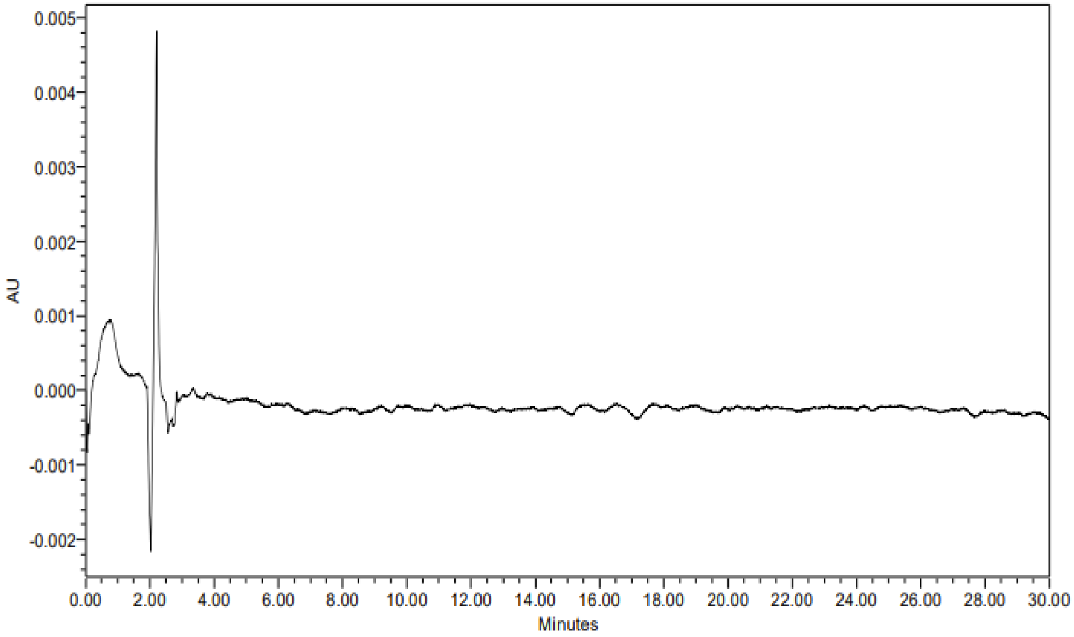

Figure S1. Chromatogram registered with the solvent used for samples preparation (methanol)

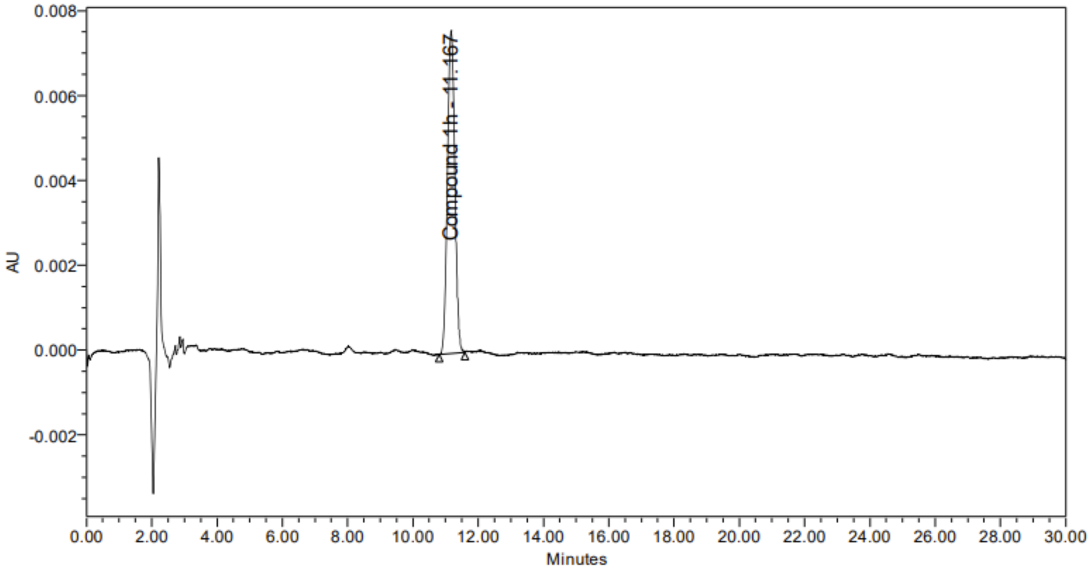

	Peak Name	RT	Area	Height (µV)	Purity1 Angle	Purity1 Threshold	Symmetry Factor	EP Plate Count
1	Compound 1h	11.167	119025	7615	0.299	0.650	1.06	1.135571e+004

(a)

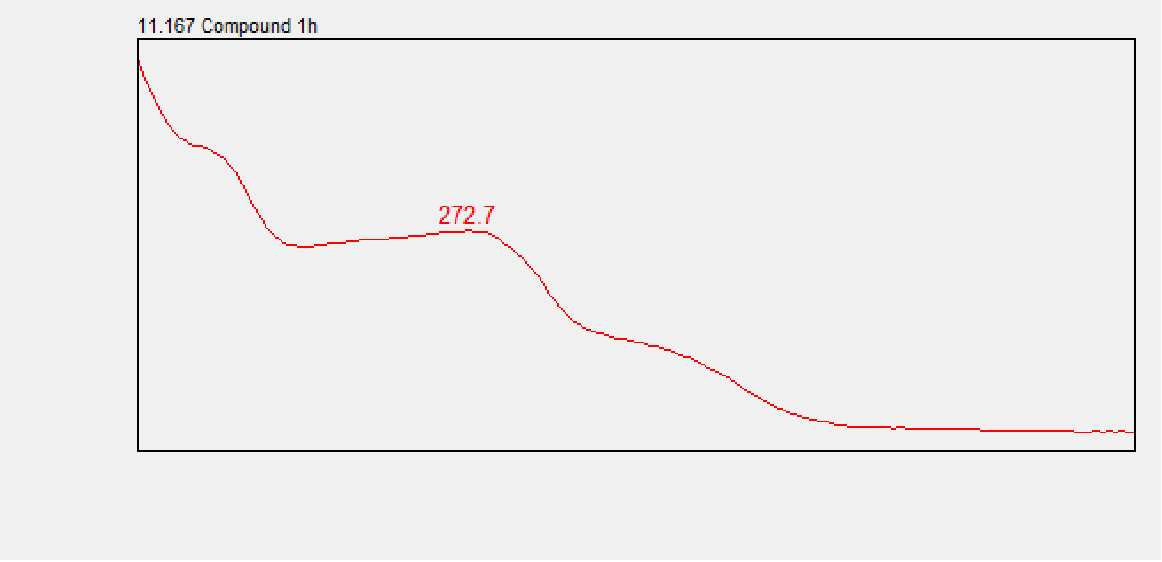

(b)

Figure S2. **(a)** Chromatogram registered with 5 µg/ mL **1h** solution;
(b) UV spectrum of compound **1h**

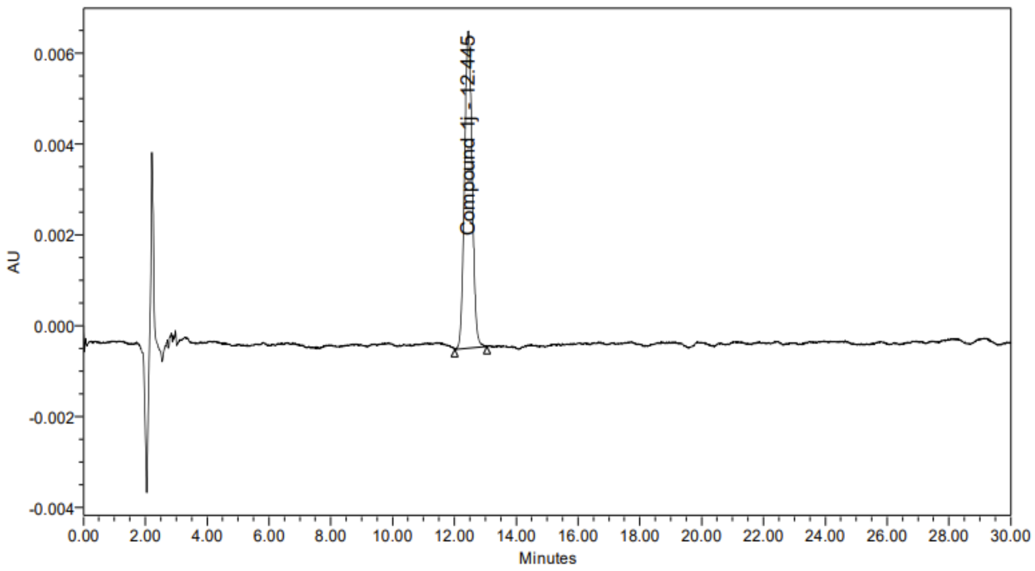

	Peak Name	RT	Area	Height (μV)	Purity1 Angle	Purity1 Threshold	Symmetry Factor	EP Plate Count
1	Compound 1j	12.445	122578	6978	0.387	0.642	1.07	1.134319e+004

(a)

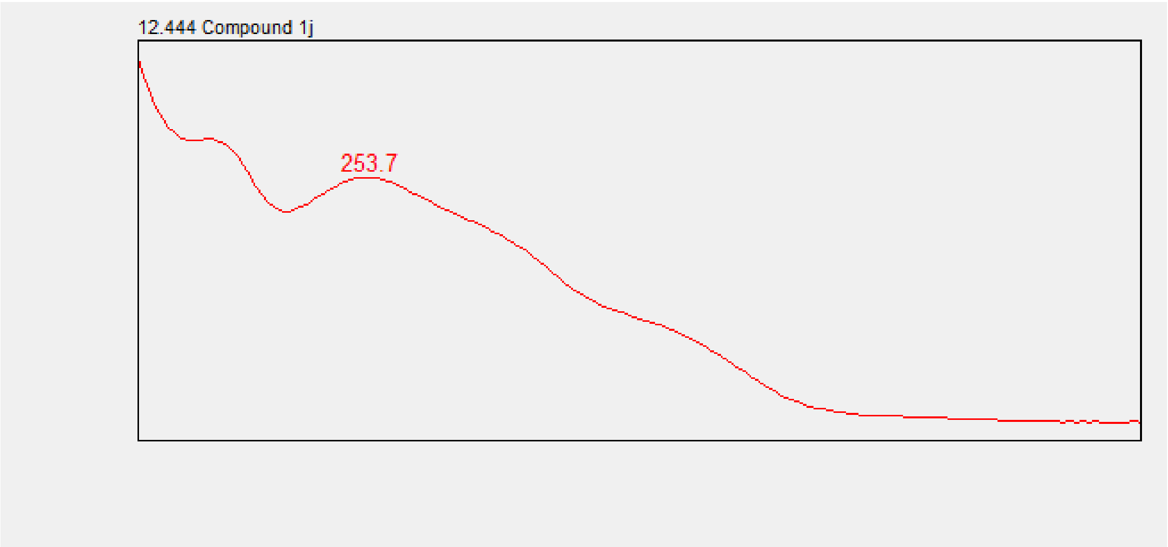

(b)

Figure S3. **(a)** Chromatogram registered with 5 μg/ mL **1j** solution; **(b)** UV spectrum of compound **1j**

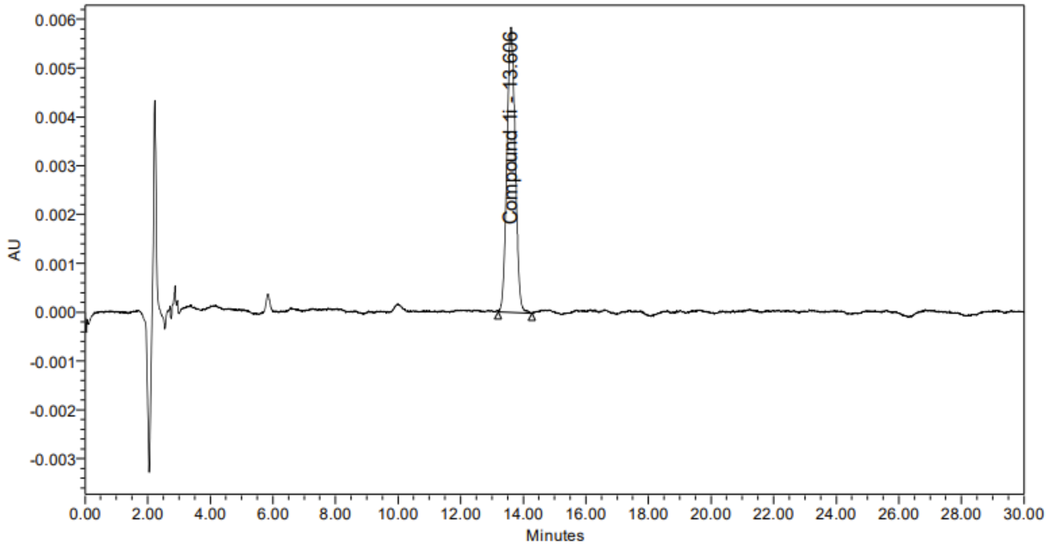

	Peak Name	RT	Area	Height (μV)	Purity1 Angle	Purity1 Threshold	Symmetry Factor	EP Plate Count
1	Compound 1i	13.606	110199	5835	0.337	0.636	1.07	1.165344e+004

(a)

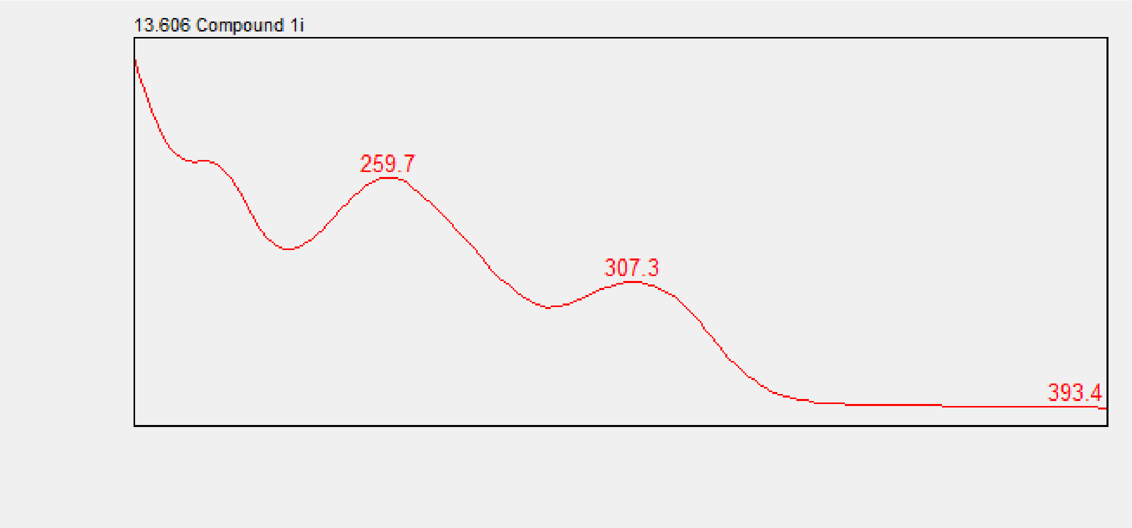

(b)

Figure S4. **(a)** Chromatogram registered with 5 μg/ mL **1i** solution; **(b)** UV spectrum of compound **1i**

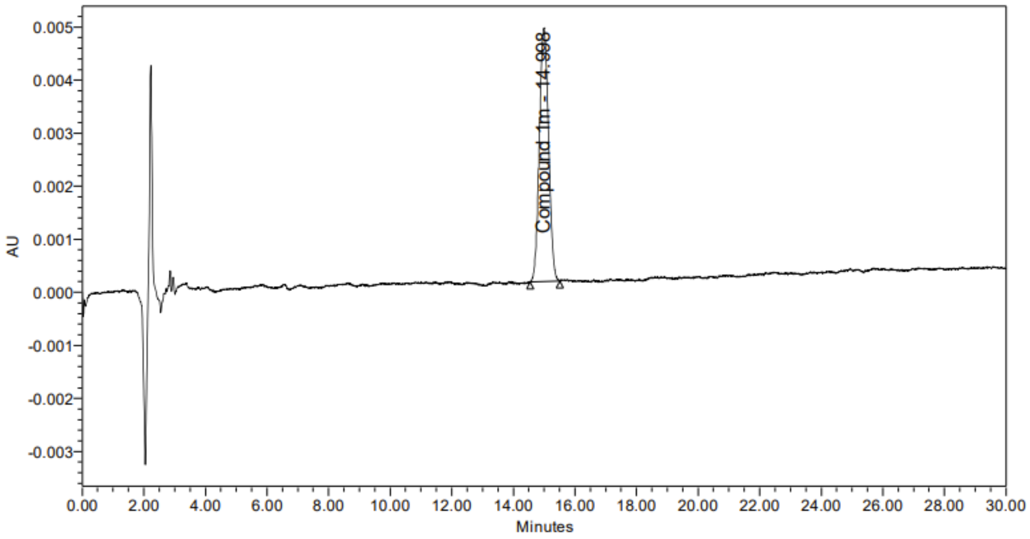

	Peak Name	RT	Area	Height (μV)	Purity1 Angle	Purity1 Threshold	Symmetry Factor	EP Plate Count
1	Compound 1m	14.998	99488	4779	0.380	0.604	1.02	1.145172e+004

(a)

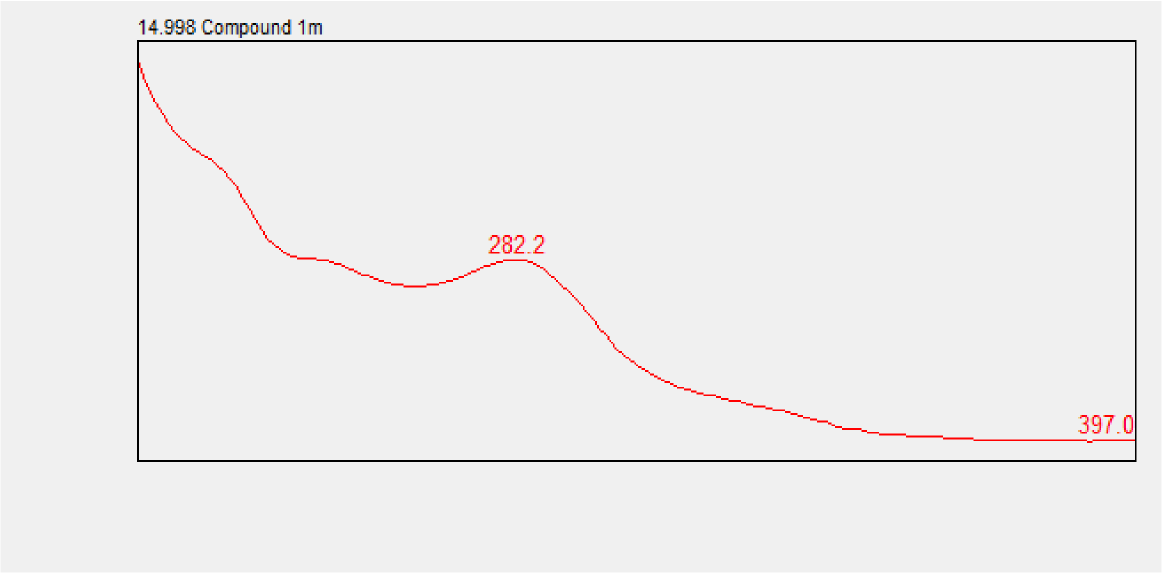

(b)

Figure S5. **(a)** Chromatogram registered with 5 μg/ mL **1m** solution;
(b) UV spectrum of compound **1m**

pharmaceutics

MDPI

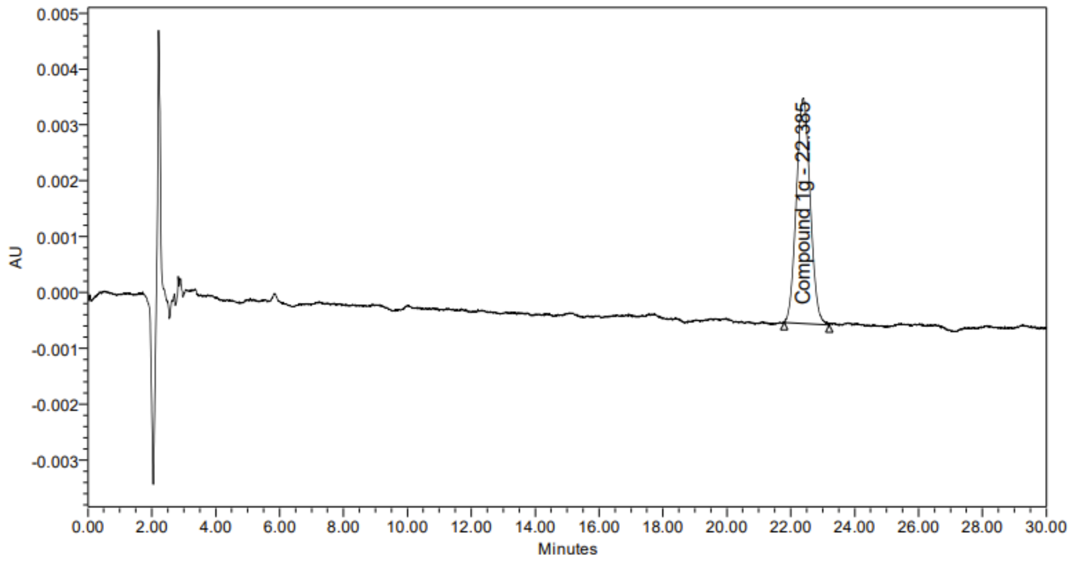

	Peak Name	RT	Area	Height (μV)	Purity1 Angle	Purity1 Threshold	Symmetry Factor	EP Plate Count
1	Compound 1g	22.385	122960	4032	0.785	1.369	1.07	1.209107e+004

(a)

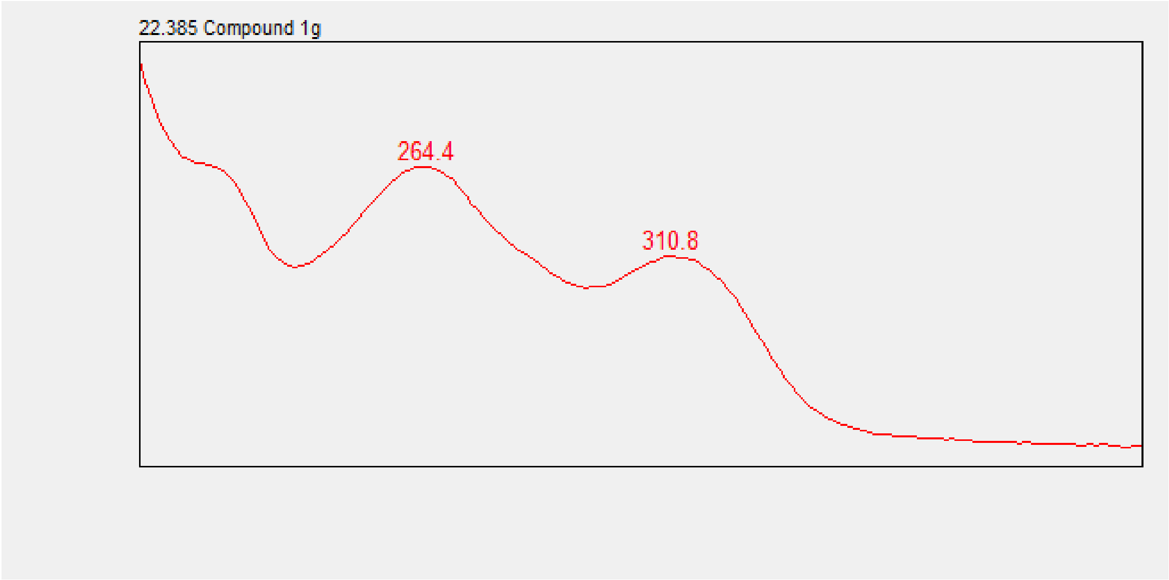

(b)

Figure S6. **(a)** Chromatogram registered with 5 μg/ mL **1g** solution;
(b) UV spectrum of compound **1g**

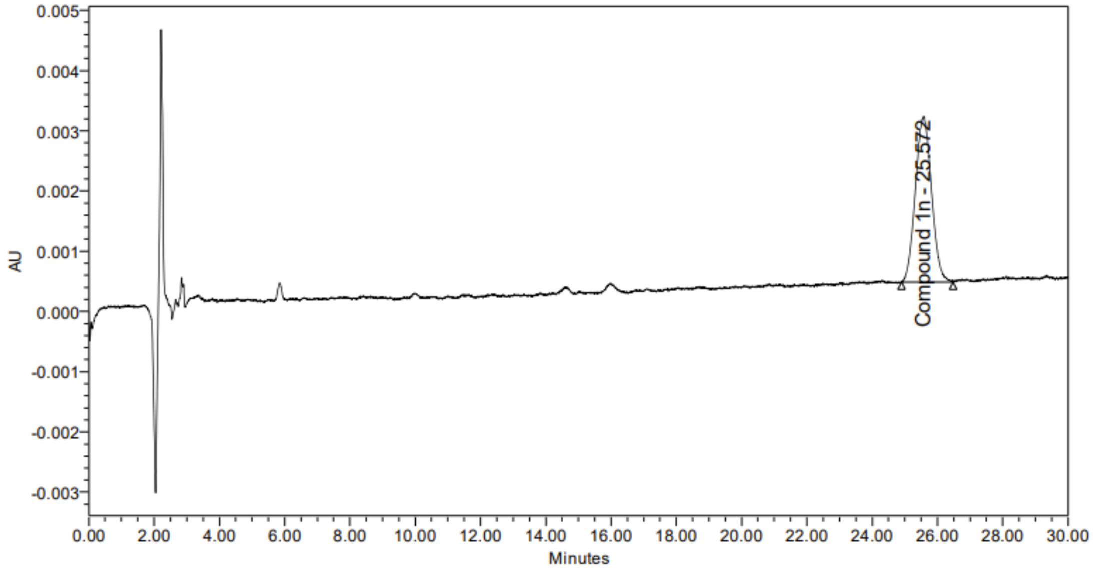

	Peak Name	RT	Area	Height (µV)	Purity1 Angle	Purity1 Threshold	Symmetry Factor	EP Plate Count
1	Compound 1n	25.572	95177	2744	0.693	1.463	1.05	1.208410e+004

(a)

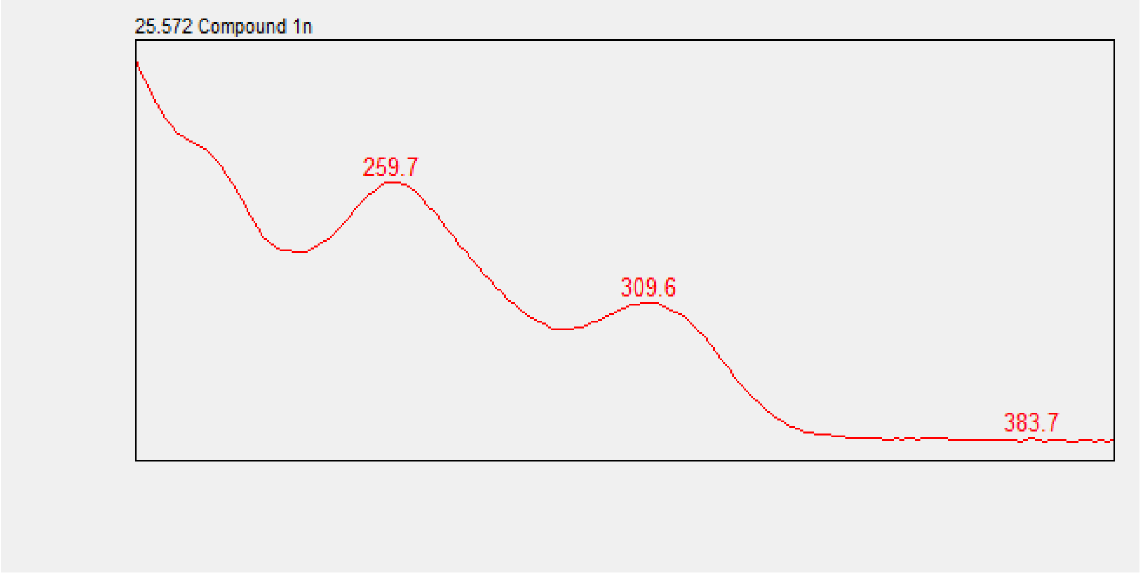

(b)

Figure S7. **(a)** Chromatogram registered with 5 µg/ mL **1n** solution;
(b) UV spectrum of compound **1n**

pharmaceutics

MDPI

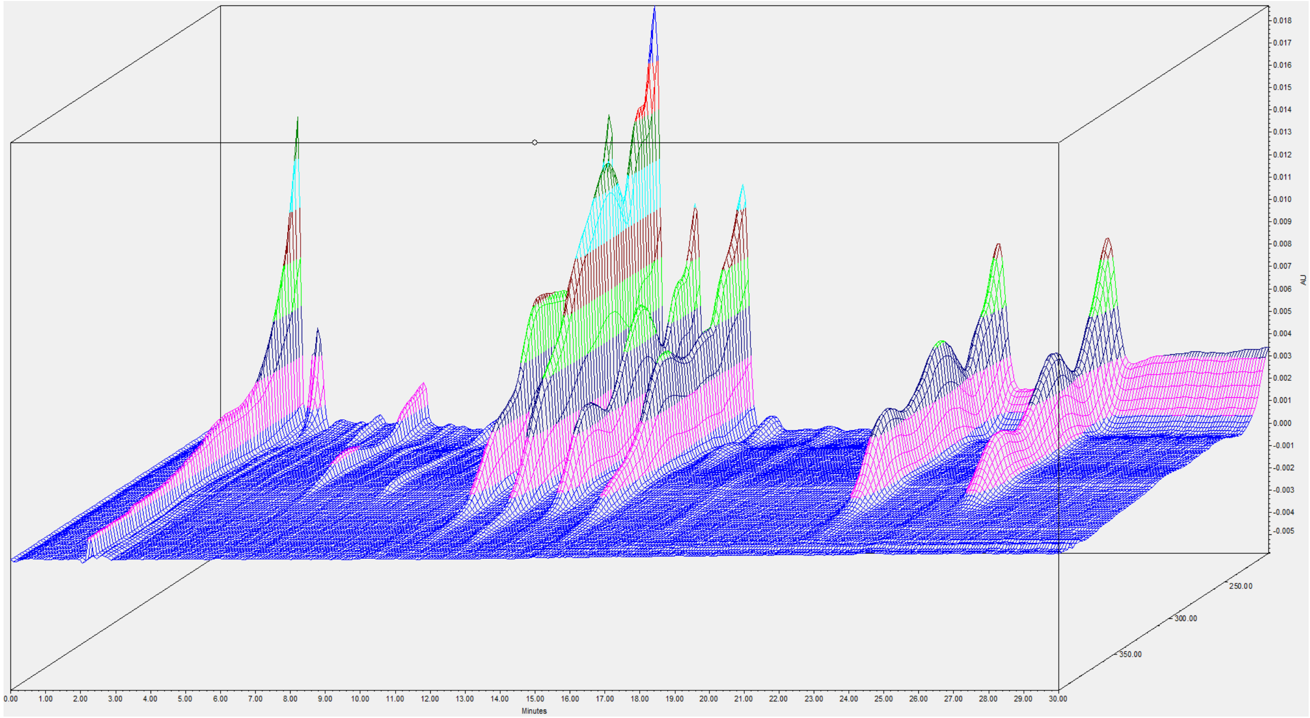

Figure S8. 3D Plot of compounds **1h**, **1j**, **1i**, **1m**, **1g**, and **1n**

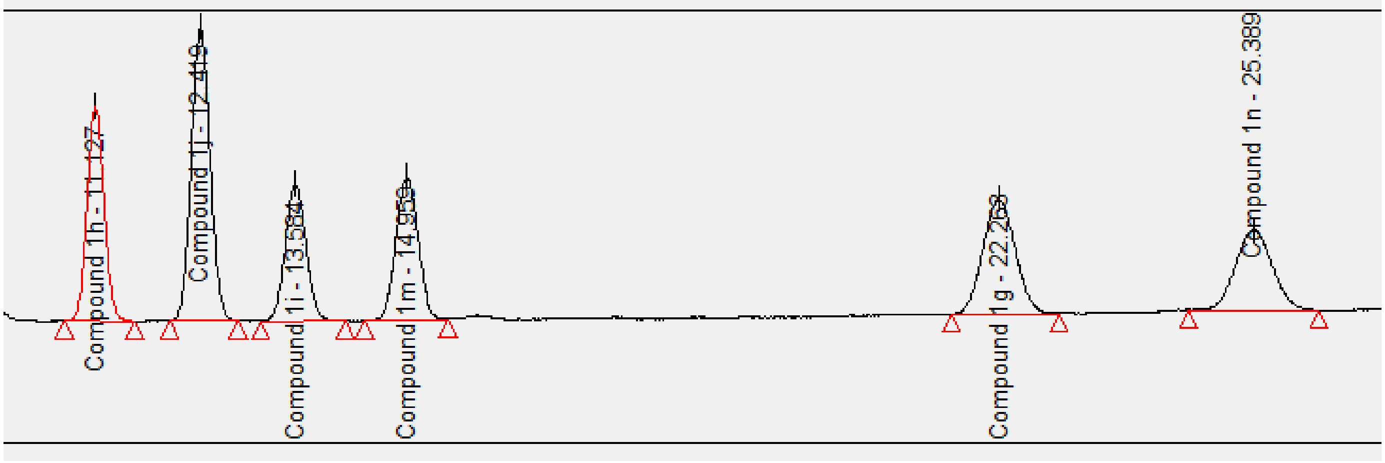

Figure S9. Typical chromatogram depicting **1h**, **1j**, **1i**, **1m**, **1g**, and **1n** peaks, for the proposed RP-HPLC separation method

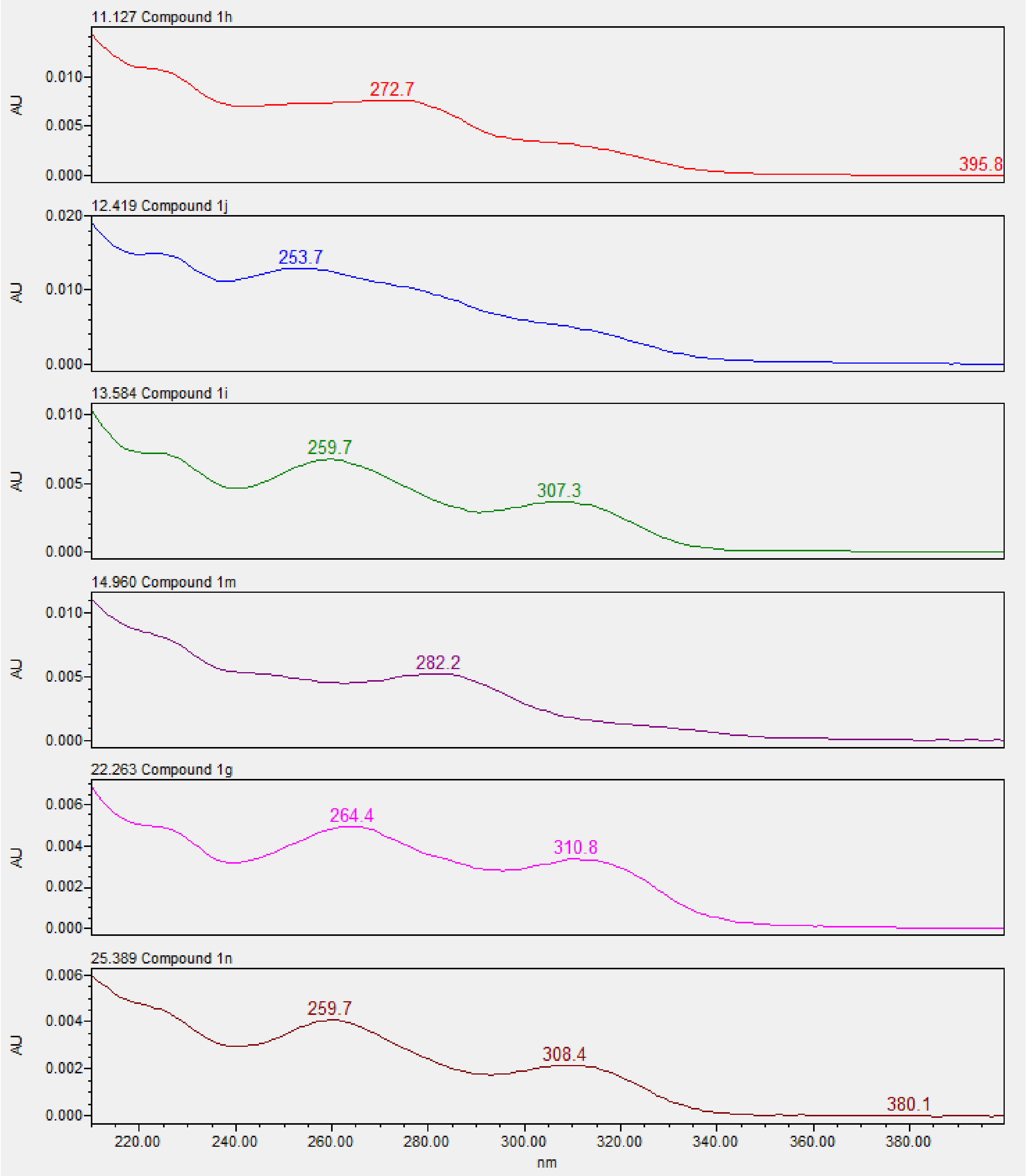

Figure S10. UV Spectra of the chemical compounds **1h**, **1j**, **1i**, **1m**, **1g**, and **1n**

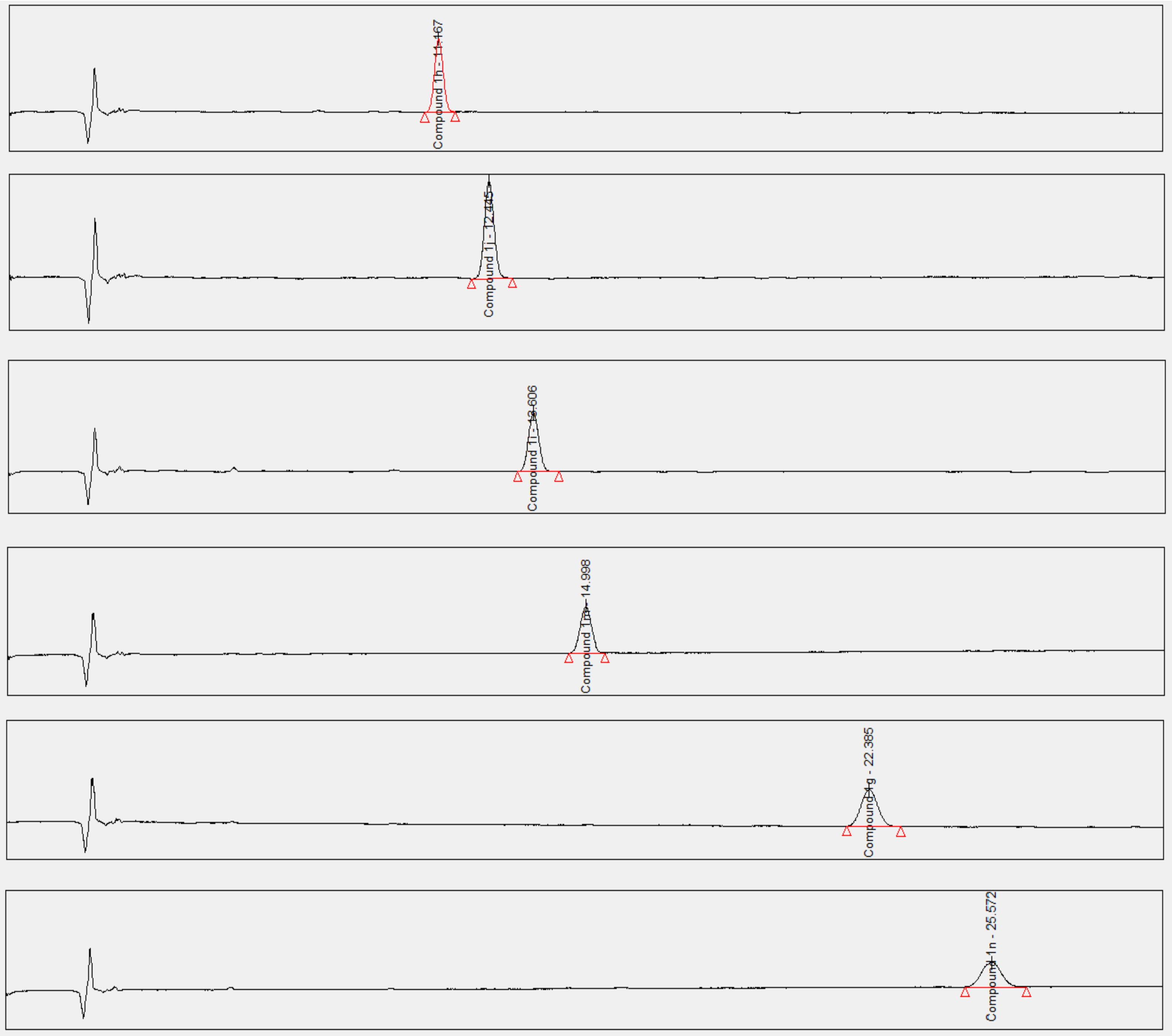

Figure S11. Identification chromatograms depicting **1h**, **1j**, **1i**, **1m**, **1g**, and **1n** peaks, registered following the proposed RP-HPLC separation method conditions

PRECISION

The precision of an analytical procedure expresses the closeness of agreement (degree of scatter) between a series of measurements obtained from multiple sampling of the same homogeneous sample under the prescribed conditions.

Precision may be considered at three levels: repeatability, intermediate precision and reproducibility. Precision should be investigated using homogeneous, authentic samples. However, if it is not possible to obtain a homogeneous sample, it may be investigated using artificially prepared samples or a sample solution.

The precision of an analytical procedure is usually expressed as the variance, standard deviation or coefficient of variation of a series of measurements.

Repeatability

Reapetability expresses the precision under the same operating conditions over a short interval of time. Repeatability is also termed intra-assay precision.

Intermediate precision

Intermediate precision expresses within-laboratories variations: different days, different analysts, different equipment etc.

Reproducibility

Reproducibility expresses the precision between laboratories (collaborative studies, usually applied to standardization of methodology).

Precision (Repeatability):

In order to demonstrate the precision of the method for ***the validation of separation and quantitative determination of compounds 1h***, ***1j***, ***1i***, ***1m***, ***1g***, *and* ***1n***, the following solution was prepared and analyzed in 2 different days:

- *Test solution* (5 µg/ mL compound **1h**, 5 µg/ mL compound **1j**, 5 µg/ mL compound **1i**, 5 µg/ mL compound **1m**, 5 µg/ mL compound **1g**, 5 µg/ mL compound **1n**).

<u>Preparation of solutions</u>:

Stock solutions of 500 µg/ mL are prepared from each of the tested compounds:

*Stock solution **1h** (500 µg/ mL):* 5 mg compound **1h** were transferred into a 10 mL volumetric flask, 5 mL acetonitrile R were added, the flask was sonicated for 5 minutes, cooled to room temperature and diluted to volume with acetonitrile R.

Stock solution ***1j*** *(500 µg/ mL):* 5 mg compound **1j** were transferred into a 10 mL volumetric flask, 5 mL acetonitrile R were added, the flask was sonicated for 5 minutes, cooled to room temperature and diluted to volume with acetonitrile R.

Stock solution ***1i*** *(500 µg/ mL):* 5 mg compound **1i** were transferred into a 10 mL volumetric flask, 5 mL acetonitrile R were added, the flask was sonicated for 5 minutes, cooled to room temperature and diluted to volume with acetonitrile R.

Stock solution ***1m*** *(500 µg/ mL):* 5 mg compound **1m** were transferred into a 10 mL volumetric flask, 5 mL acetonitrile R were added, the flask was sonicated for 5 minutes, cooled to room temperature and diluted to volume with acetonitrile R.

Stock solution ***1g*** *(500 µg/ mL):* 5 mg compound **1g** were transferred into a 10 mL volumetric flask, 5 mL acetonitrile R were added, the flask was sonicated for 5 minutes, cooled to room temperature and diluted to volume with acetonitrile R.

Stock solution ***1n*** *(500 µg/ mL):* 5 mg compound **1n** were transferred into a 10 mL volumetric flask, 5 mL acetonitrile R were added, the flask was sonicated for 5 minutes, cooled to room temperature and diluted to volume with acetonitrile R.

Test solution (5 µg/ mL compound ***1h****, 5 µg/ mL compound* ***1j****, 5 µg/ mL compound* ***1i****, 5 µg/ mL compound* ***1m****, 5 µg/ mL compound* ***1g****, 5 µg/ mL compound* ***1n****)*: 0.25 mL of each stock solution were diluted to 25 mL with methanol R.

Test solution was prepared six times.

Procedure:

The following sequence was used: TS_1, TS_2, TS_3, TS_4, TS_5, TS_6,

Where:

TS_x – Test solution.

pharmaceutics

Results:

The experimental results are presented in the following tables (**Tables S2 – S4**).

Table S2. Precision results for compounds **1h**, **1j**, **1i**, **1m**, **1g**, **1n** – 5 µg/ mL solution – first day

**Precision (I) results for compounds 1h, 1j, 1i, 1m, 1g, 1n – 5 µg/ mL solution**												
	**Compound 1h**		**Compound 1j**		**Compound 1i**		**Compound 1m**		**Compound 1g**		**Compound 1n**	
Sample No.	Ret. Time [min.]	Peak Area [µV*sec]	Ret. Time [min.]	Peak Area [µV*sec]	Ret. Time [min.]	Peak Area [µV*sec]	Ret. Time [min.]	Peak Area [µV*sec]	Ret. Time [min.]	Peak Area [µV*sec]	Ret. Time [min.]	Peak Area [µV*sec]
1.	11.270	111,591	12.596	169,135	13.789	82,009	15.167	97,170	22.721	108,491	25.932	84,215
2.	11.272	111,110	12.602	168,465	13.801	82,735	15.168	96,179	22.742	109,245	25.950	84,630
3.	11.276	111,024	12.612	168,231	13.818	82,661	15.178	96,381	22.766	108,577	25.974	82,741
4.	11.281	110,663	12.614	168,785	13.816	82,849	15.190	95,668	22.768	107,246	25.991	83,968
5.	11.286	110,997	12.620	169,796	13.824	82,632	15.196	97,012	22.779	108,996	26.003	84,959
6.	11.293	110,417	12.632	166,835	13.836	82,261	15.197	96,243	22.789	106,276	26.013	83,531
Average	11.280	110,967	12.613	168,541	13.814	82,525	15.183	96,442	22.761	108,138	25.977	84,007
[1] SD	0.009	402	0.013	1000	0.017	321	0.014	560	0.025	1144	0.031	796
[2] RSD %	0.08	0.36	0.10	0.59	0.12	0.39	0.09	0.58	0.11	1.06	0.12	0.95

[1] Standard deviation
[2] Relative standard deviation, %

Table S3. Precision results for compounds **1h**, **1j**, **1i**, **1m**, **1g**, **1n** – 5 µg/ mL solution – different day

**Precision (II) results for compounds 1h, 1j, 1i, 1m, 1g, 1n – 5 µg/ mL solution**												
	**Compound 1h**		**Compound 1j**		**Compound 1i**		**Compound 1m**		**Compound 1g**		**Compound 1n**	
Sample No.	Ret. Time [min.]	Peak Area [µV*sec]	Ret. Time [min.]	Peak Area [µV*sec]	Ret. Time [min.]	Peak Area [µV*sec]	Ret. Time [min.]	Peak Area [µV*sec]	Ret. Time [min.]	Peak Area [µV*sec]	Ret. Time [min.]	Peak Area [µV*sec]
1.	11.142	110,842	12.456	168,780	13.649	80,744	15.006	97,663	22.563	106,865	25.801	83,077
2.	11.330	109,970	12.672	166,002	13.886	79,884	15.250	97,797	22.896	106,337	26.148	81,794
3.	11.306	109,387	12.646	165,117	13.853	79,171	15.209	96,058	22.831	104,014	26.056	82,753
4.	11.281	114,244	12.615	172,220	13.805	81,127	15.179	98,678	22.722	108,103	25.945	80,985
5.	11.261	111,967	12.585	166,791	13.783	82,099	15.138	98,075	22.681	108,183	25.875	84,121
6.	11.245	108,129	12.562	163,043	13.749	77,962	15.126	94,606	22.594	103,635	25.773	79,668
Average	11.261	110757	12.589	166992	13.787	80164	15.151	97146	22.714	106189	25.933	82066
[1] SD	0.066	2147	0.076	3183	0.084	1478	0.085	1519	0.131	1968	0.147	1594
[2] RSD %	0.58	1.94	0.61	1.91	0.61	1.84	0.56	1.56	0.57	1.85	0.57	1.94

[1] Standard deviation
[2] Relative standard deviation, %

Table S4. Intermediate precision results for compounds **1h**, **1j**, **1i**, **1m**, **1g**, **1n** – 5 µg/ mL solution

Precision results for compounds 1h, 1j, 1i, 1m, 1g, 1n – 5 µg/ mL solution												
	**Compound 1h**		**Compound 1j**		**Compound 1i**		**Compound 1m**		**Compound 1g**		**Compound 1n**	
Sample No.	Ret. Time [min.]	Peak Area [µV*sec]	Ret. Time [min.]	Peak Area [µV*sec]	Ret. Time [min.]	Peak Area [µV*sec]	Ret. Time [min.]	Peak Area [µV*sec]	Ret. Time [min.]	Peak Area [µV*sec]	Ret. Time [min.]	Peak Area [µV*sec]
1.	11.270	111,591	12.596	169,135	13.789	82,009	15.167	97,170	22.721	108,491	25.932	84,215
2.	11.272	111,110	12.602	168,465	13.801	82,735	15.168	96,179	22.742	109,245	25.950	84,630
3.	11.276	111,024	12.612	168,231	13.818	82,661	15.178	96,381	22.766	108,577	25.974	82,741
4.	11.281	110,663	12.614	168,785	13.816	82,849	15.190	95,668	22.768	107,246	25.991	83,968
5.	11.286	110,997	12.620	169,796	13.824	82,632	15.196	97,012	22.779	108,996	26.003	84,959
6.	11.293	110,417	12.632	166,835	13.836	82,261	15.197	96,243	22.789	106,276	26.013	83,531
7.	11.142	110,842	12.456	168,780	13.649	80,744	15.006	97,663	22.563	106,865	25.801	83,077
8.	11.330	109,970	12.672	166,002	13.886	79,884	15.250	97,797	22.896	106,337	26.148	81,794
9.	11.306	109,387	12.646	165,117	13.853	79,171	15.209	96,058	22.831	104,014	26.056	82,753
10.	11.281	114,244	12.615	172,220	13.805	81,127	15.179	98,678	22.722	108,103	25.945	80,985
11.	11.261	111,967	12.585	166,791	13.783	82,099	15.138	98,075	22.681	108,183	25.875	84,121
12.	11.245	108,129	12.562	163,043	13.749	77,962	15.126	94,606	22.594	103,635	25.773	79,668
Average	11.270	110,862	12.601	167,767	13.801	81,345	15.167	96,794	22.738	107,164	25.955	83,037
[1] SD	0.046	1476	0.054	2390	0.059	1600	0.060	1152	0.093	1842	0.104	1572
[2] RSD %	0.40	1.33	0.43	1.42	0.43	1.97	0.40	1.19	0.41	1.72	0.40	1.89

[1] Standard deviation

[2] Relative standard deviation, %

LOD

The limit of detection of an individual analytical method is the lowest quantity of an analyte that can be distinguished from a substance within a stated confidence limit.

The limit of detection was determined based on the calibration curve at low concentrations. Solutions of compounds **1h**, **1j**, **1i**, **1m**, **1g**, and **1n** were prepared and the calibration curve was traced. Limit of detection was estimated based on the following formula:

LOD=$\frac{F\times SD}{b}$, where:

F – 3.3 in case of LOD;

SD – standard deviation;

b – slope of the calibration curve.

LOQ

The limit of quantitation of an individual analytical method is the lowest quantity of an analyte that can be quantitated from a substance within a stated confidence limit.

The limit of quantitation was determined based on the calibration curve at low concentrations. Solutions of compounds **1h**, **1j**, **1i**, **1m**, **1g**, and **1n** were prepared and the calibration curve was traced. Limit of quantitation was estimated based on the following formula:

LOQ=$\frac{F\times SD}{b}$, where:

F – 10.0 in case of LOQ;

SD – standard deviation;

b – slope of the calibration curve.

<u>Preparation of solutions</u>:

In order to obtain the LOD and LOQ of the method for compounds **1h**, **1j**, **1i**, **1m**, **1g**, and **1n**, a calibration curve was created by preparing increasingly concentrated solutions from the *Stock Solution*.

<u>Procedure</u>:

Stock solutions of 500 µg/ mL are prepared from each of the tested compounds:

Stock solution ***1h*** *(500 µg/ mL):* 5 mg compound **1h** were transferred into a 10 mL volumetric flask, 5 mL acetonitrile R were added, the flask was sonicated for 5 minutes, cooled to room temperature and diluted to volume with acetonitrile R.

Stock solution ***1j*** *(500 µg/ mL):* 5 mg compound **1j** were transferred into a 10 mL volumetric flask, 5 mL acetonitrile R were added, the flask was sonicated for 5 minutes, cooled to room temperature and diluted to volume with acetonitrile R.

Stock solution ***1i*** *(500 µg/ mL):* 5 mg compound **1i** were transferred into a 10 mL volumetric flask, 5 mL acetonitrile R were added, the flask was sonicated for 5 minutes, cooled to room temperature and diluted to volume with acetonitrile R.

Stock solution ***1m*** *(500 µg/ mL):* 5 mg compound **1m** were transferred into a 10 mL volumetric flask, 5 mL acetonitrile R were added, the flask was sonicated for 5 minutes, cooled to room temperature and diluted to volume with acetonitrile R.

Stock solution ***1g*** *(500 µg/ mL):* 5 mg compound **1g** were transferred into a 10 mL volumetric flask, 5 mL acetonitrile R were added, the flask was sonicated for 5 minutes, cooled to room temperature and diluted to volume with acetonitrile R.

Stock solution ***1n*** *(500 µg/ mL):* 5 mg compound **1n** were transferred into a 10 mL volumetric flask, 5 mL acetonitrile R were added, the flask was sonicated for 5 minutes, cooled to room temperature and diluted to volume with acetonitrile R.

Cumulative stock solution (4.0 µg/mL 1h, 4.0 µg/mL 1j, 4.0 µg/mL 1i, 4.0 µg/mL 1m, 4.0 µg/mL 1g, 4.0 µg/mL 1n): 0.2 mL of each *Stock Solution* were diluted to 25 mL with methanol R.

LOD-LOQ Solution 1, 1.2% (0.06 µg/mL 1h, 0.06 µg/mL 1j, 0.06 µg/mL 1i, 0.06 µg/mL 1m, 0.06 µg/mL 1g, 0.06 µg/mL 1n): 0.15 mL of the *Cumulative stock solution* were diluted to 10 mL with methanol R.

LOD-LOQ Solution 2, 1.6% (0.08 µg/mL 1h, 0.08 µg/mL 1j, 0.08 µg/mL 1i, 0.08 µg/mL 1m, 0.08 µg/mL 1g, 0.08 µg/mL 1n): 0.2 mL of the *Cumulative stock solution* were diluted to 10 mL with methanol R.

LOD-LOQ Solution 3, 2% (0.1 µg/mL 1h, 0.1 µg/mL 1j, 0.1 µg/mL 1i, 0.1 µg/mL 1m, 0.1 µg/mL 1g, 0.1 µg/mL 1n): 0.5 mL of each *Cumulative stock solution* were diluted to 20 mL with methanol R.

LOD-LOQ Solution 4, 4% (0.2 µg/mL 1h, 0.2 µg/mL 1j, 0.2 µg/mL 1i, 0.2 µg/mL 1m, 0.2 µg/mL 1g, 0.2

μg/mL 1n): 1.0 mL of each *Stock Solution* were diluted to 20 mL with methanol R.

LOD-LOQ Solution 5, 10% (0.5 μg/mL 1h, 0.5 μg/mL 1j, 0.5 μg/mL 1i, 0.5 μg/mL 1m, 0.5 μg/mL 1g, 0.5 μg/mL 1n): 0.1 mL of each *Stock Solution* were diluted to 100 mL with methanol R.

The following sequence was used: DQS_1, DQS_2, DQS_3, DQS_4, DQS_5,

where:

DQS = Limit of detection/limit of quantitation solution.

Results:

The obtained results for the LOD-LOQ related to compounds **1h**, **1j**, **1i**, **1m**, **1g**, **1n** are presented in **Table S5**.

The R^2 correlation coefficient of each LOD-LOQ calibration curve is greater than 0.99 (**Figure S12**), in compliance with the acceptance criteria.

Table S5. LOD-LOQ results for compounds **1h**, **1j**, **1i**, **1m**, **1g**, and **1n**, expressed in µg/ mL and % (considering 100% a solution of 5 µg/ mL of each tested compound)

Compound	STDEV	SLOPE	LOD		LOQ	
			µg/ mL	%	µg/ mL	%
**1h**	181.72	23750.89	0.0252	0.504	0.0765	1.530
**1j**	183.68	26862.68	0.0205	0.410	0.0684	1.368
**1i**	118.33	19265.90	0.0184	0.368	0.0614	1.228
**1m**	139.32	18696.35	0.0224	0.448	0.0745	1.490
**1g**	127.23	16188.43	0.0236	0.472	0.0786	1.572
**1n**	110.25	13827.25	0.0239	0.478	0.0797	1.594

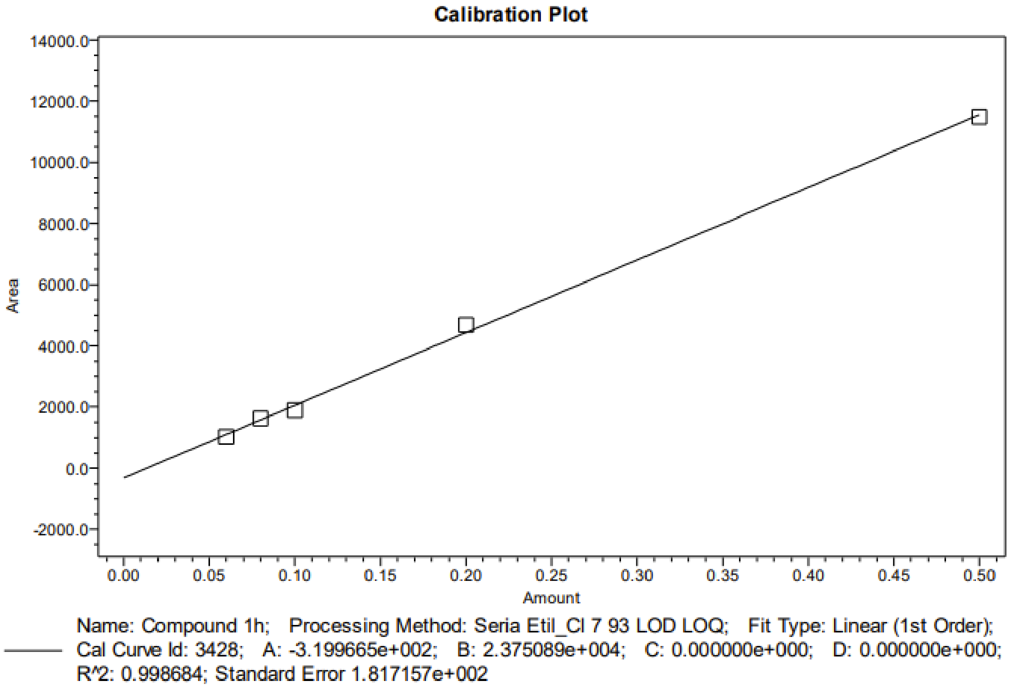
Calibration Plot
Area
Amount
14000.0
12000.0
10000.0
8000.0
6000.0
4000.0
2000.0
0.0
-2000.0
0.00 0.05 0.10 0.15 0.20 0.25 0.30 0.35 0.40 0.45 0.50
Name: Compound 1h; Processing Method: Seria Etil_Cl 7 93 LOD LOQ; Fit Type: Linear (1st Order);
Cal Curve Id: 3428; A: -3.199665e+002; B: 2.375089e+004; C: 0.000000e+000; D: 0.000000e+000;
R^2: 0.998684; Standard Error 1.817157e+002

(a)

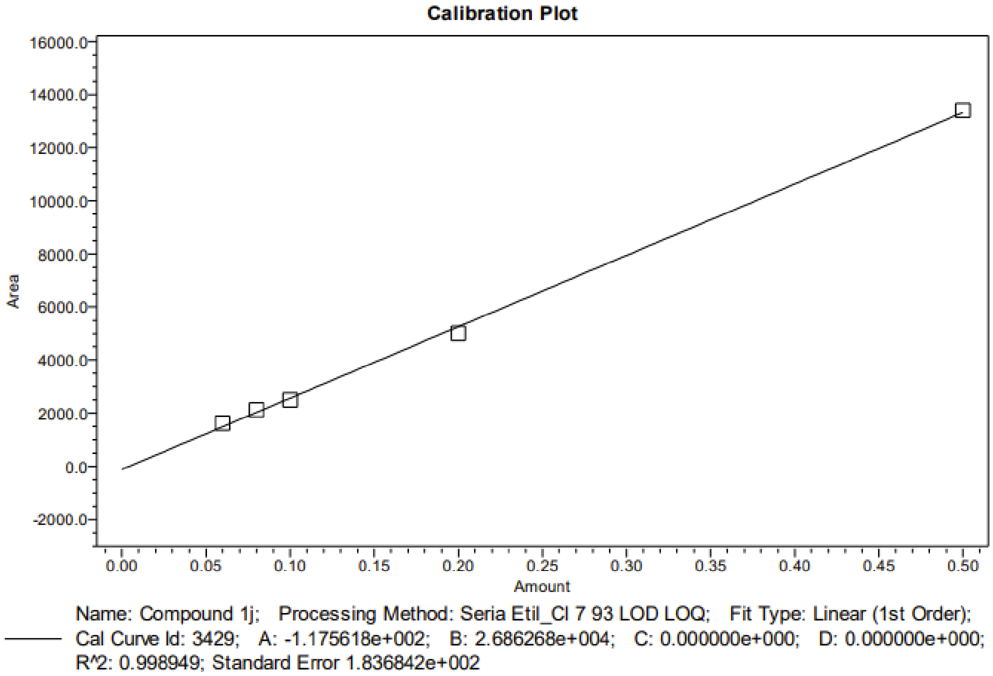
Calibration Plot
Area
Amount
16000.0
14000.0
12000.0
10000.0
8000.0
6000.0
4000.0
2000.0
0.0
-2000.0
0.00 0.05 0.10 0.15 0.20 0.25 0.30 0.35 0.40 0.45 0.50
Name: Compound 1j; Processing Method: Seria Etil_Cl 7 93 LOD LOQ; Fit Type: Linear (1st Order);
Cal Curve Id: 3429; A: -1.175618e+002; B: 2.686268e+004; C: 0.000000e+000; D: 0.000000e+000;
R^2: 0.998949; Standard Error 1.836842e+002

(b)

pharmaceutics

MDPI

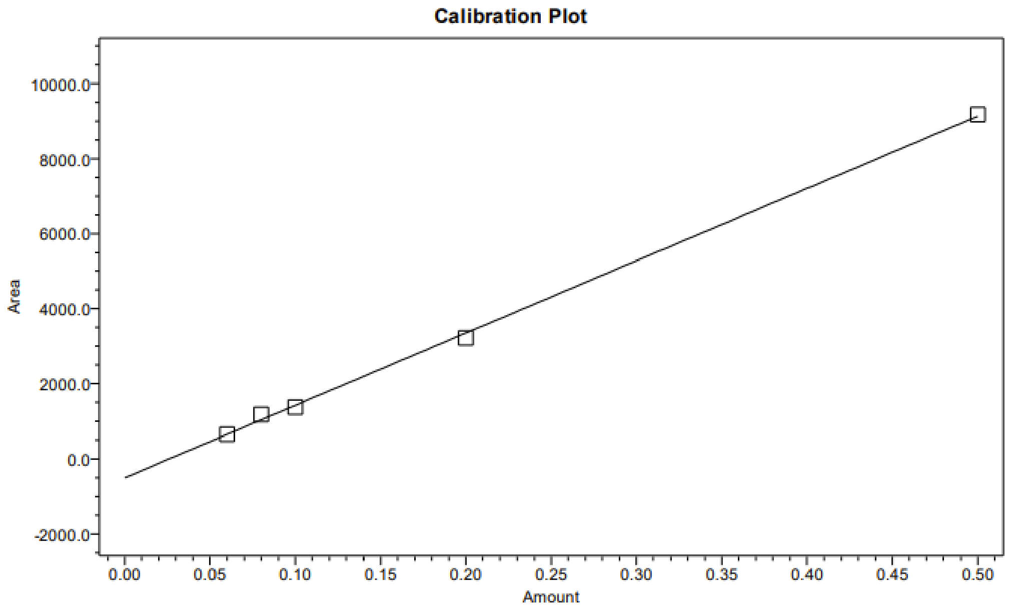

Name: Compound 1i; Processing Method: Seria Etil_Cl 7 93 LOD LOQ; Fit Type: Linear (1st Order); Cal Curve Id: 3430; A: -5.017253e+002; B: 1.926590e+004; C: 0.000000e+000; D: 0.000000e+000; R^2: 0.999152; Standard Error 1.183270e+002

(c)

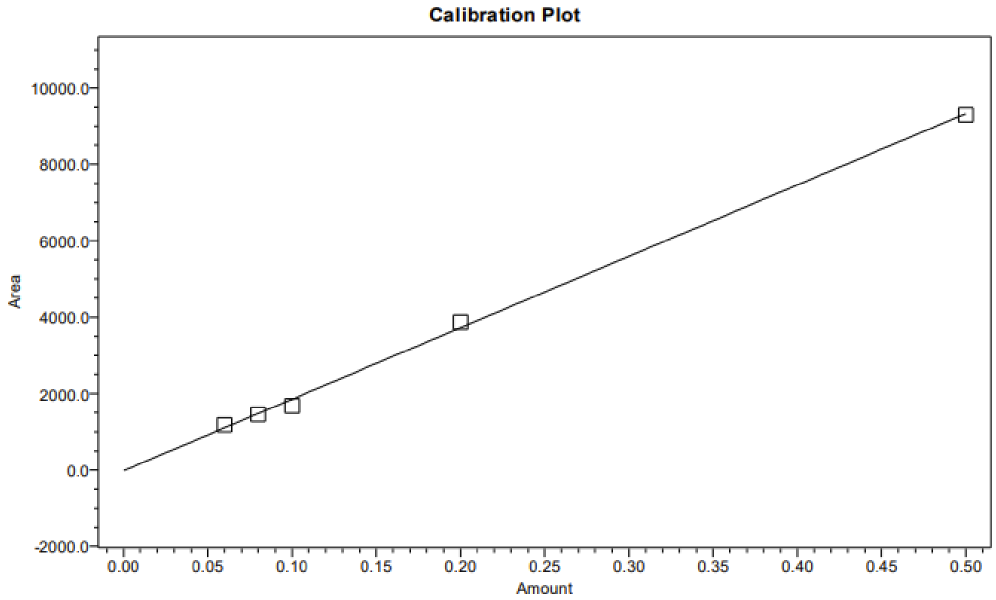

Name: Compound 1m; Processing Method: Seria Etil_Cl 7 93 LOD LOQ; Fit Type: Linear (1st Order); Cal Curve Id: 3431; A: -1.678743e+001; B: 1.869635e+004; C: 0.000000e+000; D: 0.000000e+000; R^2: 0.998752; Standard Error 1.393188e+002

(d)

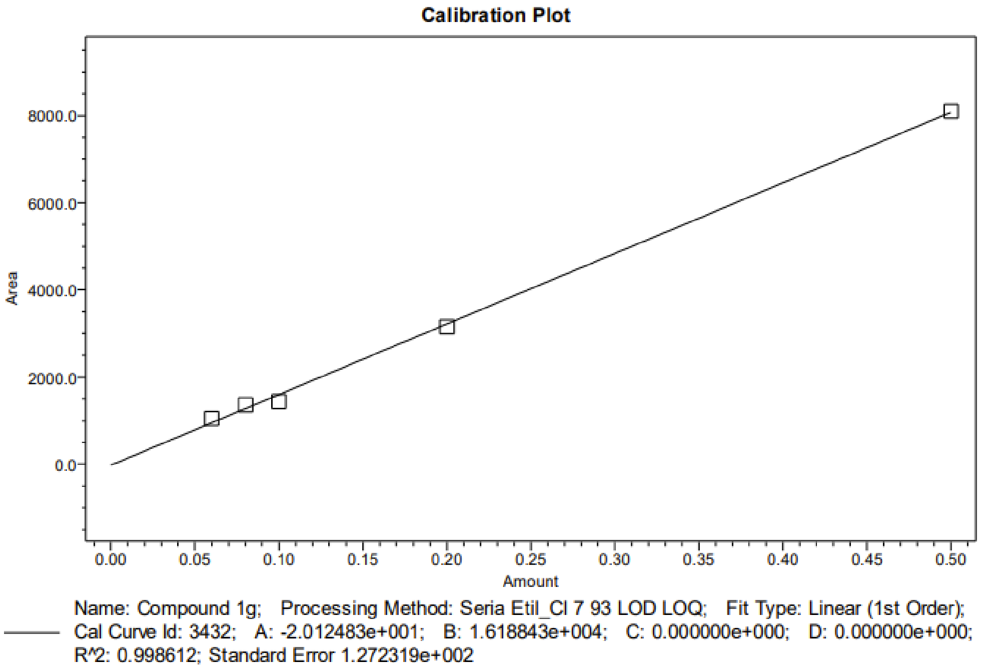

(e)

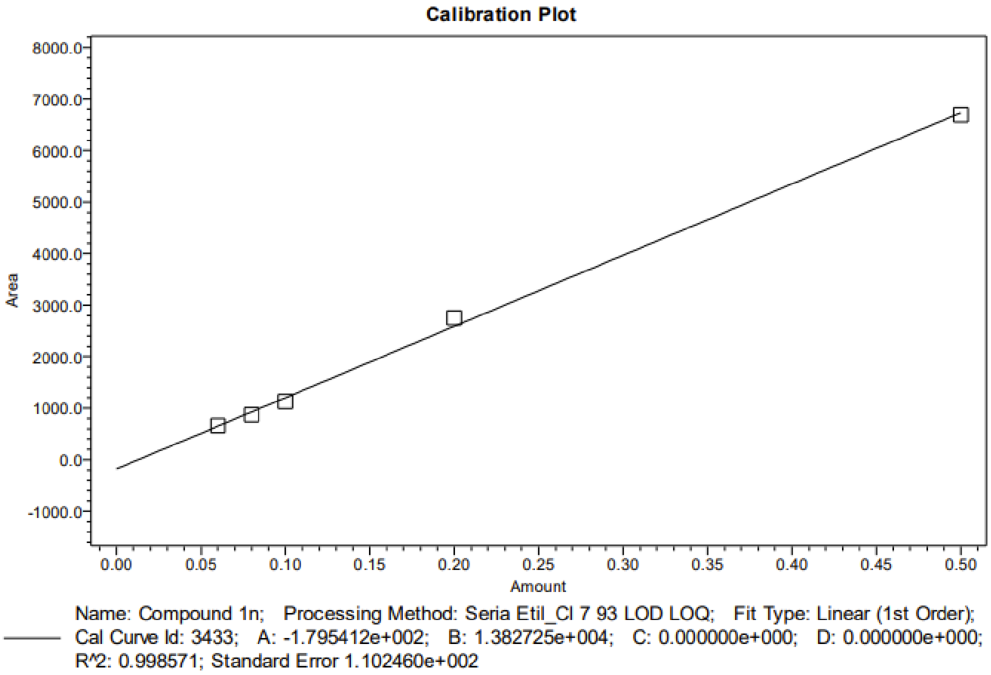

(f)

Figure S12. LOD-LOQ Calibration Curves for **(a) 1h**, amount in µg/mL; **(b) 1j**, amount in µg/mL; **(c) 1i** amount in µg/mL; **(d) 1m**, amount in µg/mL; **(e) 1g**, amount in µg/mL; **(f) 1n**, amount in µg/mL.

LINEARITY

The linearity of an analytical procedure represents its ability (within a given range) to obtain test results which are directly proportional to the concentration of analyte in the analyzed sample.

Linearity range:

The range of an analytical procedure is the interval between the upper and lower concentration (amounts) of analyte in the sample (including these concentrations) for which it has been demonstrated that the analytical procedure has a suitable level of precision, accuracy and linearity.

The linearity of the method is demonstrated within the concentration range of the theoretical working concentration (LOQ – 6.0 µg/ mL compounds **1h**, **1j**, **1i**, **1m**, **1g**, and **1n**).

Preparation of solutions:

In order to demonstrate the linearity of the method, a calibration curve was created by preparing increasingly concentrated solutions. The range of concentrations was LOQ – 6.0 µg/ mL compounds **1h**, **1j**, **1i**, **1m**, **1g**, and **1n**.

Stock solutions of 500 µg/ mL are prepared from each of the tested compounds:

*Stock solution **1h** (500 µg/ mL):* 5 mg compound **1h** were transferred into a 10 mL volumetric flask, 5 mL acetonitrile R were added, the flask was sonicated for 5 minutes, cooled to room temperature and diluted to volume with acetonitrile R.

*Stock solution **1j** (500 µg/ mL):* 5 mg compound **1j** were transferred into a 10 mL volumetric flask, 5 mL acetonitrile R were added, the flask was sonicated for 5 minutes, cooled to room temperature and diluted to volume with acetonitrile R.

*Stock solution **1i** (500 µg/ mL):* 5 mg compound **1i** were transferred into a 10 mL volumetric flask, 5 mL acetonitrile R were added, the flask was sonicated for 5 minutes, cooled to room temperature and diluted to volume with acetonitrile R.

*Stock solution **1m** (500 µg/ mL):* 5 mg compound **1m** were transferred into a 10 mL volumetric flask, 5 mL acetonitrile R were added, the flask was sonicated for 5 minutes, cooled to room temperature and diluted to volume with acetonitrile R.

*Stock solution **1g** (500 µg/ mL):* 5 mg compound **1g** were transferred into a 10 mL volumetric flask, 5 mL acetonitrile R were added, the flask was sonicated for 5 minutes, cooled to room temperature and diluted to volume with acetonitrile R.

*Stock solution **1n** (500 µg/ mL):* 5 mg compound **1n** were transferred into a 10 mL volumetric flask, 5

mL acetonitrile R were added, the flask was sonicated for 5 minutes, cooled to room temperature and diluted to volume with acetonitrile R.

Linearity Solution 1, 1.2% (0.06 µg/mL 1h, 0.06 µg/mL 1j, 0.06 µg/mL 1i, 0.06 µg/mL 1m, 0.06 µg/mL 1g, 0.06 µg/mL 1n): 0.2 mL of each *Stock Solution* were diluted to 25 mL with methanol R. 0.15 mL of the obtained solution were diluted to 10 mL with methanol R.

Linearity Solution 2, 1.6% (0.08 µg/mL 1h, 0.08 µg/mL 1j, 0.08 µg/mL 1i, 0.08 µg/mL 1m, 0.08 µg/mL 1g, 0.08 µg/mL 1n): 0.2 mL of each *Stock Solution* were diluted to 25 mL with methanol R. 0.2 mL of the obtained solution were diluted to 10 mL with methanol R.

Linearity Solution 3, 2% (0.1 µg/mL 1h, 0.1 µg/mL 1j, 0.1 µg/mL 1i, 0.1 µg/mL 1m, 0.1 µg/mL 1g, 0.1 µg/mL 1n): 0.2 mL of each *Stock Solution* were diluted to 25 mL with methanol R. 0.5 mL of the obtained solution were diluted to 20 mL with methanol R.

Linearity Solution 4, 4% (0.2 µg/mL 1h, 0.2 µg/mL 1j, 0.2 µg/mL 1i, 0.2 µg/mL 1m, 0.2 µg/mL 1g, 0.2 µg/mL 1n): 0.2 mL of each *Stock Solution* were diluted to 25 mL with methanol R. 1.0 mL of the obtained solution were diluted to 20 mL with methanol R.

Linearity Solution 5, 10% (0.5 µg/mL 1h, 0.5 µg/mL 1j, 0.5 µg/mL 1i, 0.5 µg/mL 1m, 0.5 µg/mL 1g, 0.5 µg/mL 1n): 0.1 mL of each *Stock Solution* were diluted to 100 mL with methanol R.

Linearity Solution 5, 80% (4.0 µg/mL 1h, 4.0 µg/mL 1j, 4.0 µg/mL 1i, 4.0 µg/mL 1m, 4.0 µg/mL 1g, 4.0 µg/mL 1n): 0.20 mL of each *Stock Solution* were diluted to 25 mL with methanol R.

Linearity Solution 5, 100% (5.0 µg/mL 1h, 5.0 µg/mL 1j, 5.0 µg/mL 1i, 5.0 µg/mL 1m, 5.0 µg/mL 1g, 5.0 µg/mL 1n): 0.25 mL of each *Stock Solution* were diluted to 25 mL with methanol R.

Linearity Solution 5, 120% (6.0 µg/mL 1h, 6.0 µg/mL 1j, 6.0 µg/mL 1i, 6.0 µg/mL 1m, 6.0 µg/mL 1g, 6.0 µg/mL 1n): 0.30 mL of each *Stock Solution* were diluted to 25 mL with methanol R.

The following sequence was used: $SL_1, SL_2, SL_3, SL_4, SL_5, SL_6, SL_7, SL_8,$ where:

SL = linearity solution.

pharmaceutics

MDPI

Results:

The linearity results are presented in the **Table S6**.

The R^2 correlation coefficient of each linearity plot is greater than 0.99 (**Figure S13**), in compliance with the acceptance criteria.

Table S6. Linearity results for compounds **1h, 1j, 1i, 1m, 1g,** and **1n**

Level [%]	Concentration [µg/ mL]	Peak areas [µV*sec] of the evaluated compounds					
		**1h**	**1j**	**1i**	**1m**	**1g**	**1n**
**1.2**	0.06	1034	1630	507	1178	1053	658
**1.6**	0.08	1627	2133	1180	1459	1365	872
**2.0**	0.10	1897	2500	1374	1682	1438	1130
**4.0**	0.20	4680	5007	3217	3871	3157	2753
**10.0**	0.50	11,488	13,394	9171	9300	8106	6692
**80.0**	4.00	894,36	135,007	69,752	78,851	90,083	73,829
**100.0**	5.00	110,698	166,926	85,573	97,456	112,175	90,045
**120.0**	6.00	133,370	200,966	102,149	118,926	133,117	10,8604

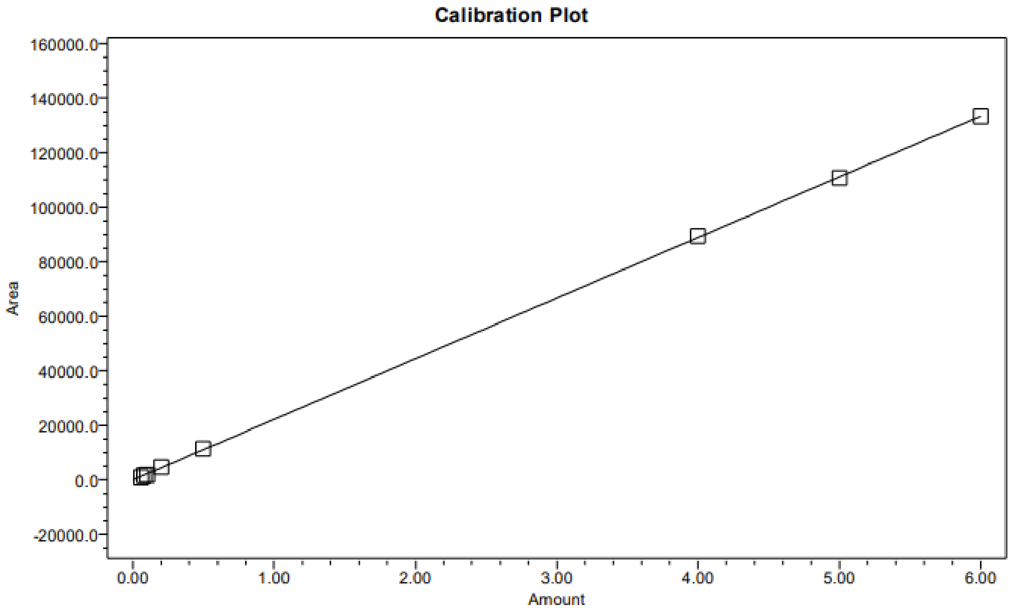

(a)

pharmaceutics

MDPI

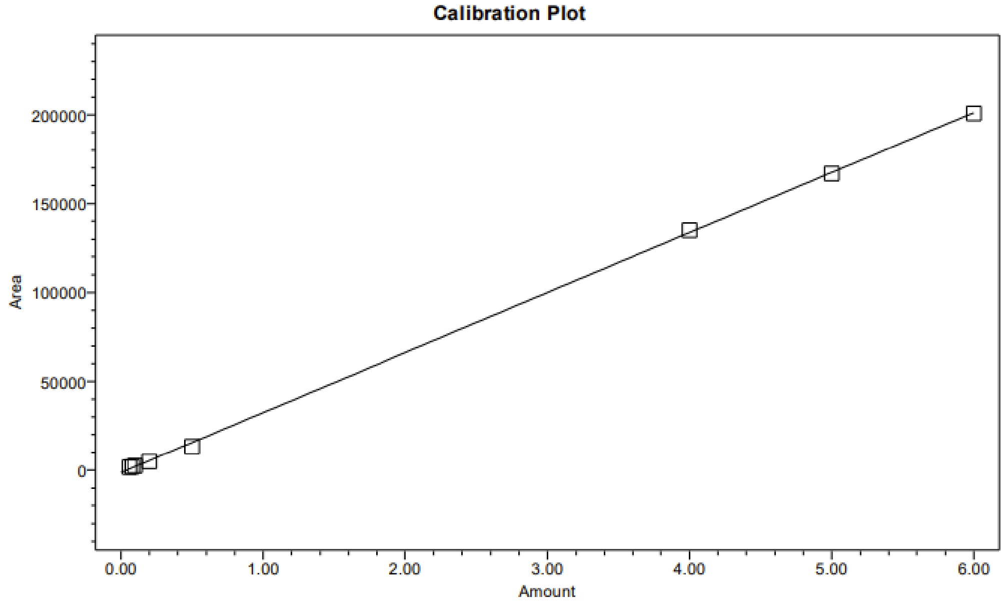

(b)

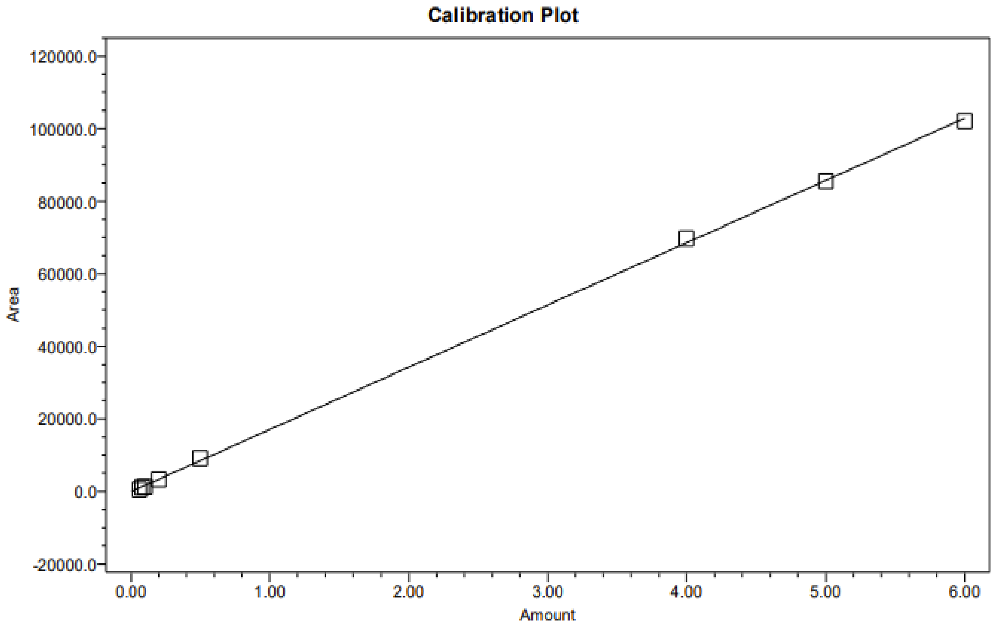

(c)

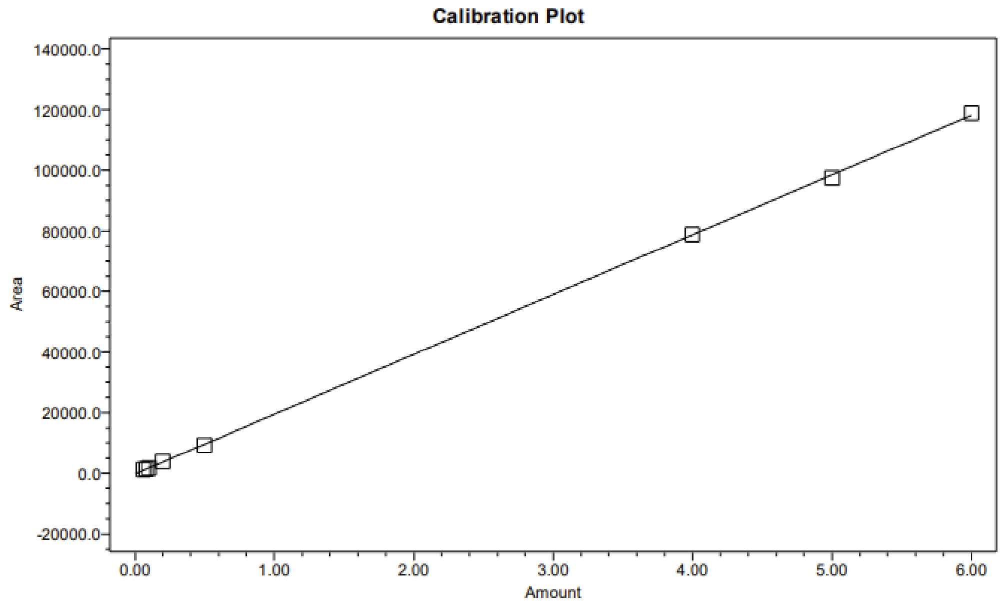

Calibration Plot
140000.0
120000.0
100000.0
80000.0
60000.0
40000.0
20000.0
0.0
-20000.0
Area
0.00
1.00
2.00
3.00
4.00
5.00
6.00
Amount

(d)

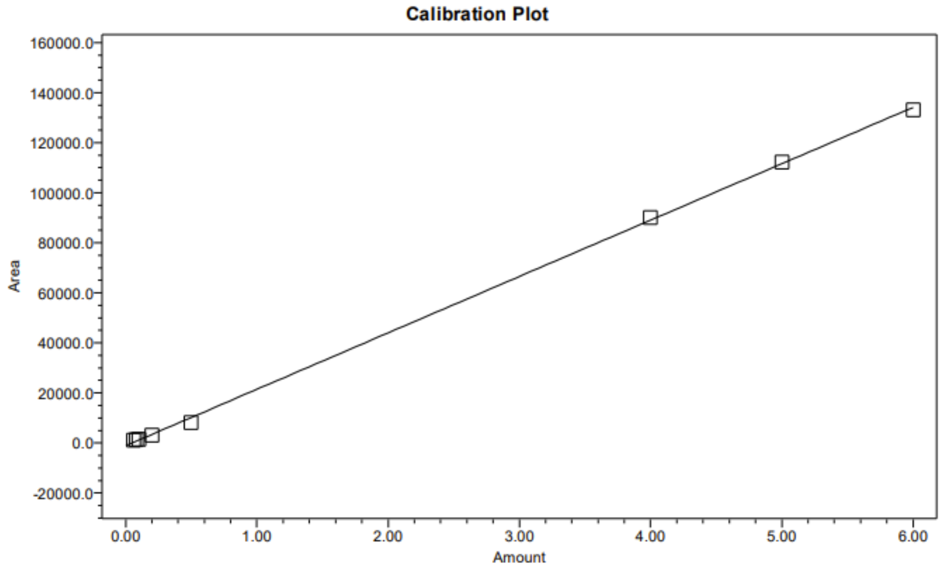

Calibration Plot
160000.0
140000.0
120000.0
100000.0
80000.0
60000.0
40000.0
20000.0
0.0
-20000.0
Area
0.00
1.00
2.00
3.00
4.00
5.00
6.00
Amount

(e)

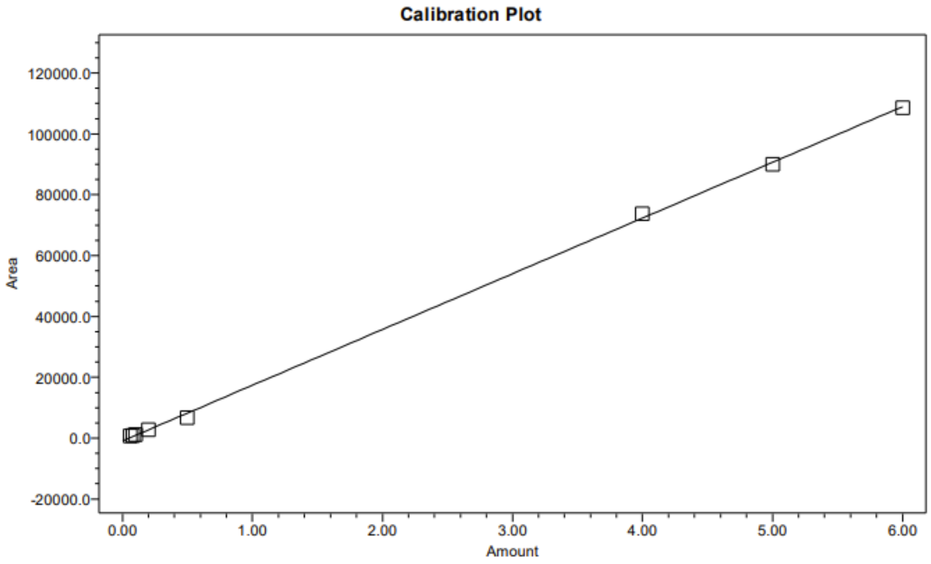

(f)

Figure S13. Linearity Plots extracted from Empower 3 software, for **(a) 1h** (R^2 = 0.9999; B = 22233.20); **(b) 1j** (R^2 = 0.9998; B = 1191.98); **(c) 1i** (R^2 = 0.9998; B = 673.52); **(d) 1m** (R^2 = 0.9999; B = 541.35); **(e) 1g** (R^2 = 0.9997; B = 1147.85); **(f) 1n** (R^2 = 0.9996; B = 957.13).

pharmaceutics

ACCURACY

The accuracy of an analytical procedure expresses the closeness of agreement between the value which is accepted either as a conventional true value or an accepted reference value and the value found.

In order to demonstrate the accuracy of the method, three concentration levels (low, medium and high concentration) were prepared and analyzed.

Preparation of solutions:

Stock solutions of 500 µg/ mL are prepared from each of the tested compounds:

*Stock solution **1h** (500 µg/ mL):* 5.00 mg compound **1h** were transferred into a 10 mL volumetric flask, 5 mL acetonitrile R were added, the flask was sonicated for 5 minutes, cooled to room temperature and diluted to volume with acetonitrile R.

*Stock solution **1j** (502 µg/ mL):* 5.02 mg compound **1j** were transferred into a 10 mL volumetric flask, 5 mL acetonitrile R were added, the flask was sonicated for 5 minutes, cooled to room temperature and diluted to volume with acetonitrile R.

*Stock solution **1i** (505 µg/ mL):* 5.05 mg compound **1i** were transferred into a 10 mL volumetric flask, 5 mL acetonitrile R were added, the flask was sonicated for 5 minutes, cooled to room temperature and diluted to volume with acetonitrile R.

*Stock solution **1m** (498 µg/ mL):* 4.98 mg compound **1m** were transferred into a 10 mL volumetric flask, 5 mL acetonitrile R were added, the flask was sonicated for 5 minutes, cooled to room temperature and diluted to volume with acetonitrile R.

*Stock solution **1g** (508 µg/ mL):* 5.08 mg compound **1g** were transferred into a 10 mL volumetric flask, 5 mL acetonitrile R were added, the flask was sonicated for 5 minutes, cooled to room temperature and diluted to volume with acetonitrile R.

*Stock solution **1n** (525 µg/ mL):* 5.25 mg compound **1n** were transferred into a 10 mL volumetric flask, 5 mL acetonitrile R were added, the flask was sonicated for 5 minutes, cooled to room temperature and diluted to volume with acetonitrile R.

*Standard solution (5 µg/ mL compound **1h**, 5 µg/ mL compound **1j**, 5 µg/ mL compound **1i**, 5 µg/ mL compound **1m**, 5 µg/ mL compound **1g**, 5 µg/ mL compound **1n**)*: 0.25 mL of each stock solution were diluted to 25 mL with methanol R.

Accuracy – 80%:

*Stock solution **1h** (502 µg/ mL):* 5.02 mg compound **1h** were transferred into a 10 mL volumetric flask, 5 mL acetonitrile R were added, the flask was sonicated for 5 minutes, cooled to room temperature and diluted to volume with acetonitrile R.

*Stock solution **1j** (502 µg/ mL):* 5.02 mg compound **1j** were transferred into a 10 mL volumetric flask, 5 mL acetonitrile R were added, the flask was sonicated for 5 minutes, cooled to room temperature and diluted to volume with acetonitrile R.

*Stock solution **1i** (501 µg/ mL):* 5.01 mg compound **1i** were transferred into a 10 mL volumetric flask, 5 mL acetonitrile R were added, the flask was sonicated for 5 minutes, cooled to room temperature and diluted to volume with acetonitrile R.

*Stock solution **1m** (500 µg/ mL):* 5.00 mg compound **1m** were transferred into a 10 mL volumetric flask, 5 mL acetonitrile R were added, the flask was sonicated for 5 minutes, cooled to room temperature and diluted to volume with acetonitrile R.

*Stock solution **1g** (503 µg/ mL):* 5.03 mg compound **1g** were transferred into a 10 mL volumetric flask, 5 mL acetonitrile R were added, the flask was sonicated for 5 minutes, cooled to room temperature and diluted to volume with acetonitrile R.

*Stock solution **1n** (514 µg/ mL):* 5.14 mg compound **1n** were transferred into a 10 mL volumetric flask, 5 mL acetonitrile R were added, the flask was sonicated for 5 minutes, cooled to room temperature and diluted to volume with acetonitrile R.

*Test solution accuracy 1 – 80% (4 µg/ mL compound **1h**, 4 µg/ mL compound **1j**, 4 µg/ mL compound **1i**, 4 µg/ mL compound **1m**, 4 µg/ mL compound **1g**, 4 µg/ mL compound **1n**)*: 0.20 mL of each stock solution were diluted to 25 mL with methanol R.

Test solution accuracy 1 – 80% was prepared six times.

Accuracy – 100%:

*Stock solution **1h** (500 µg/ mL):* 5.00 mg compound **1h** were transferred into a 10 mL volumetric flask, 5 mL acetonitrile R were added, the flask was sonicated for 5 minutes, cooled to room temperature and diluted to volume with acetonitrile R.

*Stock solution **1j** (500 µg/ mL):* 5.00 mg compound **1j** were transferred into a 10 mL volumetric flask, 5 mL acetonitrile R were added, the flask was sonicated for 5 minutes, cooled to room temperature and diluted to volume with acetonitrile R.

*Stock solution **1i** (495 µg/ mL):* 4.95 mg compound **1i** were transferred into a 10 mL volumetric flask, 5 mL acetonitrile R were added, the flask was sonicated for 5 minutes, cooled to room temperature and diluted to volume with acetonitrile R.

*Stock solution **1m** (501 µg/ mL):* 5.01 mg compound **1m** were transferred into a 10 mL volumetric flask, 5 mL acetonitrile R were added, the flask was sonicated for 5 minutes, cooled to room temperature and diluted to volume with acetonitrile R.

*Stock solution **1g** (498 µg/ mL):* 4.98 mg compound **1g** were transferred into a 10 mL volumetric flask, 5 mL acetonitrile R were added, the flask was sonicated for 5 minutes, cooled to room temperature and diluted to volume with acetonitrile R.

*Stock solution **1n** (494 µg/ mL):* 4.94 mg compound **1n** were transferred into a 10 mL volumetric flask, 5 mL acetonitrile R were added, the flask was sonicated for 5 minutes, cooled to room temperature and diluted to volume with acetonitrile R.

*Test solution accuracy 2 – 100%: (5 µg/ mL compound **1h**, 5 µg/ mL compound **1j**, 5 µg/ mL compound **1i**, 5 µg/ mL compound **1m**, 5 µg/ mL compound **1g**, 5 µg/ mL compound **1n**)*: 0.25 mL of each stock solution were diluted to 25 mL with methanol R.

Test solution accuracy 2 – 100% was prepared six times.

Accuracy – 120%:

Stock solution ***1h*** *(501 µg/ mL):* 5.01 mg compound **1h** were transferred into a 10 mL volumetric flask, 5 mL acetonitrile R were added, the flask was sonicated for 5 minutes, cooled to room temperature and diluted to volume with acetonitrile R.

Stock solution ***1j*** *(502 µg/ mL):* 5.02 mg compound **1j** were transferred into a 10 mL volumetric flask, 5 mL acetonitrile R were added, the flask was sonicated for 5 minutes, cooled to room temperature and diluted to volume with acetonitrile R.

Stock solution ***1i*** *(498 µg/ mL):* 4.98 mg compound **1i** were transferred into a 10 mL volumetric flask, 5 mL acetonitrile R were added, the flask was sonicated for 5 minutes, cooled to room temperature and diluted to volume with acetonitrile R.

Stock solution ***1m*** *(511 µg/ mL):* 5.11 mg compound **1m** were transferred into a 10 mL volumetric flask, 5 mL acetonitrile R were added, the flask was sonicated for 5 minutes, cooled to room temperature and diluted to volume with acetonitrile R.

Stock solution ***1g*** *(502 µg/ mL):* 5.02 mg compound **1g** were transferred into a 10 mL volumetric flask, 5 mL acetonitrile R were added, the flask was sonicated for 5 minutes, cooled to room temperature and diluted to volume with acetonitrile R.

Stock solution ***1n*** *(508 µg/ mL):* 5.08 mg compound **1n** were transferred into a 10 mL volumetric flask, 5 mL acetonitrile R were added, the flask was sonicated for 5 minutes, cooled to room temperature and diluted to volume with acetonitrile R.

Test solution accuracy 3 – 120% (6 µg/ mL compound ***1h****, 6 µg/ mL compound* ***1j****, 6 µg/ mL compound* ***1i****, 6 µg/ mL compound* ***1m****, 6 µg/ mL compound* ***1g****, 6 µg/ mL compound* ***1n****)*: 0.30 mL of each stock solution were diluted to 25 mL with methanol R.

Test solution accuracy 3 – 120% was prepared six times.

Procedure:

The following sequence was used: SS, SS, SS, SA_1, SA_1, SA_1, SA_1, SA_1, SA_1, SA_2, SA_2, SA_2, SA_2, SA_2, SA_2, SA_3, SA_3, SA_3, SA_3, SA_3, SA_3,

where:- SS – standard solution;

- SA – test solution;

Data calculation:

$$\text{Absolute recovery (\%)} = \frac{\text{Recovered concentration}}{\text{Theoretical concentration}} \times 100.$$

$$\text{Theoretical concentration (80\%), µg/ mL} = \frac{S_m}{10} \times \frac{0.2}{25} \times 1000 = S_m \times 0.0008 \times 1000 = S_m \times 0.8,$$

$$\text{Theoretical concentration (100\%), µg/ mL} = \frac{S_m}{10} \times \frac{0.25}{25} \times 1000 = S_m \times 0.001 \times 1000 = S_m \times 1,$$

$$\text{Theoretical concentration (120\%), µg/ mL} = \frac{S_m}{10} \times \frac{0.3}{25} \times 1000 = S_m \times 0.0012 \times 1000 = S_m \times 1.2,$$

where,

S_m = sample mass, mg;

0.0008, 0.001, 0.0012 = dilution factors;

1000 = conversion from mg to µg.

$$\text{Recovered concentration, µg/ mL} = \frac{A_P}{A_S} \times Ref_m \times \frac{0.25}{10} \times \frac{1000}{25} = \frac{A_P}{A_S} \times Ref_m \times 0.001 \times 1000,$$

where,

Ref_m = reference mass, mg;

A_P = Area of the peak due to each evaluated compound, in the chromatogram obtained with sample solution,

A_S = Average area of the peak due to each evaluated compound in the chromatogram obtained with standard solution,

0.001= dilution factor;

1000 = conversion from mg to µg.

pharmaceutics

Results:

The experimental results are presented in the **Tables S7 – S12**.

Table S7. Accuracy levels – real (theoretical) and determined concentrations of compound **1h** and mean percent recovery (%) of six injections of each accuracy concentration (80%, 100%, and 120% analytical concentrations)

Sample No.	**Compound 1h concentration**								
	**4 µg/ mL solution**			**5 µg/ mL solution**			**6 µg/ mL solution**		
	**Real [µg/ mL]**	**Determined [µg/ mL]**	**Recovery [%]**	**Real [µg/ mL]**	**Determined [µg/ mL]**	**Recovery, [%]**	**Real [µg/ mL]**	**Determined [µg/ mL]**	**Recovery, [%]**
1.	4.0160	3.9941	99.45	5.0000	5.0308	100.62	6.0120	5.9875	99.59
2.	4.0160	4.0314	100.38	5.0000	5.0091	100.18	6.0120	6.0257	100.23
3.	4.0160	3.9790	99.08	5.0000	5.0052	100.10	6.0120	6.0517	100.66
4.	4.0160	3.9763	99.01	5.0000	4.9889	99.78	6.0120	6.0198	100.13
5.	4.0160	4.0117	99.89	5.0000	5.0040	100.08	6.0120	6.0648	100.88
6.	4.0160	3.9536	98.45	5.0000	4.9778	99.56	6.0120	6.0027	99.84
Average:			99.88						
Standard Deviation:			0.62						
Relative standard deviation (RSD%):			0.62						
Confidence interval (Probability 95%):			99.60 – 100.17						

Table S8. Accuracy levels – real (theoretical) and determined concentrations of compound **1j** and mean percent recovery (%) of six injections of each accuracy concentration (80%, 100%, and 120% analytical concentrations)

Sample No.	**Compound 1j concentration**								
	**4 µg/ mL solution**			**5 µg/ mL solution**			**6 µg/ mL solution**		
	**Real [µg/ mL]**	**Determined [µg/ mL]**	**Recovery [%]**	**Real [µg/ mL]**	**Determined [µg/ mL]**	**Recovery, [%]**	**Real [µg/ mL]**	**Determined [µg/ mL]**	**Recovery, [%]**
1.	4.0160	4.0301	100.35	5.0000	5.0571	101.14	6.0240	6.0375	100.22
2.	4.0160	4.0712	101.37	5.0000	5.0371	100.74	6.0240	6.0667	100.71
3.	4.0160	4.0537	100.94	5.0000	5.0301	100.60	6.0240	6.0223	99.97
4.	4.0160	4.0186	100.06	5.0000	5.0466	100.93	6.0240	6.0419	100.30
5.	4.0160	4.0491	100.82	5.0000	5.0769	101.54	6.0240	6.0723	100.80
6.	4.0160	3.9742	98.96	5.0000	4.9883	99.77	6.0240	6.0228	99.98
Average:			100.51						
Standard Deviation:			0.63						
Relative standard deviation (RSD%):			0.63						
Confidence interval (Probability 95%):			100.22 – 100.81						

pharmaceutics MDPI

Table S9. Accuracy levels – real (theoretical) and determined concentrations of compound **1i** and mean percent recovery (%) of six injections of each accuracy concentration (80%, 100%, and 120% analytical concentrations)

Sample No.	Compound 1i concentration								
	4 µg/ mL solution			5 µg/ mL solution			6 µg/ mL solution		
	Real [µg/ mL]	Determined [µg/ mL]	Recovery [%]	Real [µg/ mL]	Determined [µg/ mL]	Recovery, [%]	Real [µg/ mL]	Determined [µg/ mL]	Recovery, [%]
1.	4.0080	3.9700	99.05	4.9500	4.8641	98.26	5.9760	6.0261	100.84
2.	4.0080	4.0130	100.13	4.9500	4.9072	99.13	5.9760	6.0114	100.59
3.	4.0080	3.9996	99.79	4.9500	4.9028	99.05	5.9760	5.9827	100.11
4.	4.0080	3.9988	99.77	4.9500	4.9139	99.27	5.9760	6.0559	101.34
5.	4.0080	4.0086	100.01	4.9500	4.9011	99.01	5.9760	6.0050	100.48
6.	4.0080	3.8852	96.94	4.9500	4.8791	98.57	5.9760	6.0126	100.61
Average:			99.61						
Standard Deviation:			1.07						
Relative standard deviation (RSD%):			1.07						
Confidence interval (Probability 95%):			99.12 – 100.10						

Table S10. Accuracy levels – real (theoretical) and determined concentrations of compound **1m** and mean percent recovery (%) of six injections of each accuracy concentration (80%, 100%, and 120% analytical concentrations)

Sample No.	Compound 1m concentration								
	4 µg/ mL solution			5 µg/ mL solution			6 µg/ mL solution		
	Real [µg/ mL]	Determined [µg/ mL]	Recovery [%]	Real [µg/ mL]	Determined [µg/ mL]	Recovery, [%]	Real [µg/ mL]	Determined [µg/ mL]	Recovery, [%]
1.	4.0000	4.0299	100.75	5.0100	5.0287	100.37	6.1320	6.0211	98.19
2.	4.0000	4.0621	101.55	5.0100	4.9774	99.35	6.1320	6.1496	100.29
3.	4.0000	4.0122	100.30	5.0100	4.9878	99.56	6.1320	6.0550	98.74
4.	4.0000	4.0183	100.46	5.0100	4.9509	98.82	6.1320	6.1599	100.46
5.	4.0000	4.0091	100.23	5.0100	5.0205	100.21	6.1320	6.1337	100.03
6.	4.0000	3.9423	98.56	5.0100	4.9807	99.41	6.1320	6.0462	98.60
Average:			99.77						
Standard Deviation:			0.91						
Relative standard deviation (RSD%):			0.91						
Confidence interval (Probability 95%):			99.35 – 100.19						

Table S11. Accuracy levels – real (theoretical) and determined concentrations of compound **1g** and mean percent recovery (%) of six injections of each accuracy concentration (80%, 100%, and 120% analytical concentrations)

Sample No.	Compound 1g concentration								
	**4 µg/ mL solution**			**5 µg/ mL solution**			**6 µg/ mL solution**		
	**Real [µg/ mL]**	**Determined [µg/ mL]**	**Recovery [%]**	**Real [µg/ mL]**	**Determined [µg/ mL]**	**Recovery, [%]**	**Real [µg/ mL]**	**Determined [µg/ mL]**	**Recovery, [%]**
1.	4.0240	4.0729	101.21	4.9800	5.0496	101.40	6.0240	6.0501	100.43
2.	4.0240	4.1037	101.98	4.9800	5.0847	102.10	6.0240	6.1279	101.72
3.	4.0240	3.9574	98.34	4.9800	5.0536	101.48	6.0240	5.9366	98.55
4.	4.0240	3.9844	99.02	4.9800	4.9916	100.23	6.0240	6.0975	101.22
5.	4.0240	4.0029	99.48	4.9800	5.0731	101.87	6.0240	6.1198	101.59
6.	4.0240	3.9538	98.26	4.9800	4.9465	99.33	6.0240	6.0647	100.68
Average:			100.49						
Standard Deviation:			1.34						
Relative standard deviation (RSD%):			1.33						
Confidence interval (Probability 95%):			99.88 – 101.11						

Table S12. Accuracy levels – real (theoretical) and determined concentrations of compound **1n** and mean percent recovery (%) of six injections of each accuracy concentration (80%, 100%, and 120% analytical concentrations)

Sample No.	Compound 1n concentration								
	**4 µg/ mL solution**			**5 µg/ mL solution**			**6 µg/ mL solution**		
	**Real [µg/ mL]**	**Determined [µg/ mL]**	**Recovery [%]**	**Real [µg/ mL]**	**Determined [µg/ mL]**	**Recovery, [%]**	**Real [µg/ mL]**	**Determined [µg/ mL]**	**Recovery, [%]**
1.	4.1120	4.1585	101.13	4.9400	4.9708	100.62	6.0960	6.0443	99.15
2.	4.1120	4.1828	101.72	4.9400	4.9953	101.12	6.0960	6.1840	101.44
3.	4.1120	4.1322	100.49	4.9400	4.8838	98.86	6.0960	6.2049	101.79
4.	4.1120	4.1020	99.76	4.9400	4.9562	100.33	6.0960	6.1416	100.75
5.	4.1120	4.1044	99.82	4.9400	5.0147	101.51	6.0960	6.0619	99.44
6.	4.1120	4.0721	99.03	4.9400	4.9304	99.81	6.0960	6.0620	99.44
Average:			100.34						
Standard Deviation:			0.97						
Relative standard deviation (RSD%):			0.96						
Confidence interval (Probability 95%):			99.90 – 100.79						

Compound **1n** content – Accuracy Results

ROBUSTNESS

The robustness of an analytical procedure is a measure of its capacity to remain unaffected by small, but deliberate variations in method parameters and provides an indication of its reliability during normal usage.

It should show the reliability of an analysis with respect to deliberate variations in method parameters.

The following variations were investigated, while keeping all other chromatographic parameters unchanged (normal conditions, according to section 2 of this document, "*Description of the method*"):

- column temperature = 35°C, with constant normal chromatographic conditions;
- mobile phase composition:
  - Solvent A: Solvent B (91: 9, V/V);
  - Solvent A: Solvent B (95: 5, V/V);
- flow rate = 0.8 mL/min, other chromatographic conditions being maintained normal;
- flow rate = 1.2 mL/min, other chromatographic conditions being maintained normal;

Preparation of solutions:

Stock solutions of 500 µg/ mL are prepared from each of the tested compounds:

Stock solution ***1h*** *(500 µg/ mL):* 5 mg compound **1h** were transferred into a 10 mL volumetric flask, 5 mL acetonitrile R were added, the flask was sonicated for 5 minutes, cooled to room temperature and diluted to volume with acetonitrile R.

Stock solution ***1j*** *(500 µg/ mL):* 5 mg compound **1j** were transferred into a 10 mL volumetric flask, 5 mL acetonitrile R were added, the flask was sonicated for 5 minutes, cooled to room temperature and diluted to volume with acetonitrile R.

Stock solution ***1i*** *(500 µg/ mL):* 5 mg compound **1i** were transferred into a 10 mL volumetric flask, 5 mL acetonitrile R were added, the flask was sonicated for 5 minutes, cooled to room temperature and diluted to volume with acetonitrile R.

Stock solution ***1m*** *(500 µg/ mL):* 5 mg compound **1m** were transferred into a 10 mL volumetric flask, 5 mL acetonitrile R were added, the flask was sonicated for 5 minutes, cooled to room temperature and diluted to volume with acetonitrile R.

Stock solution ***1g*** *(500 µg/ mL):* 5 mg compound **1g** were transferred into a 10 mL volumetric flask, 5 mL acetonitrile R were added, the flask was sonicated for 5 minutes, cooled to room temperature and

diluted to volume with acetonitrile R.

*Stock solution **1n** (500 µg/ mL):* 5 mg compound **1n** were transferred into a 10 mL volumetric flask, 5 mL acetonitrile R were added, the flask was sonicated for 5 minutes, cooled to room temperature and diluted to volume with acetonitrile R.

*Standard solution (5 µg/ mL compound **1h**, 5 µg/ mL compound **1j**, 5 µg/ mL compound **1i**, 5 µg/ mL compound **1m**, 5 µg/ mL compound **1g**, 5 µg/ mL compound **1n**)*: 0.25 mL of each stock solution were diluted to 25 mL with methanol R.

Procedure:

The following sequence was used: SS, $SS_{flow\ 0.8\ mL/min}$, $SS_{flow\ 1.2\ mL/min}$, $SS_{m.phase\ 91/9}$, $SS_{m.phase\ 95/5}$, $SS_{temp.\ 35°C}$, where,

SS – Standard Solution, chromatographic conditions maintained normal;

$SS_{flow\ 0.8\ mL/min}$ – Standard Solution, flow rate = 0.8 mL/min, other chromatographic conditions being maintained normal;

$SS_{flow\ 1.2\ mL/min}$ – Standard Solution, flow rate = 1.2 mL/min, other chromatographic conditions being maintained normal;

$SS_{m.phase\ 91/9}$ – Standard Solution, registered with altered percentage of mobile phase, Solvent A: Solvent B (91: 9, V/V);

$SS_{m.phase\ 95/5}$ – Standard Solution, registered with altered percentage of mobile phase, Solvent A: Solvent B (95: 5, V/V);

$SS_{temp.\ 35°C}$ – Standard Solution, column temperature = 35°C.

Results:

The effect of small variations of chromatographic parameters on the performance of the method in presented in the **Table S13**.

Table S13. Robustness results for the proposed RP-HPLC analytical method

**Robustness – Deliberate Variations in Method Parameters – 5 µg/ mL Solution 1h, 1j, 1i, 1m, 1g, 1n**													
			Mobile phase variation				Flow rate variation				Column temperature variation	
Main peaks names	Validated chromatographic parameters [(1)]		Solvent A: Solvent B (91: 9, V/V)		Solvent A: Solvent B (95: 5, V/V)		Flow Rate 0.8 mL/ min		Flow Rate 1.2 mL/ min		Column Temperature 35°C	
	Ret. Time [min.]	Resolution [(2)]	Ret. Time [min.]	Resolution [(2)]	Ret. Time [min.]	Resolution [(2)]	Ret. Time [min.]	Resolution [(2)]	Ret. Time [min.]	Resolution [(2)]	Ret. Time [min.]	Resolution [(2)]
**1h**	11.134		12.367		10.095		13.941		9.307		10.289	
**1j**	12.430	3.23	13.947	3.49	11.169	2.96	15.571	3.46	10.393	3.06	11.384	3.10
**1i**	13.600	2.67	15.254	2.60	12.236	2.69	17.044	2.85	11.374	2.53	12.392	2.63
**1m**	14.969	2.82	16.984	3.18	13.317	2.50	18.751	2.99	12.510	2.64	13.733	3.20
**1g**	22.319	11.88	25.732	12.37	19.563	11.42	27.995	12.66	18.659	11.21	19.759	11.42
**1n**	25.466	4.05	29.367	4.16	22.208	3.87	31.927	4.28	21.292	3.83	22.509	4.19

(1) The method has been validated by setting the following chromatographic conditions: mobile phase = Solvent A: Solvent B (93: 7, V/V), flow rate = 1.0 mL/min, column temperature = room temperature;

(2) Resolution calculated between couples of two successive peaks.

CONCLUSIONS

After evaluating the specificity of the method, no interference was observed between the analytical signal of solvent, and the peaks of interest (compounds **1h**, **1j**, **1i**, **1m**, **1g**, **1n**). Purity angles of all peaks recorded in the solutions chromatograms are below their corresponding thresholds, indicating proper separation of the compounds.

Therefore, the analytical procedure used to separate and quantitatively evaluate the compounds **1h**, **1j**, **1i**, **1m**, **1g**, and **1n** is **specific**.

The **precision** of the analytical method has been evaluated by repeatability and RSD values do not exceed the acceptance threshold (≤ 2.0%).

The detection limit and quantitation limit results determined on *test solutions* range between 0.0184 – 0.0252 μg/ mL, and 1.228 – 1.594 μg/ mL. The R^2 correlation coefficient of the **LOQ** and **linearity** curves are greater than 0.99, indicating that the method does not face procedural errors in the quantitative analysis, over the specified concentrations interval.

The **accuracy** study of the HPLC method showed that the determined concentrations of compounds **1h**, **1j**, **1i**, **1m**, **1g**, and **1n** are within proposed acceptance criterion, 98% - 102%. The given range is related to the real content of test solutions.

The effect of small variations of chromatographic parameters on the performance of the method has been evaluated. Obtained results indicate that the method is **robust**, allowing for proper separation of **1h**, **1j**, **1i**, **1m**, **1g**, and **1n** peaks, as demonstrated by a resolution value greater than or equal to 2.0 for the investigated variations of the chromatographic method.

Considering the presented results, the method for quatitative determination of compounds **1h**, **1j**, **1i**, **1m**, **1g**, and **1n** fulfills the acceptance criteria for the parameters mentioned in the validation report and can bwe used for routine quality control analyses.

Quantitative determination of

compounds 1g, 1h, 1i, 1j, 1m, 1n

- Validation of the analytical method -

Validation parameters:

- Specificity
- Precision 1
- Precision 2
- LOD – LOQ
- Linearity
- Range
- Accuracy
- Robustness

Quantitative determination of

compounds 1g, 1h, 1i, 1j, 1m, 1n

- Validation of the analytical method -

Validation parameters:

- **Specificity**
- Precision 1
- Precision 2
- LOD – LOQ
- Linearity
- Range
- Accuracy
- Robustness

Chloride_Ethyl_Specificity

Reported by User: Roman Roxana (roman_roxana)
Acquisition Server Waters-cd3
Project Name: Teste\Thiazides
Sample Set Name: Seria Etil_Cl Specificity
Code column: Inertsil ODS-3 250*4.6 mm 5um

SAMPLE INFORMATION

Sample Name:	Solvent - Methanol		
Sample Type:	Standard	Date Acquired:	2/13/2023 6:01:17 PM EET
Vial:	7	Acq. Method Set:	Seria Etil_Cl 7 93 Specificity
Injection #:	1	Date Processed:	2/27/2023 7:02:57 AM EET
Injection Volume:	10.00 ul	Processing Method:	Seria Etil_Cl 7 93 Specificity
Run Time:	30.0 Minutes	Channel Name:	Extract 275
Acquired By:	roman_roxana	Proc. Chnl. Descr.:	2998 PDA 275.0 nm (2998

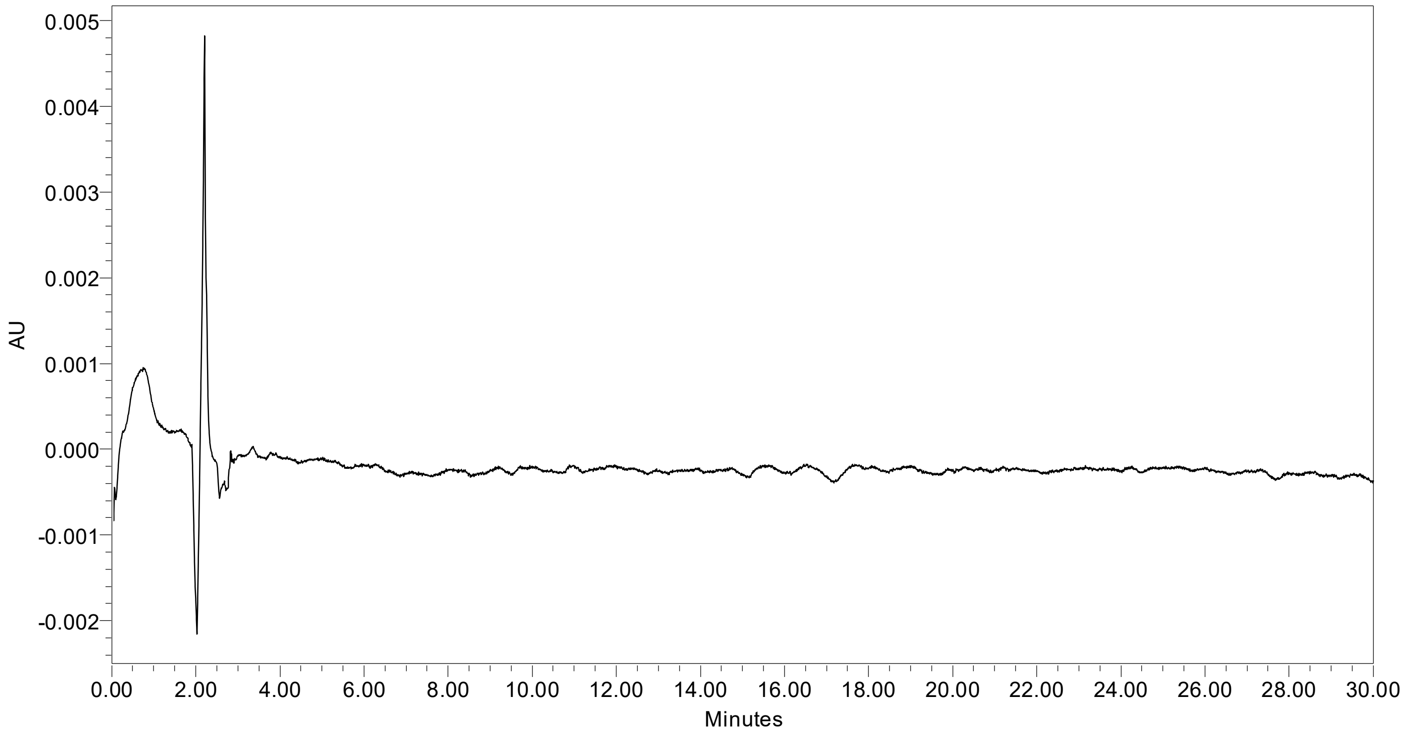

Error Log
Basic LC Peaks Table group contains information that doesn't match the data being reported.

Chloride_Ethyl_Specificity

Reported by User: Roman Roxana (roman_roxana)
Acquisition Server Waters-cd3
Project Name: Teste\Thiazides
Sample Set Name: Seria Etil_Cl Specificity
Code column: Inertsil ODS-3 250*4.6 mm 5um

SAMPLE INFORMATION

Sample Name:	Solution 1h 5 ug/ mL		
Sample Type:	Standard	Date Acquired:	2/25/2023 11:31:26 AM EET
Vial:	2	Acq. Method Set:	Seria Etil_Cl 7 93 Specificity
Injection #:	1	Date Processed:	2/27/2023 6:56:15 AM EET
Injection Volume:	10.00 ul	Processing Method:	Seria Etil_Cl 7 93 Specificity
Run Time:	30.0 Minutes	Channel Name:	Extract 275
Acquired By:	roman_roxana	Proc. Chnl. Descr.:	2998 PDA 275.0 nm (2998

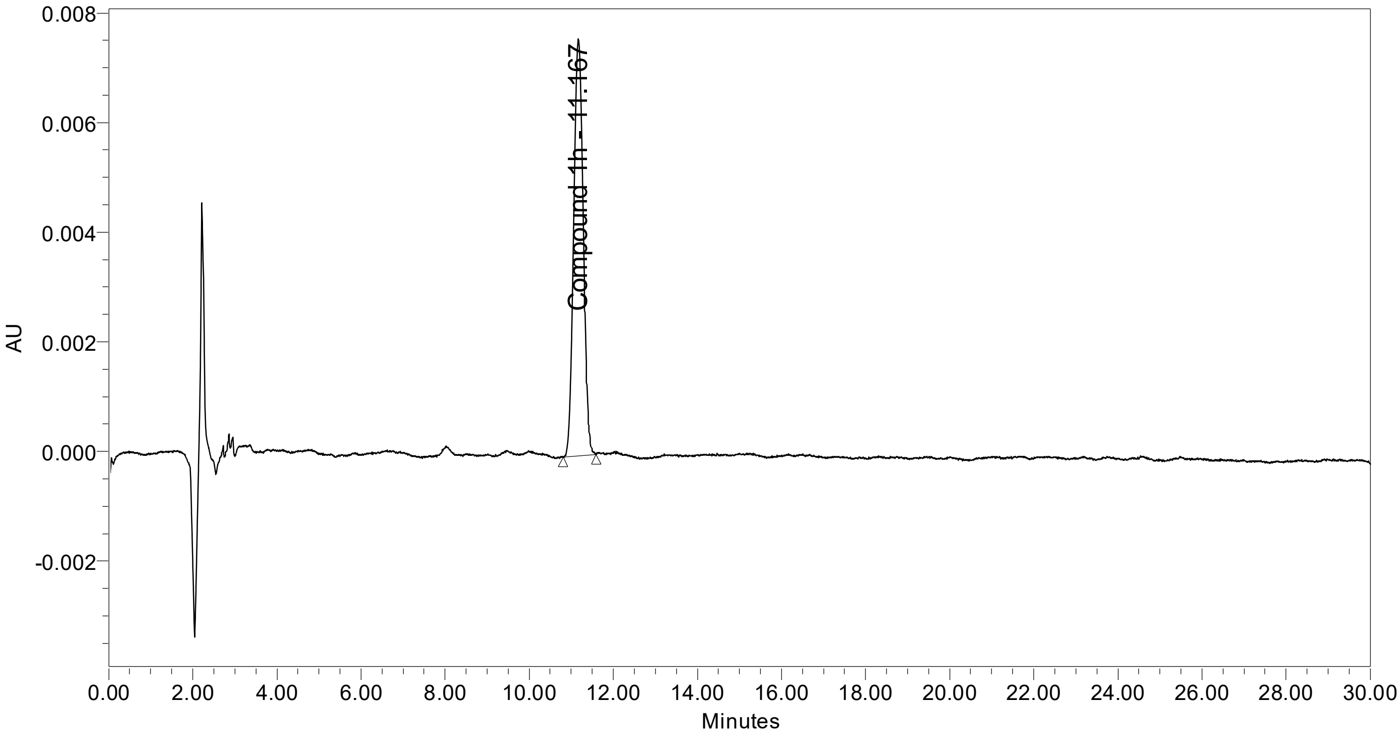

	Peak Name	RT	Area	Height (µV)	Purity1 Angle	Purity1 Threshold	Symmetry Factor	EP Plate Count
1	Compound 1h	11.167	119025	7615	0.299	0.650	1.06	1.135571e+004

Chloride_Ethyl_Specificity

Reported by User: Roman Roxana (roman_roxana)
Acquisition Server Waters-cd3
Project Name: Teste\Thiazides
Sample Set Name: Seria Etil_Cl Specificity
Code column: Inertsil ODS-3 250*4.6 mm 5um

SAMPLE INFORMATION

Sample Name:	Solution 1j 5.0 ug/ mL		
Sample Type:	Standard	Date Acquired:	2/25/2023 12:54:58 PM EET
Vial:	4	Acq. Method Set:	Seria Etil_Cl 7 93 Specificity
Injection #:	1	Date Processed:	2/27/2023 6:55:47 AM EET
Injection Volume:	10.00 ul	Processing Method:	Seria Etil_Cl 7 93 Specificity
Run Time:	30.0 Minutes	Channel Name:	Extract 275
Acquired By:	roman_roxana	Proc. Chnl. Descr.:	2998 PDA 275.0 nm (2998

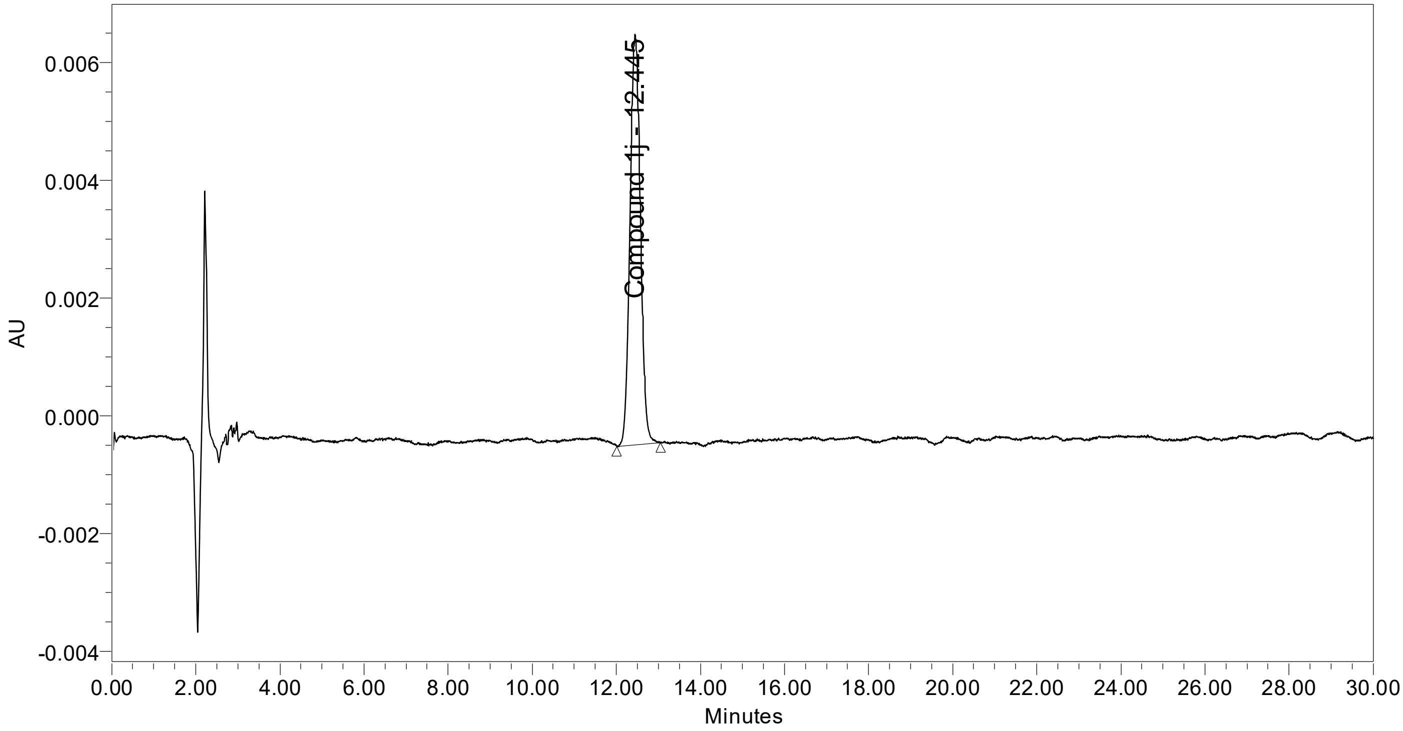

	Peak Name	RT	Area	Height (µV)	Purity1 Angle	Purity1 Threshold	Symmetry Factor	EP Plate Count
1	Compound 1j	12.445	122578	6978	0.387	0.642	1.07	1.134319e+004

Chloride_Ethyl_Specificity

Reported by User: Roman Roxana (roman_roxana)
Acquisition Server Waters-cd3
Project Name: Teste\Thiazides
Sample Set Name: Seria Etil_Cl Specificity
Code column: Inertsil ODS-3 250*4.6 mm 5um

SAMPLE INFORMATION

Sample Name:	Solution 1i 5.0 ug/ mL		
Sample Type:	Standard	Date Acquired:	2/25/2023 12:17:03 PM EET
Vial:	3	Acq. Method Set:	Seria Etil_Cl 7 93 Specificity
Injection #:	1	Date Processed:	2/27/2023 6:56:09 AM EET
Injection Volume:	10.00 ul	Processing Method:	Seria Etil_Cl 7 93 Specificity
Run Time:	30.0 Minutes	Channel Name:	Extract 275
Acquired By:	roman_roxana	Proc. Chnl. Descr.:	2998 PDA 275.0 nm (2998

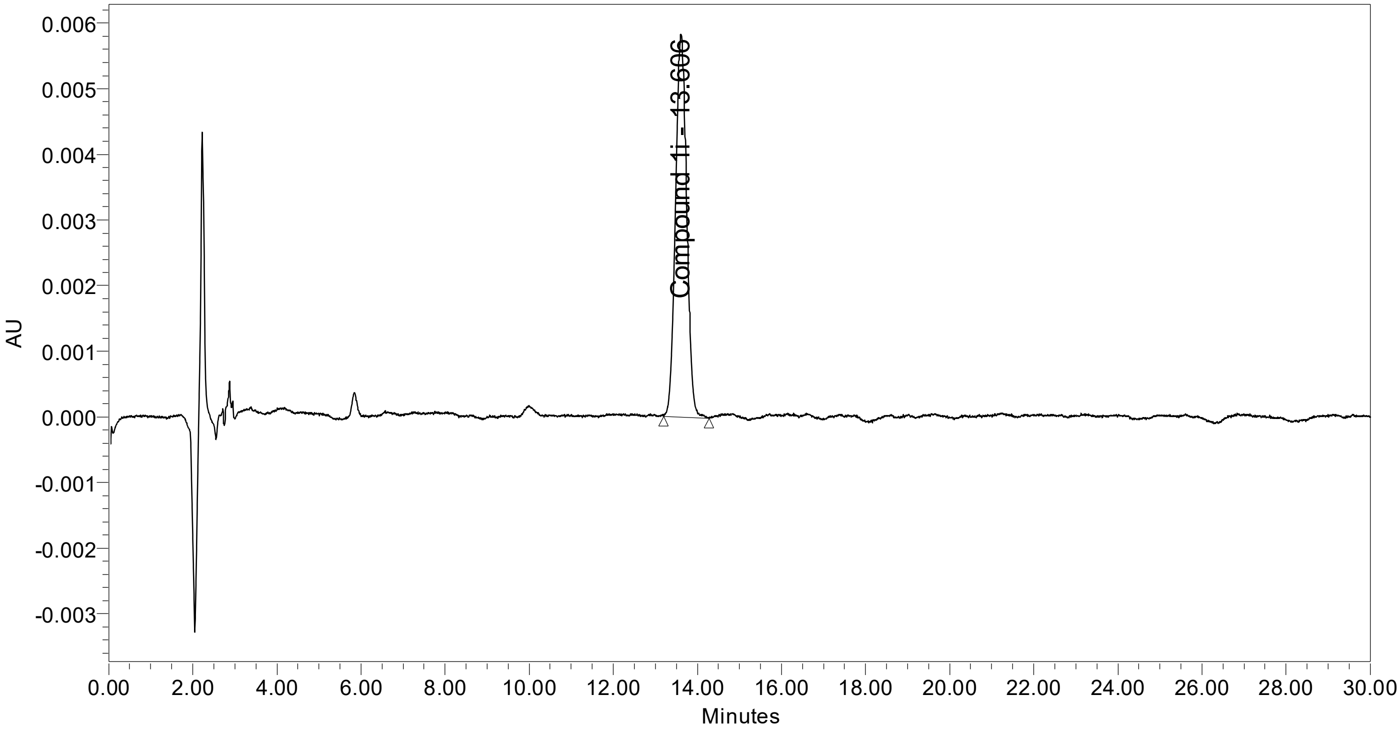

	Peak Name	RT	Area	Height (µV)	Purity1 Angle	Purity1 Threshold	Symmetry Factor	EP Plate Count
1	Compound 1i	13.606	110199	5835	0.337	0.636	1.07	1.165344e+004

Chloride_Ethyl_Specificity

Reported by User: Roman Roxana (roman_roxana)
Acquisition Server Waters-cd3
Project Name: Teste\Thiazides
Sample Set Name: Seria Etil_Cl Specificity
Code column: Inertsil ODS-3 250*4.6 mm 5um

SAMPLE INFORMATION

Sample Name:	Solution 1m 5.0 ug/ mL		
Sample Type:	Standard	Date Acquired:	2/25/2023 1:40:57 PM EET
Vial:	5	Acq. Method Set:	Seria Etil_Cl 7 93 Specificity
Injection #:	1	Date Processed:	2/27/2023 6:58:44 AM EET
Injection Volume:	10.00 ul	Processing Method:	Seria Etil_Cl 7 93 Specificity
Run Time:	30.0 Minutes	Channel Name:	Extract 275
Acquired By:	roman_roxana	Proc. Chnl. Descr.:	2998 PDA 275.0 nm (2998

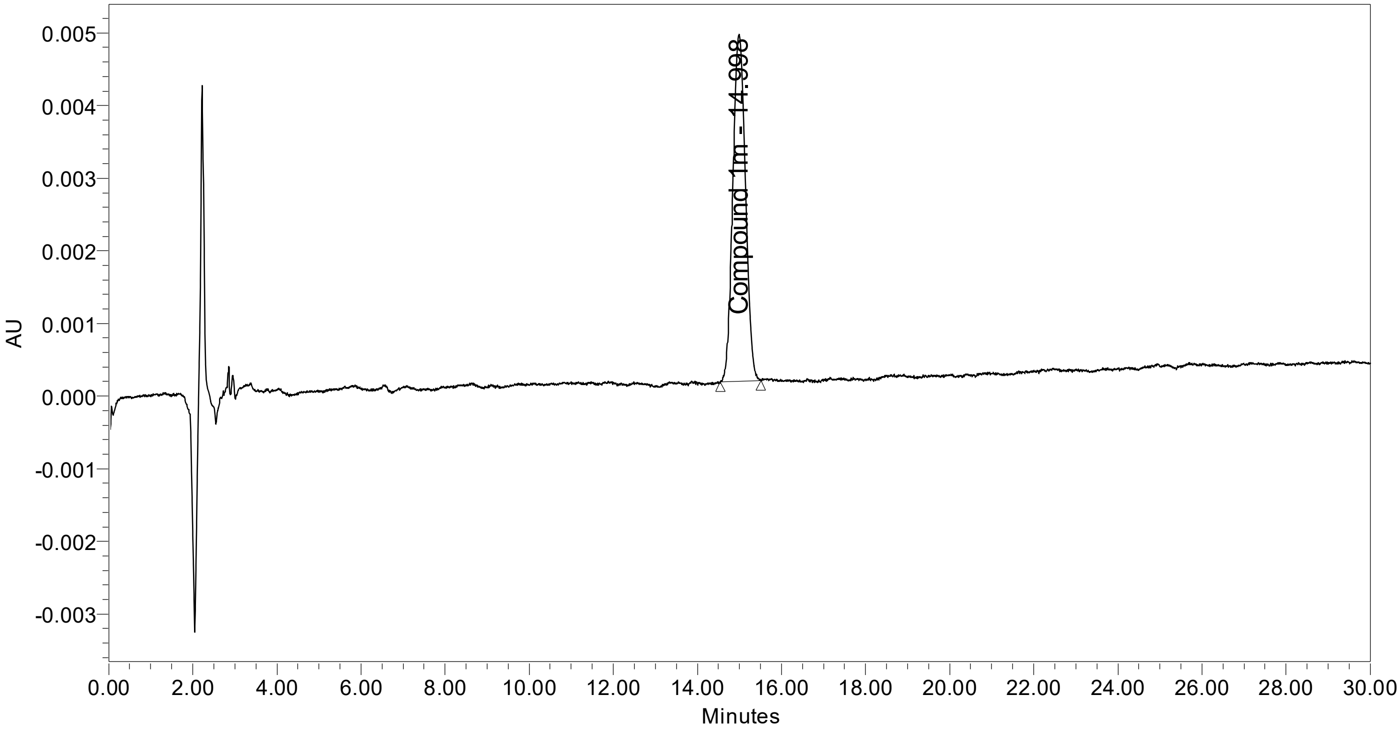

	Peak Name	RT	Area	Height (µV)	Purity1 Angle	Purity1 Threshold	Symmetry Factor	EP Plate Count
1	Compound 1m	14.998	99488	4779	0.380	0.604	1.02	1.145172e+004

Chloride_Ethyl_Specificity

Reported by User: Roman Roxana (roman_roxana)
Acquisition Server Waters-cd3
Project Name: Teste\Thiazides
Sample Set Name: Seria Etil_Cl Specificity
Code column: Inertsil ODS-3 250*4.6 mm 5um

SAMPLE INFORMATION

Sample Name:	Solution 1g 5.0 ug/ mL		
Sample Type:	Standard	Date Acquired:	2/25/2023 10:50:51 AM EET
Vial:	1	Acq. Method Set:	Seria Etil_Cl 7 93 Specificity
Injection #:	1	Date Processed:	2/27/2023 6:55:34 AM EET
Injection Volume:	10.00 ul	Processing Method:	Seria Etil_Cl 7 93 Specificity
Run Time:	30.0 Minutes	Channel Name:	Extract 275
Acquired By:	roman_roxana	Proc. Chnl. Descr.:	2998 PDA 275.0 nm (2998

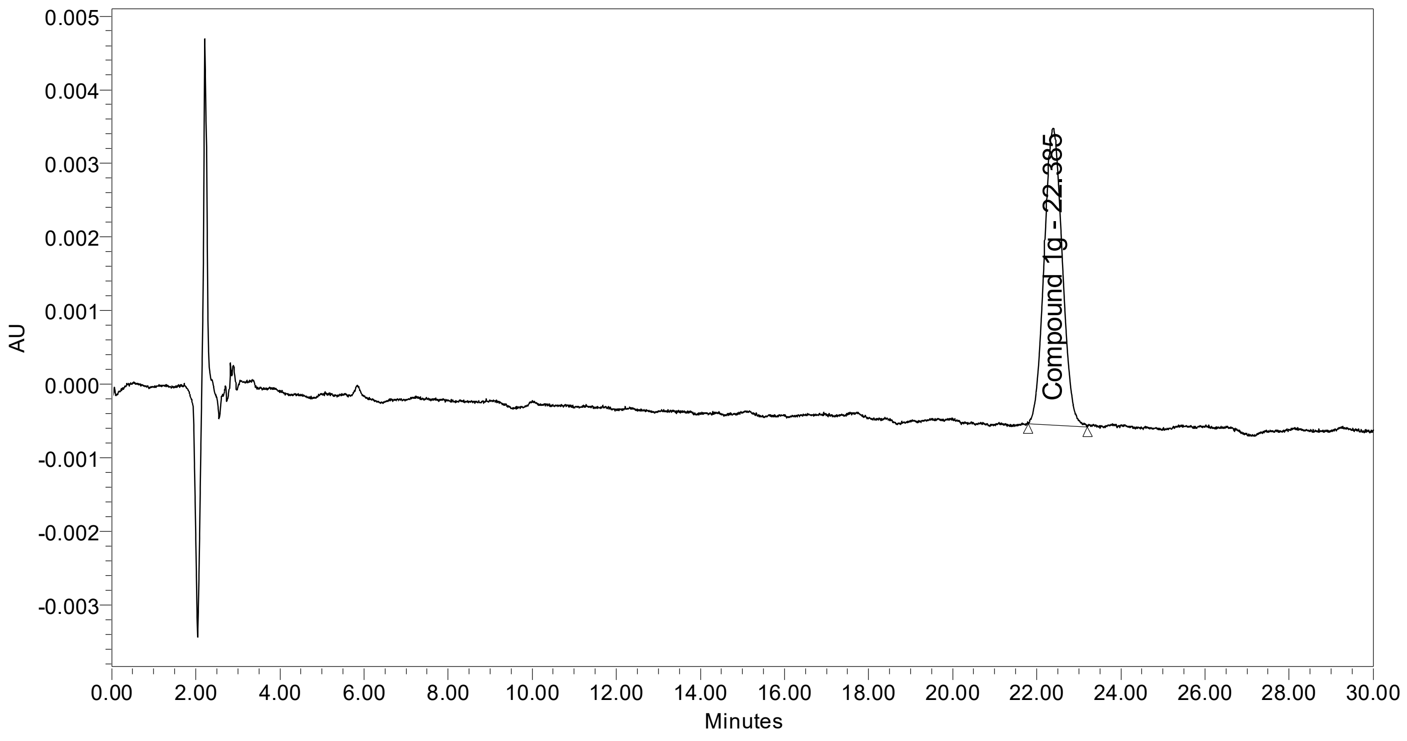

	Peak Name	RT	Area	Height (µV)	Purity1 Angle	Purity1 Threshold	Symmetry Factor	EP Plate Count
1	Compound 1g	22.385	122960	4032	0.785	1.369	1.07	1.209107e+004

Chloride_Ethyl_Specificity

Reported by User: Roman Roxana (roman_roxana)
Acquisition Server Waters-cd3
Project Name: Teste\Thiazides
Sample Set Name: Seria Etil_Cl Specificity
Code column: Inertsil ODS-3 250*4.6 mm 5um

SAMPLE INFORMATION

Sample Name:	Solution 1n 5.0 ug/ mL		
Sample Type:	Unknown	Date Acquired:	2/25/2023 2:26:35 PM EET
Vial:	6	Acq. Method Set:	Seria Etil_Cl 7 93 Specificity
Injection #:	1	Date Processed:	2/27/2023 6:58:56 AM EET
Injection Volume:	10.00 ul	Processing Method:	Seria Etil_Cl 7 93 Specificity
Run Time:	30.0 Minutes	Channel Name:	Extract 275
Acquired By:	roman_roxana	Proc. Chnl. Descr.:	2998 PDA 275.0 nm (2998

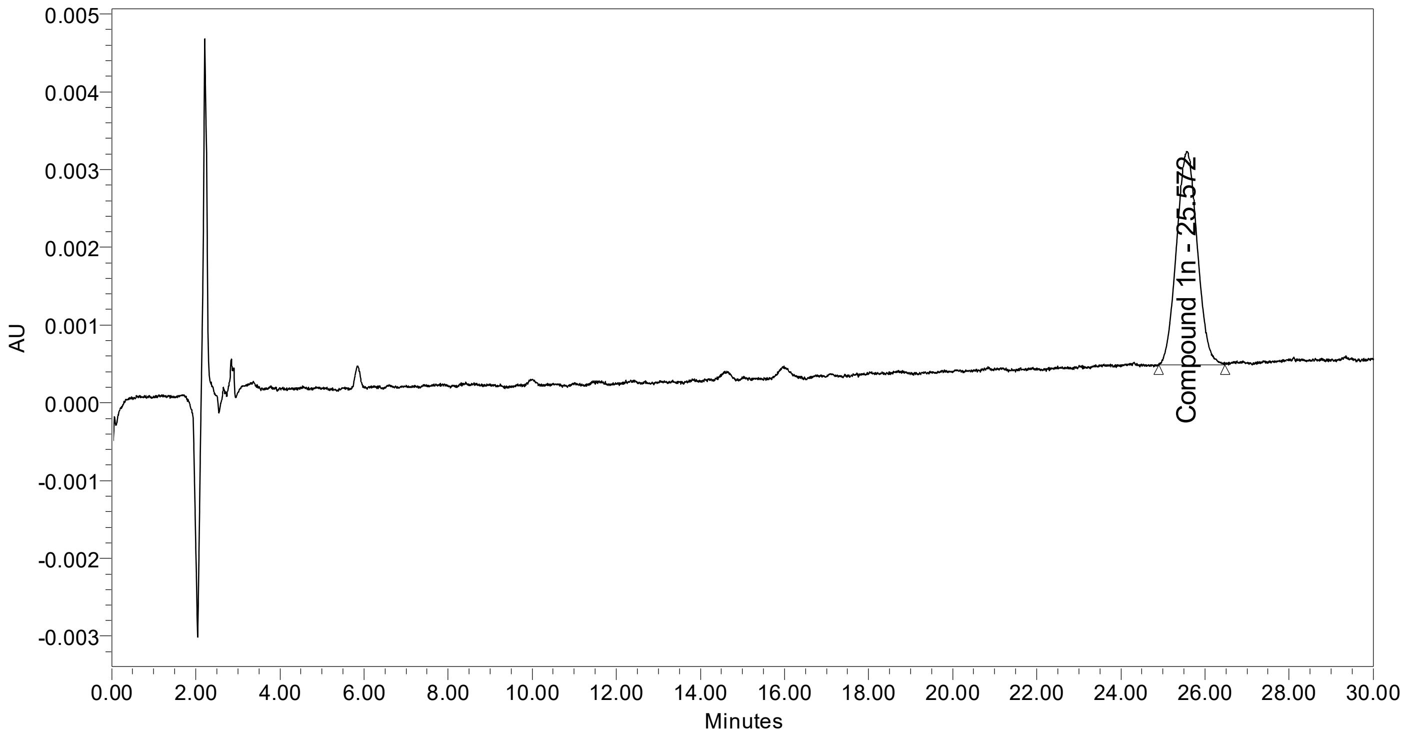

	Peak Name	RT	Area	Height (µV)	Purity1 Angle	Purity1 Threshold	Symmetry Factor	EP Plate Count
1	Compound 1n	25.572	95177	2744	0.693	1.463	1.05	1.208410e+004

Quantitative determination of

compounds 1g, 1h, 1i, 1j, 1m, 1n

- Validation of the analytical method -

Validation parameters:

- Specificity
- **Precision 1**
- Precision 2
- LOD – LOQ
- Linearity
- Range
- Accuracy
- Robustness

Chloride_Ethyl_Precision 1

Reported by User: Roman Roxana (roman_roxana)
Acquisition Server Waters-cd3
Project Name: Teste\Thiazides
Sample Set Name: Seria Etil_Cl 7 93 Accuracy
Code column: Inertsil ODS-3 250*4.6 mm 5um LA140

SAMPLE INFORMATION

Sample Name:	Solution 5.0 ug/ mL		
Sample Type:	Unknown	Date Acquired:	2/11/2023 10:54:16 PM EET
Vial:	8	Acq. Method Set:	Seria Etil_Cl 7 93 Accuracy
Injection #:	1	Date Processed:	4/19/2023 2:19:19 PM EEST
Injection Volume:	10.00 ul	Processing Method:	Seria Etil_Cl 7 93 Accuracy
Run Time:	35.0 Minutes	Channel Name:	Extract 275
Acquired By:	roman_roxana	Proc. Chnl. Descr.:	2998 PDA 275.0 nm (2998

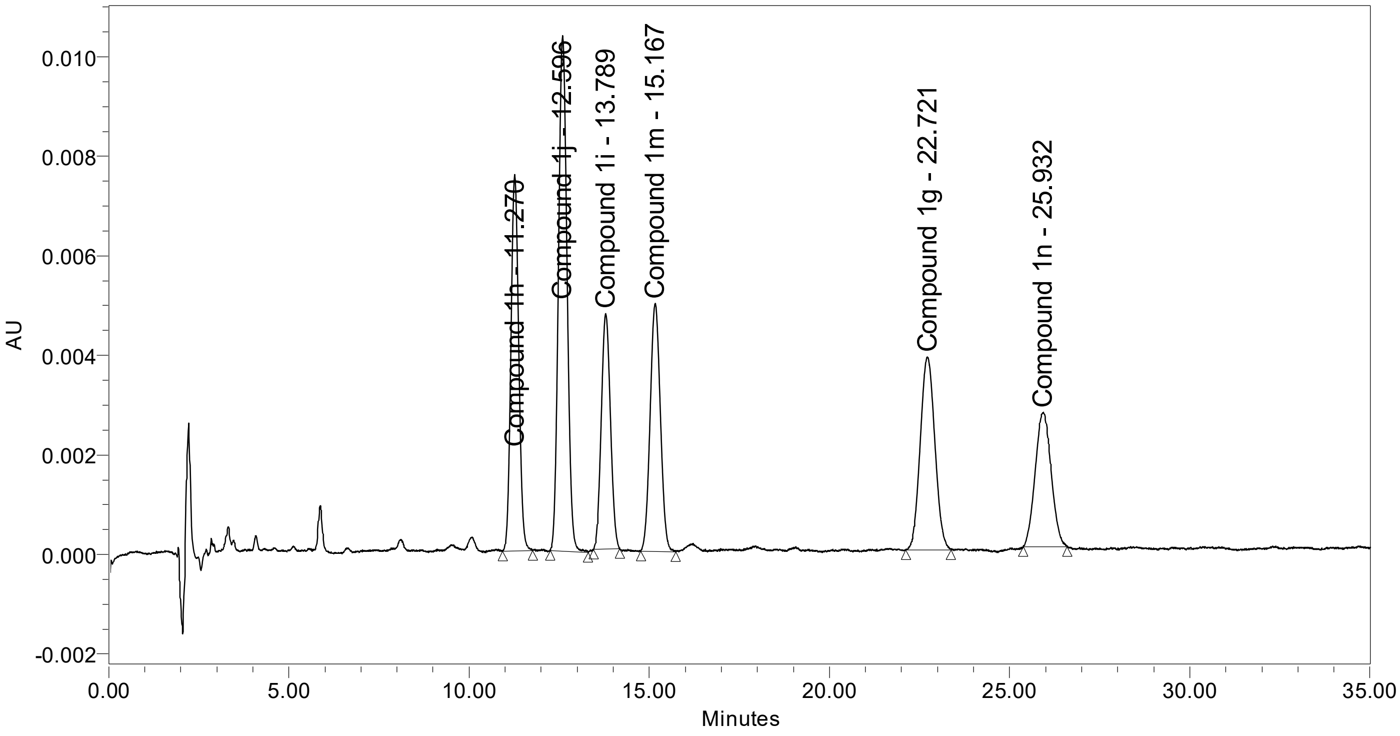

	Peak Name	RT	Area	Height (µV)	Resolution
1	Compound 1h	11.270	111591	7560	
2	Compound 1j	12.596	169135	10358	3.24
3	Compound 1i	13.789	82009	4729	2.67
4	Compound 1m	15.167	97170	4975	2.81
5	Compound 1g	22.721	108491	3876	11.98

	Peak Name	RT	Area	Height (µV)	Resolution
6	Compound 1n	25.932	84215	2703	4.06

Chloride_Ethyl_Precision 1

Reported by User: Roman Roxana (roman_roxana)
Acquisition Server Waters-cd3
Project Name: Teste\Thiazides
Sample Set Name: Seria Etil_Cl 7 93 Accuracy
Code column: Inertsil ODS-3 250*4.6 mm 5um LA140

SAMPLE INFORMATION

Sample Name:	Solution 5.0 ug/ mL		
Sample Type:	Unknown	Date Acquired:	2/11/2023 11:29:57 PM EET
Vial:	9	Acq. Method Set:	Seria Etil_Cl 7 93 Accuracy
Injection #:	1	Date Processed:	4/19/2023 2:20:41 PM EEST
Injection Volume:	10.00 ul	Processing Method:	Seria Etil_Cl 7 93 Accuracy
Run Time:	35.0 Minutes	Channel Name:	Extract 275
Acquired By:	roman_roxana	Proc. Chnl. Descr.:	2998 PDA 275.0 nm (2998

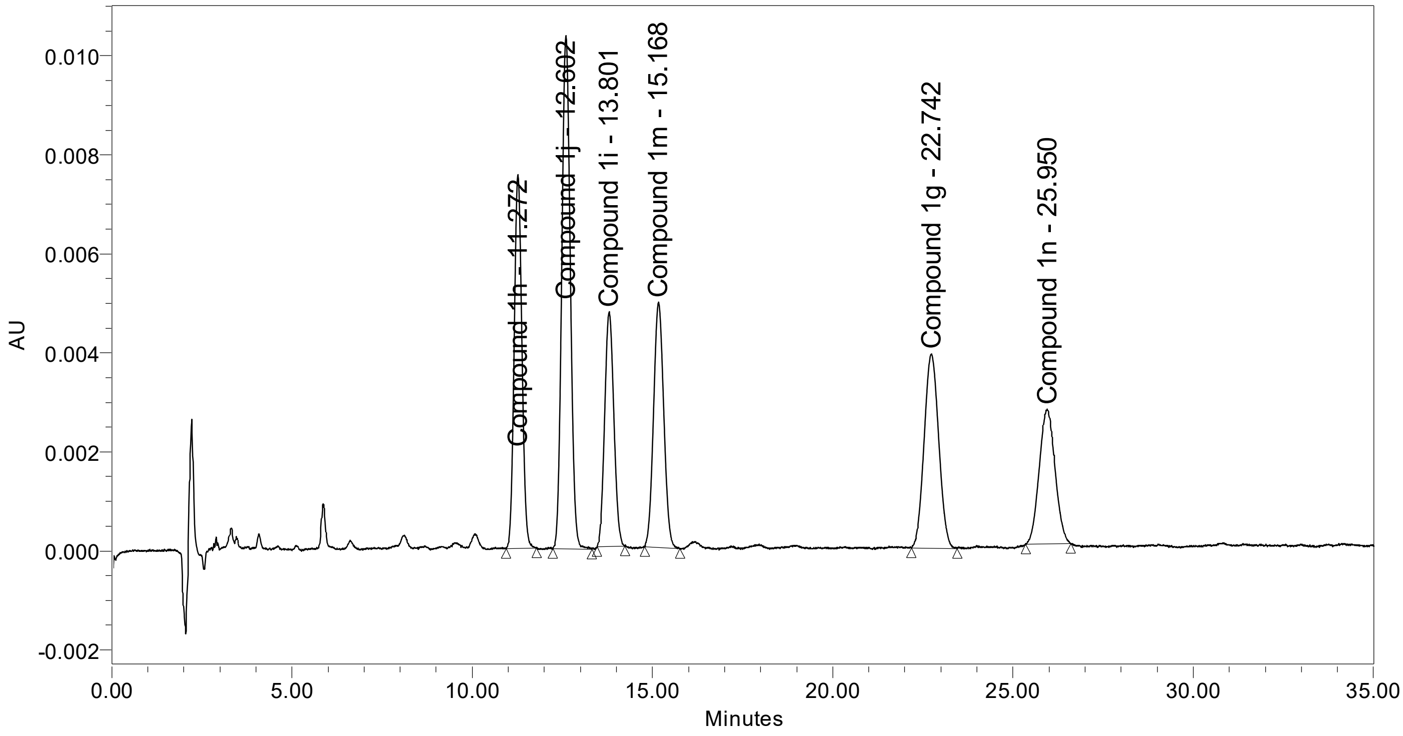

	Peak Name	RT	Area	Height (µV)	Resolution
1	Compound 1h	11.272	111110	7556	
2	Compound 1j	12.602	168465	10362	3.26
3	Compound 1i	13.801	82735	4741	2.68
4	Compound 1m	15.168	96179	4953	2.79
5	Compound 1g	22.742	109245	3926	12.05

	Peak Name	RT	Area	Height (µV)	Resolution
6	Compound 1n	25.950	84630	2728	4.06

Chloride_Ethyl_Precision 1

Reported by User: Roman Roxana (roman_roxana)
Acquisition Server Waters-cd3
Project Name: Teste\Thiazides
Sample Set Name: Seria Etil_Cl 7 93 Accuracy
Code column: Inertsil ODS-3 250*4.6 mm 5um LA140

SAMPLE INFORMATION

Sample Name:	Solution 5.0 ug/ mL		
Sample Type:	Unknown	Date Acquired:	2/12/2023 12:05:37 AM EET
Vial:	10	Acq. Method Set:	Seria Etil_Cl 7 93 Accuracy
Injection #:	1	Date Processed:	4/19/2023 2:21:41 PM EEST
Injection Volume:	10.00 ul	Processing Method:	Seria Etil_Cl 7 93 Accuracy
Run Time:	35.0 Minutes	Channel Name:	Extract 275
Acquired By:	roman_roxana	Proc. Chnl. Descr.:	2998 PDA 275.0 nm (2998

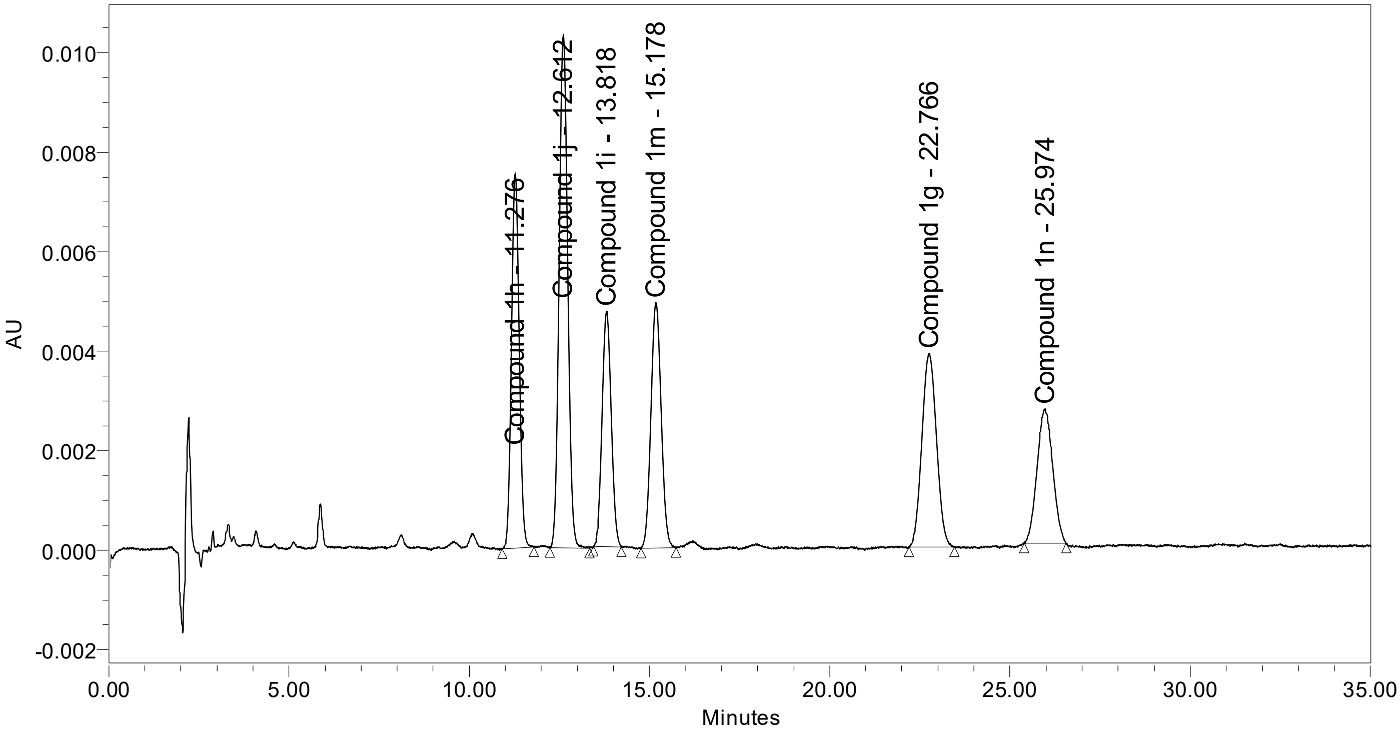

	Peak Name	RT	Area	Height (µV)	Resolution
1	Compound 1h	11.276	111024	7548	
2	Compound 1j	12.612	168231	10320	3.27
3	Compound 1i	13.818	82661	4734	2.69
4	Compound 1m	15.178	96381	4942	2.77
5	Compound 1g	22.766	108577	3894	12.07
6	Compound 1n	25.974	82741	2697	4.08

Chloride_Ethyl_Precision 1

Reported by User: Roman Roxana (roman_roxana)
Acquisition Server Waters-cd3
Project Name: Teste\Thiazides
Sample Set Name: Seria Etil_Cl 7 93 Accuracy
Code column: Inertsil ODS-3 250*4.6 mm 5um LA140

SAMPLE INFORMATION

Sample Name:	Solution 5.0 ug/ mL		
Sample Type:	Unknown	Date Acquired:	2/12/2023 12:41:19 AM EET
Vial:	11	Acq. Method Set:	Seria Etil_Cl 7 93 Accuracy
Injection #:	1	Date Processed:	4/19/2023 2:22:11 PM EEST
Injection Volume:	10.00 ul	Processing Method:	Seria Etil_Cl 7 93 Accuracy
Run Time:	35.0 Minutes	Channel Name:	Extract 275
Acquired By:	roman_roxana	Proc. Chnl. Descr.:	2998 PDA 275.0 nm (2998

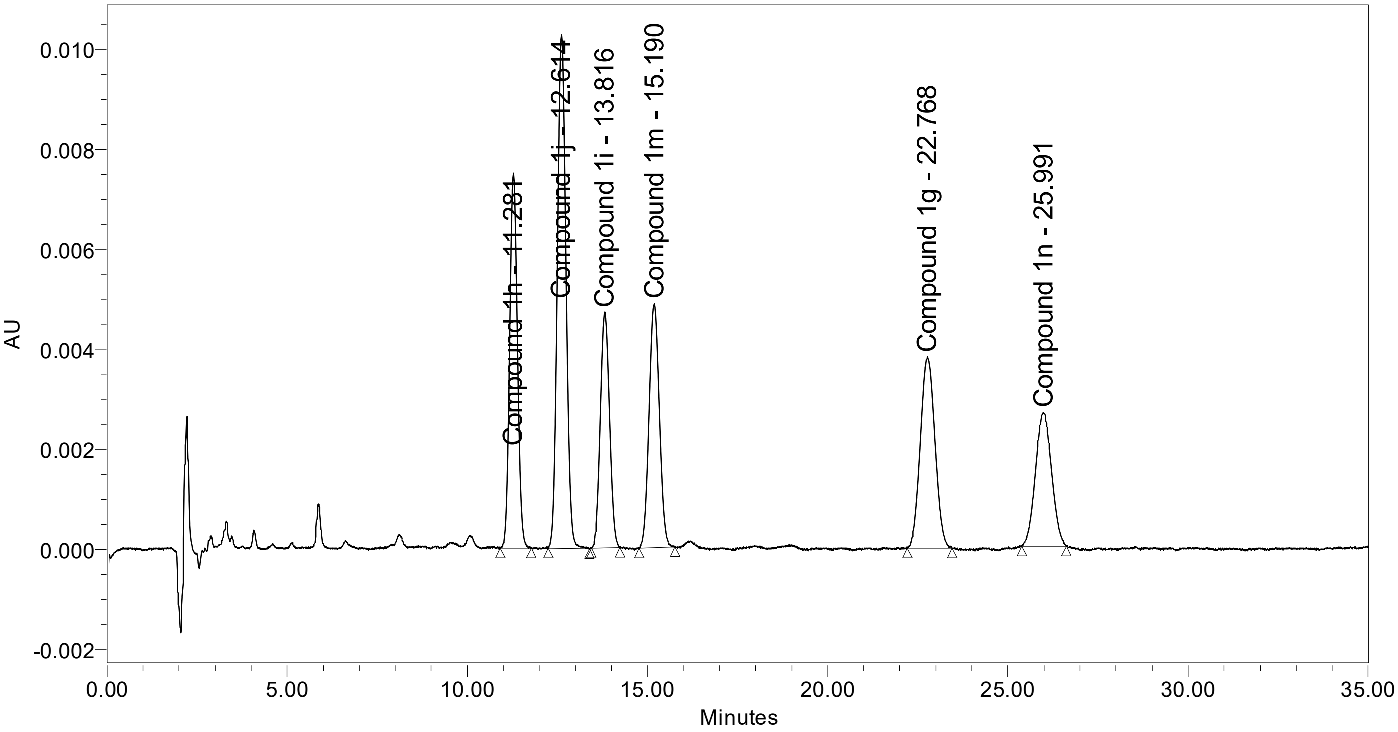

	Peak Name	RT	Area	Height (µV)	Resolution
1	Compound 1h	11.281	110663	7501	
2	Compound 1j	12.614	168785	10270	3.24
3	Compound 1i	13.816	82849	4720	2.67
4	Compound 1m	15.190	95668	4879	2.78
5	Compound 1g	22.768	107246	3831	12.02

	Peak Name	RT	Area	Height (µV)	Resolution
6	Compound 1n	25.991	83968	2673	4.05

Chloride_Ethyl_Precision 1

Reported by User: Roman Roxana (roman_roxana)
Acquisition Server Waters-cd3
Project Name: Teste\Thiazides
Sample Set Name: Seria Etil_Cl 7 93 Accuracy
Code column: Inertsil ODS-3 250*4.6 mm 5um LA140

SAMPLE INFORMATION

Sample Name: Solution 5.0 ug/ mL
Sample Type: Unknown
Vial: 12
Injection #: 1
Injection Volume: 10.00 ul
Run Time: 35.0 Minutes
Acquired By: roman_roxana

Date Acquired: 2/12/2023 1:17:02 AM EET
Acq. Method Set: Seria Etil_Cl 7 93 Accuracy
Date Processed: 4/19/2023 2:23:16 PM EEST
Processing Method: Seria Etil_Cl 7 93 Accuracy
Channel Name: Extract 275
Proc. Chnl. Descr.: 2998 PDA 275.0 nm (2998

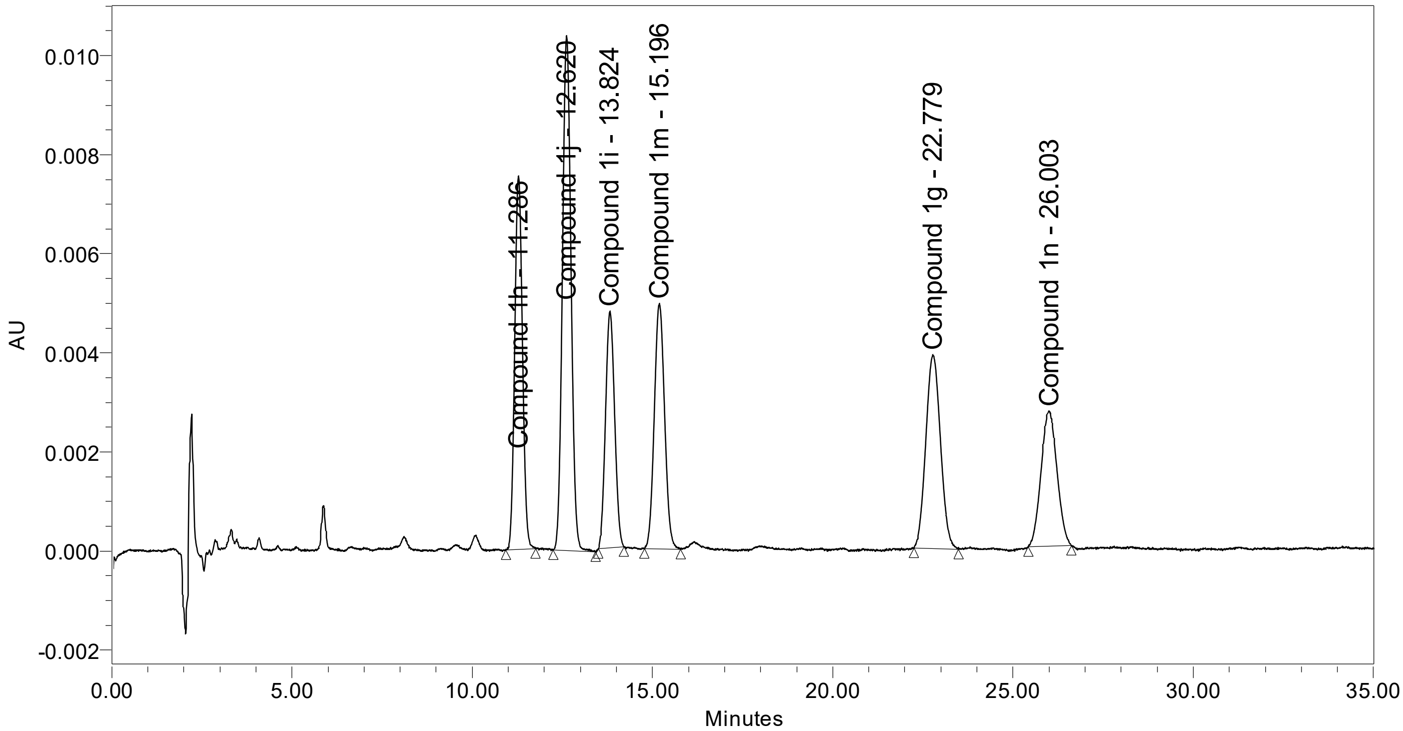

	Peak Name	RT	Area	Height (μV)	Resolution
1	Compound 1h	11.286	110997	7538	
2	Compound 1j	12.620	169796	10394	3.25
3	Compound 1i	13.824	82632	4775	2.69
4	Compound 1m	15.196	97012	4948	2.80
5	Compound 1g	22.779	108996	3908	12.07

	Peak Name	RT	Area	Height (μV)	Resolution
6	Compound 1n	26.003	84959	2724	4.08

Chloride_Ethyl_Precision 1

Reported by User: Roman Roxana (roman_roxana)
Acquisition Server Waters-cd3
Project Name: Teste\Thiazides
Sample Set Name: Seria Etil_Cl 7 93 Accuracy
Code column: Inertsil ODS-3 250*4.6 mm 5um LA140

SAMPLE INFORMATION

Sample Name:	Solution 5.0 ug/ mL		
Sample Type:	Unknown	Date Acquired:	2/12/2023 1:52:42 AM EET
Vial:	13	Acq. Method Set:	Seria Etil_Cl 7 93 Accuracy
Injection #:	1	Date Processed:	4/19/2023 2:23:44 PM EEST
Injection Volume:	10.00 ul	Processing Method:	Seria Etil_Cl 7 93 Accuracy
Run Time:	35.0 Minutes	Channel Name:	Extract 275
Acquired By:	roman_roxana	Proc. Chnl. Descr.:	2998 PDA 275.0 nm (2998

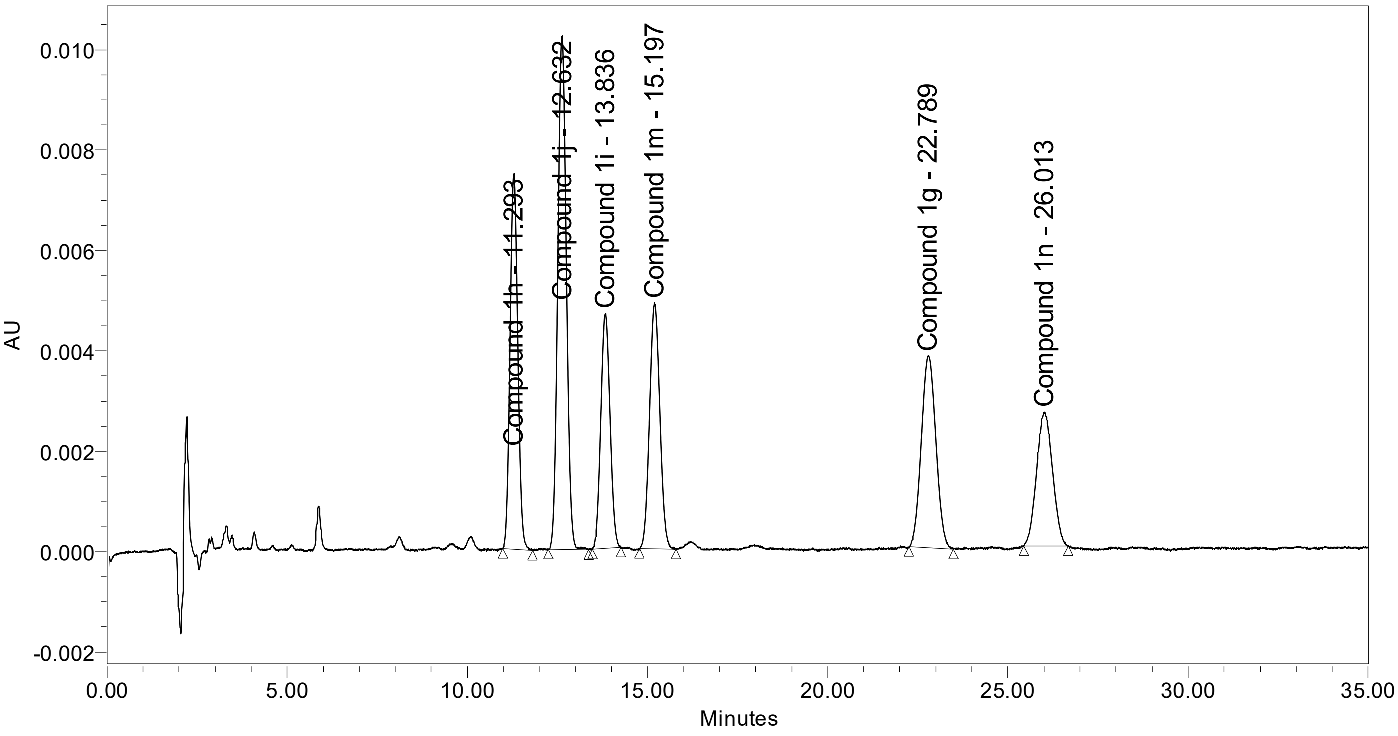

	Peak Name	RT	Area	Height (µV)	Resolution
1	Compound 1h	11.293	110417	7486	
2	Compound 1j	12.632	166835	10236	3.26
3	Compound 1i	13.836	82261	4679	2.68
4	Compound 1m	15.197	96243	4902	2.76
5	Compound 1g	22.789	106276	3822	12.03
6	Compound 1n	26.013	83531	2673	4.06

Component Summary Area Time

Reported by User: Roman Roxana (roman_roxana)
Acquisition Server Waters-cd3

Project Name: Teste\Thiazides
Sample Set Name: Seria Etil_Cl 7 93 Accuracy
Code column: Inertsil ODS-3 250*4.6 mm 5 um LA140

Retention Time Summarized by Name
Channel: 2998

	SampleName	Inj	Channel	Vial	Compound 1h	Compound 1j	Compound 1i	Compound 1m
1	Solution 5.0 ug/ mL	1	2998	8	11.270	12.596	13.789	15.167
2	Solution 5.0 ug/ mL	1	2998	9	11.272	12.602	13.801	15.168
3	Solution 5.0 ug/ mL	1	2998	10	11.276	12.612	13.818	15.178
4	Solution 5.0 ug/ mL	1	2998	11	11.281	12.614	13.816	15.190
5	Solution 5.0 ug/ mL	1	2998	12	11.286	12.620	13.824	15.196
6	Solution 5.0 ug/ mL	1	2998	13	11.293	12.632	13.836	15.197
Mean					11.280	12.613	13.814	15.183
Std. Dev.					0.009	0.013	0.017	0.014
% RSD					0.08	0.10	0.12	0.09

Retention Time Summarized by Name
Channel: 2998

	Compound 1g	Compound 1n
1	22.721	25.932
2	22.742	25.950
3	22.766	25.974
4	22.768	25.991
5	22.779	26.003
6	22.789	26.013
Mean	22.761	25.977
Std. Dev.	0.025	0.031
% RSD	0.11	0.12

Component Summary Area Time

Reported by User: Roman Roxana (roman_roxana)
Acquisition Server Waters-cd3
Project Name: Teste\Thiazides
Sample Set Name: Seria Etil_Cl 7 93 Accuracy
Code column: Inertsil ODS-3 250*4.6 mm 5 um LA140

Area Summarized by Name
Channel: 2998

	SampleName	Inj	Channel	Vial	Compound 1h	Compound 1j	Compound 1i	Compound 1m
1	Solution 5.0 ug/ mL	1	2998	8	111591	169135	82009	97170
2	Solution 5.0 ug/ mL	1	2998	9	111110	168465	82735	96179
3	Solution 5.0 ug/ mL	1	2998	10	111024	168231	82661	96381
4	Solution 5.0 ug/ mL	1	2998	11	110663	168785	82849	95668
5	Solution 5.0 ug/ mL	1	2998	12	110997	169796	82632	97012
6	Solution 5.0 ug/ mL	1	2998	13	110417	166835	82261	96243
Mean					110967	168541	82525	96442
Std. Dev.					402	1000	321	560
% RSD					0.36	0.59	0.39	0.58

Area Summarized by Name
Channel: 2998

	Compound 1g	Compound 1n
1	108491	84215
2	109245	84630
3	108577	82741
4	107246	83968
5	108996	84959
6	106276	83531
Mean	108138	84007
Std. Dev.	1144	796
% RSD	1.06	0.95

Quantitative determination of

compounds 1g, 1h, 1i, 1j, 1m, 1n

- Validation of the analytical method -

Validation parameters:

- Specificity
- Precision 1
- **Precision 2**
- LOD – LOQ
- Linearity
- Range
- Accuracy
- Robustness

Chloride_Ethyl_Precision 2

Reported by User: Roman Roxana (roman_roxana)
Acquisition Server Waters-cd3
Project Name: Teste\Thiazides
Sample Set Name: Seria Etil_Cl 7 93 Precision 2
Code column: Inertsil ODS-3 250*4.6 mm 5um LA140

SAMPLE INFORMATION

Sample Name:	Solution 5.0 ug/ mL		
Sample Type:	Unknown	Date Acquired:	2/13/2023 6:08:21 AM EET
Vial:	1	Acq. Method Set:	Seria Etil_Cl 7 93 Precision 2
Injection #:	1	Date Processed:	4/10/2023 1:38:56 PM EEST
Injection Volume:	10.00 ul	Processing Method:	Seria Etil_Cl 7 93 Precision 2
Run Time:	35.0 Minutes	Channel Name:	Extract 275
Acquired By:	roman_roxana	Proc. Chnl. Descr.:	2998 PDA 275.0 nm (2998

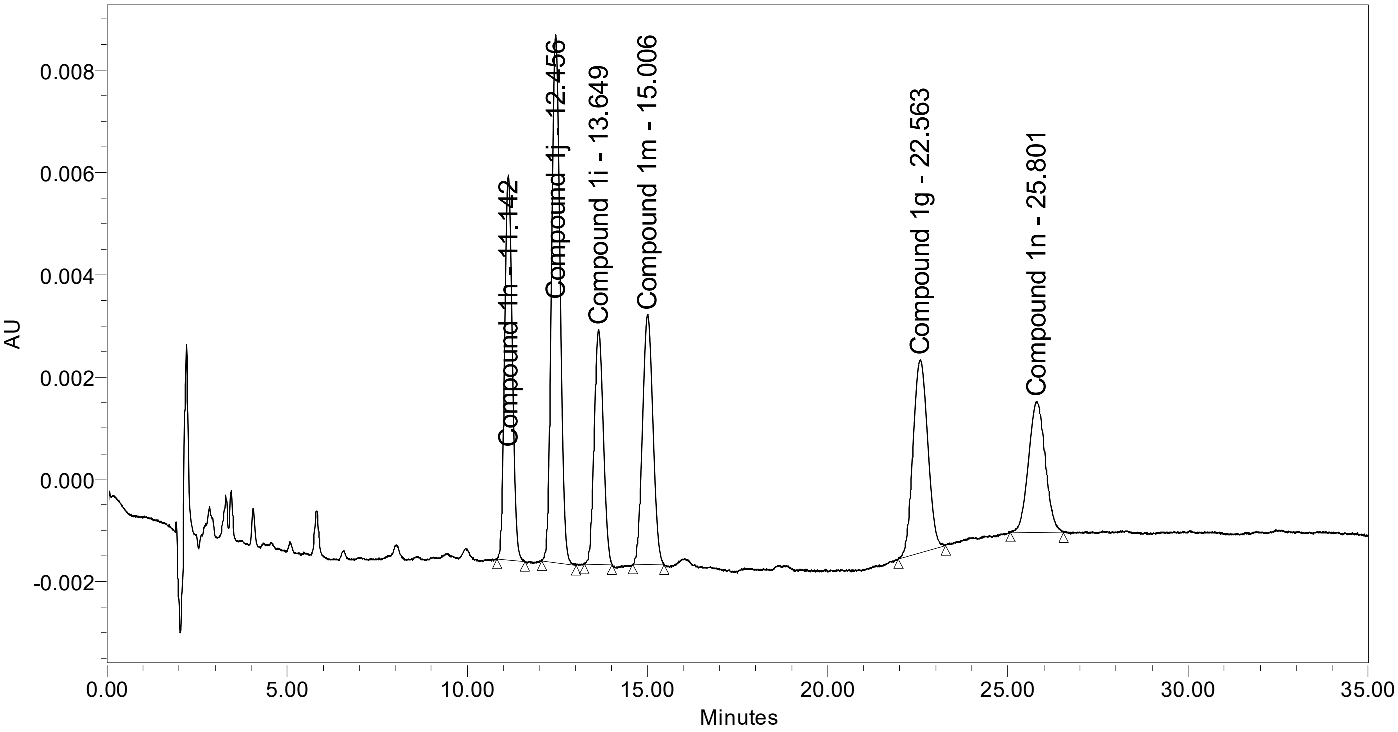

	Peak Name	RT	Area	Height (μV)	Resolution
1	Compound 1h	11.142	110842	7539	
2	Compound 1j	12.456	168780	10321	3.19
3	Compound 1i	13.649	80744	4594	2.64
4	Compound 1m	15.006	97663	4889	2.71
5	Compound 1g	22.563	106865	3776	11.76
6	Compound 1n	25.801	83077	2559	4.01

Chloride_Ethyl_Precision 2

Reported by User: Roman Roxana (roman_roxana)
Acquisition Server Waters-cd3
Project Name: Teste\Thiazides
Sample Set Name: Seria Etil_Cl 7 93 Precision 2
Code column: Inertsil ODS-3 250*4.6 mm 5um LA140

SAMPLE INFORMATION

Sample Name:	Solution 5.0 ug/ mL		
Sample Type:	Unknown	Date Acquired:	2/13/2023 6:44:03 AM EET
Vial:	2	Acq. Method Set:	Seria Etil_Cl 7 93 Precision 2
Injection #:	1	Date Processed:	4/10/2023 1:33:54 PM EEST
Injection Volume:	10.00 ul	Processing Method:	Seria Etil_Cl 7 93 Precision 2
Run Time:	35.0 Minutes	Channel Name:	Extract 275
Acquired By:	roman_roxana	Proc. Chnl. Descr.:	2998 PDA 275.0 nm (2998

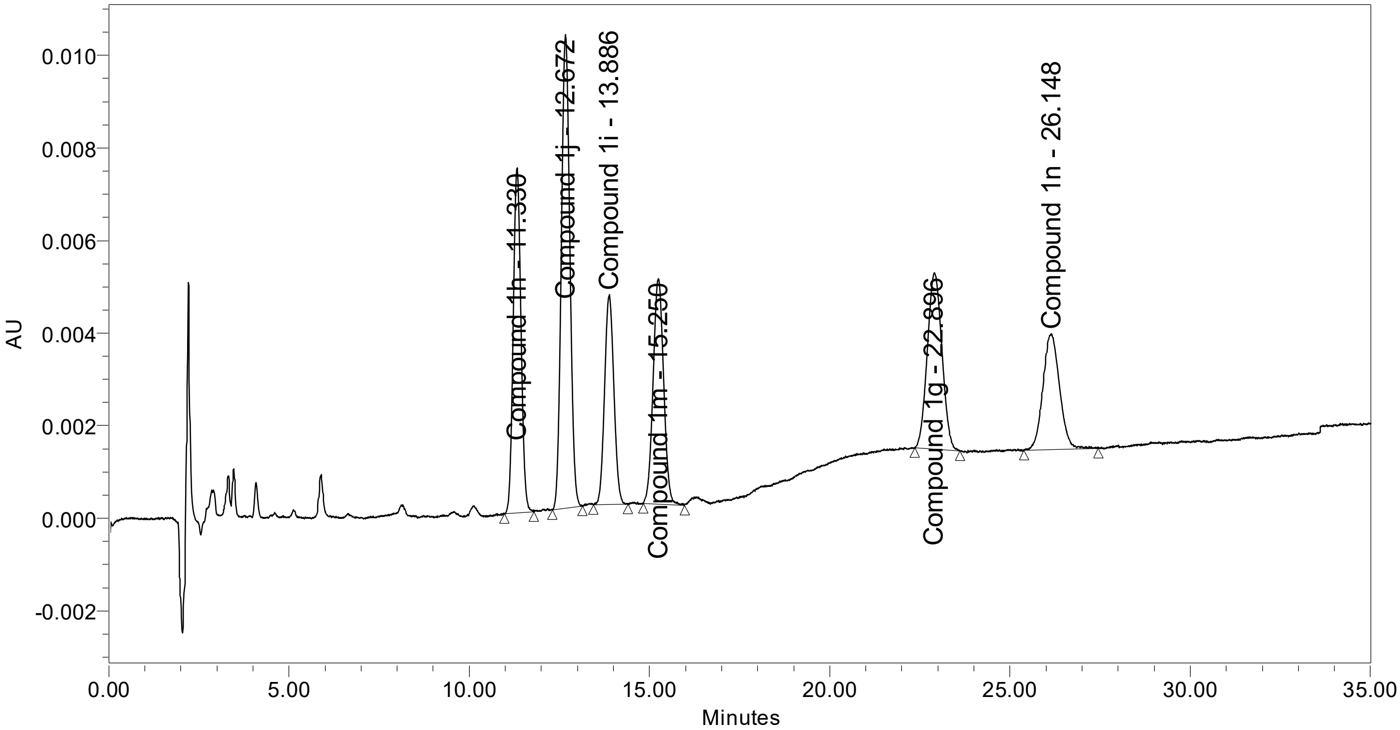

	Peak Name	RT	Area	Height (µV)	Resolution
1	Compound 1h	11.330	109970	7449	
2	Compound 1j	12.672	166002	10235	3.28
3	Compound 1i	13.886	79884	4529	2.70
4	Compound 1m	15.250	97797	4877	2.74
5	Compound 1g	22.896	106337	3808	12.01
6	Compound 1n	26.148	81794	2506	4.08

Chloride_Ethyl_Precision 2

Reported by User: Roman Roxana (roman_roxana)
Acquisition Server Waters-cd3
Project Name: Teste\Thiazides
Sample Set Name: Seria Etil_Cl 7 93 Precision 2
Code column: Inertsil ODS-3 250*4.6 mm 5um LA140

SAMPLE INFORMATION

Sample Name:	Solution 5.0 ug/ mL		
Sample Type:	Unknown	Date Acquired:	2/13/2023 7:19:56 AM EET
Vial:	3	Acq. Method Set:	Seria Etil_Cl 7 93 Precision 2
Injection #:	1	Date Processed:	4/10/2023 1:34:26 PM EEST
Injection Volume:	10.00 ul	Processing Method:	Seria Etil_Cl 7 93 Precision 2
Run Time:	35.0 Minutes	Channel Name:	Extract 275
Acquired By:	roman_roxana	Proc. Chnl. Descr.:	2998 PDA 275.0 nm (2998

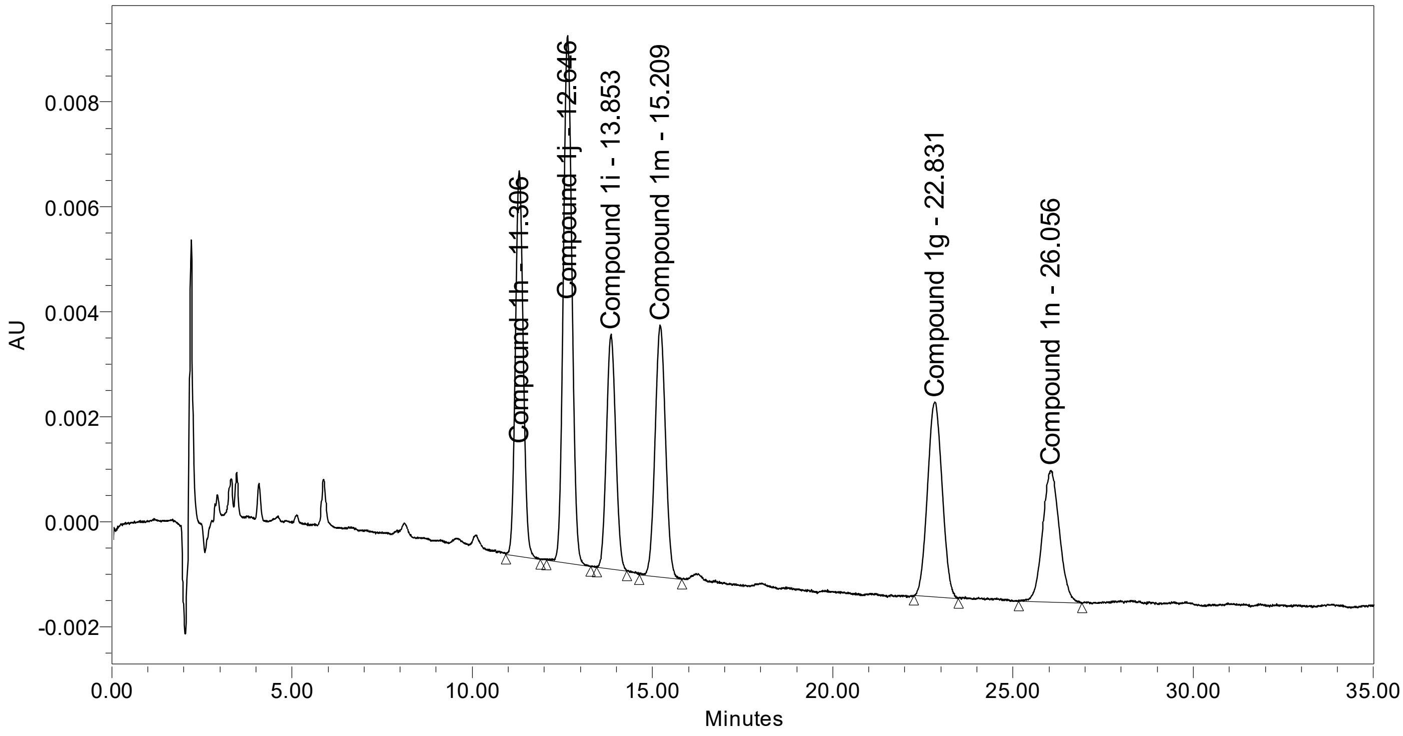

	Peak Name	RT	Area	Height (µV)	Resolution
1	Compound 1h	11.306	109387	7344	
2	Compound 1j	12.646	165117	10061	3.26
3	Compound 1i	13.853	79171	4474	2.68
4	Compound 1m	15.209	96058	4803	2.72
5	Compound 1g	22.831	104014	3717	11.97

	Peak Name	RT	Area	Height (µV)	Resolution
6	Compound 1n	26.056	82753	2513	4.01

Chloride_Ethyl_Precision 2

Reported by User: Roman Roxana (roman_roxana)
Acquisition Server Waters-cd3
Project Name: Teste\Thiazides
Sample Set Name: Seria Etil_Cl 7 93 Precision 2
Code column: Inertsil ODS-3 250*4.6 mm 5um LA140

SAMPLE INFORMATION

Sample Name:	Solution 5.0 ug/ mL		
Sample Type:	Unknown	Date Acquired:	2/13/2023 7:55:39 AM EET
Vial:	4	Acq. Method Set:	Seria Etil_Cl 7 93 Precision 2
Injection #:	1	Date Processed:	4/10/2023 1:49:56 PM EEST
Injection Volume:	10.00 ul	Processing Method:	Seria Etil_Cl 7 93 Precision 2
Run Time:	35.0 Minutes	Channel Name:	Extract 275
Acquired By:	roman_roxana	Proc. Chnl. Descr.:	2998 PDA 275.0 nm (2998

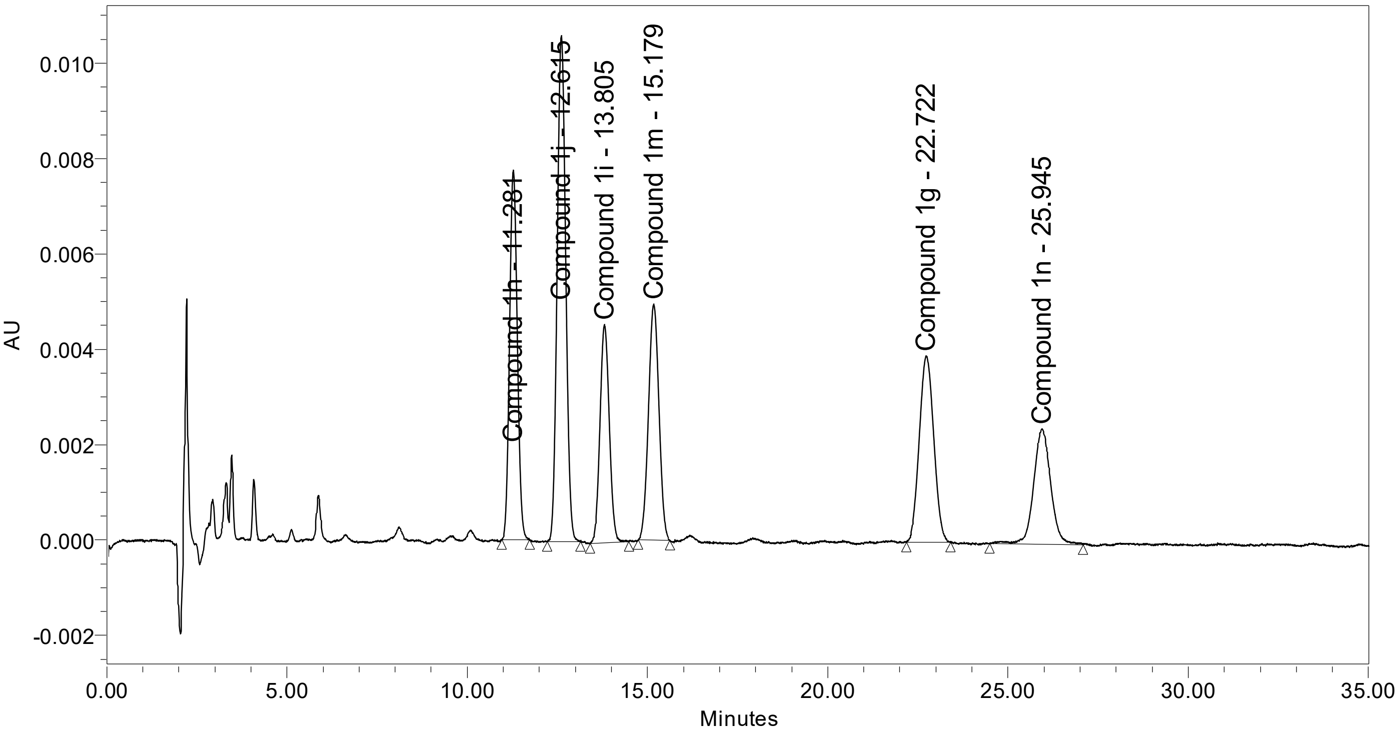

	Peak Name	RT	Area	Height (µV)	Resolution
1	Compound 1h	11.281	114244	7758	
2	Compound 1j	12.615	172220	10613	3.26
3	Compound 1i	13.805	81127	4570	2.65
4	Compound 1m	15.179	98678	4944	2.75
5	Compound 1g	22.722	108103	3909	11.86

	Peak Name	RT	Area	Height (µV)	Resolution
6	Compound 1n	25.945	80985	2417	4.04

Chloride_Ethyl_Precision 2

Reported by User: Roman Roxana (roman_roxana)
Acquisition Server Waters-cd3
Project Name: Teste\Thiazides
Sample Set Name: Seria Etil_Cl 7 93 Precision 2
Code column: Inertsil ODS-3 250*4.6 mm 5um LA140

SAMPLE INFORMATION

Sample Name:	Solution 5.0 ug/ mL		
Sample Type:	Unknown	Date Acquired:	2/13/2023 8:31:33 AM EET
Vial:	5	Acq. Method Set:	Seria Etil_Cl 7 93 Precision 2
Injection #:	1	Date Processed:	4/10/2023 1:38:16 PM EEST
Injection Volume:	10.00 ul	Processing Method:	Seria Etil_Cl 7 93 Precision 2
Run Time:	35.0 Minutes	Channel Name:	Extract 275
Acquired By:	roman_roxana	Proc. Chnl. Descr.:	2998 PDA 275.0 nm (2998

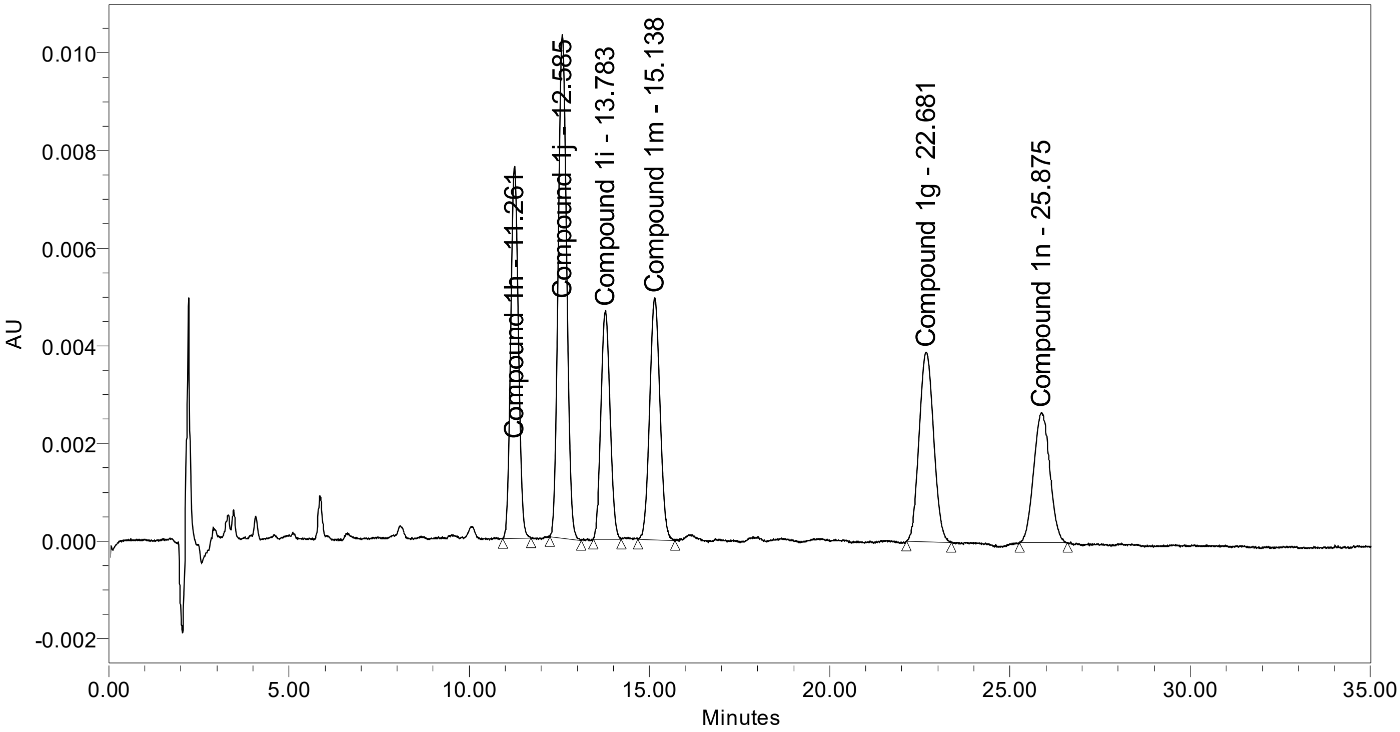

	Peak Name	RT	Area	Height (µV)	Resolution
1	Compound 1h	11.261	111967	7620	
2	Compound 1j	12.585	166791	10317	3.25
3	Compound 1i	13.783	82099	4682	2.68
4	Compound 1m	15.138	98075	4962	2.75
5	Compound 1g	22.681	108183	3889	11.98

	Peak Name	RT	Area	Height (µV)	Resolution
6	Compound 1n	25.875	84121	2671	4.03

Chloride_Ethyl_Precision 2

Reported by User: Roman Roxana (roman_roxana)
Acquisition Server Waters-cd3
Project Name: Teste\Thiazides
Sample Set Name: Seria Etil_Cl 7 93 Precision 2
Code column: Inertsil ODS-3 250*4.6 mm 5um LA140

SAMPLE INFORMATION

Sample Name:	Solution 5.0 ug/ mL		
Sample Type:	Unknown	Date Acquired:	2/13/2023 9:07:12 AM EET
Vial:	6	Acq. Method Set:	Seria Etil_Cl 7 93 Precision 2
Injection #:	1	Date Processed:	4/10/2023 1:40:21 PM EEST
Injection Volume:	10.00 ul	Processing Method:	Seria Etil_Cl 7 93 Precision 2
Run Time:	35.0 Minutes	Channel Name:	Extract 275
Acquired By:	roman_roxana	Proc. Chnl. Descr.:	2998 PDA 275.0 nm (2998

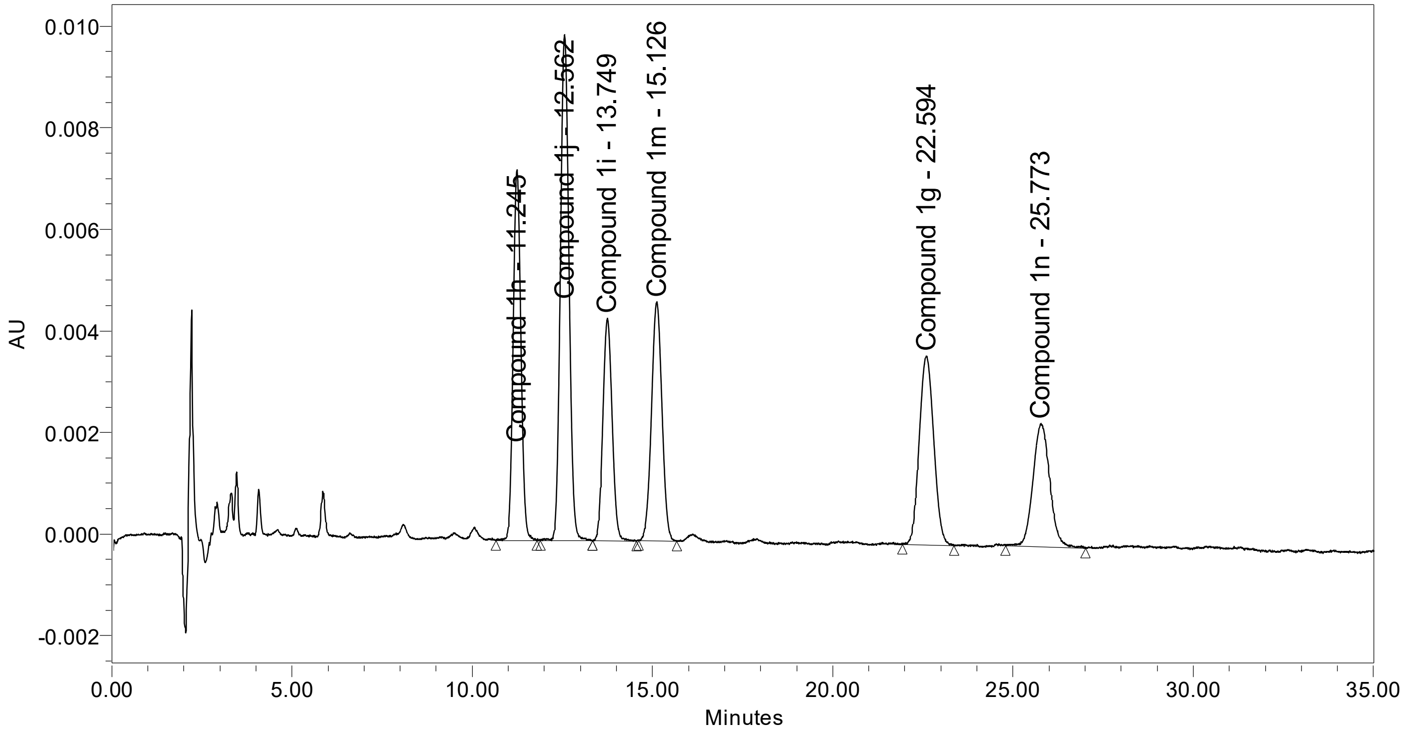

	Peak Name	RT	Area	Height (µV)	Resolution
1	Compound 1h	11.245	108129	7304	
2	Compound 1j	12.562	163043	9968	3.23
3	Compound 1i	13.749	77962	4390	2.65
4	Compound 1m	15.126	94606	4712	2.77
5	Compound 1g	22.594	103635	3726	11.81

	Peak Name	RT	Area	Height (µV)	Resolution
6	Compound 1n	25.773	79668	2431	4.01

Component Summary Area Time

Reported by User: Roman Roxana (roman_roxana)
Acquisition Server Waters-cd3
Project Name: Teste\Thiazides
Sample Set Name: Seria Etil_Cl 7 93 Precision 2
Code column: Inertsil ODS-3 250*4.6 mm 5 um LA140

Retention Time Summarized by Name
Channel: 2998

	SampleName	Inj	Channel	Vial	Compound 1h	Compound 1j	Compound 1i	Compound 1m
1	Solution 5.0 ug/ mL	1	2998	1	11.142	12.456	13.649	15.006
2	Solution 5.0 ug/ mL	1	2998	2	11.330	12.672	13.886	15.250
3	Solution 5.0 ug/ mL	1	2998	3	11.306	12.646	13.853	15.209
4	Solution 5.0 ug/ mL	1	2998	4	11.281	12.615	13.805	15.179
5	Solution 5.0 ug/ mL	1	2998	5	11.261	12.585	13.783	15.138
6	Solution 5.0 ug/ mL	1	2998	6	11.245	12.562	13.749	15.126
Mean					11.261	12.589	13.787	15.151
Std. Dev.					0.066	0.076	0.084	0.085
% RSD					0.58	0.61	0.61	0.56

Retention Time Summarized by Name
Channel: 2998

	Compound 1g	Compound 1n
1	22.563	25.801
2	22.896	26.148
3	22.831	26.056
4	22.722	25.945
5	22.681	25.875
6	22.594	25.773
Mean	22.714	25.933
Std. Dev.	0.131	0.147
% RSD	0.57	0.57

Component Summary Area Time

Reported by User: Roman Roxana (roman_roxana)
Acquisition Server Waters-cd3

Project Name: Teste\Thiazides
Sample Set Name: Seria Etil_Cl 7 93 Precision 2
Code column: Inertsil ODS-3 250*4.6 mm 5 um LA140

Area Summarized by Name
Channel: 2998

	SampleName	Inj	Channel	Vial	Compound 1h	Compound 1j	Compound 1i	Compound 1m
1	Solution 5.0 ug/ mL	1	2998	1	110842	168780	80744	97663
2	Solution 5.0 ug/ mL	1	2998	2	109970	166002	79884	97797
3	Solution 5.0 ug/ mL	1	2998	3	109387	165117	79171	96058
4	Solution 5.0 ug/ mL	1	2998	4	114244	172220	81127	98678
5	Solution 5.0 ug/ mL	1	2998	5	111967	166791	82099	98075
6	Solution 5.0 ug/ mL	1	2998	6	108129	163043	77962	94606
Mean					110757	166992	80164	97146
Std. Dev.					2147	3183	1478	1519
% RSD					1.94	1.91	1.84	1.56

Area Summarized by Name
Channel: 2998

	Compound 1g	Compound 1n
1	106865	83077
2	106337	81794
3	104014	82753
4	108103	80985
5	108183	84121
6	103635	79668
Mean	106189	82066
Std. Dev.	1968	1594
% RSD	1.85	1.94

Component Summary Area Time

Reported by User: Roman Roxana (roman_roxana)
Acquisition Server Waters-cd3

Project Name: Teste\Thiazides
Sample Set Name: Seria Etil_Cl 7 93 Precision 2, Seria Etil_Cl 7 93 Accuracy
Code column: Inertsil ODS-3 250*4.6 mm 5 um LA140

Retention Time Summarized by Name
Channel: 2998

	SampleName	Inj	Channel	Vial	Compound 1h	Compound 1j	Compound 1i	Compound 1m
1	Solution 5.0 ug/mL	1	2998	8	11.270	12.596	13.789	15.167
2	Solution 5.0 ug/mL	1	2998	9	11.272	12.602	13.801	15.168
3	Solution 5.0 ug/mL	1	2998	10	11.276	12.612	13.818	15.178
4	Solution 5.0 ug/mL	1	2998	11	11.281	12.614	13.816	15.190
5	Solution 5.0 ug/mL	1	2998	12	11.286	12.620	13.824	15.196
6	Solution 5.0 ug/mL	1	2998	13	11.293	12.632	13.836	15.197
7	Solution 5.0 ug/mL	1	2998	1	11.142	12.456	13.649	15.006
8	Solution 5.0 ug/mL	1	2998	2	11.330	12.672	13.886	15.250
9	Solution 5.0 ug/mL	1	2998	3	11.306	12.646	13.853	15.209
10	Solution 5.0 ug/mL	1	2998	4	11.281	12.615	13.805	15.179
11	Solution 5.0 ug/mL	1	2998	5	11.261	12.585	13.783	15.138
12	Solution 5.0 ug/mL	1	2998	6	11.245	12.562	13.749	15.126
Mean					11.270	12.601	13.801	15.167
Std. Dev.					0.046	0.054	0.059	0.060
% RSD					0.40	0.43	0.43	0.40

Retention Time Summarized by Name
Channel: 2998

	Compound 1g	Compound 1n
1	22.721	25.932
2	22.742	25.950
3	22.766	25.974
4	22.768	25.991
5	22.779	26.003
6	22.789	26.013
7	22.563	25.801
8	22.896	26.148
9	22.831	26.056

	Compound 1g	Compound 1n
10	22.722	25.945
11	22.681	25.875
12	22.594	25.773
Mean	22.738	25.955
Std. Dev.	0.093	0.104
% RSD	0.41	0.40

Component Summary Area Time

Reported by User: Roman Roxana (roman_roxana)
Acquisition Server Waters-cd3

Project Name: Teste\Thiazides
Sample Set Name: Seria Etil_Cl 7 93 Precision 2, Seria Etil_Cl 7 93 Accuracy
Code column: Inertsil ODS-3 250*4.6 mm 5 um LA140

Area Summarized by Name
Channel: 2998

	SampleName	Inj	Channel	Vial	Compound 1h	Compound 1j	Compound 1i	Compound 1m
1	Solution 5.0 ug/ mL	1	2998	8	111591	169135	82009	97170
2	Solution 5.0 ug/ mL	1	2998	9	111110	168465	82735	96179
3	Solution 5.0 ug/ mL	1	2998	10	111024	168231	82661	96381
4	Solution 5.0 ug/ mL	1	2998	11	110663	168785	82849	95668
5	Solution 5.0 ug/ mL	1	2998	12	110997	169796	82632	97012
6	Solution 5.0 ug/ mL	1	2998	13	110417	166835	82261	96243
7	Solution 5.0 ug/ mL	1	2998	1	110842	168780	80744	97663
8	Solution 5.0 ug/ mL	1	2998	2	109970	166002	79884	97797
9	Solution 5.0 ug/ mL	1	2998	3	109387	165117	79171	96058
10	Solution 5.0 ug/ mL	1	2998	4	114244	172220	81127	98678
11	Solution 5.0 ug/ mL	1	2998	5	111967	166791	82099	98075
12	Solution 5.0 ug/ mL	1	2998	6	108129	163043	77962	94606
Mean					110862	167767	81345	96794
Std. Dev.					1476	2390	1600	1152
% RSD					1.33	1.42	1.97	1.19

Area Summarized by Name
Channel: 2998

	Compound 1g	Compound 1n
1	108491	84215
2	109245	84630
3	108577	82741
4	107246	83968
5	108996	84959
6	106276	83531
7	106865	83077
8	106337	81794
9	104014	82753

	Compound 1g	Compound 1n
10	108103	80985
11	108183	84121
12	103635	79668
Mean	107164	83037
Std. Dev.	1842	1572
% RSD	1.72	1.89

Quantitative determination of

compounds 1g, 1h, 1i, 1j, 1m, 1n

- Validation of the analytical method -

Validation parameters:

- Specificity
- Precision 1
- Precision 2
- **LOD – LOQ**
- Linearity
- Range
- Accuracy
- Robustness

Calibration Curve

Reported by User: Roman Roxana (roman_roxana)
Acquisition Server Waters-cd3
Project Name: Teste\Thiazides
Sample Set Name: Seria Etil_Cl 7 93 LOD LOQ
Code column: Inertsil ODS-3, 4,6*250 mm, 5 um

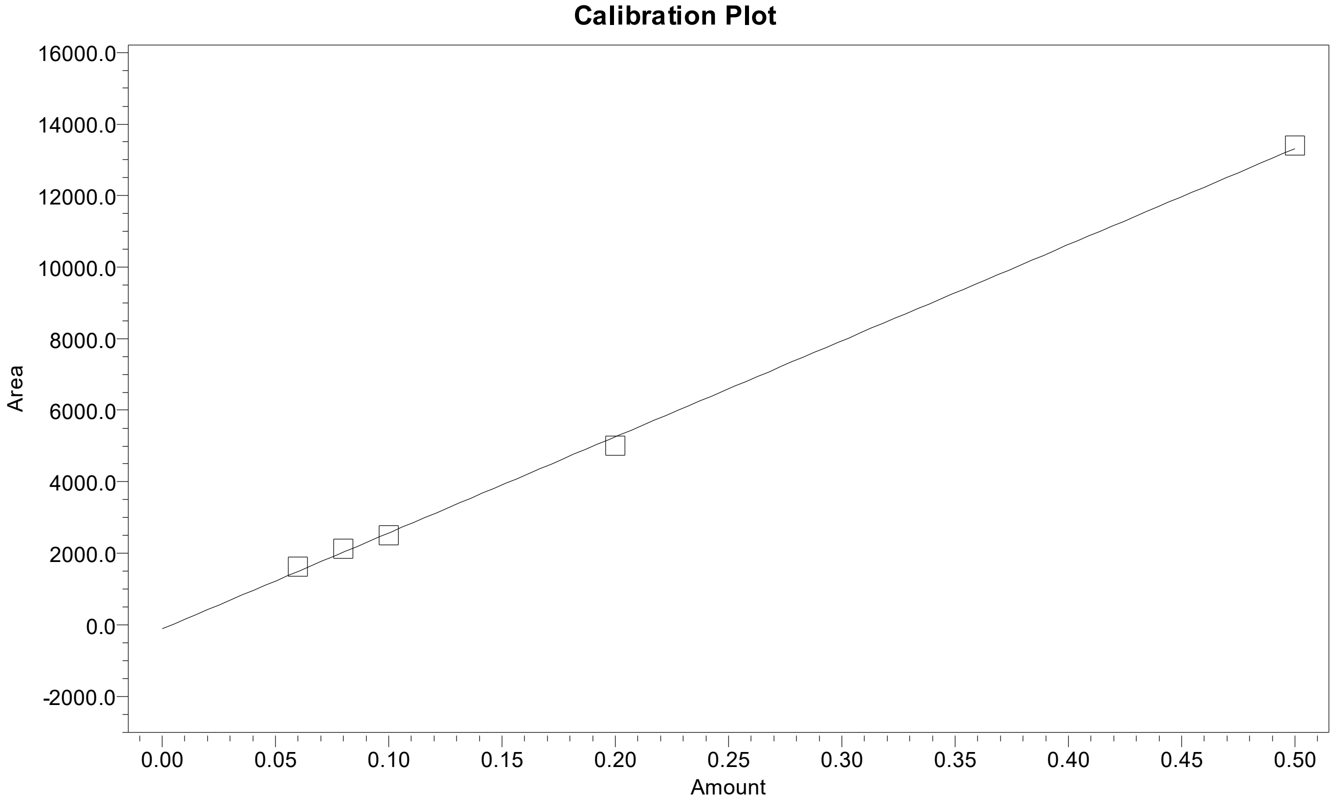

Name: Compound 1j; Processing Method: Seria Etil_Cl 7 93 LOD LOQ; Fit Type: Linear (1st Order); Cal Curve Id: 3429; A: -1.175618e+002; B: 2.686268e+004; C: 0.000000e+000; D: 0.000000e+000; R^2: 0.998949; Standard Error 1.836842e+002

Calibration Curve

Reported by User: Roman Roxana (roman_roxana)
Acquisition Server Waters-cd3
Project Name: Teste\Thiazides
Sample Set Name: Seria Etil_Cl 7 93 LOD LOQ
Code column: Inertsil ODS-3, 4,6*250 mm, 5 um

Calibration Plot

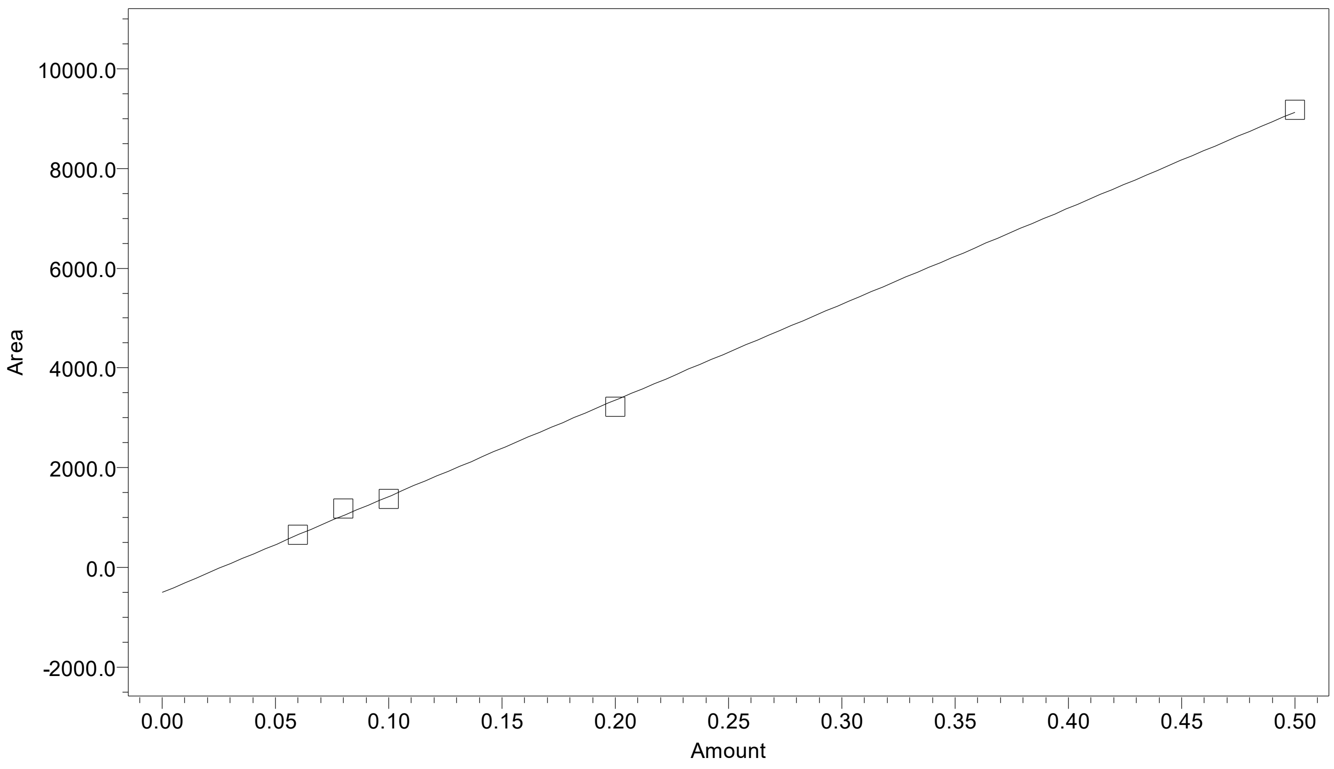

Name: Compound 1i; Processing Method: Seria Etil_Cl 7 93 LOD LOQ; Fit Type: Linear (1st Order); Cal Curve Id: 3430; A: -5.017253e+002; B: 1.926590e+004; C: 0.000000e+000; D: 0.000000e+000; R^2: 0.999152; Standard Error 1.183270e+002

Calibration Curve

Reported by User: Roman Roxana (roman_roxana)
Acquisition Server Waters-cd3
Project Name: Teste\Thiazides
Sample Set Name: Seria Etil_Cl 7 93 LOD LOQ
Code column: Inertsil ODS-3, 4,6*250 mm, 5 um

Calibration Plot

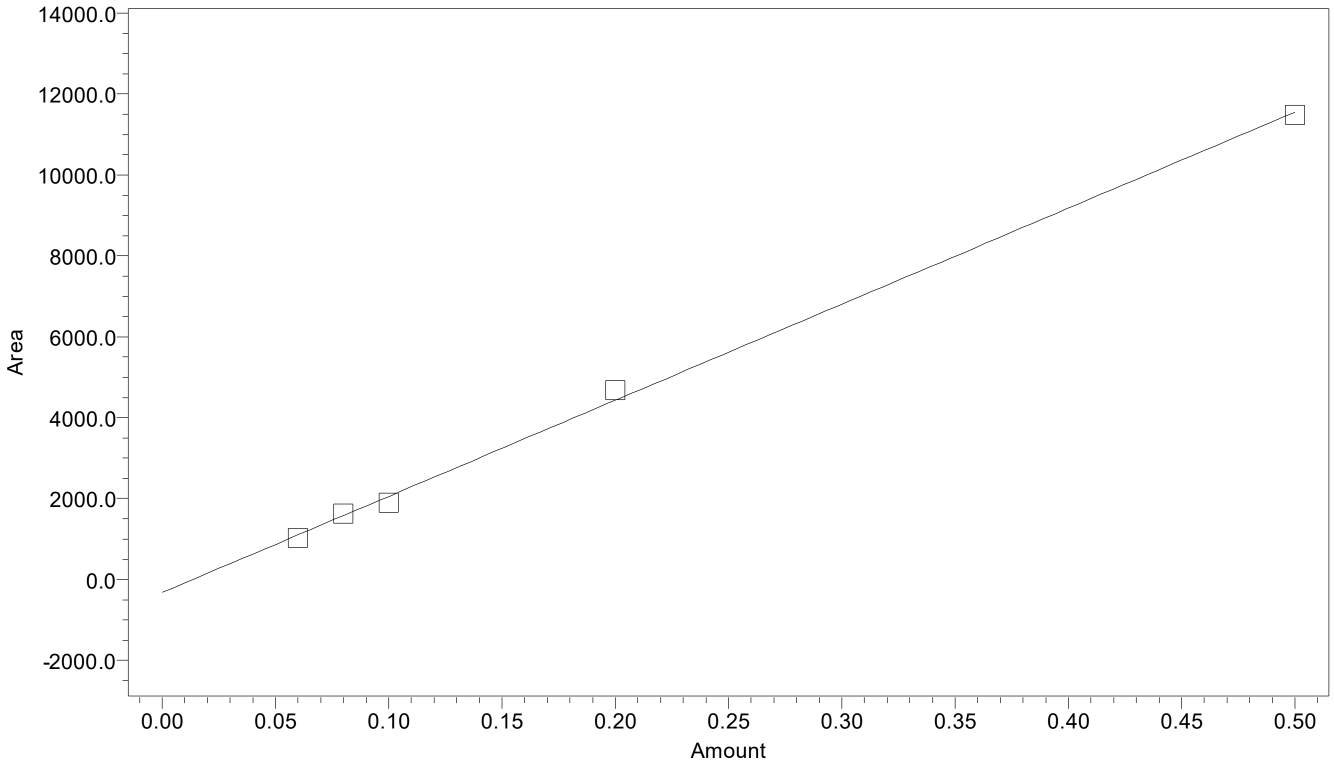

Name: Compound 1h; Processing Method: Seria Etil_Cl 7 93 LOD LOQ; Fit Type: Linear (1st Order); Cal Curve Id: 3428; A: -3.199665e+002; B: 2.375089e+004; C: 0.000000e+000; D: 0.000000e+000; R^2: 0.998684; Standard Error 1.817157e+002

Calibration Curve

Reported by User: Roman Roxana (roman_roxana)
Acquisition Server Waters-cd3
Project Name: Teste\Thiazides
Sample Set Name: Seria Etil_Cl 7 93 LOD LOQ
Code column: Inertsil ODS-3, 4,6*250 mm, 5 um

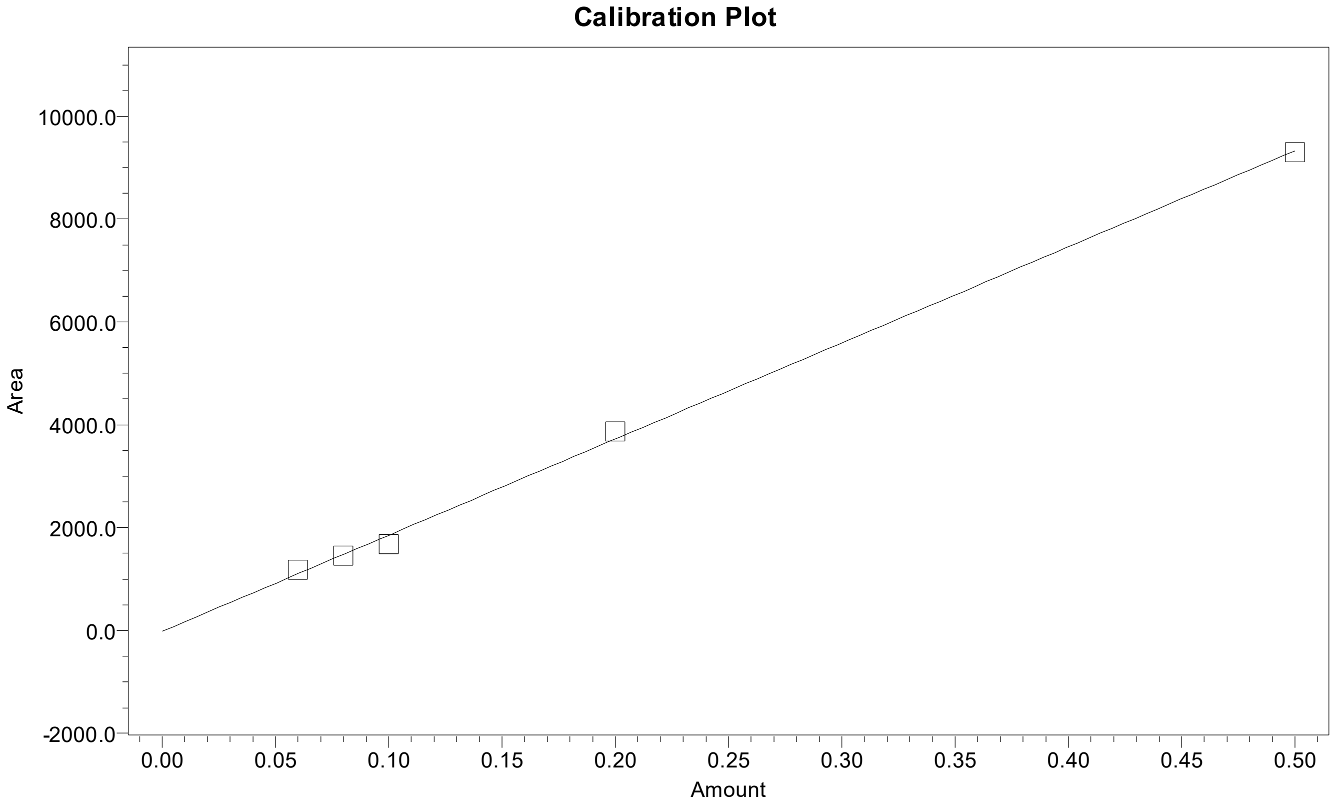

Name: Compound 1m; Processing Method: Seria Etil_Cl 7 93 LOD LOQ; Fit Type: Linear (1st Order); Cal Curve Id: 3431; A: -1.678743e+001; B: 1.869635e+004; C: 0.000000e+000; D: 0.000000e+000; R^2: 0.998752; Standard Error 1.393188e+002

Calibration Curve

Reported by User: Roman Roxana (roman_roxana)
Acquisition Server Waters-cd3
Project Name: Teste\Thiazides
Sample Set Name: Seria Etil_Cl 7 93 LOD LOQ
Code column: Inertsil ODS-3, 4,6*250 mm, 5 um

Calibration Plot

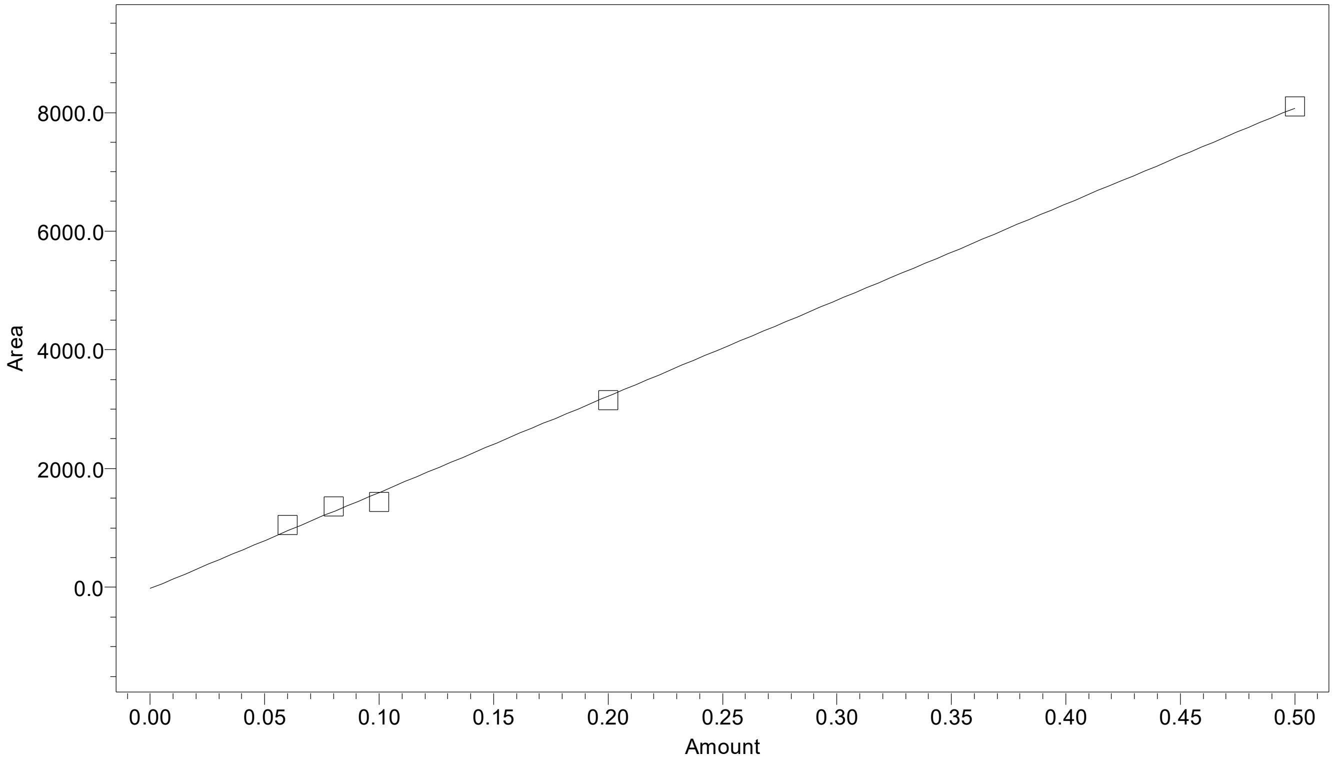

Name: Compound 1g; Processing Method: Seria Etil_Cl 7 93 LOD LOQ; Fit Type: Linear (1st Order); Cal Curve Id: 3432; A: -2.012483e+001; B: 1.618843e+004; C: 0.000000e+000; D: 0.000000e+000; R^2: 0.998612; Standard Error 1.272319e+002

Calibration Curve

Reported by User: Roman Roxana (roman_roxana)
Acquisition Server Waters-cd3
Project Name: Teste\Thiazides
Sample Set Name: Seria Etil_Cl 7 93 LOD LOQ
Code column: Inertsil ODS-3, 4,6*250 mm, 5 um

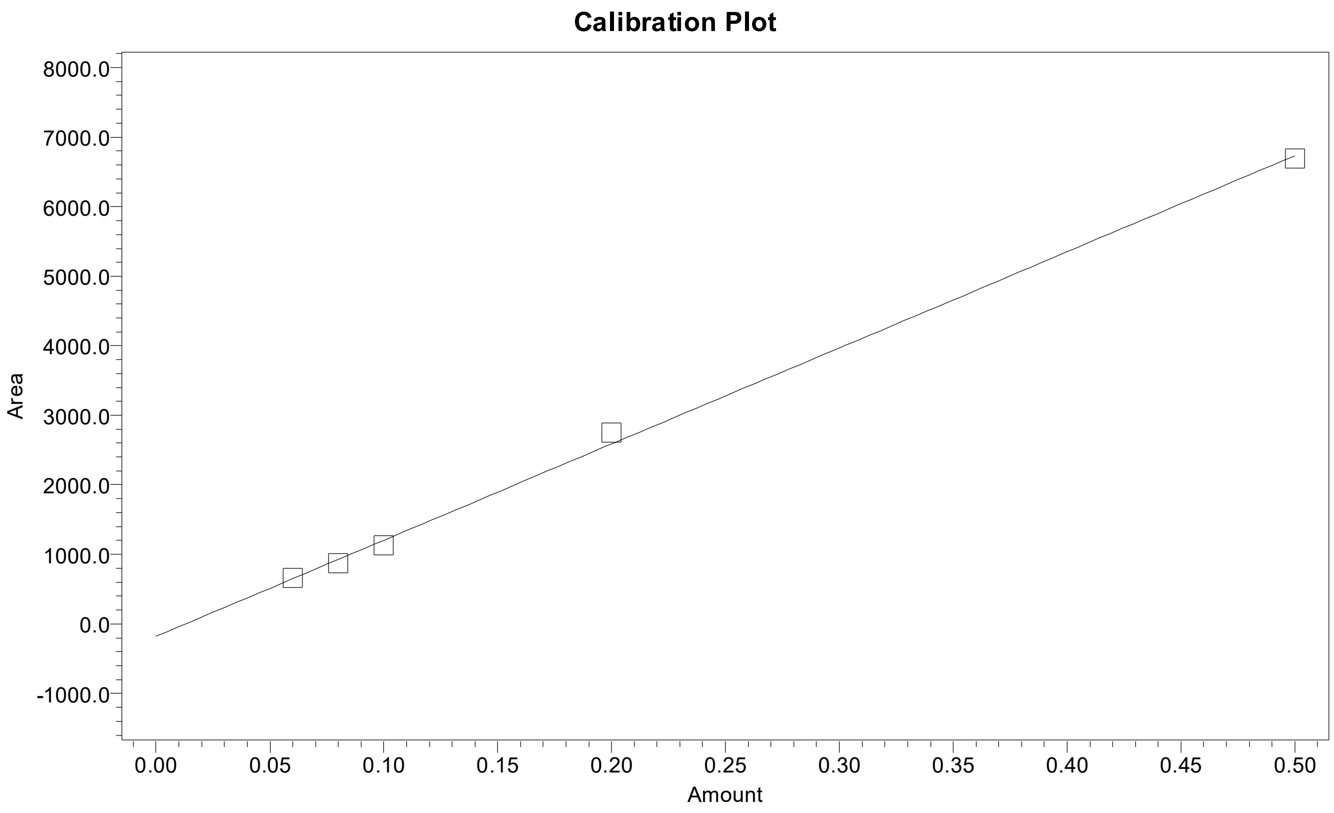

Name: Compound 1n; Processing Method: Seria Etil_Cl 7 93 LOD LOQ; Fit Type: Linear (1st Order); Cal Curve Id: 3433; A: -1.795412e+002; B: 1.382725e+004; C: 0.000000e+000; D: 0.000000e+000; R^2: 0.998571; Standard Error 1.102460e+002

Calibration Curve

Reported by User: Roman Roxana (roman_roxana)
Acquisition Server Waters-cd3
Project Name: Teste\Thiazides
Sample Set Name: Seria Etil_Cl 7 93 LOD LOQ
Code column: Inertsil ODS-3, 4,6*250 mm, 5 um

Peak: Compound 1j

	Name	Level	X Value	Response	Calc. Value	% Deviation	Manual	Ignore
1	Compound 1j	1.2	0.060000	1630.472851	0.065073	8.455	No	No
2	Compound 1j	1.6	0.080000	2131.073691	0.083709	4.636	No	No
3	Compound 1j	2	0.100000	2500.255530	0.097452	-2.548	No	No
4	Compound 1j	4	0.200000	5006.904369	0.190765	-4.617	No	No
5	Compound 1j	10	0.500000	13394.404392	0.503001	0.600	No	No

Peak: Compound 1i

	Name	Level	X Value	Response	Calc. Value	% Deviation	Manual	Ignore
1	Compound 1i	1.2	0.060000	658.292950	0.060211	0.352	No	No
2	Compound 1i	1.6	0.080000	1179.678589	0.087274	9.092	No	No
3	Compound 1i	2	0.100000	1374.135930	0.097367	-2.633	No	No
4	Compound 1i	4	0.200000	3216.945031	0.193018	-3.491	No	No
5	Compound 1i	10	0.500000	9172.262513	0.502130	0.426	No	No

Calibration Curve

Reported by User: Roman Roxana (roman_roxana)
Acquisition Server Waters-cd3
Project Name: Teste\Thiazides
Sample Set Name: Seria Etil_Cl 7 93 LOD LOQ
Code column: Inertsil ODS-3, 4,6*250 mm, 5 um

Peak: Compound 1h

	Name	Level	X Value	Response	Calc. Value	% Deviation	Manual	Ignore
1	Compound 1h	1.2	0.060000	1033.810066	0.056999	-5.002	No	No
2	Compound 1h	1.6	0.080000	1626.691286	0.081961	2.452	No	No
3	Compound 1h	2	0.100000	1897.291020	0.093355	-6.645	No	No
4	Compound 1h	4	0.200000	4679.969503	0.210516	5.258	No	No
5	Compound 1h	10	0.500000	11488.238406	0.497169	-0.566	No	No

Peak: Compound 1m

	Name	Level	X Value	Response	Calc. Value	% Deviation	Manual	Ignore
1	Compound 1m	1.2	0.060000	1178.133697	0.063912	6.520	No	No
2	Compound 1m	1.6	0.080000	1459.015714	0.078935	-1.331	No	No
3	Compound 1m	2	0.100000	1681.569370	0.090839	-9.161	No	No
4	Compound 1m	4	0.200000	3871.449351	0.207968	3.984	No	No
5	Compound 1m	10	0.500000	9300.465892	0.498346	-0.331	No	No

Calibration Curve

Reported by User: Roman Roxana (roman_roxana)
Acquisition Server Waters-cd3
Project Name: Teste\Thiazides
Sample Set Name: Seria Etil_Cl 7 93 LOD LOQ
Code column: Inertsil ODS-3, 4,6*250 mm, 5 um

Peak: Compound 1g

	Name	Level	X Value	Response	Calc. Value	% Deviation	Manual	Ignore
1	Compound 1g	1.2	0.060000	1052.049420	0.066231	10.385	No	No
2	Compound 1g	1.6	0.080000	1365.145509	0.085572	6.965	No	No
3	Compound 1g	2	0.100000	1438.295635	0.090090	-9.910	No	No
4	Compound 1g	4	0.200000	3157.245813	0.196274	-1.863	No	No
5	Compound 1g	10	0.500000	8103.768340	0.501833	0.367	No	No

Peak: Compound 1n

	Name	Level	X Value	Response	Calc. Value	% Deviation	Manual	Ignore
1	Compound 1n	1.2	0.060000	657.712692	0.060551	0.918	No	No
2	Compound 1n	1.6	0.080000	872.309723	0.076071	-4.911	No	No
3	Compound 1n	2	0.100000	1130.181323	0.094720	-5.280	No	No
4	Compound 1n	4	0.200000	2748.131849	0.211732	5.866	No	No
5	Compound 1n	10	0.500000	6691.573399	0.496926	-0.615	No	No

LOD_LOQ_Cl_Report

Reported by User: Roman Roxana (roman_roxana)
Acquisition Server Waters-cd3
Project Name: Teste\Thiazides
Sample Set Name: Seria Etil_Cl 7 93 LOD LOQ
Code column: Inertsil ODS-3 250*4.6 mm 5um LA140

SAMPLE INFORMATION

Sample Name:	Solution 0.06 ug/ mL		
Sample Type:	Standard	Date Acquired:	2/11/2023 12:28:03 PM EET
Vial:	10	Acq. Method Set:	Seria Etil_Cl 7 93 LOD LOQ
Injection #:	1	Date Processed:	2/11/2023 2:48:22 PM EET
Injection Volume:	10.00 ul	Processing Method:	Seria Etil_Cl 7 93 LOD LOQ
Run Time:	30.0 Minutes	Channel Name:	Extract 275
Acquired By:	roman_roxana	Proc. Chnl. Descr.:	2998 PDA 275.0 nm, Smoothed by

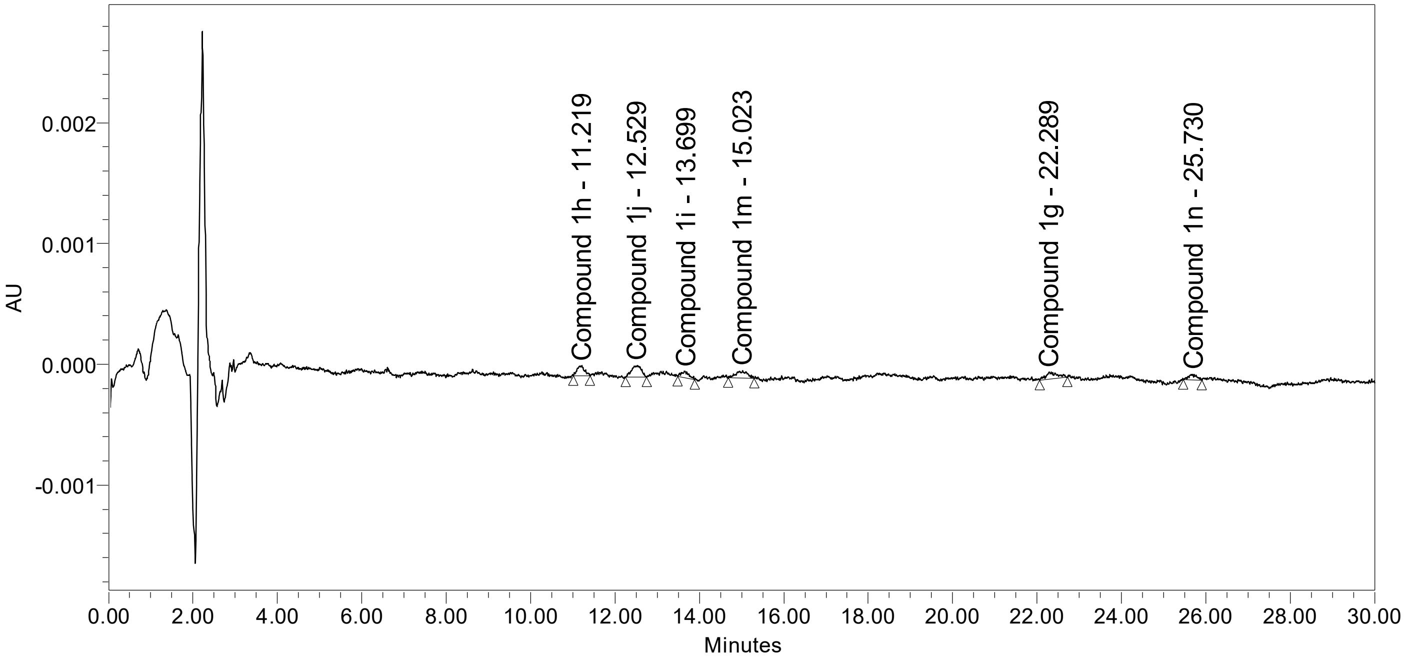

	Peak Name	RT	Area	Height (µV)	Resolution	Level
1	Compound 1h	11.219	1034	83		1.2
2	Compound 1j	12.529	1630	100	3.4	1.2
3	Compound 1i	13.699	658	52	2.7	1.2
4	Compound 1m	15.023	1178	64	2.6	1.2
5	Compound 1g	22.289	1052	57	14.9	1.2
6	Compound 1n	25.730	658	49	9.1	1.2

LOD_LOQ_Cl_Report

Reported by User: Roman Roxana (roman_roxana)
Acquisition Server Waters-cd3
Project Name: Teste\Thiazides
Sample Set Name: Seria Etil_Cl 7 93 LOD LOQ
Code column: Inertsil ODS-3 250*4.6 mm 5um LA140

SAMPLE INFORMATION

Sample Name:	Solution 0.08 ug/ mL		
Sample Type:	Standard	Date Acquired:	2/11/2023 12:59:00 PM EET
Vial:	11	Acq. Method Set:	Seria Etil_Cl 7 93 LOD LOQ
Injection #:	1	Date Processed:	2/11/2023 2:45:01 PM EET
Injection Volume:	10.00 ul	Processing Method:	Seria Etil_Cl 7 93 LOD LOQ
Run Time:	30.0 Minutes	Channel Name:	Extract 275
Acquired By:	roman_roxana	Proc. Chnl. Descr.:	2998 PDA 275.0 nm, Smoothed by

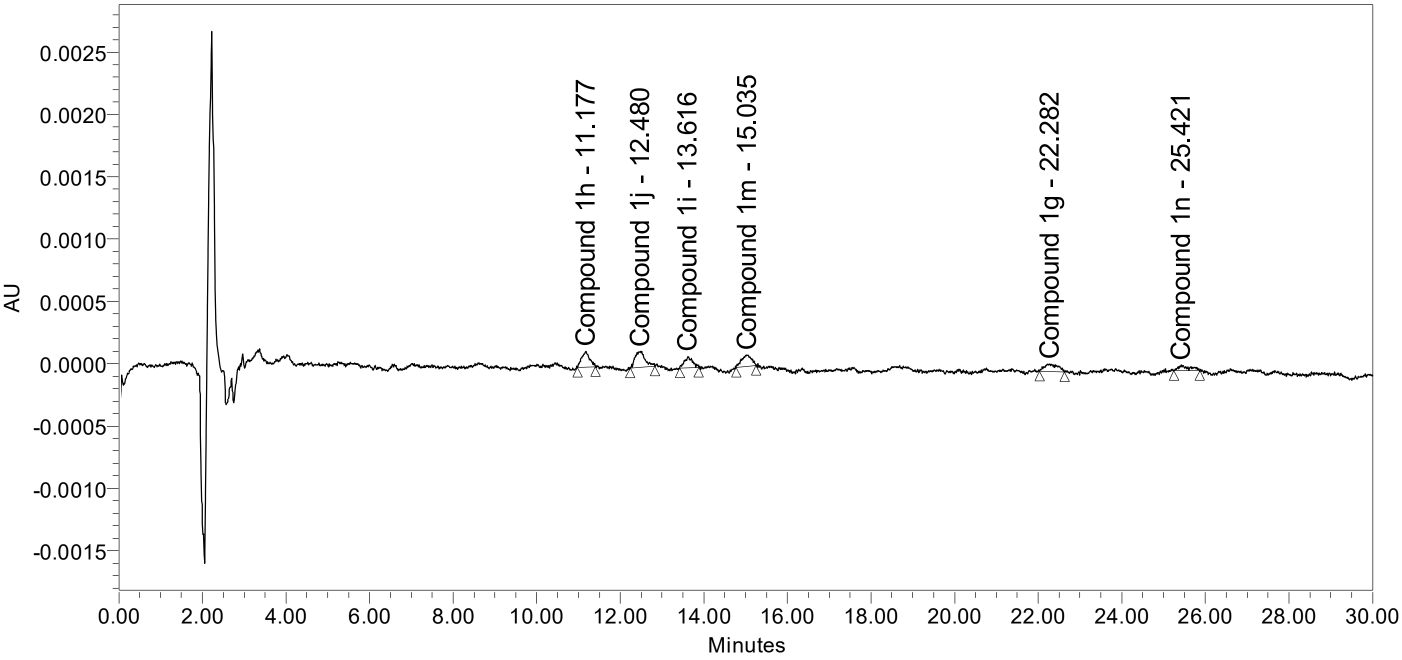

	Peak Name	RT	Area	Height (µV)	Resolution	Level
1	Compound 1h	11.177	1627	126		1.6
2	Compound 1j	12.480	2131	125	3.5	1.6
3	Compound 1i	13.616	1180	91	3.0	1.6
4	Compound 1m	15.035	1459	88	3.4	1.6
5	Compound 1g	22.282	1365	60	11.6	1.6
6	Compound 1n	25.421	872	46	5.6	1.6

LOD_LOQ_Cl_Report

Reported by User: Roman Roxana (roman_roxana)
Acquisition Server Waters-cd3
Project Name: Teste\Thiazides
Sample Set Name: Seria Etil_Cl 7 93 LOD LOQ
Code column: Inertsil ODS-3 250*4.6 mm 5um LA140

SAMPLE INFORMATION

Sample Name:	Solution 0.10 ug/ mL		
Sample Type:	Standard	Date Acquired:	2/11/2023 1:29:40 PM EET
Vial:	12	Acq. Method Set:	Seria Etil_Cl 7 93 LOD LOQ
Injection #:	1	Date Processed:	2/11/2023 3:05:49 PM EET
Injection Volume:	10.00 ul	Processing Method:	Seria Etil_Cl 7 93 LOD LOQ
Run Time:	30.0 Minutes	Channel Name:	Extract 275
Acquired By:	roman_roxana	Proc. Chnl. Descr.:	2998 PDA 275.0 nm, Smoothed by

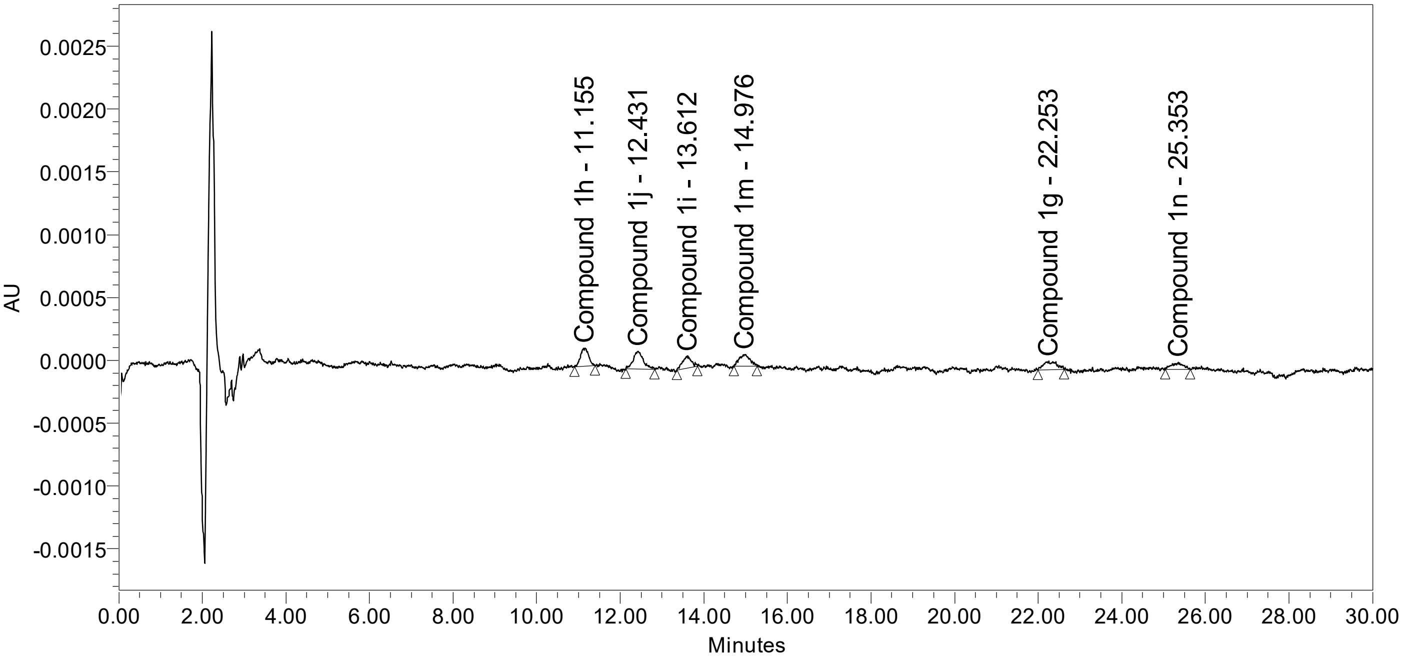

	Peak Name	RT	Area	Height (µV)	Resolution	Level
1	Compound 1h	11.155	1897	144		2
2	Compound 1j	12.431	2500	143	3.3	2
3	Compound 1i	13.612	1374	95	3.0	2
4	Compound 1m	14.976	1682	94	3.1	2
5	Compound 1g	22.253	1438	67	13.8	2
6	Compound 1n	25.353	1130	59	5.8	2

LOD_LOQ_Cl_Report

Reported by User: Roman Roxana (roman_roxana)
Acquisition Server Waters-cd3
Project Name: Teste\Thiazides
Sample Set Name: Seria Etil_Cl 7 93 LOD LOQ
Code column: Inertsil ODS-3 250*4.6 mm 5um LA140

SAMPLE INFORMATION

Sample Name:	Solution 0.20 ug/ mL		
Sample Type:	Standard	Date Acquired:	2/11/2023 2:00:20 PM EET
Vial:	13	Acq. Method Set:	Seria Etil_Cl 7 93 LOD LOQ
Injection #:	1	Date Processed:	2/11/2023 3:11:22 PM EET
Injection Volume:	10.00 ul	Processing Method:	Seria Etil_Cl 7 93 LOD LOQ
Run Time:	30.0 Minutes	Channel Name:	Extract 275
Acquired By:	roman_roxana	Proc. Chnl. Descr.:	2998 PDA 275.0 nm, Smoothed by

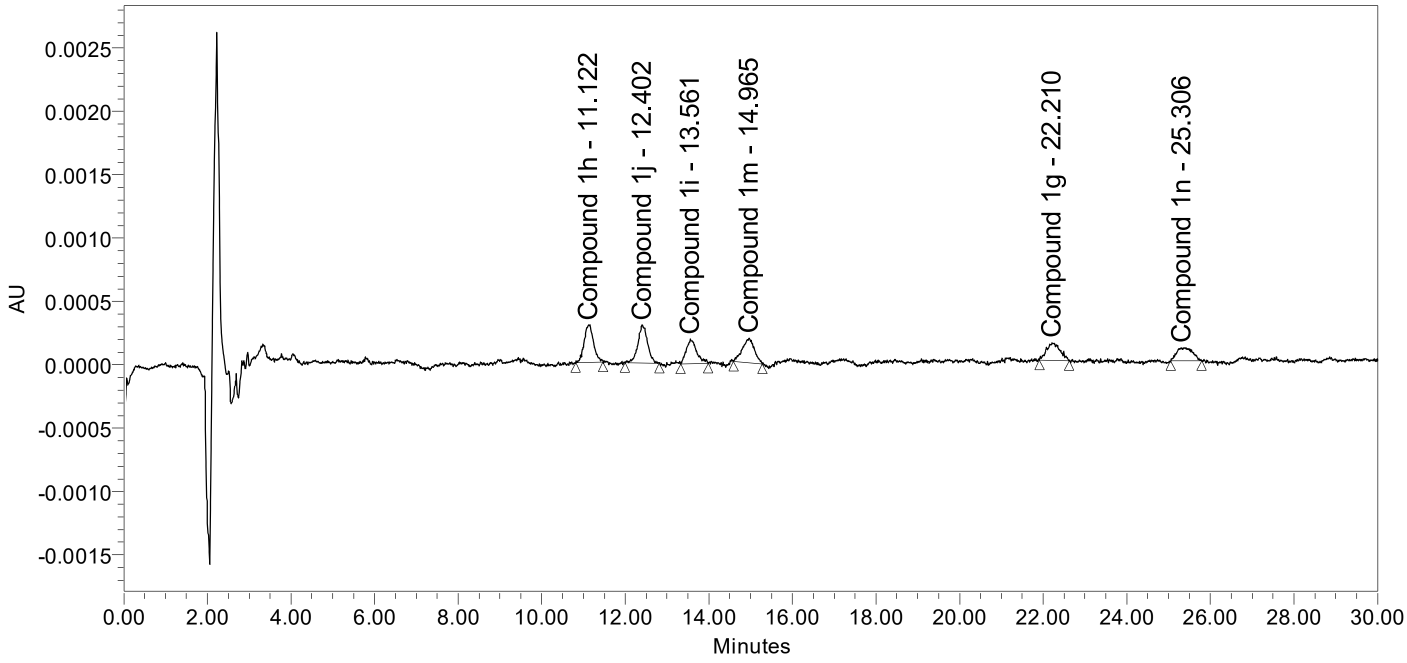

	Peak Name	RT	Area	Height (µV)	Resolution	Level
1	Compound 1h	11.122	4680	297		4
2	Compound 1j	12.402	5007	302	3.1	4
3	Compound 1i	13.561	3217	192	2.8	4
4	Compound 1m	14.965	3871	195	2.9	4
5	Compound 1g	22.210	3157	141	12.3	4
6	Compound 1n	25.306	2748	104	4.4	4

LOD_LOQ_Cl_Report

Reported by User: Roman Roxana (roman_roxana)
Acquisition Server Waters-cd3
Project Name: Teste\Thiazides
Sample Set Name: Seria Etil_Cl 7 93 LOD LOQ
Code column: Inertsil ODS-3 250*4.6 mm 5um LA140

SAMPLE INFORMATION

Sample Name:	Solution 0.50 ug/ mL		
Sample Type:	Standard	Date Acquired:	2/11/2023 11:52:09 AM EET
Vial:	14	Acq. Method Set:	Seria Etil_Cl 7 93 LOD LOQ
Injection #:	1	Date Processed:	2/11/2023 2:31:36 PM EET
Injection Volume:	10.00 ul	Processing Method:	Seria Etil_Cl 7 93 LOD LOQ
Run Time:	40.0 Minutes	Channel Name:	Extract 275
Acquired By:	roman_roxana	Proc. Chnl. Descr.:	2998 PDA 275.0 nm, Smoothed by

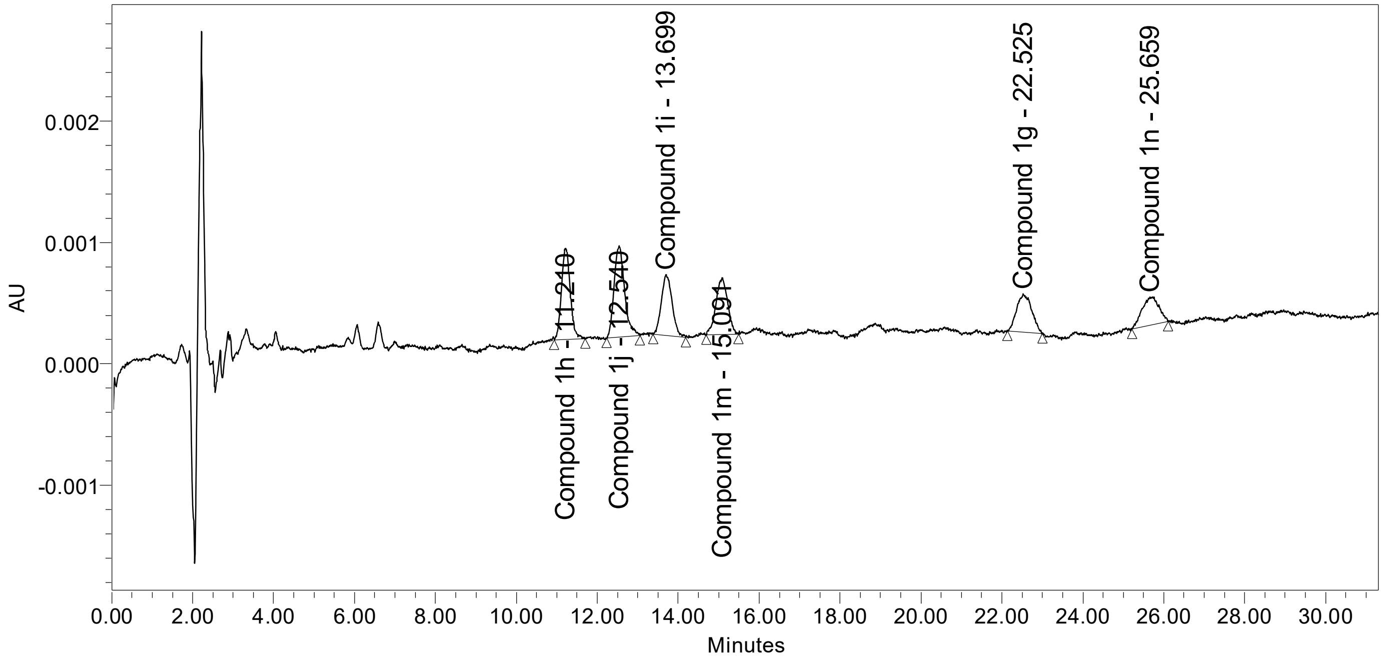

	Peak Name	RT	Area	Height (µV)	Resolution	Level
1	Compound 1h	11.210	11488	754		10
2	Compound 1j	12.540	13394	751	3.1	10
3	Compound 1i	13.699	9172	500	2.5	10
4	Compound 1m	15.091	9300	466	2.8	10
5	Compound 1g	22.525	8104	315	12.0	10
6	Compound 1n	25.659	6692	230	4.0	10

Quantitative determination of

compounds 1g, 1h, 1i, 1j, 1m, 1n

- Validation of the analytical method -

Validation parameters:

- Specificity
- Precision 1
- Precision 2
- LOD – LOQ
- **Linearity**
- **Range**
- Accuracy
- Robustness

Calibration Curve

Reported by User: Roman Roxana (roman_roxana)
Acquisition Server Waters-cd3
Project Name: Teste\Thiazides
Sample Set Name: Seria Etil_Cl 7 93 LOD LOQ
Code column: Inertsil ODS-3, 4,6*250 mm, 5 um

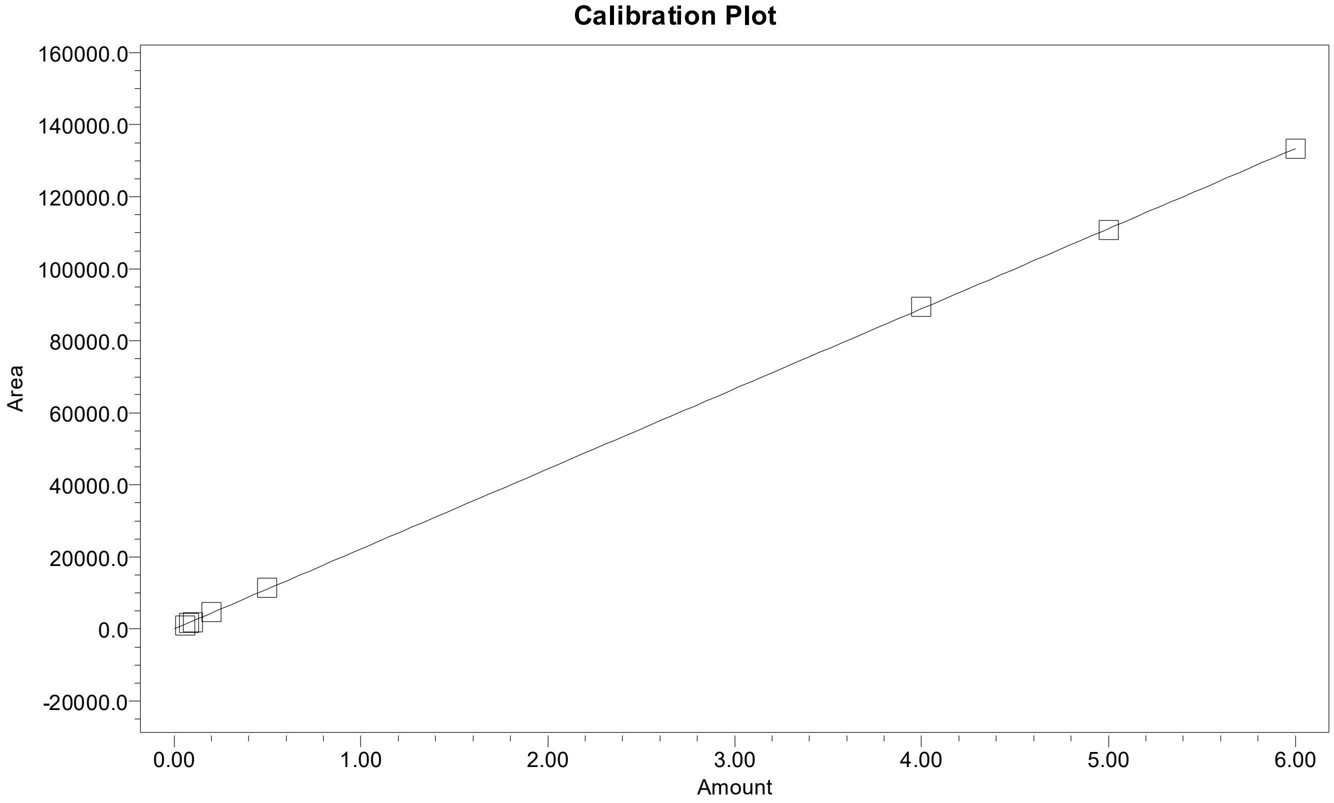

Name: Compound 1h; Processing Method: Seria Etil_Cl 7 93 Linearity; Fit Type: Linear (1st Order); Ca
Curve Id: 3987; A: -2.092550e+001; B: 2.223320e+004; C: 0.000000e+000; D: 0.000000e+000; R^2:
0.999961; Standard Error 3.833989e+002

Calibration Curve

Reported by User: Roman Roxana (roman_roxana)
Acquisition Server Waters-cd3
Project Name: Teste\Thiazides
Sample Set Name: Seria Etil_Cl 7 93 LOD LOQ
Code column: Inertsil ODS-3, 4,6*250 mm, 5 um

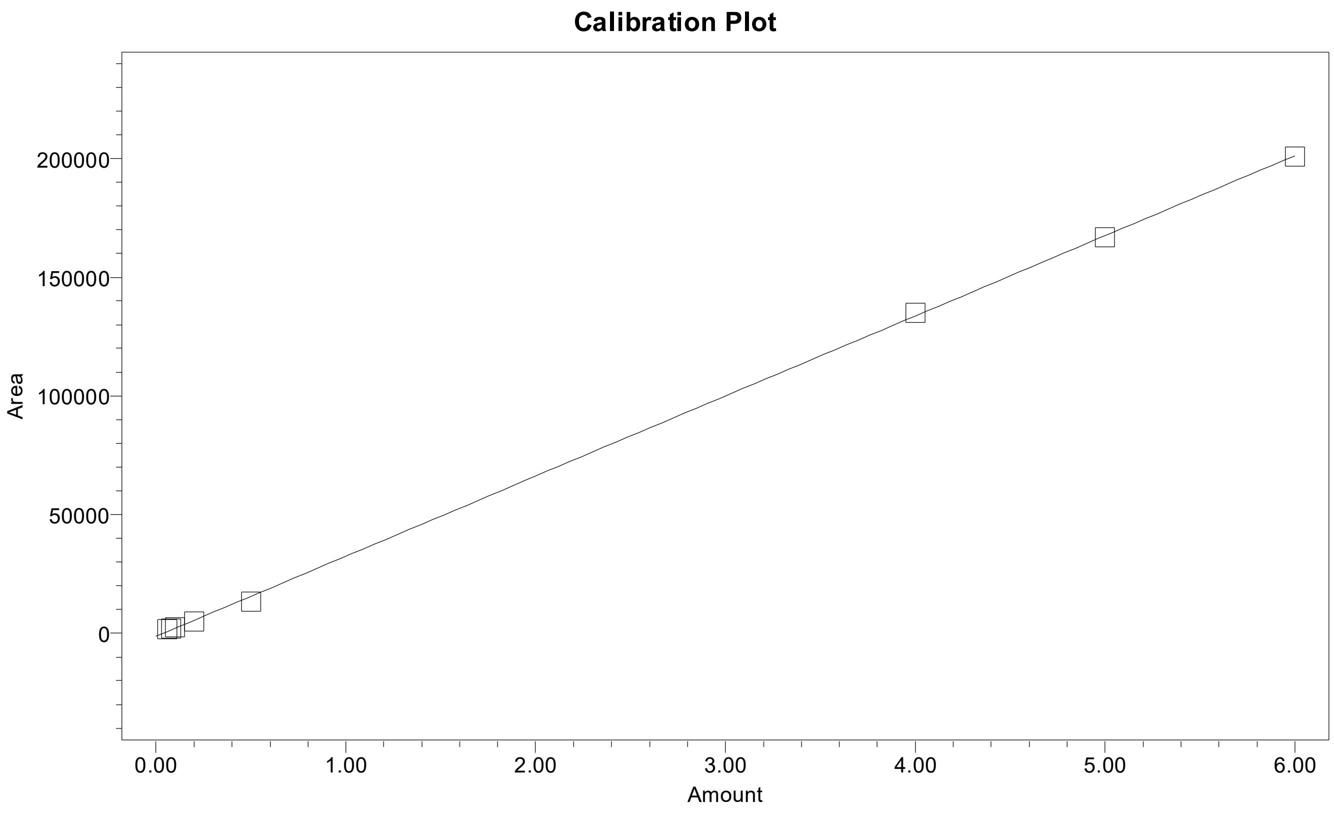

Name: Compound 1j; Processing Method: Seria Etil_Cl 7 93 Linearity; Fit Type: Linear (1st Order); Cal Curve Id: 3988; A: -1.310292e+003; B: 3.375445e+004; C: 0.000000e+000; D: 0.000000e+000; R^2: 0.999836; Standard Error 1.191983e+003

Calibration Curve

Reported by User: Roman Roxana (roman_roxana)
Acquisition Server Waters-cd3
Project Name: Teste\Thiazides
Sample Set Name: Seria Etil_Cl 7 93 LOD LOQ
Code column: Inertsil ODS-3, 4,6*250 mm, 5 um

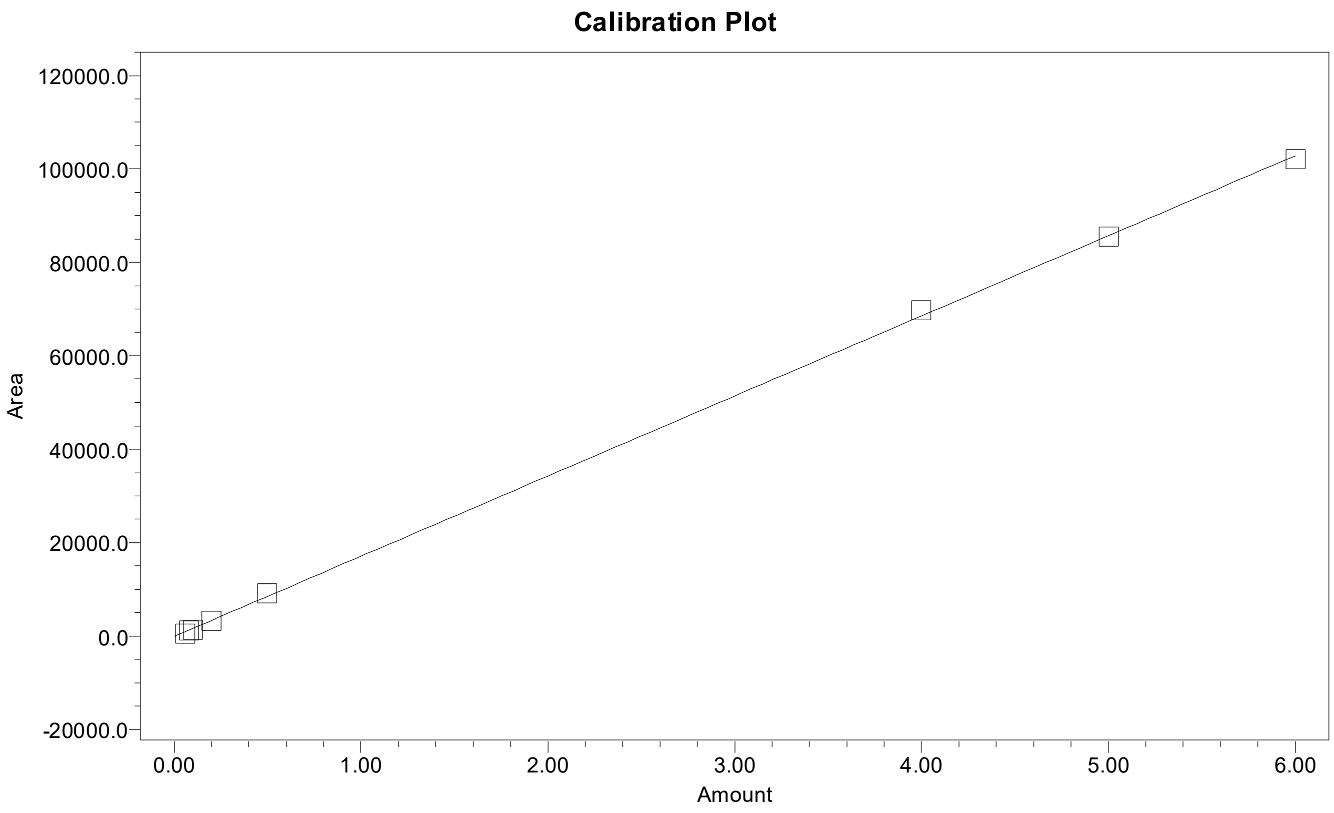

Name: Compound 1i; Processing Method: Seria Etil_Cl 7 93 Linearity; Fit Type: Linear (1st Order); Cal Curve Id: 3989; A: -6.838016e+001; B: 1.715619e+004; C: 0.000000e+000; D: 0.000000e+000; R^2: 0.999797; Standard Error 6.735168e+002

Calibration Curve

Reported by User: Roman Roxana (roman_roxana)
Acquisition Server Waters-cd3
Project Name: Teste\Thiazides
Sample Set Name: Seria Etil_Cl 7 93 LOD LOQ
Code column: Inertsil ODS-3, 4,6*250 mm, 5 um

Calibration Plot

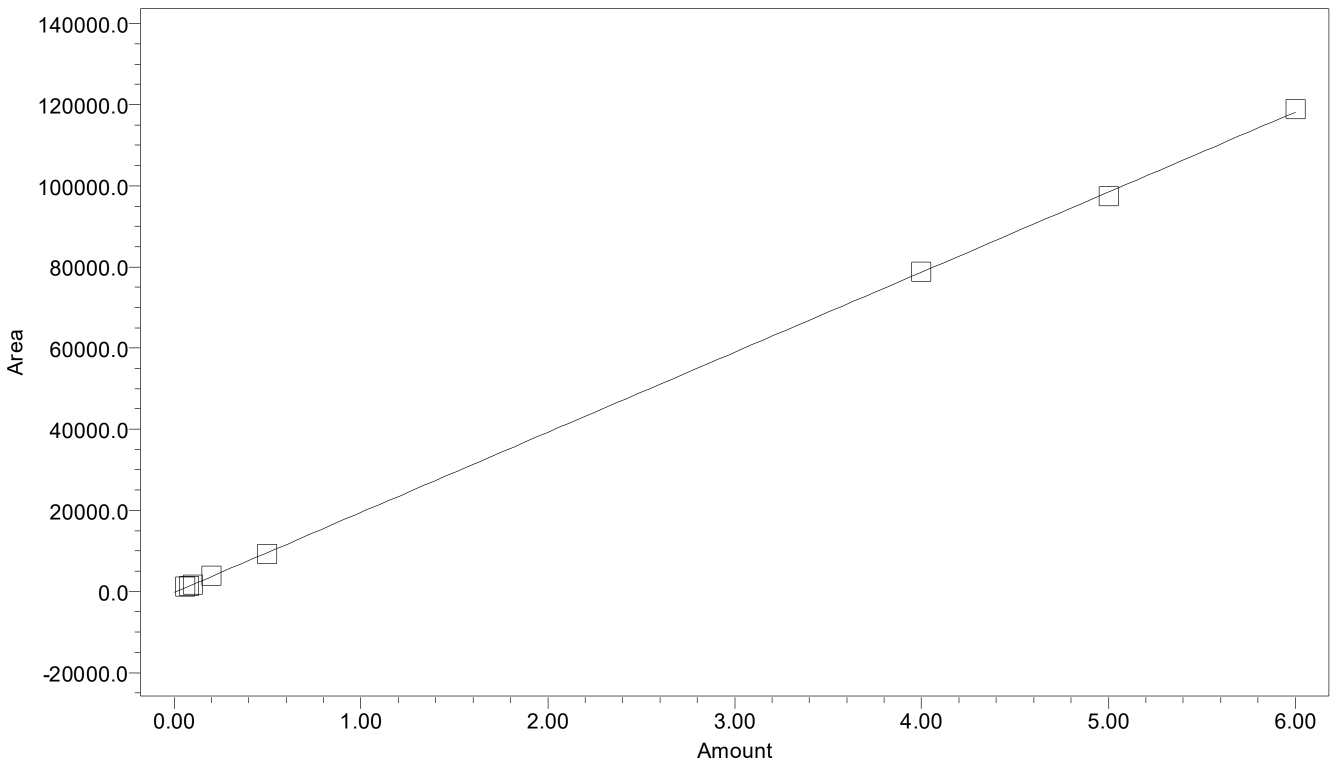

Name: Compound 1m; Processing Method: Seria Etil_Cl 7 93 Linearity; Fit Type: Linear (1st Order);
Cal Curve Id: 3990; A: -2.314274e+002; B: 1.973490e+004; C: 0.000000e+000; D: 0.000000e+000;
R^2: 0.999901; Standard Error 5.413499e+002

Calibration Curve

Reported by User: Roman Roxana (roman_roxana)
Acquisition Server Waters-cd3
Project Name: Teste\Thiazides
Sample Set Name: Seria Etil_Cl 7 93 LOD LOQ
Code column: Inertsil ODS-3, 4,6*250 mm, 5 um

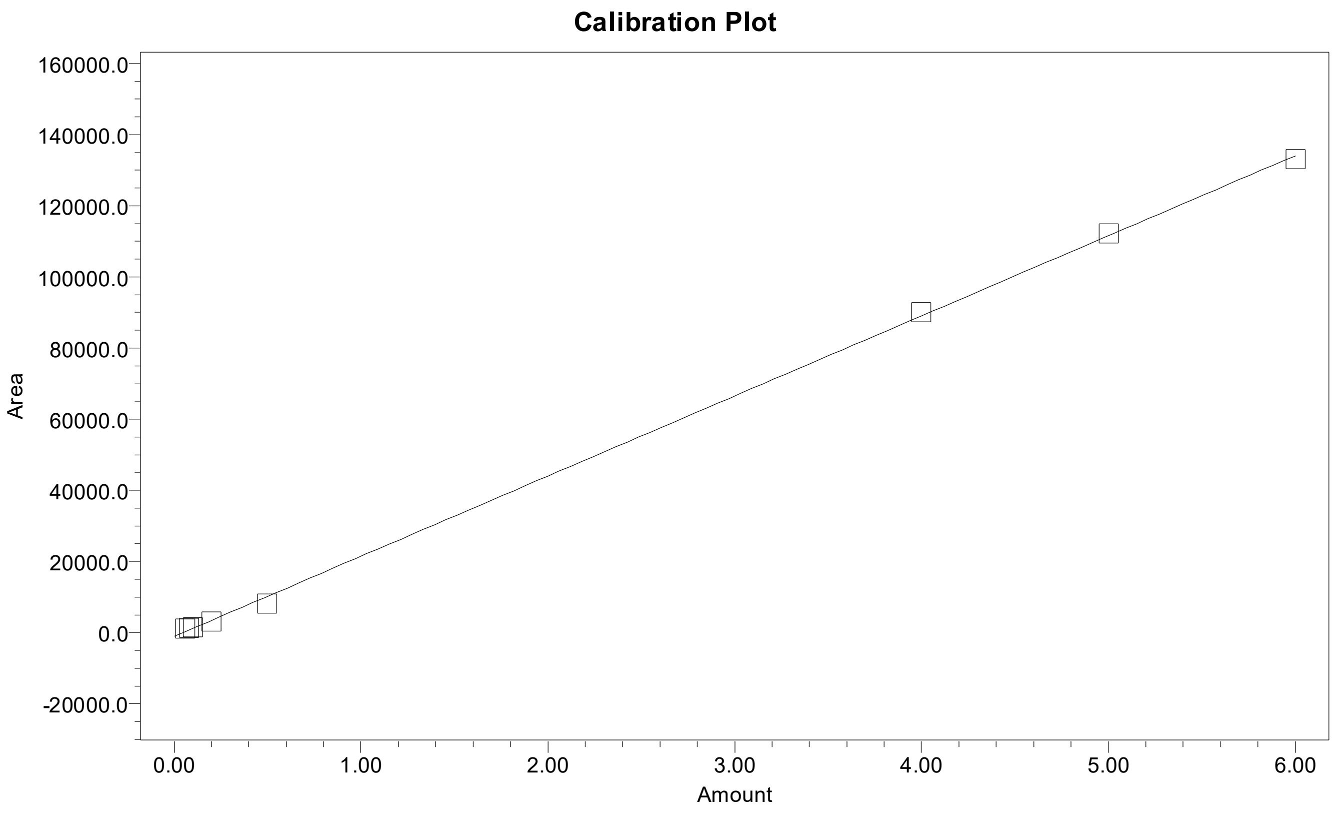

Name: Compound 1g; Processing Method: Seria Etil_Cl 7 93 Linearity; Fit Type: Linear (1st Order); Ca
Curve Id: 3991; A: -1.092978e+003; B: 2.253696e+004; C: 0.000000e+000; D: 0.000000e+000; R^2:
0.999658; Standard Error 1.147850e+003

Calibration Curve

Reported by User: Roman Roxana (roman_roxana)
Acquisition Server Waters-cd3
Project Name: Teste\Thiazides
Sample Set Name: Seria Etil_Cl 7 93 LOD LOQ
Code column: Inertsil ODS-3, 4,6*250 mm, 5 um

Calibration Plot

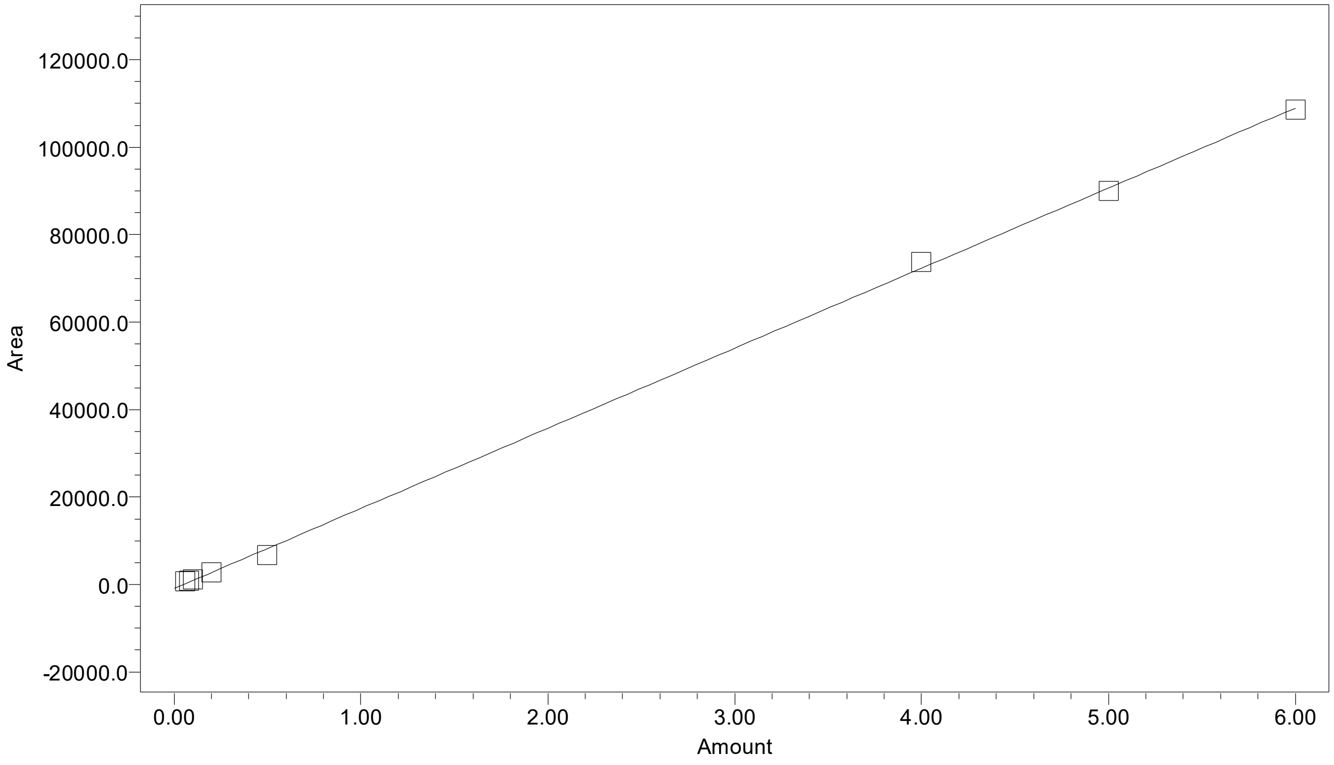

Name: Compound 1n; Processing Method: Seria Etil_Cl 7 93 Linearity; Fit Type: Linear (1st Order); Ca
Curve Id: 3992; A: -9.203579e+002; B: 1.831526e+004; C: 0.000000e+000; D: 0.000000e+000; R^2:
0.999640; Standard Error 9.571321e+002

Calibration Curve

Reported by User: Roman Roxana (roman_roxana)
Acquisition Server Waters-cd3
Project Name: Teste\Thiazides
Sample Set Name: Seria Etil_Cl 7 93 LOD LOQ
Code column: Inertsil ODS-3, 4,6*250 mm, 5 um

Peak Compound 1h

	Name	Level	X Value	Response	Calc. Value	% Deviation	Manual	Ignore
1	Compound 1h	1.2	0.060000	1034.327104	0.047463	-20.895	No	No
2	Compound 1h	1.6	0.080000	1626.691286	0.074106	-7.367	No	No
3	Compound 1h	2	0.100000	1896.802622	0.086255	-13.745	No	No
4	Compound 1h	4	0.200000	4679.969503	0.211436	5.718	No	No
5	Compound 1h	10	0.500000	11488.238406	0.517657	3.531	No	No
6	Compound 1h	80	4.000000	89435.928781	4.023572	0.589	No	No
7	Compound 1h	100	5.000000	110697.564728	4.979873	-0.403	No	No
8	Compound 1h	120	6.000000	133370.214705	5.999639	-0.006	No	No

Peak Compound 1j

	Name	Level	X Value	Response	Calc. Value	% Deviation	Manual	Ignore
1	Compound 1j	1.2	0.060000	1630.336477	0.087118	45.197	No	No
2	Compound 1j	1.6	0.080000	2133.223118	0.102017	27.521	No	No
3	Compound 1j	2	0.100000	2500.255530	0.112890	12.890	No	No
4	Compound 1j	4	0.200000	5006.601112	0.187143	-6.429	No	No
5	Compound 1j	10	0.500000	13394.404392	0.435637	-12.873	No	No
6	Compound 1j	80	4.000000	135006.550565	4.038485	0.962	No	No
7	Compound 1j	100	5.000000	166926.181411	4.984127	-0.317	No	No
8	Compound 1j	120	6.000000	200966.107959	5.992584	-0.124	No	No

Calibration Curve

Reported by User: Roman Roxana (roman_roxana)
Acquisition Server Waters-cd3
Project Name: Teste\Thiazides
Sample Set Name: Seria Etil_Cl 7 93 LOD LOQ
Code column: Inertsil ODS-3, 4,6*250 mm, 5 um

Peak Compound 1i

	Name	Level	X Value	Response	Calc. Value	% Deviation	Manual	Ignore
1	Compound 1i	1.2	0.060000	507.119752	0.033545	-44.092	No	No
2	Compound 1i	1.6	0.080000	1179.678589	0.072747	-9.066	No	No
3	Compound 1i	2	0.100000	1374.135930	0.084081	-15.919	No	No
4	Compound 1i	4	0.200000	3216.945031	0.191495	-4.252	No	No
5	Compound 1i	10	0.500000	9171.385143	0.538567	7.713	No	No
6	Compound 1i	80	4.000000	69751.545178	4.069664	1.742	No	No
7	Compound 1i	100	5.000000	85573.309645	4.991883	-0.162	No	No
8	Compound 1i	120	6.000000	102148.503946	5.958018	-0.700	No	No

Peak Compound 1m

	Name	Level	X Value	Response	Calc. Value	% Deviation	Manual	Ignore
1	Compound 1m	1.2	0.060000	1178.133697	0.071425	19.041	No	No
2	Compound 1m	1.6	0.080000	1459.015714	0.085658	7.072	No	No
3	Compound 1m	2	0.100000	1681.569370	0.096935	-3.065	No	No
4	Compound 1m	4	0.200000	3871.449351	0.207899	3.950	No	No
5	Compound 1m	10	0.500000	9300.465892	0.482997	-3.401	No	No
6	Compound 1m	80	4.000000	78850.929572	4.007233	0.181	No	No
7	Compound 1m	100	5.000000	97455.666410	4.949965	-1.001	No	No
8	Compound 1m	120	6.000000	118925.732651	6.037889	0.631	No	No

Calibration Curve

Reported by User: Roman Roxana (roman_roxana)
Acquisition Server Waters-cd3
Project Name: Teste\Thiazides
Sample Set Name: Seria Etil_Cl 7 93 LOD LOQ
Code column: Inertsil ODS-3, 4,6*250 mm, 5 um

Peak Compound 1g

	Name	Level	X Value	Response	Calc. Value	% Deviation	Manual	Ignore
1	Compound 1g	1.2	0.060000	1053.315079	0.095234	58.724	No	No
2	Compound 1g	1.6	0.080000	1365.145509	0.109071	36.338	No	No
3	Compound 1g	2	0.100000	1438.295635	0.112317	12.317	No	No
4	Compound 1g	4	0.200000	3157.245813	0.188589	-5.705	No	No
5	Compound 1g	10	0.500000	8106.260151	0.408184	-18.363	No	No
6	Compound 1g	80	4.000000	90082.883769	4.045615	1.140	No	No
7	Compound 1g	100	5.000000	112175.124170	5.025882	0.518	No	No
8	Compound 1g	120	6.000000	133117.079209	5.955109	-0.748	No	No

Peak Compound 1n

	Name	Level	X Value	Response	Calc. Value	% Deviation	Manual	Ignore
1	Compound 1n	1.2	0.060000	657.858954	0.086170	43.616	No	No
2	Compound 1n	1.6	0.080000	872.309723	0.097878	22.348	No	No
3	Compound 1n	2	0.100000	1130.181323	0.111958	11.958	No	No
4	Compound 1n	4	0.200000	2752.719131	0.200547	0.274	No	No
5	Compound 1n	10	0.500000	6691.625978	0.415609	-16.878	No	No
6	Compound 1n	80	4.000000	73828.753068	4.081248	2.031	No	No
7	Compound 1n	100	5.000000	90044.927543	4.966640	-0.667	No	No
8	Compound 1n	120	6.000000	108603.946820	5.979949	-0.334	No	No

Linearity_CI_Report

Reported by User: Roman Roxana (roman_roxana)
Acquisition Server Waters-cd3
Project Name: Teste\Thiazides
Sample Set Name: Seria Etil_Cl 7 93 LOD LOQ
Code column: Inertsil ODS-3 250*4.6 mm 5um LA140

SAMPLE INFORMATION

Sample Name:	Solution 0.06 ug/ mL		
Sample Type:	Standard	Date Acquired:	2/11/2023 12:28:03 PM EET
Vial:	10	Acq. Method Set:	Seria Etil_Cl 7 93 LOD LOQ
Injection #:	1	Date Processed:	2/13/2023 6:46:03 PM EET
Injection Volume:	10.00 ul	Processing Method:	Seria Etil_Cl 7 93 Linearity
Run Time:	30.0 Minutes	Channel Name:	Extract 275
Acquired By:	roman_roxana	Proc. Chnl. Descr.:	2998 PDA 275.0 nm, Smoothed by

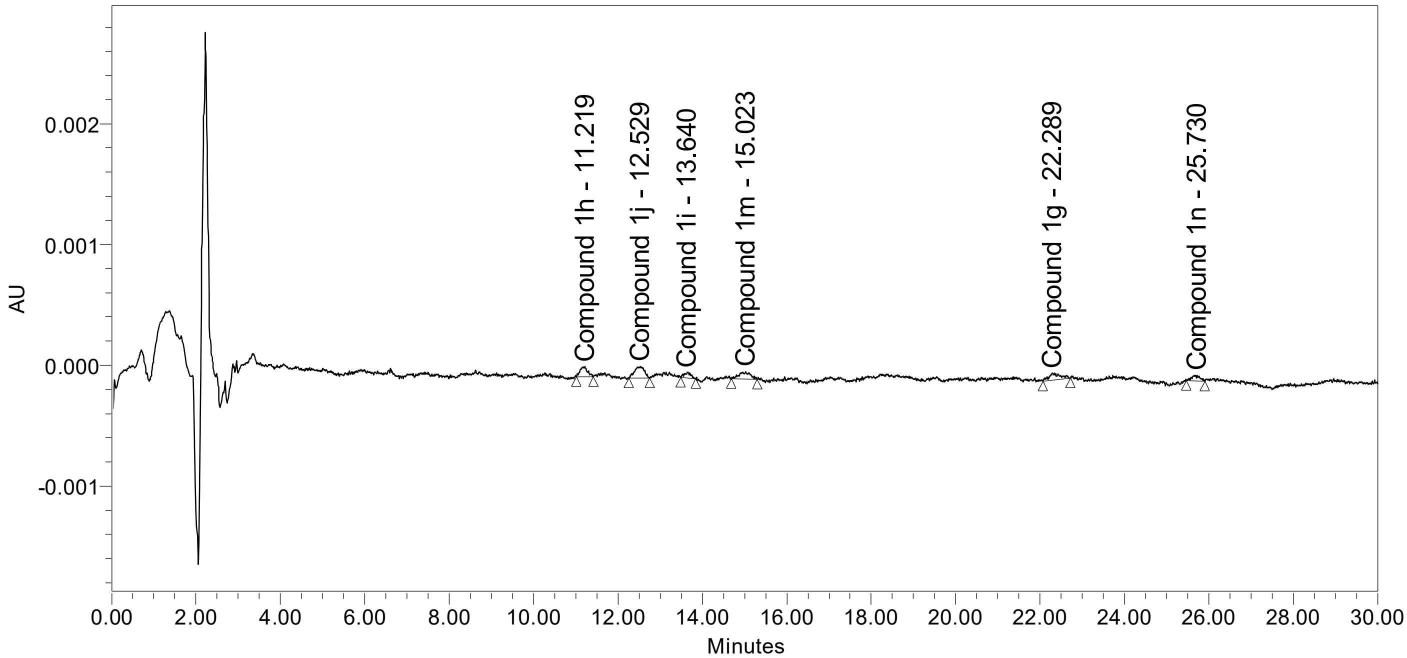

	Peak Name	RT	Area	Height (µV)	Resolution	Level
1	Compound 1h	11.219	1034	83		1.2
2	Compound 1j	12.529	1630	100	3.45	1.2
3	Compound 1i	13.640	507	46	2.60	1.2
4	Compound 1m	15.023	1178	64	2.78	1.2
5	Compound 1g	22.289	1053	57	14.89	1.2
6	Compound 1n	25.730	658	49	9.14	1.2

Linearity_CI_Report

Reported by User: Roman Roxana (roman_roxana)
Acquisition Server Waters-cd3
Project Name: Teste\Thiazides
Sample Set Name: Seria Etil_Cl 7 93 LOD LOQ
Code column: Inertsil ODS-3 250*4.6 mm 5um LA140

SAMPLE INFORMATION

Sample Name:	Solution 0.08 ug/ mL		
Sample Type:	Standard	Date Acquired:	2/11/2023 12:59:00 PM EET
Vial:	11	Acq. Method Set:	Seria Etil_Cl 7 93 LOD LOQ
Injection #:	1	Date Processed:	2/13/2023 6:55:17 PM EET
Injection Volume:	10.00 ul	Processing Method:	Seria Etil_Cl 7 93 Linearity
Run Time:	30.0 Minutes	Channel Name:	Extract 275
Acquired By:	roman_roxana	Proc. Chnl. Descr.:	2998 PDA 275.0 nm, Smoothed by

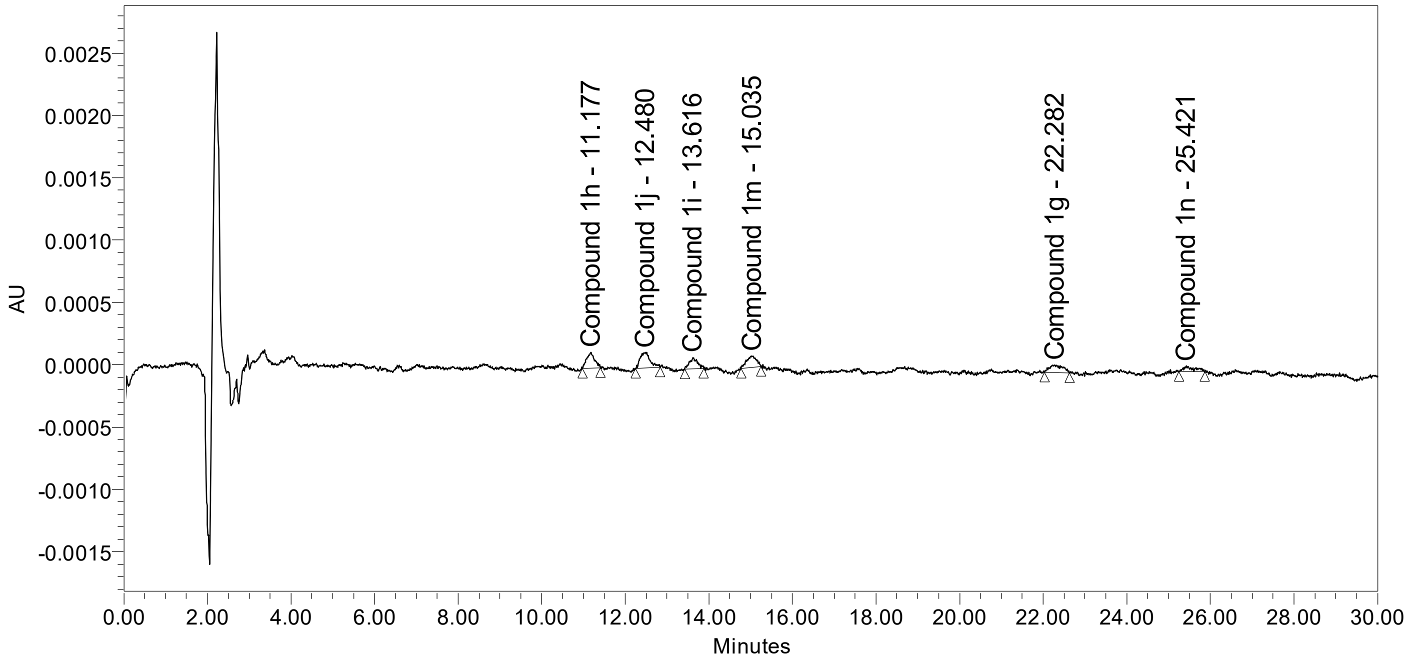

	Peak Name	RT	Area	Height (µV)	Resolution	Level
1	Compound 1h	11.177	1627	126		1.6
2	Compound 1j	12.480	2133	125	3.46	1.6
3	Compound 1i	13.616	1180	91	3.02	1.6
4	Compound 1m	15.035	1459	88	3.41	1.6
5	Compound 1g	22.282	1365	60	11.63	1.6
6	Compound 1n	25.421	872	46	5.61	1.6

Linearity_Cl_Report

Reported by User: Roman Roxana (roman_roxana)
Acquisition Server Waters-cd3
Project Name: Teste\Thiazides
Sample Set Name: Seria Etil_Cl 7 93 LOD LOQ
Code column: Inertsil ODS-3 250*4.6 mm 5um LA140

SAMPLE INFORMATION

Sample Name:	Solution 0.10 ug/ mL		
Sample Type:	Standard	Date Acquired:	2/11/2023 1:29:40 PM EET
Vial:	12	Acq. Method Set:	Seria Etil_Cl 7 93 LOD LOQ
Injection #:	1	Date Processed:	2/13/2023 7:00:25 PM EET
Injection Volume:	10.00 ul	Processing Method:	Seria Etil_Cl 7 93 Linearity
Run Time:	30.0 Minutes	Channel Name:	Extract 275
Acquired By:	roman_roxana	Proc. Chnl. Descr.:	2998 PDA 275.0 nm, Smoothed by

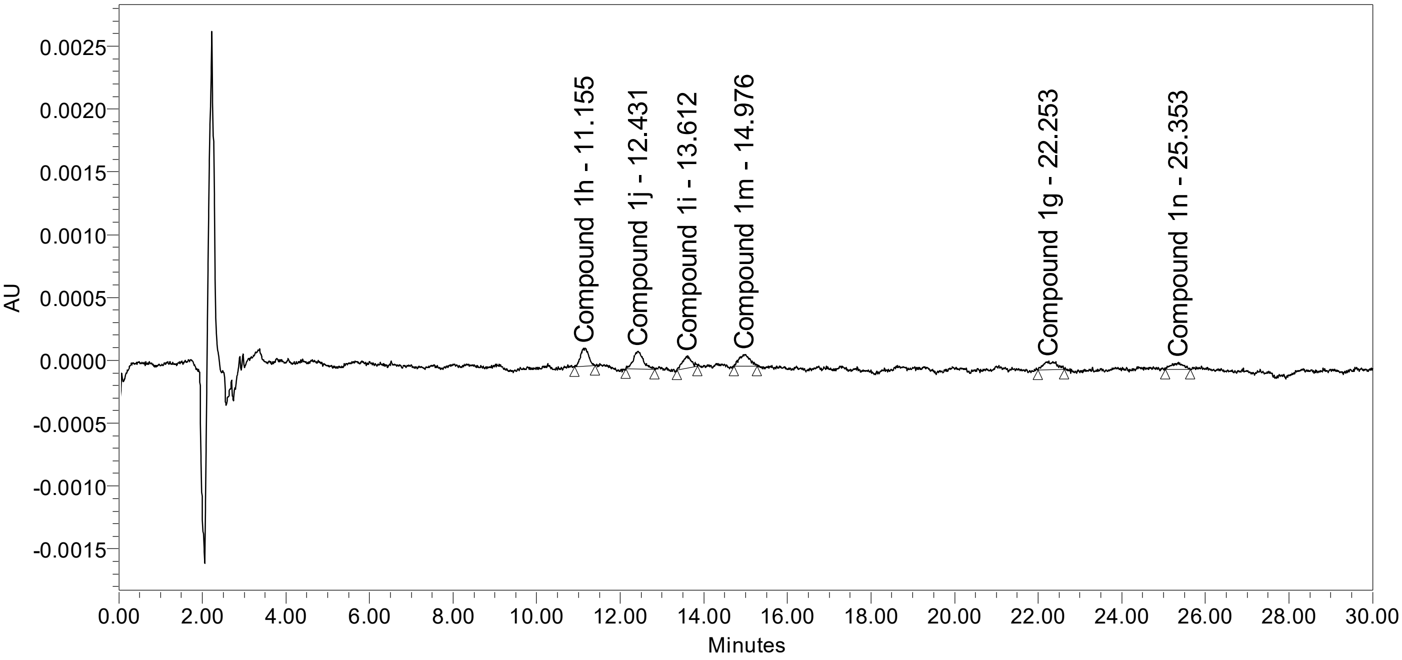

	Peak Name	RT	Area	Height (µV)	Resolution	Level
1	Compound 1h	11.155	1897	144		2
2	Compound 1j	12.431	2500	143	3.29	2
3	Compound 1i	13.612	1374	95	2.95	2
4	Compound 1m	14.976	1682	94	3.11	2
5	Compound 1g	22.253	1438	67	13.75	2
6	Compound 1n	25.353	1130	59	5.75	2

Linearity_Cl_Report

Reported by User: Roman Roxana (roman_roxana)
Acquisition Server Waters-cd3
Project Name: Teste\Thiazides
Sample Set Name: Seria Etil_Cl 7 93 LOD LOQ
Code column: Inertsil ODS-3 250*4.6 mm 5um LA140

SAMPLE INFORMATION

Sample Name:	Solution 0.20 ug/ mL		
Sample Type:	Standard	Date Acquired:	2/11/2023 2:00:20 PM EET
Vial:	13	Acq. Method Set:	Seria Etil_Cl 7 93 LOD LOQ
Injection #:	1	Date Processed:	2/13/2023 7:13:36 PM EET
Injection Volume:	10.00 ul	Processing Method:	Seria Etil_Cl 7 93 Linearity
Run Time:	30.0 Minutes	Channel Name:	Extract 275
Acquired By:	roman_roxana	Proc. Chnl. Descr.:	2998 PDA 275.0 nm, Smoothed by

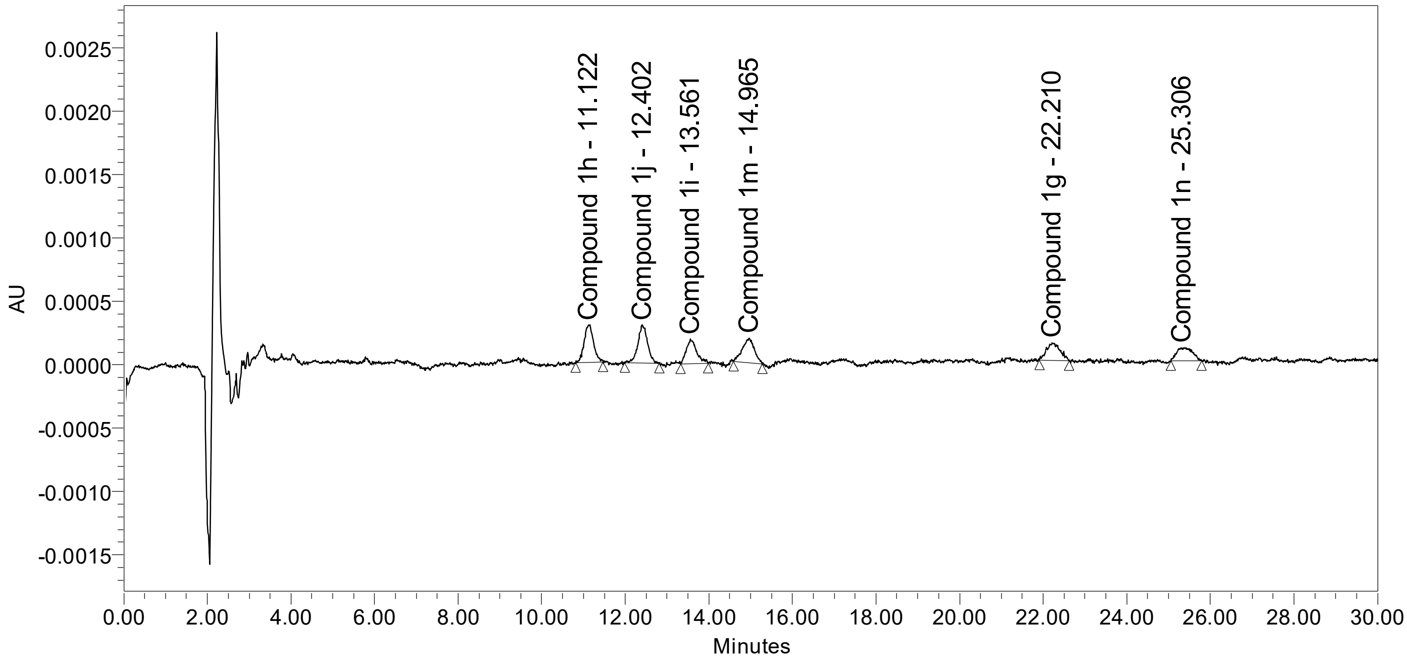

	Peak Name	RT	Area	Height (µV)	Resolution	Level
1	Compound 1h	11.122	4680	297		4
2	Compound 1j	12.402	5007	302	3.13	4
3	Compound 1i	13.561	3217	192	2.76	4
4	Compound 1m	14.965	3871	195	2.91	4
5	Compound 1g	22.210	3157	141	12.26	4
6	Compound 1n	25.306	2753	103	4.42	4

Linearity_Cl_Report

Reported by User: Roman Roxana (roman_roxana)
Acquisition Server Waters-cd3
Project Name: Teste\Thiazides
Sample Set Name: Seria Etil_Cl 7 93 LOD LOQ
Code column: Inertsil ODS-3 250*4.6 mm 5um LA140

SAMPLE INFORMATION

Sample Name:	Solution 0.50 ug/ mL		
Sample Type:	Standard	Date Acquired:	2/11/2023 11:52:09 AM EET
Vial:	14	Acq. Method Set:	Seria Etil_Cl 7 93 LOD LOQ
Injection #:	1	Date Processed:	2/13/2023 7:21:05 PM EET
Injection Volume:	10.00 ul	Processing Method:	Seria Etil_Cl 7 93 Linearity
Run Time:	40.0 Minutes	Channel Name:	Extract 275
Acquired By:	roman_roxana	Proc. Chnl. Descr.:	2998 PDA 275.0 nm, Smoothed by

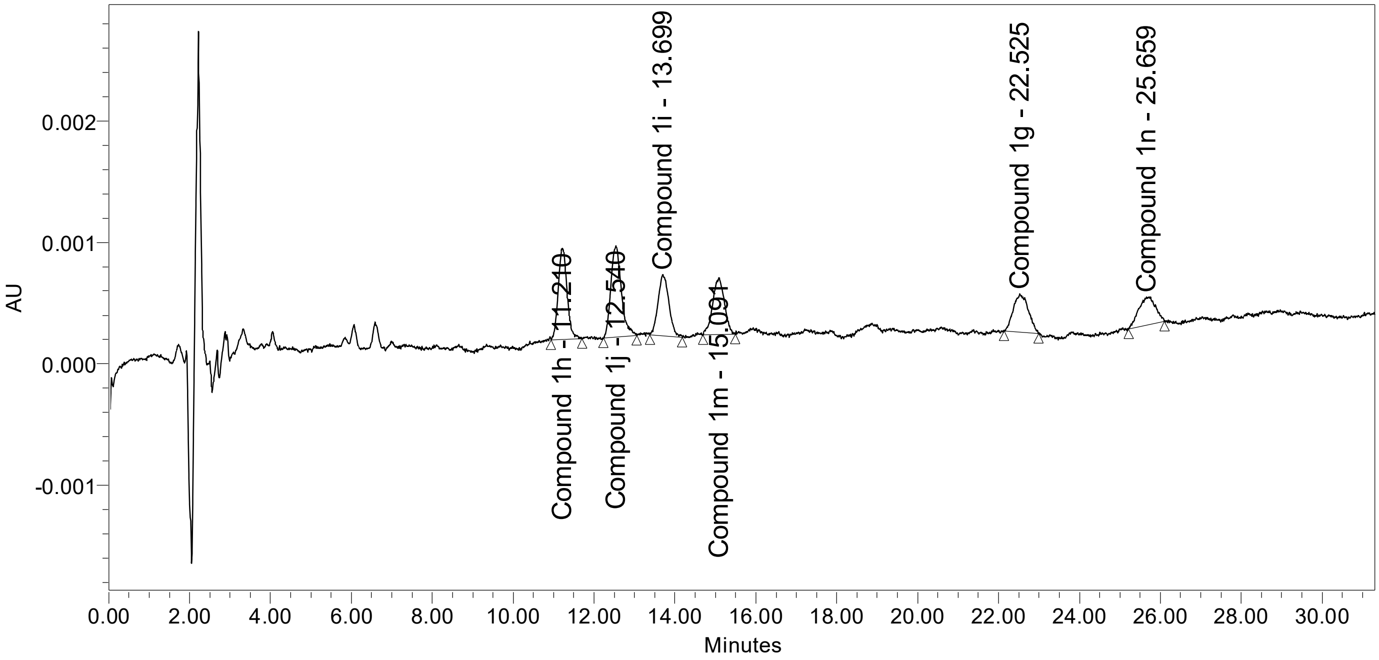

	Peak Name	RT	Area	Height (µV)	Resolution	Level
1	Compound 1h	11.210	11488	754		10
2	Compound 1j	12.540	13394	751	3.12	10
3	Compound 1i	13.699	9171	500	2.46	10
4	Compound 1m	15.091	9300	466	2.75	10
5	Compound 1g	22.525	8106	315	11.98	10
6	Compound 1n	25.659	6692	230	4.01	10

Linearity_CI_Report

Reported by User: Roman Roxana (roman_roxana)
Acquisition Server Waters-cd3
Project Name: Teste\Thiazides
Sample Set Name: Seria Etil_Cl 7 93 Linearity
Code column: Inertsil ODS-3 250*4.6 mm 5um LA140

SAMPLE INFORMATION

Sample Name:	Solution 4.0 ug/ mL		
Sample Type:	Standard	Date Acquired:	2/11/2023 3:30:17 PM EET
Vial:	25	Acq. Method Set:	Seria Etil_Cl 7 93 Linearity
Injection #:	1	Date Processed:	2/13/2023 7:23:22 PM EET
Injection Volume:	10.00 ul	Processing Method:	Seria Etil_Cl 7 93 Linearity
Run Time:	30.0 Minutes	Channel Name:	Extract 275
Acquired By:	roman_roxana	Proc. Chnl. Descr.:	2998 PDA 275.0 nm, Smoothed by

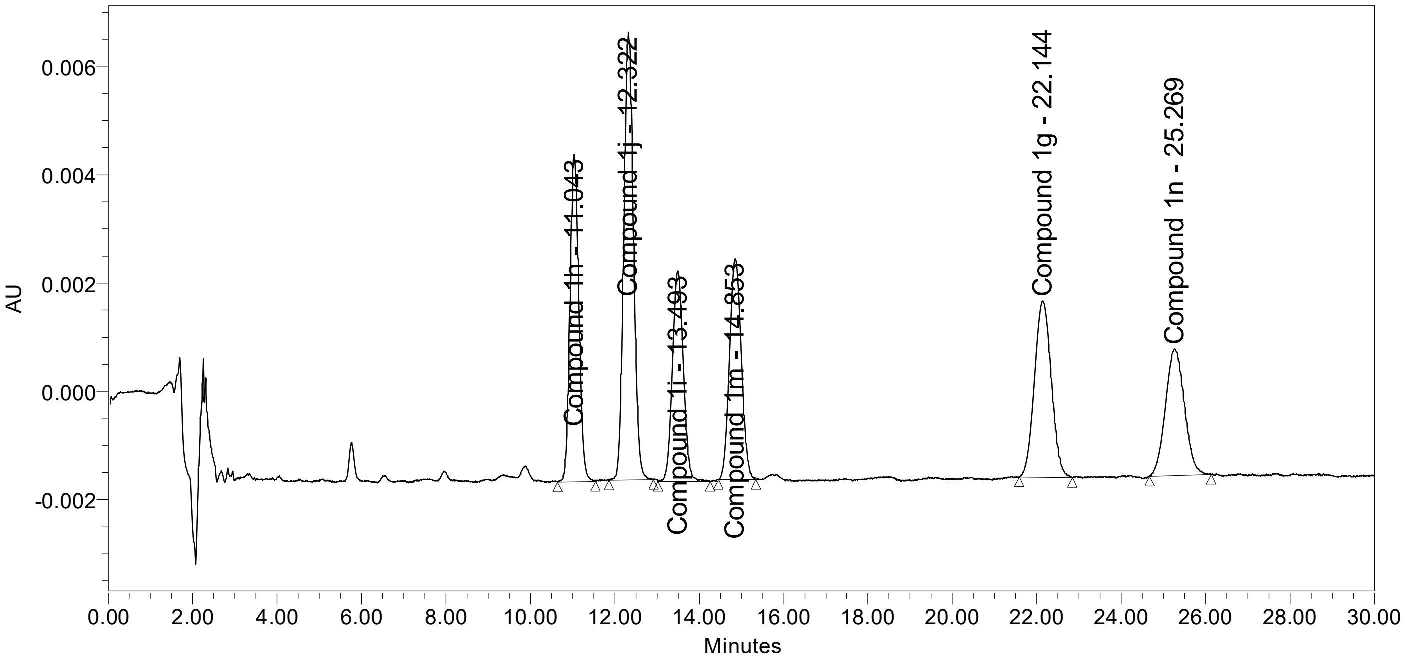

	Peak Name	RT	Area	Height (µV)	Resolution	Level
1	Compound 1h	11.043	89436	6043		80
2	Compound 1j	12.322	135007	8269	3.13	80
3	Compound 1i	13.493	69752	3880	2.60	80
4	Compound 1m	14.853	78851	4080	2.76	80
5	Compound 1g	22.144	90083	3249	11.65	80
6	Compound 1n	25.269	73829	2333	3.98	80

Linearity_Cl_Report

Reported by User: Roman Roxana (roman_roxana)
Acquisition Server Waters-cd3
Project Name: Teste\Thiazides
Sample Set Name: Seria Etil_Cl 7 93 Linearity
Code column: Inertsil ODS-3 250*4.6 mm 5um LA140

SAMPLE INFORMATION

Sample Name:	Solution 5.0 ug/ mL		
Sample Type:	Standard	Date Acquired:	2/11/2023 4:01:13 PM EET
Vial:	26	Acq. Method Set:	Seria Etil_Cl 7 93 Linearity
Injection #:	1	Date Processed:	2/13/2023 7:25:37 PM EET
Injection Volume:	10.00 ul	Processing Method:	Seria Etil_Cl 7 93 Linearity
Run Time:	30.0 Minutes	Channel Name:	Extract 275
Acquired By:	roman_roxana	Proc. Chnl. Descr.:	2998 PDA 275.0 nm, Smoothed by

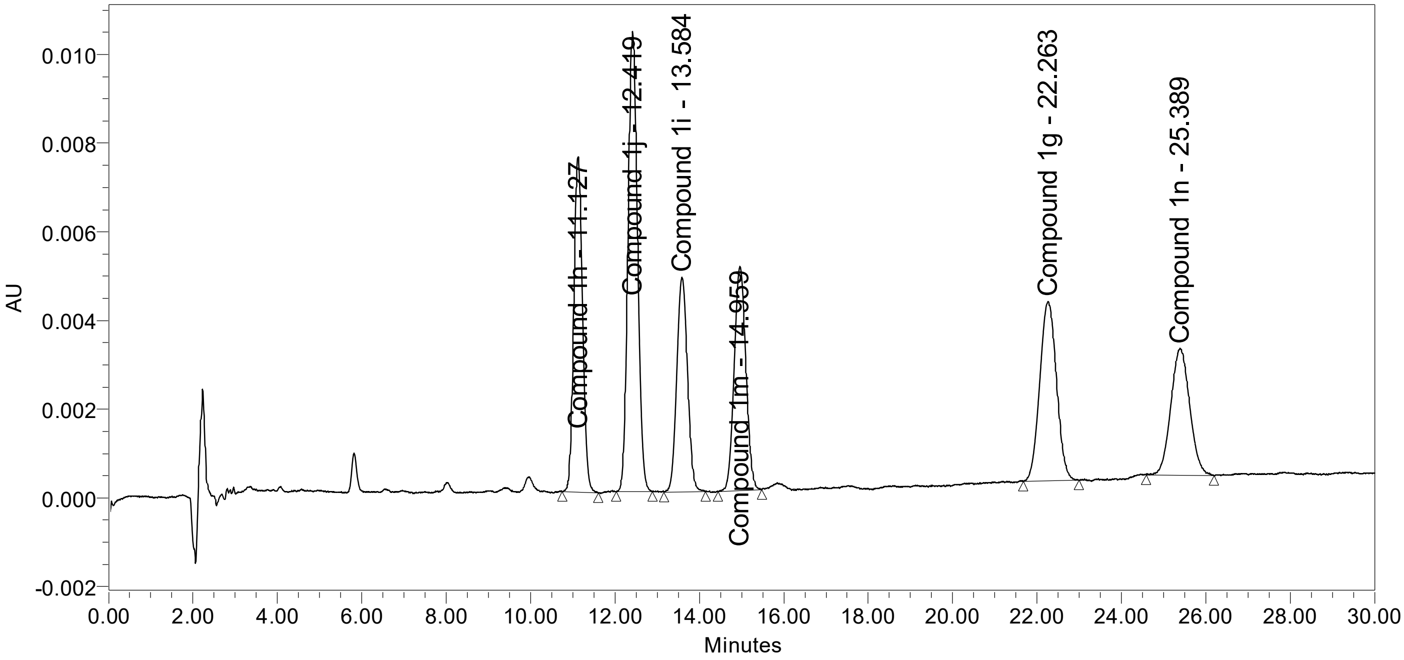

	Peak Name	RT	Area	Height (µV)	Resolution	Level
1	Compound 1h	11.127	110698	7565		100
2	Compound 1j	12.419	166926	10382	3.17	100
3	Compound 1i	13.584	85573	4842	2.61	100
4	Compound 1m	14.959	97456	5062	2.81	100
5	Compound 1g	22.263	112175	4052	11.70	100
6	Compound 1n	25.389	90045	2855	3.96	100

Linearity_Cl_Report

Reported by User: Roman Roxana (roman_roxana)
Acquisition Server Waters-cd3
Project Name: Teste\Thiazides
Sample Set Name: Seria Etil_Cl 7 93 Linearity
Code column: Inertsil ODS-3 250*4.6 mm 5um LA140

SAMPLE INFORMATION

Sample Name:	Solution 6.0 ug/ mL		
Sample Type:	Standard	Date Acquired:	2/11/2023 4:32:08 PM EET
Vial:	27	Acq. Method Set:	Seria Etil_Cl 7 93 Linearity
Injection #:	1	Date Processed:	2/13/2023 7:27:26 PM EET
Injection Volume:	10.00 ul	Processing Method:	Seria Etil_Cl 7 93 Linearity
Run Time:	30.0 Minutes	Channel Name:	Extract 275
Acquired By:	roman_roxana	Proc. Chnl. Descr.:	2998 PDA 275.0 nm, Smoothed by

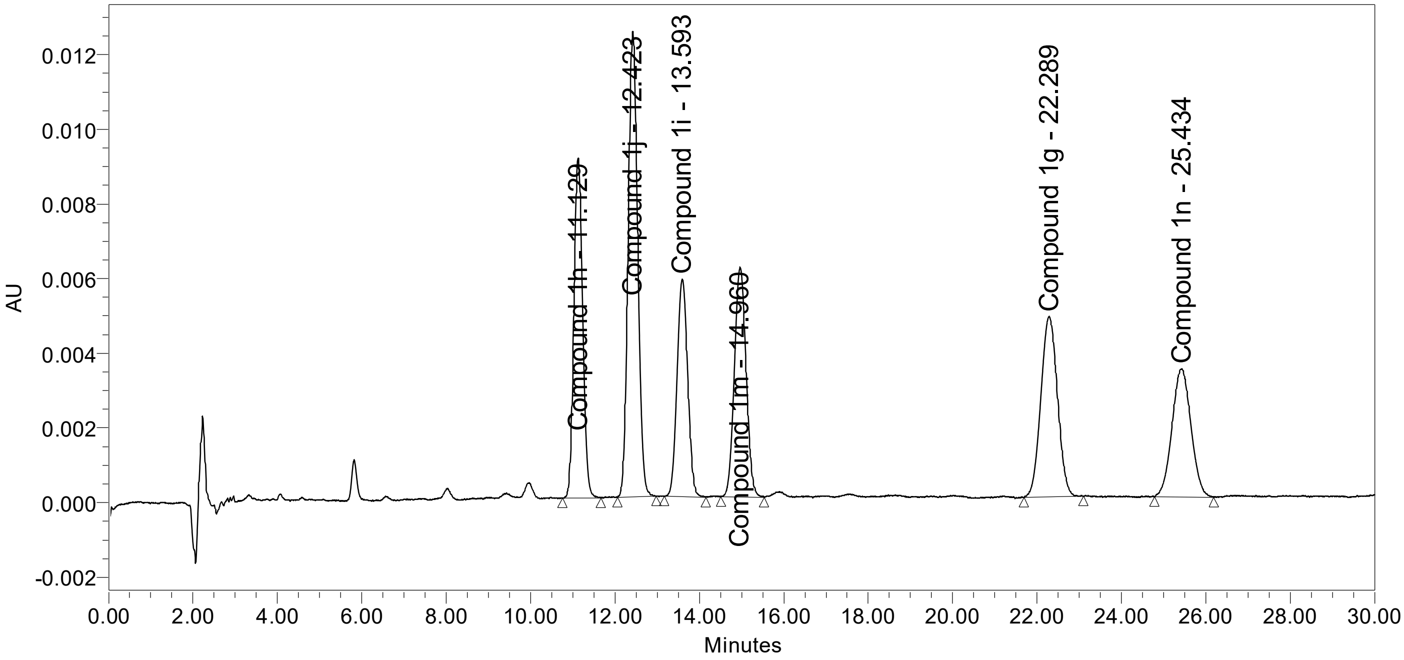

	Peak Name	RT	Area	Height (µV)	Resolution	Level
1	Compound 1h	11.129	133370	9103		120
2	Compound 1j	12.423	200966	12469	3.18	120
3	Compound 1i	13.593	102149	5837	2.63	120
4	Compound 1m	14.960	118926	6159	2.81	120
5	Compound 1g	22.289	133117	4835	11.80	120
6	Compound 1n	25.434	108604	3435	4.00	120

Quantitative determination of

compounds 1g, 1h, 1i, 1j, 1m, 1n

- Validation of the analytical method -

Validation parameters:

- Specificity
- Precision 1
- Precision 2
- LOD – LOQ
- Linearity
- Range
- **Accuracy**
- Robustness

Chloride_Ethyl_Accuracy

Reported by User: Roman Roxana (roman_roxana)
Acquisition Server Waters-cd3
Project Name: Teste\Thiazides
Sample Set Name: Seria Etil_Cl 7 93 Accuracy
Code column: Inertsil ODS-3 250*4.6 mm 5um LA140

SAMPLE INFORMATION

Sample Name:	Solution 5.0 ug/ mL		
Sample Type:	Standard	Date Acquired:	2/11/2023 5:18:21 PM EET
Vial:	1	Acq. Method Set:	Seria Etil_Cl 7 93 Accuracy
Injection #:	1	Date Processed:	4/19/2023 1:39:07 PM EEST
Injection Volume:	10.00 ul	Processing Method:	Seria Etil_Cl 7 93 Accuracy
Run Time:	35.0 Minutes	Channel Name:	Extract 275
Acquired By:	roman_roxana	Proc. Chnl. Descr.:	2998 PDA 275.0 nm (2998

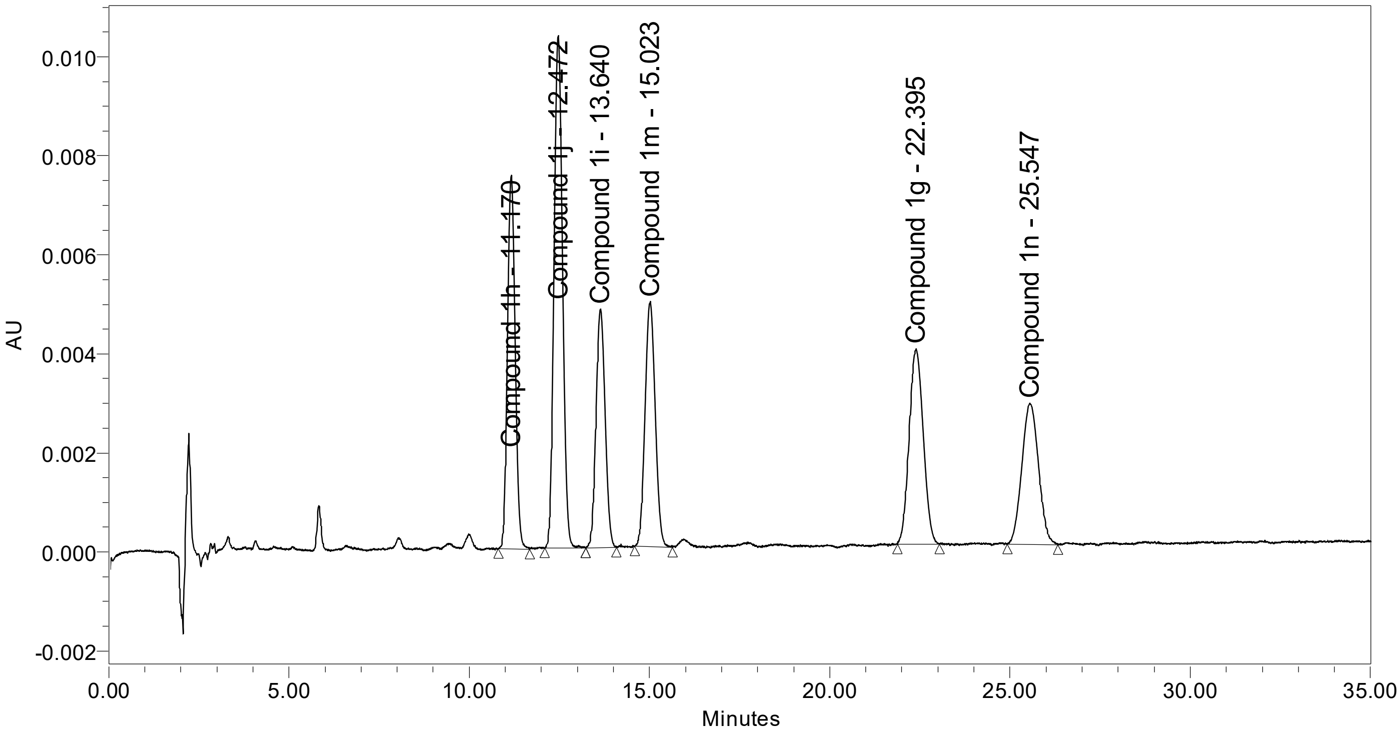

	Peak Name	RT	Area	Height (µV)	Resolution
1	Compound 1h	11.170	111264	7545	
2	Compound 1j	12.472	168093	10349	3.18
3	Compound 1i	13.640	84696	4822	2.61
4	Compound 1m	15.023	96091	4950	2.82
5	Compound 1g	22.395	108286	3943	11.78

	Peak Name	RT	Area	Height (µV)	Resolution
6	Compound 1n	25.547	90633	2849	4.00

Chloride_Ethyl_Accuracy

Reported by User: Roman Roxana (roman_roxana)
Acquisition Server Waters-cd3
Project Name: Teste\Thiazides
Sample Set Name: Seria Etil_Cl 7 93 Accuracy
Code column: Inertsil ODS-3 250*4.6 mm 5um LA140

SAMPLE INFORMATION

Sample Name:	Solution 5.0 ug/ mL		
Sample Type:	Standard	Date Acquired:	2/11/2023 5:53:59 PM EET
Vial:	1	Acq. Method Set:	Seria Etil_Cl 7 93 Accuracy
Injection #:	2	Date Processed:	4/19/2023 1:39:23 PM EEST
Injection Volume:	10.00 ul	Processing Method:	Seria Etil_Cl 7 93 Accuracy
Run Time:	35.0 Minutes	Channel Name:	Extract 275
Acquired By:	roman_roxana	Proc. Chnl. Descr.:	2998 PDA 275.0 nm (2998

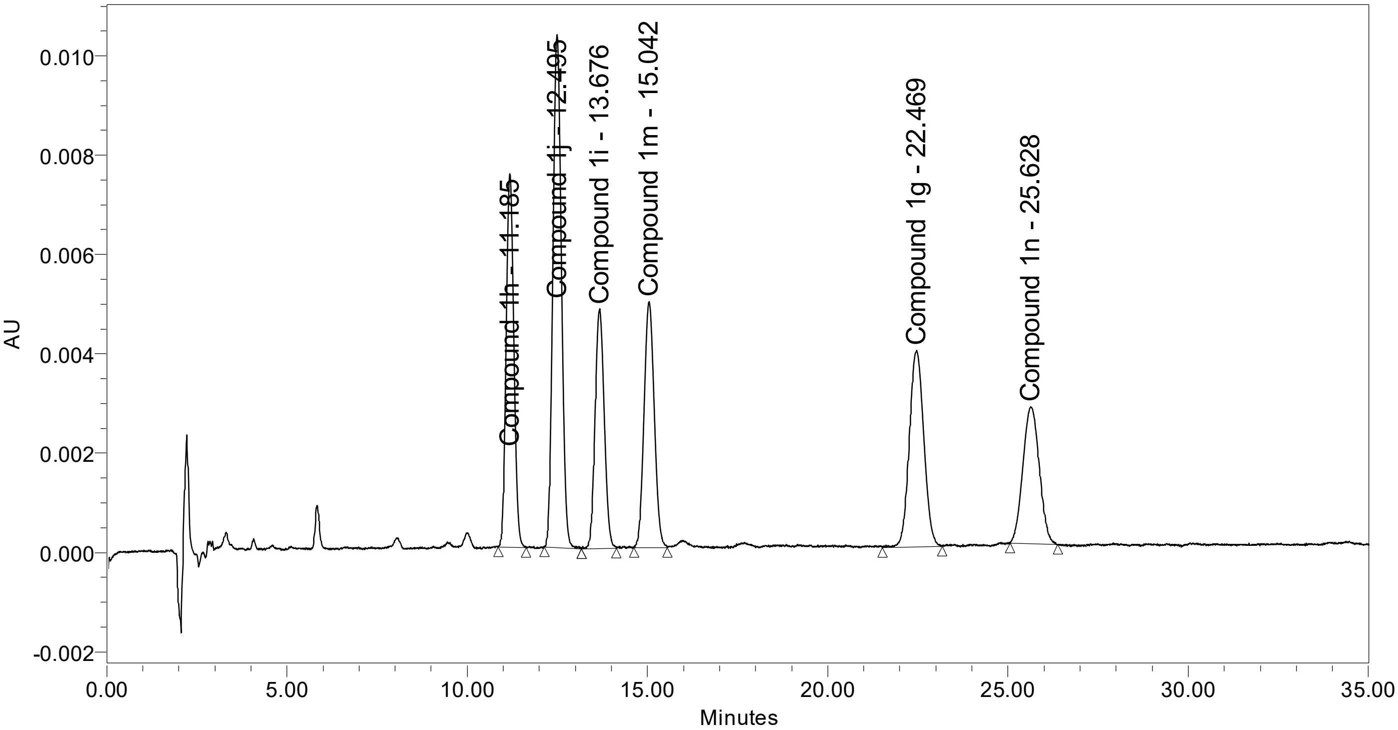

	Peak Name	RT	Area	Height (μV)	Resolution
1	Compound 1h	11.185	110378	7518	
2	Compound 1j	12.495	167763	10330	3.20
3	Compound 1i	13.676	85020	4820	2.63
4	Compound 1m	15.042	96322	4952	2.78
5	Compound 1g	22.469	110962	3958	11.81

	Peak Name	RT	Area	Height (μV)	Resolution
6	Compound 1n	25.628	87515	2768	3.97

Chloride_Ethyl_Accuracy

Reported by User: Roman Roxana (roman_roxana)
Acquisition Server Waters-cd3
Project Name: Teste\Thiazides
Sample Set Name: Seria Etil_Cl 7 93 Accuracy
Code column: Inertsil ODS-3 250*4.6 mm 5um LA140

SAMPLE INFORMATION

Sample Name:	Solution 5.0 ug/ mL		
Sample Type:	Standard	Date Acquired:	2/11/2023 6:29:37 PM EET
Vial:	1	Acq. Method Set:	Seria Etil_Cl 7 93 Accuracy
Injection #:	3	Date Processed:	4/19/2023 1:39:25 PM EEST
Injection Volume:	10.00 ul	Processing Method:	Seria Etil_Cl 7 93 Accuracy
Run Time:	35.0 Minutes	Channel Name:	Extract 275
Acquired By:	roman_roxana	Proc. Chnl. Descr.:	2998 PDA 275.0 nm (2998

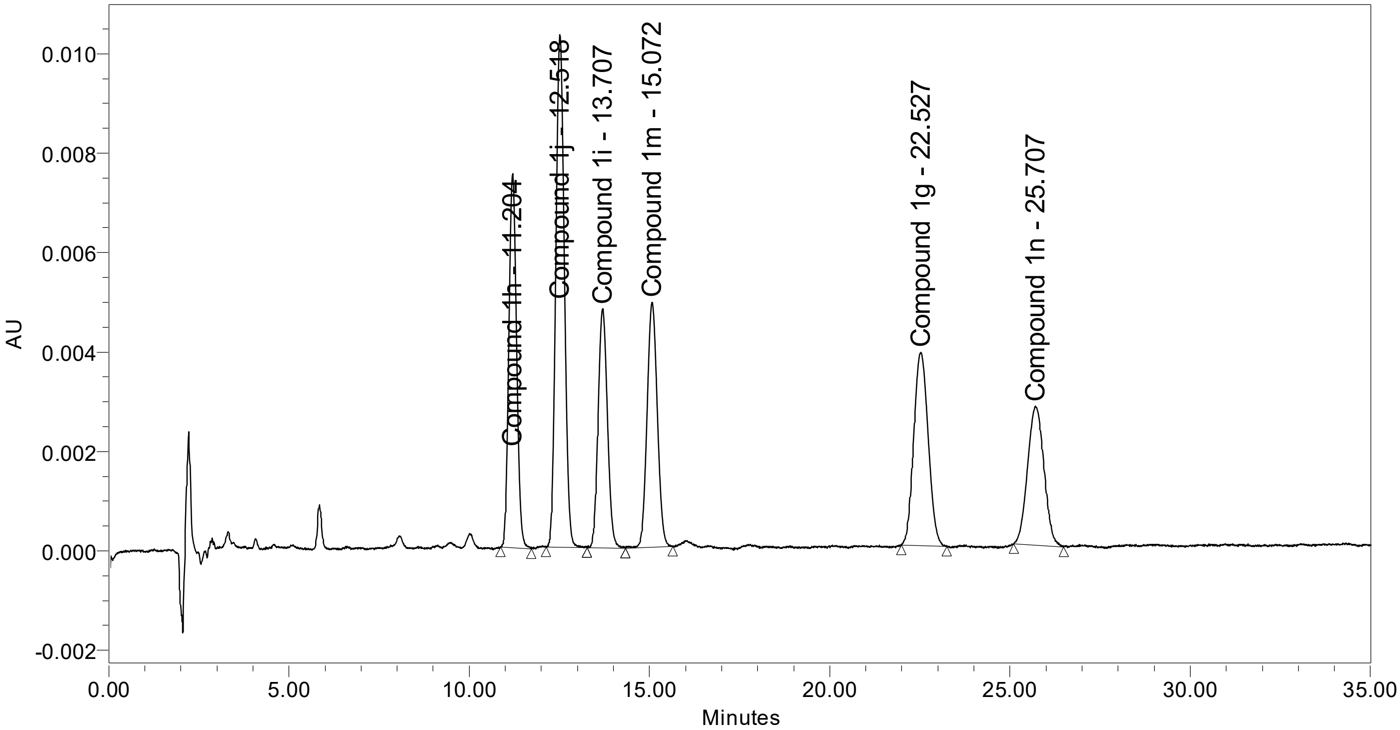

	Peak Name	RT	Area	Height (µV)	Resolution
1	Compound 1h	11.204	111083	7520	
2	Compound 1j	12.518	167826	10310	3.21
3	Compound 1i	13.707	85713	4810	2.65
4	Compound 1m	15.072	96276	4927	2.78
5	Compound 1g	22.527	108186	3894	11.86

	Peak Name	RT	Area	Height (µV)	Resolution
6	Compound 1n	25.707	88689	2796	4.00

Empower software

Component Summary Area Time

Reported by User: Roman Roxana (roman_roxana)
Acquisition Server Waters-cd3

Project Name: Teste\Thiazides
Sample Set Name: Seria Etil_Cl 7 93 Accuracy
Code column: Inertsil ODS-3 250*4.6 mm 5 um LA140

Retention Time Summarized by Name
Channel: 2998

	SampleName	Inj	Channel	Vial	Compound 1h	Compound 1j	Compound 1i	Compound 1m
1	Solution 5.0 ug/ mL	1	2998	1	11.170	12.472	13.640	15.023
2	Solution 5.0 ug/ mL	2	2998	1	11.185	12.495	13.676	15.042
3	Solution 5.0 ug/ mL	3	2998	1	11.204	12.518	13.707	15.072
Mean					11.187	12.495	13.674	15.046
Std. Dev.					0.017	0.023	0.033	0.025
% RSD					0.15	0.18	0.24	0.16

Retention Time Summarized by Name
Channel: 2998

	Compound 1g	Compound 1n
1	22.395	25.547
2	22.469	25.628
3	22.527	25.707
Mean	22.464	25.627
Std. Dev.	0.066	0.080
% RSD	0.30	0.31

Component Summary Area Time

Reported by User: Roman Roxana (roman_roxana)
Acquisition Server Waters-cd3

Project Name: Teste\Thiazides
Sample Set Name: Seria Etil_Cl 7 93 Accuracy
Code column: Inertsil ODS-3 250*4.6 mm 5 um LA140

Area Summarized by Name
Channel: 2998

	SampleName	Inj	Channel	Vial	Compound 1h	Compound 1j	Compound 1i	Compound 1m
1	Solution 5.0 ug/ mL	1	2998	1	111264	168093	84696	96091
2	Solution 5.0 ug/ mL	2	2998	1	110378	167763	85020	96322
3	Solution 5.0 ug/ mL	3	2998	1	111083	167826	85713	96276
Mean					110908	167894	85143	96230
Std. Dev.					468	175	520	122
% RSD					0.42	0.10	0.61	0.13

Area Summarized by Name
Channel: 2998

	Compound 1g	Compound 1n
1	108286	90633
2	110962	87515
3	108186	88689
Mean	109145	88945
Std. Dev.	1575	1575
% RSD	1.44	1.77

Chloride_Ethyl_Accuracy

Reported by User: Roman Roxana (roman_roxana)
Acquisition Server Waters-cd3
Project Name: Teste\Thiazides
Sample Set Name: Seria Etil_Cl 7 93 Accuracy
Code column: Inertsil ODS-3 250*4.6 mm 5um LA140

SAMPLE INFORMATION

Sample Name:	Solution 4.0 ug/ mL		
Sample Type:	Unknown	Date Acquired:	2/11/2023 7:20:15 PM EET
Vial:	2	Acq. Method Set:	Seria Etil_Cl 7 93 Accuracy
Injection #:	1	Date Processed:	4/19/2023 2:16:58 PM EEST
Injection Volume:	10.00 ul	Processing Method:	Seria Etil_Cl 7 93 Accuracy
Run Time:	35.0 Minutes	Channel Name:	Extract 275
Acquired By:	roman_roxana	Proc. Chnl. Descr.:	2998 PDA 275.0 nm (2998

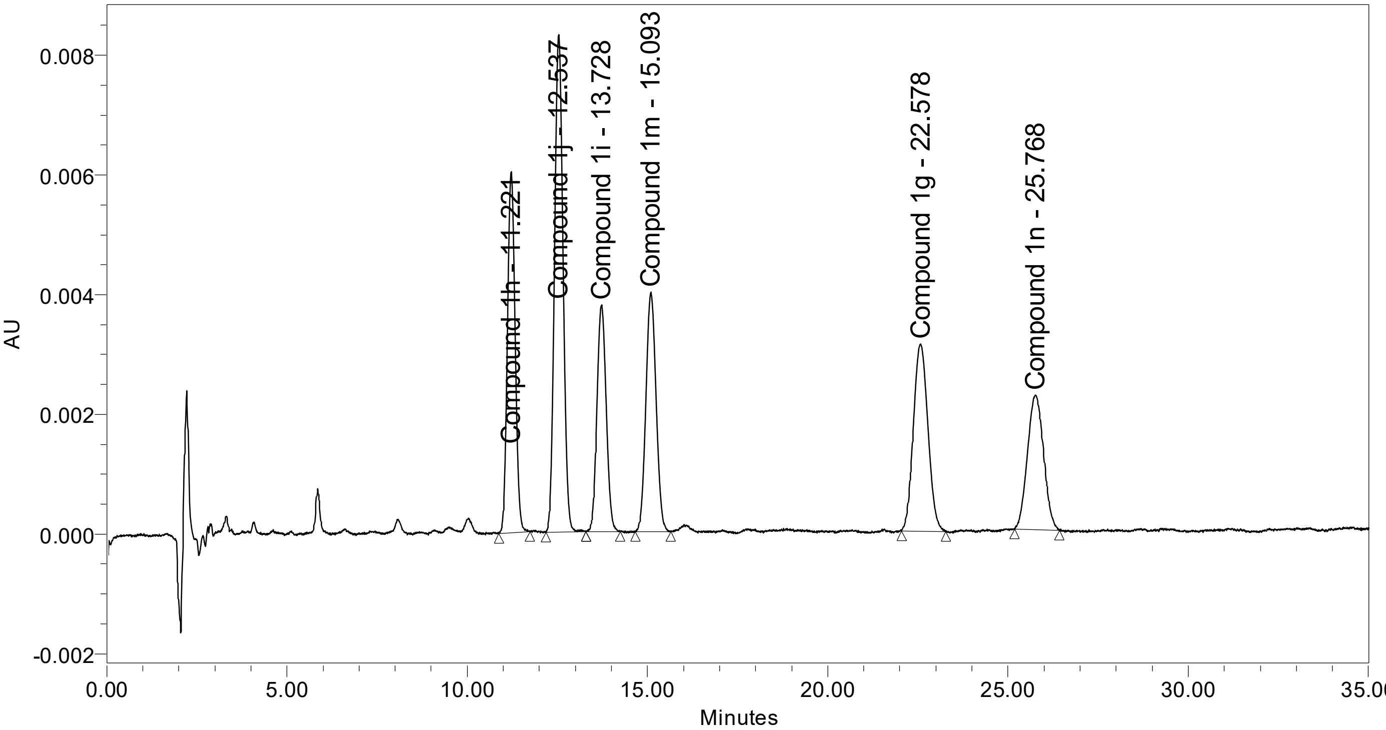

	Peak Name	RT	Area	Height (µV)	Resolution
1	Compound 1h	11.221	88596	6037	
2	Compound 1j	12.537	134788	8308	3.23
3	Compound 1i	13.728	66935	3788	2.65
4	Compound 1m	15.093	77870	4006	2.78
5	Compound 1g	22.578	87506	3130	11.91
6	Compound 1n	25.768	70454	2248	4.02

Chloride_Ethyl_Accuracy

Reported by User: Roman Roxana (roman_roxana)
Acquisition Server Waters-cd3
Project Name: Teste\Thiazides
Sample Set Name: Seria Etil_Cl 7 93 Accuracy
Code column: Inertsil ODS-3 250*4.6 mm 5um LA140

SAMPLE INFORMATION

Sample Name:	Solution 4.0 ug/ mL		
Sample Type:	Unknown	Date Acquired:	2/11/2023 7:55:54 PM EET
Vial:	3	Acq. Method Set:	Seria Etil_Cl 7 93 Accuracy
Injection #:	1	Date Processed:	4/19/2023 1:42:12 PM EEST
Injection Volume:	10.00 ul	Processing Method:	Seria Etil_Cl 7 93 Accuracy
Run Time:	35.0 Minutes	Channel Name:	Extract 275
Acquired By:	roman_roxana	Proc. Chnl. Descr.:	2998 PDA 275.0 nm (2998

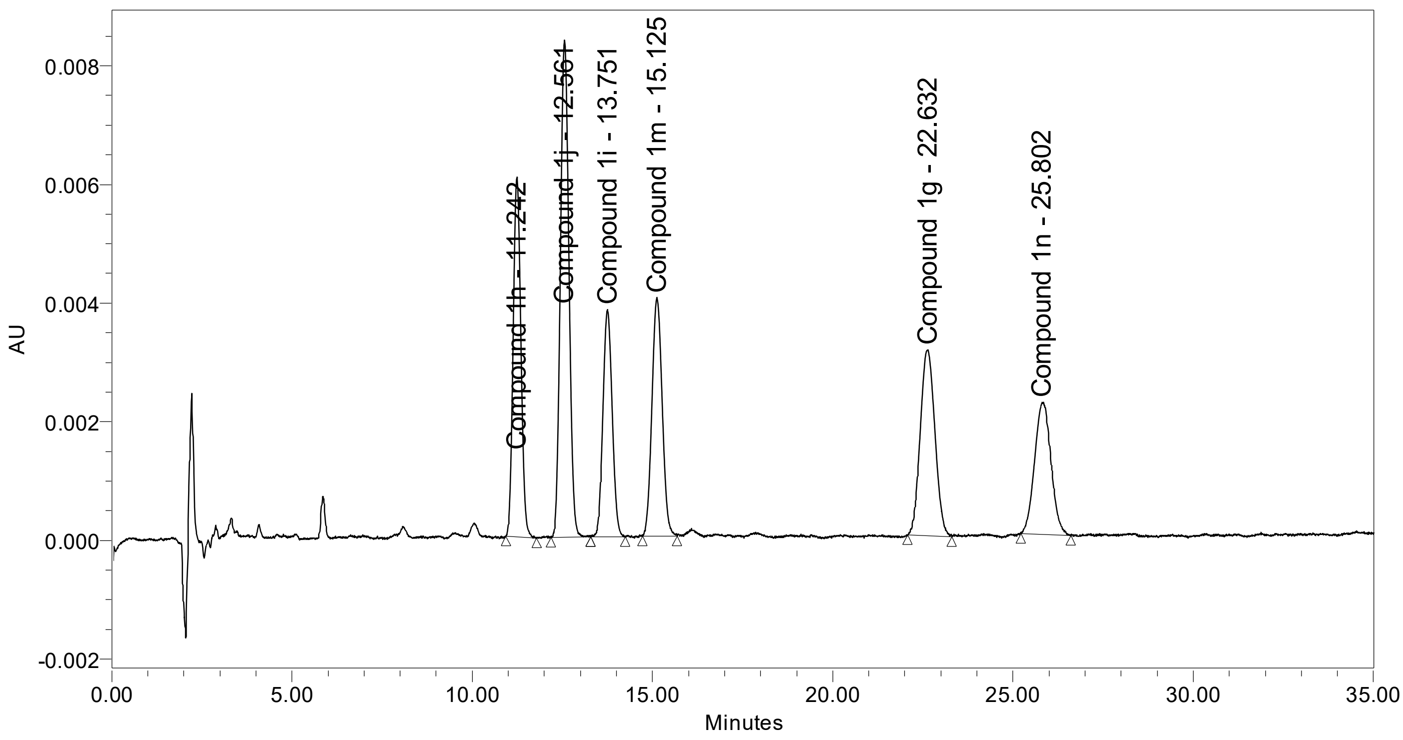

	Peak Name	RT	Area	Height (µV)	Resolution
1	Compound 1h	11.242	89424	6068	
2	Compound 1j	12.561	136160	8381	3.23
3	Compound 1i	13.751	67660	3831	2.65
4	Compound 1m	15.125	78492	4020	2.79
5	Compound 1g	22.632	88169	3138	11.88

	Peak Name	RT	Area	Height (µV)	Resolution
6	Compound 1n	25.802	70865	2225	3.97

Chloride_Ethyl_Accuracy

Reported by User: Roman Roxana (roman_roxana)
Acquisition Server Waters-cd3
Project Name: Teste\Thiazides
Sample Set Name: Seria Etil_Cl 7 93 Accuracy
Code column: Inertsil ODS-3 250*4.6 mm 5um LA140

SAMPLE INFORMATION

Sample Name:	Solution 4.0 ug/ mL		
Sample Type:	Unknown	Date Acquired:	2/11/2023 8:31:35 PM EET
Vial:	4	Acq. Method Set:	Seria Etil_Cl 7 93 Accuracy
Injection #:	1	Date Processed:	4/19/2023 2:04:12 PM EEST
Injection Volume:	10.00 ul	Processing Method:	Seria Etil_Cl 7 93 Accuracy
Run Time:	35.0 Minutes	Channel Name:	Extract 275
Acquired By:	roman_roxana	Proc. Chnl. Descr.:	2998 PDA 275.0 nm (2998

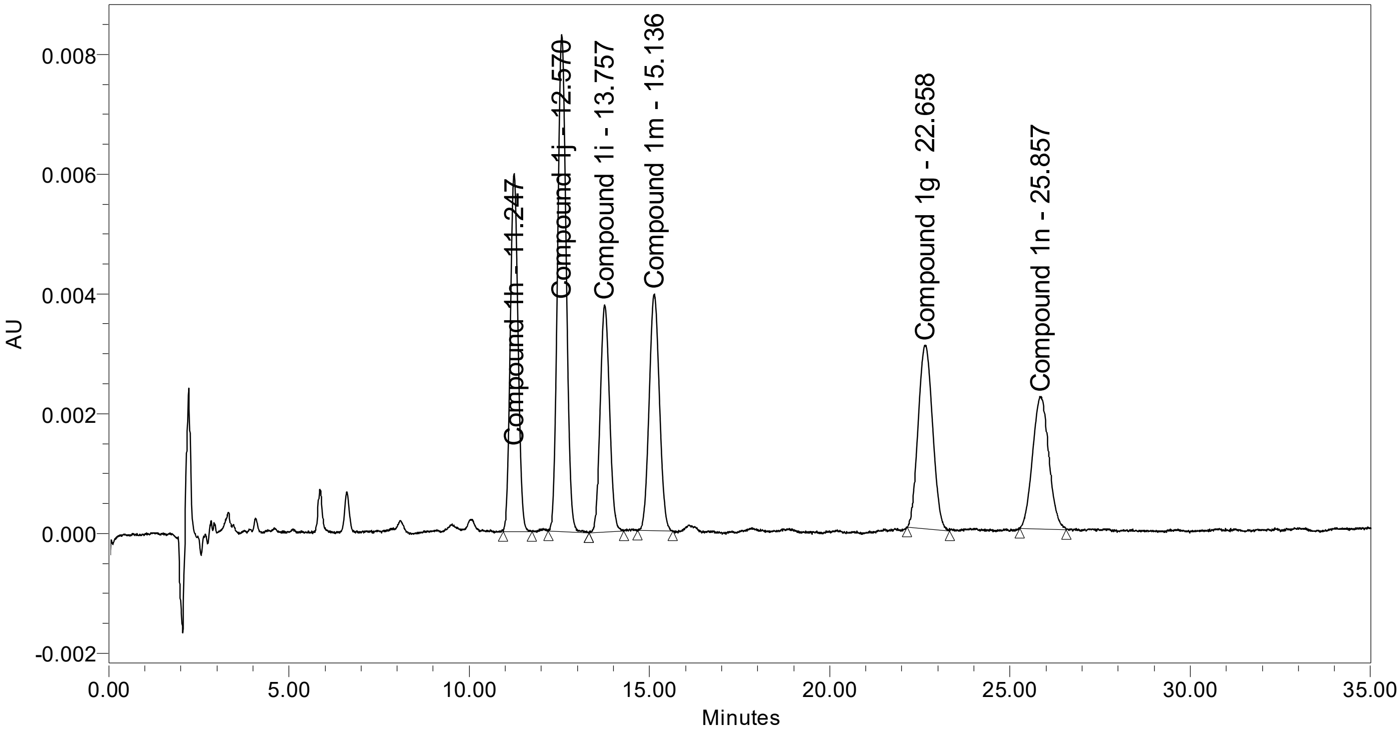

	Peak Name	RT	Area	Height (µV)	Resolution
1	Compound 1h	11.247	88261	5984	
2	Compound 1j	12.570	135576	8295	3.22
3	Compound 1i	13.757	67434	3792	2.63
4	Compound 1m	15.136	77528	3961	2.80
5	Compound 1g	22.658	85025	3068	11.97
6	Compound 1n	25.857	70008	2208	4.05

Chloride_Ethyl_Accuracy

Reported by User: Roman Roxana (roman_roxana)
Acquisition Server Waters-cd3
Project Name: Teste\Thiazides
Sample Set Name: Seria Etil_Cl 7 93 Accuracy
Code column: Inertsil ODS-3 250*4.6 mm 5um LA140

SAMPLE INFORMATION

Sample Name:	Solution 4.0 ug/ mL		
Sample Type:	Unknown	Date Acquired:	2/11/2023 9:07:14 PM EET
Vial:	5	Acq. Method Set:	Seria Etil_Cl 7 93 Accuracy
Injection #:	1	Date Processed:	4/19/2023 1:42:21 PM EEST
Injection Volume:	10.00 ul	Processing Method:	Seria Etil_Cl 7 93 Accuracy
Run Time:	35.0 Minutes	Channel Name:	Extract 275
Acquired By:	roman_roxana	Proc. Chnl. Descr.:	2998 PDA 275.0 nm (2998

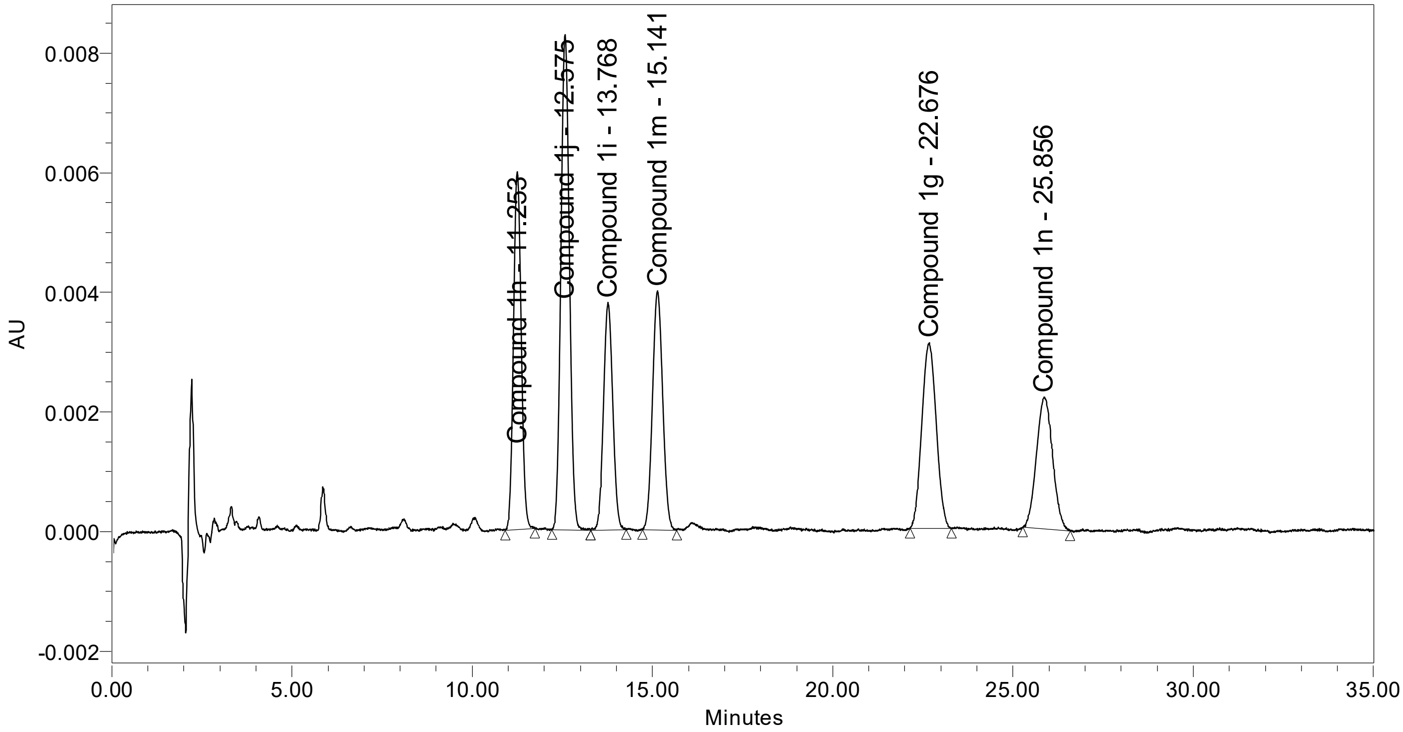

	Peak Name	RT	Area	Height (µV)	Resolution
1	Compound 1h	11.253	88200	5990	
2	Compound 1j	12.575	134401	8282	3.23
3	Compound 1i	13.768	67420	3800	2.66
4	Compound 1m	15.141	77647	3997	2.79
5	Compound 1g	22.676	85606	3113	12.04
6	Compound 1n	25.856	69497	2204	4.02

Chloride_Ethyl_Accuracy

Reported by User: Roman Roxana (roman_roxana)
Acquisition Server Waters-cd3
Project Name: Teste\Thiazides
Sample Set Name: Seria Etil_Cl 7 93 Accuracy
Code column: Inertsil ODS-3 250*4.6 mm 5um LA140

SAMPLE INFORMATION

Sample Name:	Solution 4.0 ug/ mL		
Sample Type:	Unknown	Date Acquired:	2/11/2023 9:42:53 PM EET
Vial:	6	Acq. Method Set:	Seria Etil_Cl 7 93 Accuracy
Injection #:	1	Date Processed:	4/19/2023 1:42:25 PM EEST
Injection Volume:	10.00 ul	Processing Method:	Seria Etil_Cl 7 93 Accuracy
Run Time:	35.0 Minutes	Channel Name:	Extract 275
Acquired By:	roman_roxana	Proc. Chnl. Descr.:	2998 PDA 275.0 nm (2998

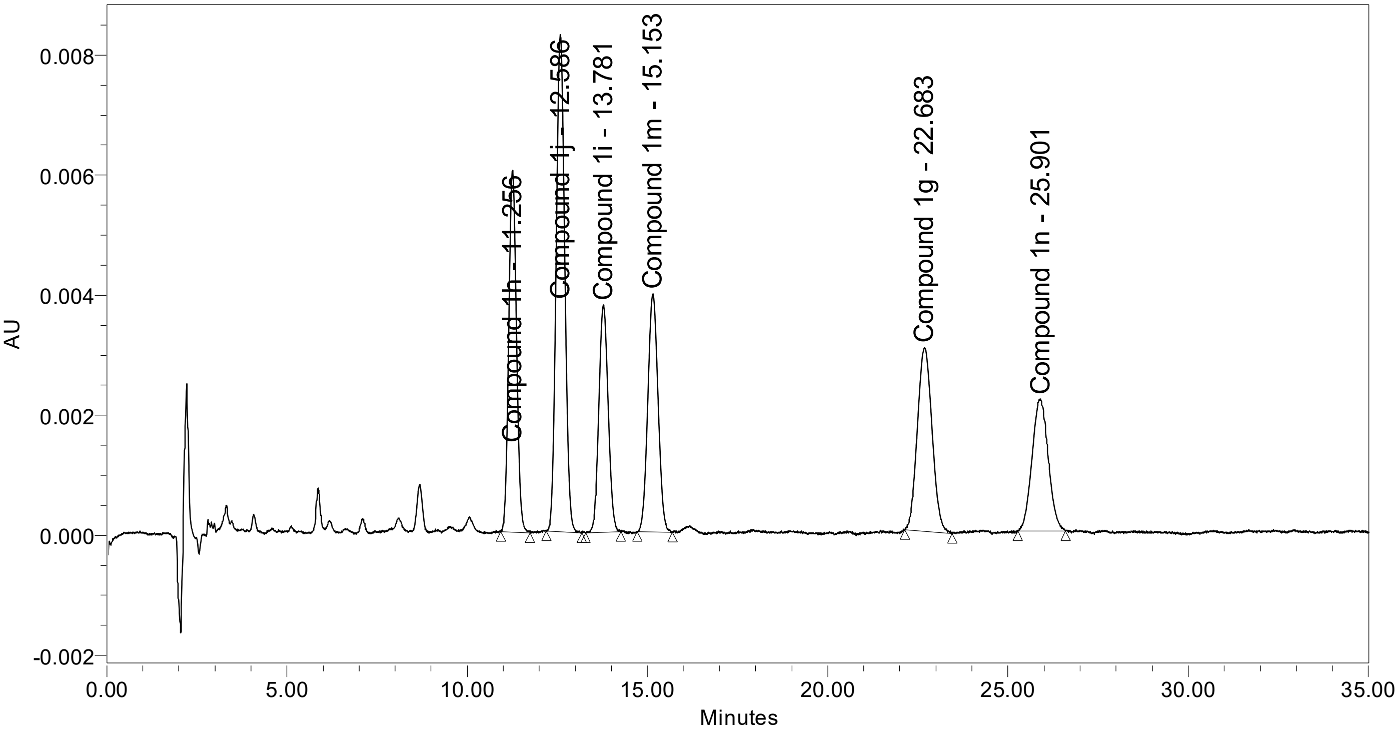

	Peak Name	RT	Area	Height (µV)	Resolution
1	Compound 1h	11.256	88987	6031	
2	Compound 1j	12.586	135422	8287	3.24
3	Compound 1i	13.781	67585	3786	2.64
4	Compound 1m	15.153	77469	3968	2.77
5	Compound 1g	22.683	86004	3062	11.94
6	Compound 1n	25.901	69537	2198	4.04

Chloride_Ethyl_Accuracy

Reported by User: Roman Roxana (roman_roxana)
Acquisition Server Waters-cd3
Project Name: Teste\Thiazides
Sample Set Name: Seria Etil_Cl 7 93 Accuracy
Code column: Inertsil ODS-3 250*4.6 mm 5um LA140

SAMPLE INFORMATION

Sample Name:	Solution 4.0 ug/ mL		
Sample Type:	Unknown	Date Acquired:	2/11/2023 10:18:33 PM EET
Vial:	7	Acq. Method Set:	Seria Etil_Cl 7 93 Accuracy
Injection #:	1	Date Processed:	4/19/2023 2:17:55 PM EEST
Injection Volume:	10.00 ul	Processing Method:	Seria Etil_Cl 7 93 Accuracy
Run Time:	35.0 Minutes	Channel Name:	Extract 275
Acquired By:	roman_roxana	Proc. Chnl. Descr.:	2998 PDA 275.0 nm (2998

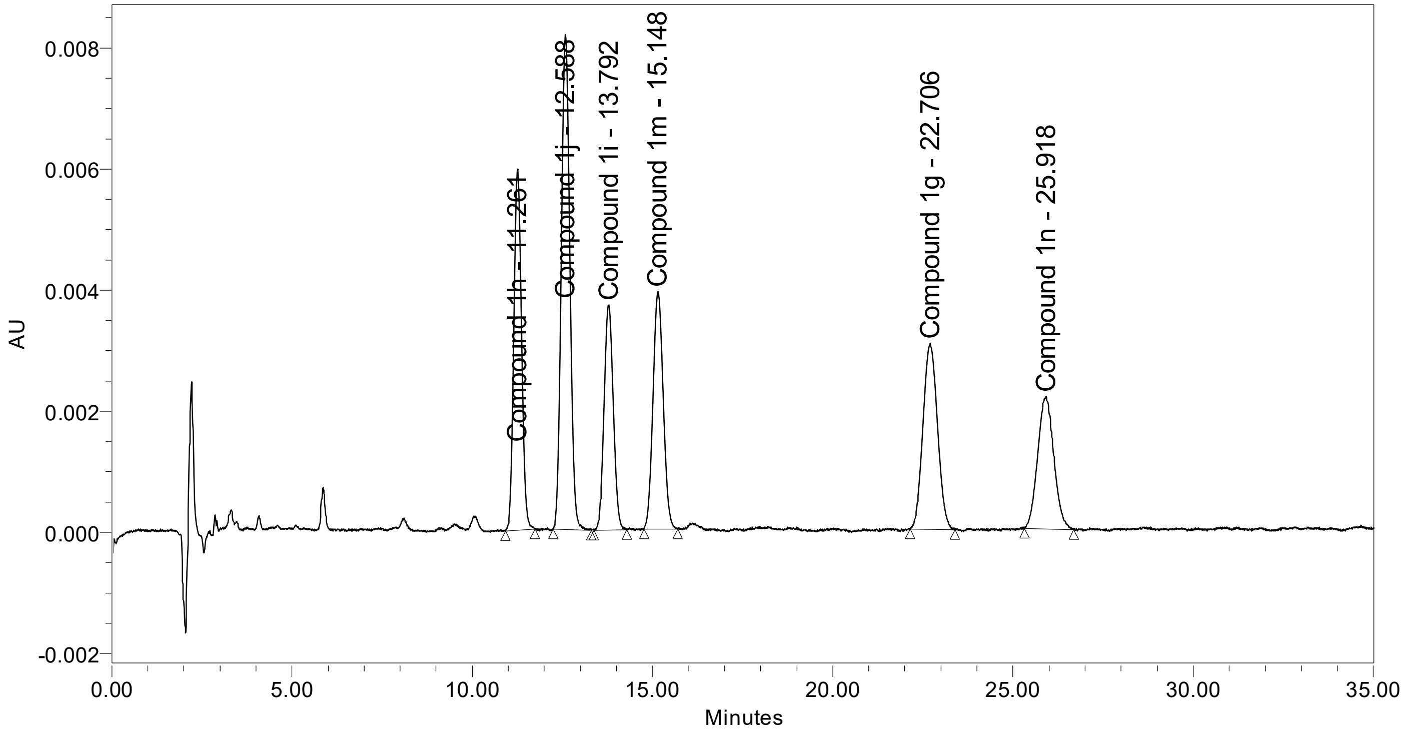

	Peak Name	RT	Area	Height (µV)	Resolution
1	Compound 1h	11.261	87697	5958	
2	Compound 1j	12.588	132917	8167	3.24
3	Compound 1i	13.792	65504	3721	2.69
4	Compound 1m	15.148	76178	3921	2.77
5	Compound 1g	22.706	84949	3066	12.01
6	Compound 1n	25.918	68989	2185	4.07

Chloride_Ethyl_Accuracy

Reported by User: Roman Roxana (roman_roxana)
Acquisition Server Waters-cd3
Project Name: Teste\Thiazides
Sample Set Name: Seria Etil_Cl 7 93 Accuracy
Code column: Inertsil ODS-3 250*4.6 mm 5um LA140

SAMPLE INFORMATION

Sample Name:	Solution 5.0 ug/ mL		
Sample Type:	Unknown	Date Acquired:	2/11/2023 10:54:16 PM EET
Vial:	8	Acq. Method Set:	Seria Etil_Cl 7 93 Accuracy
Injection #:	1	Date Processed:	4/19/2023 2:19:19 PM EEST
Injection Volume:	10.00 ul	Processing Method:	Seria Etil_Cl 7 93 Accuracy
Run Time:	35.0 Minutes	Channel Name:	Extract 275
Acquired By:	roman_roxana	Proc. Chnl. Descr.:	2998 PDA 275.0 nm (2998

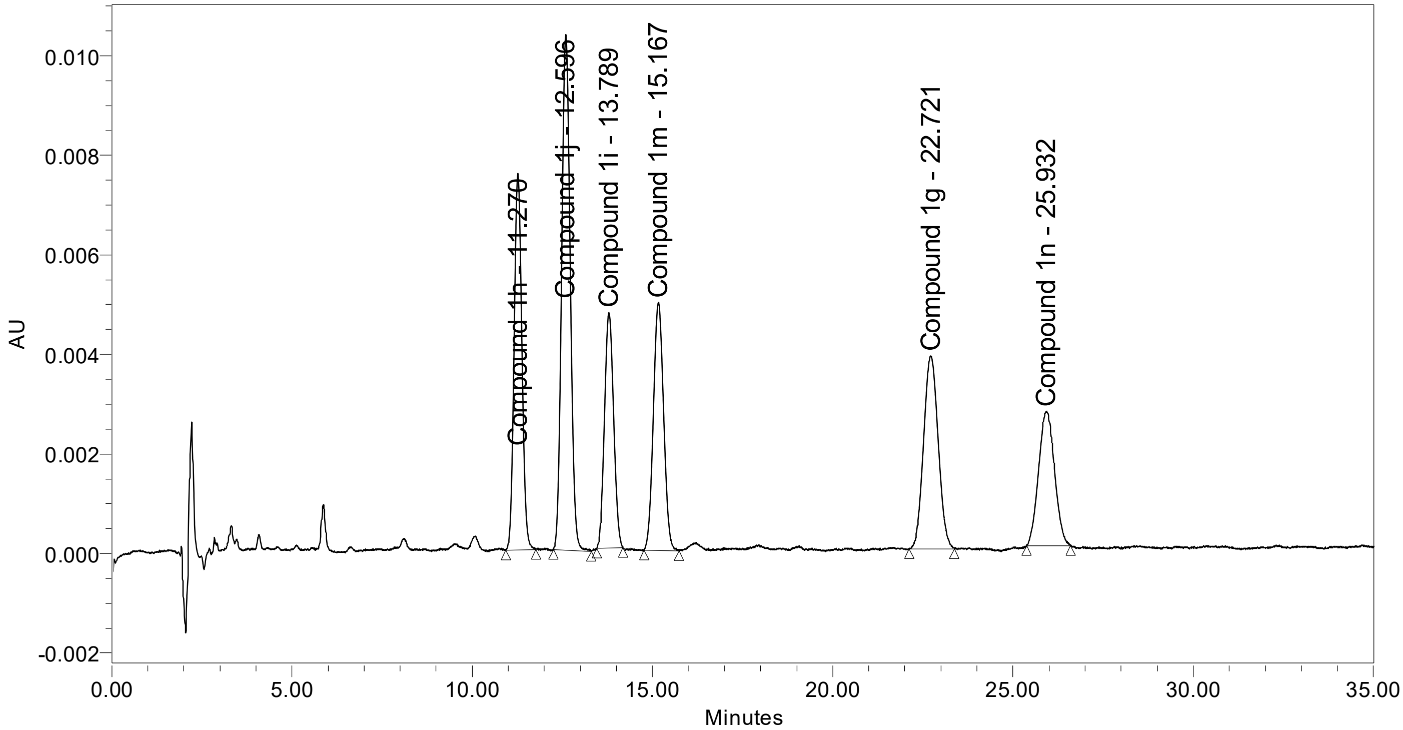

	Peak Name	RT	Area	Height (µV)	Resolution
1	Compound 1h	11.270	111591	7560	
2	Compound 1j	12.596	169135	10358	3.24
3	Compound 1i	13.789	82009	4729	2.67
4	Compound 1m	15.167	97170	4975	2.81
5	Compound 1g	22.721	108491	3876	11.98

	Peak Name	RT	Area	Height (µV)	Resolution
6	Compound 1n	25.932	84215	2703	4.06

Chloride_Ethyl_Accuracy

Reported by User: Roman Roxana (roman_roxana)
Acquisition Server Waters-cd3
Project Name: Teste\Thiazides
Sample Set Name: Seria Etil_Cl 7 93 Accuracy
Code column: Inertsil ODS-3 250*4.6 mm 5um LA140

SAMPLE INFORMATION

Sample Name:	Solution 5.0 ug/ mL		
Sample Type:	Unknown	Date Acquired:	2/11/2023 11:29:57 PM EET
Vial:	9	Acq. Method Set:	Seria Etil_Cl 7 93 Accuracy
Injection #:	1	Date Processed:	4/19/2023 2:20:41 PM EEST
Injection Volume:	10.00 ul	Processing Method:	Seria Etil_Cl 7 93 Accuracy
Run Time:	35.0 Minutes	Channel Name:	Extract 275
Acquired By:	roman_roxana	Proc. Chnl. Descr.:	2998 PDA 275.0 nm (2998

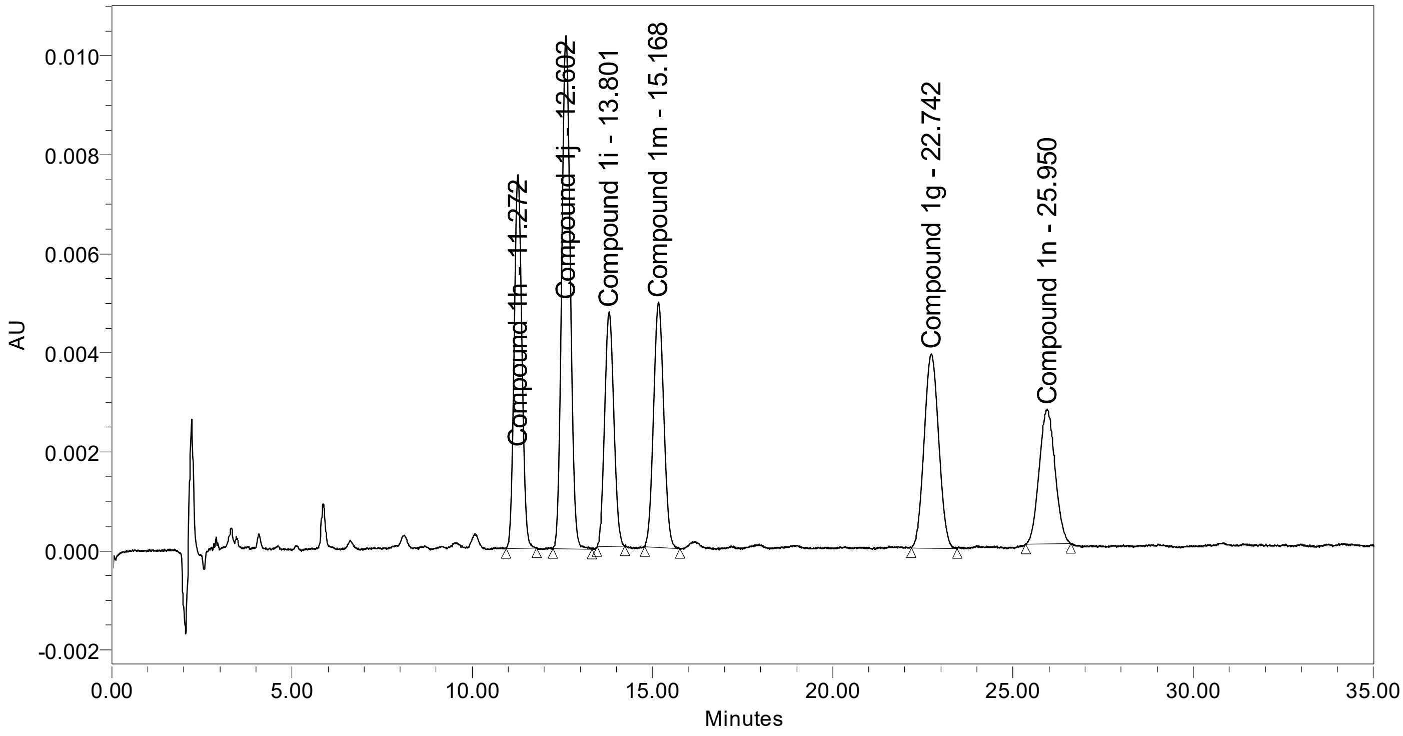

	Peak Name	RT	Area	Height (µV)	Resolution
1	Compound 1h	11.272	111110	7556	
2	Compound 1j	12.602	168465	10362	3.26
3	Compound 1i	13.801	82735	4741	2.68
4	Compound 1m	15.168	96179	4953	2.79
5	Compound 1g	22.742	109245	3926	12.05

	Peak Name	RT	Area	Height (µV)	Resolution
6	Compound 1n	25.950	84630	2728	4.06

Chloride_Ethyl_Accuracy

Reported by User: Roman Roxana (roman_roxana)
Acquisition Server Waters-cd3
Project Name: Teste\Thiazides
Sample Set Name: Seria Etil_Cl 7 93 Accuracy
Code column: Inertsil ODS-3 250*4.6 mm 5um LA140

SAMPLE INFORMATION

Sample Name:	Solution 5.0 ug/ mL		
Sample Type:	Unknown	Date Acquired:	2/12/2023 12:05:37 AM EET
Vial:	10	Acq. Method Set:	Seria Etil_Cl 7 93 Accuracy
Injection #:	1	Date Processed:	4/19/2023 2:21:41 PM EEST
Injection Volume:	10.00 ul	Processing Method:	Seria Etil_Cl 7 93 Accuracy
Run Time:	35.0 Minutes	Channel Name:	Extract 275
Acquired By:	roman_roxana	Proc. Chnl. Descr.:	2998 PDA 275.0 nm (2998

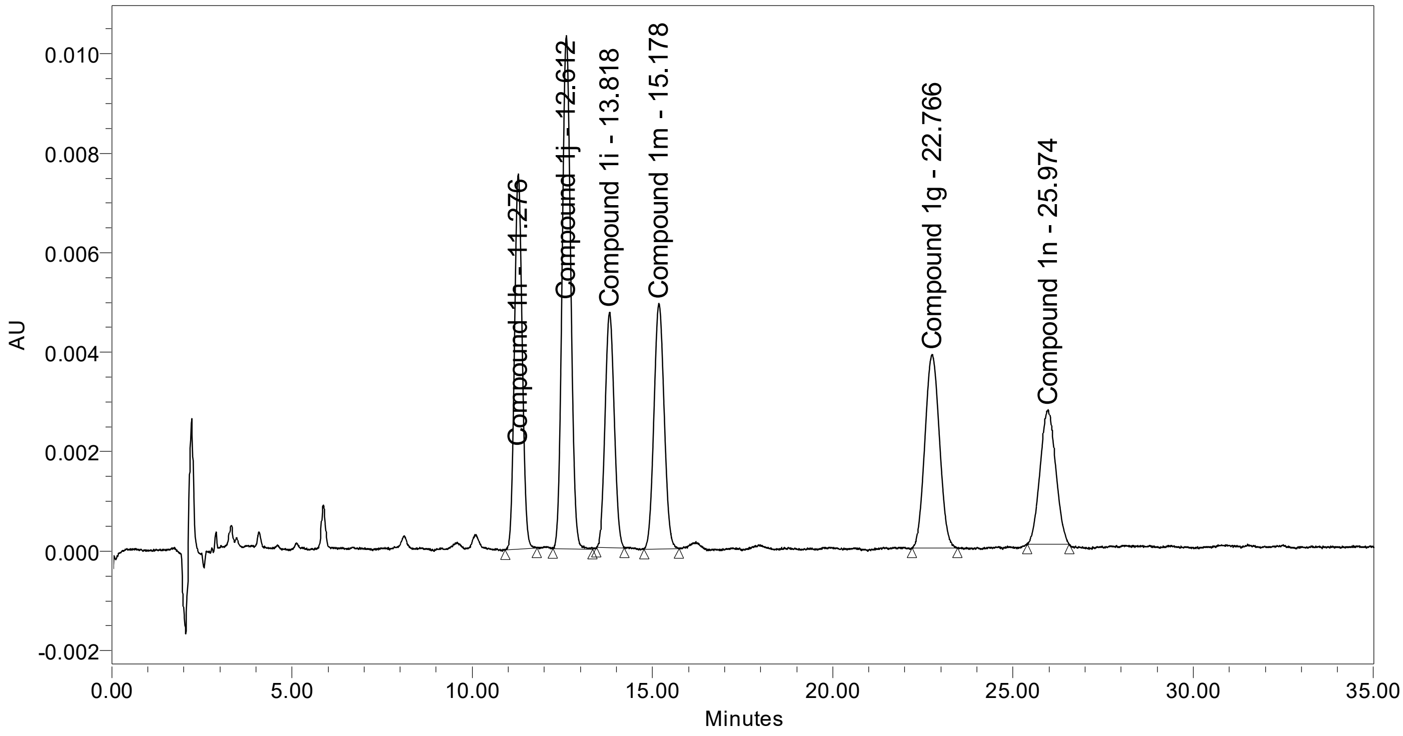

	Peak Name	RT	Area	Height (µV)	Resolution
1	Compound 1h	11.276	111024	7548	
2	Compound 1j	12.612	168231	10320	3.27
3	Compound 1i	13.818	82661	4734	2.69
4	Compound 1m	15.178	96381	4942	2.77
5	Compound 1g	22.766	108577	3894	12.07

	Peak Name	RT	Area	Height (µV)	Resolution
6	Compound 1n	25.974	82741	2697	4.08

Chloride_Ethyl_Accuracy

Reported by User: Roman Roxana (roman_roxana)
Acquisition Server Waters-cd3
Project Name: Teste\Thiazides
Sample Set Name: Seria Etil_Cl 7 93 Accuracy
Code column: Inertsil ODS-3 250*4.6 mm 5um LA140

SAMPLE INFORMATION

Sample Name:	Solution 5.0 ug/ mL		
Sample Type:	Unknown	Date Acquired:	2/12/2023 12:41:19 AM EET
Vial:	11	Acq. Method Set:	Seria Etil_Cl 7 93 Accuracy
Injection #:	1	Date Processed:	4/19/2023 2:22:11 PM EEST
Injection Volume:	10.00 ul	Processing Method:	Seria Etil_Cl 7 93 Accuracy
Run Time:	35.0 Minutes	Channel Name:	Extract 275
Acquired By:	roman_roxana	Proc. Chnl. Descr.:	2998 PDA 275.0 nm (2998

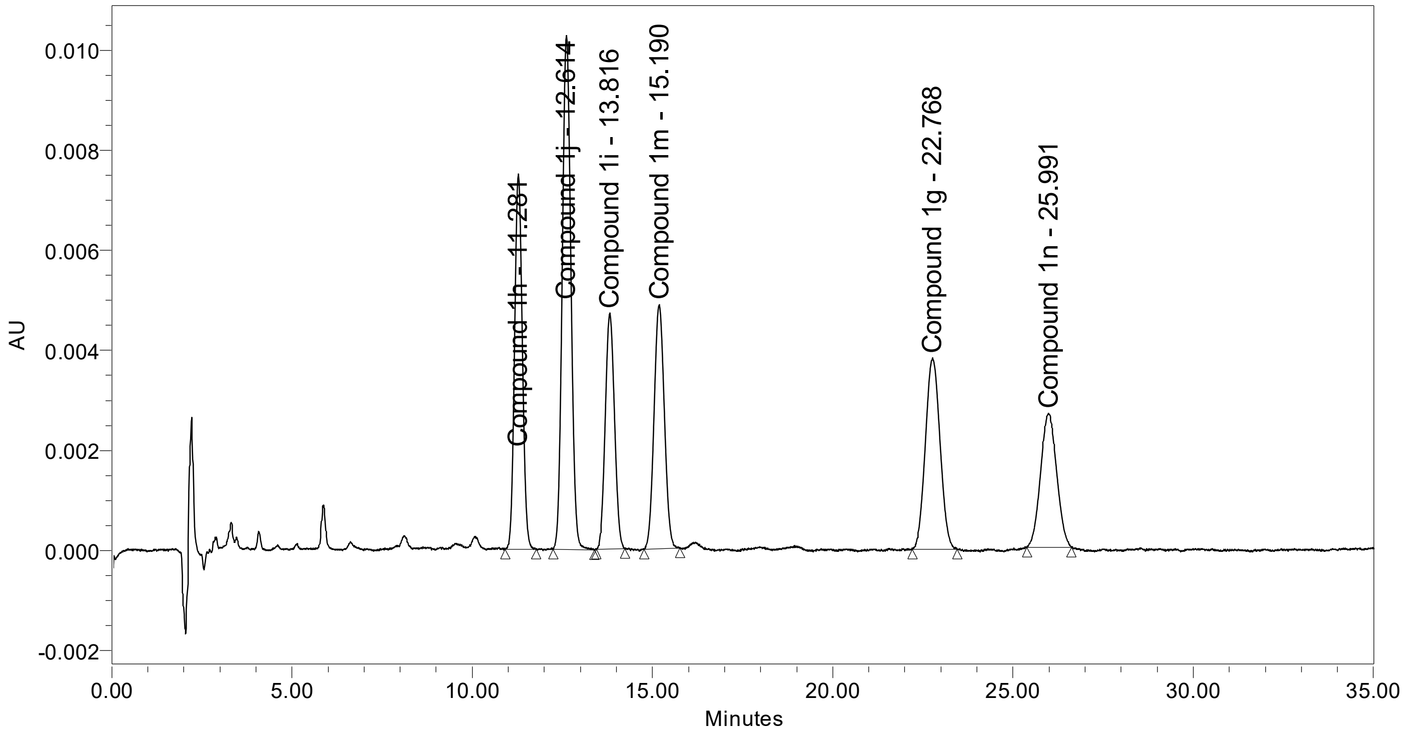

	Peak Name	RT	Area	Height (µV)	Resolution
1	Compound 1h	11.281	110663	7501	
2	Compound 1j	12.614	168785	10270	3.24
3	Compound 1i	13.816	82849	4720	2.67
4	Compound 1m	15.190	95668	4879	2.78
5	Compound 1g	22.768	107246	3831	12.02

	Peak Name	RT	Area	Height (µV)	Resolution
6	Compound 1n	25.991	83968	2673	4.05

Chloride_Ethyl_Accuracy

Reported by User: Roman Roxana (roman_roxana)
Acquisition Server Waters-cd3
Project Name: Teste\Thiazides
Sample Set Name: Seria Etil_Cl 7 93 Accuracy
Code column: Inertsil ODS-3 250*4.6 mm 5um LA140

SAMPLE INFORMATION

Sample Name:	Solution 5.0 ug/ mL		
Sample Type:	Unknown	Date Acquired:	2/12/2023 1:17:02 AM EET
Vial:	12	Acq. Method Set:	Seria Etil_Cl 7 93 Accuracy
Injection #:	1	Date Processed:	4/19/2023 2:23:16 PM EEST
Injection Volume:	10.00 ul	Processing Method:	Seria Etil_Cl 7 93 Accuracy
Run Time:	35.0 Minutes	Channel Name:	Extract 275
Acquired By:	roman_roxana	Proc. Chnl. Descr.:	2998 PDA 275.0 nm (2998

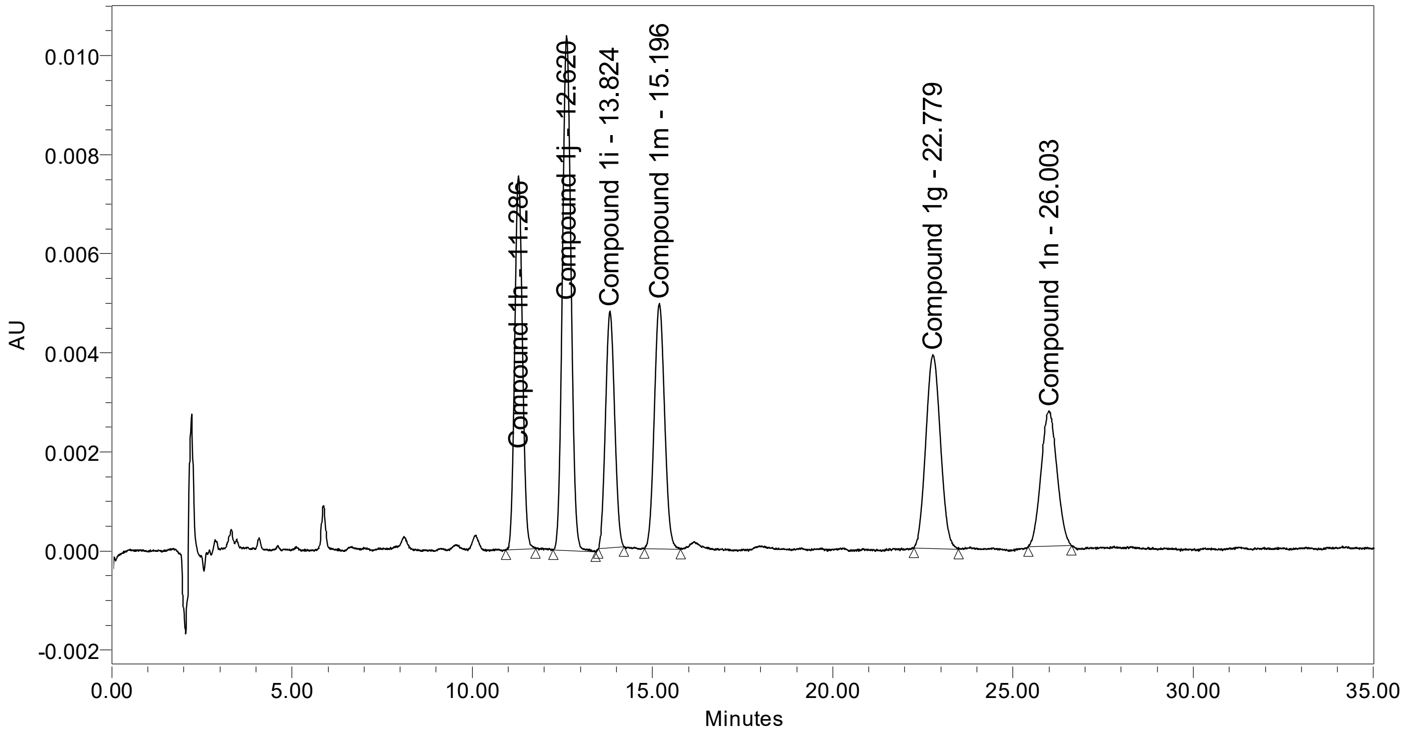

	Peak Name	RT	Area	Height (μV)	Resolution
1	Compound 1h	11.286	110997	7538	
2	Compound 1j	12.620	169796	10394	3.25
3	Compound 1i	13.824	82632	4775	2.69
4	Compound 1m	15.196	97012	4948	2.80
5	Compound 1g	22.779	108996	3908	12.07

	Peak Name	RT	Area	Height (μV)	Resolution
6	Compound 1n	26.003	84959	2724	4.08

Chloride_Ethyl_Accuracy

Reported by User: Roman Roxana (roman_roxana)
Acquisition Server Waters-cd3
Project Name: Teste\Thiazides
Sample Set Name: Seria Etil_Cl 7 93 Accuracy
Code column: Inertsil ODS-3 250*4.6 mm 5um LA140

S A M P L E I N F O R M A T I O N

Sample Name:	Solution 5.0 ug/ mL		
Sample Type:	Unknown	Date Acquired:	2/12/2023 1:52:42 AM EET
Vial:	13	Acq. Method Set:	Seria Etil_Cl 7 93 Accuracy
Injection #:	1	Date Processed:	4/19/2023 2:23:44 PM EEST
Injection Volume:	10.00 ul	Processing Method:	Seria Etil_Cl 7 93 Accuracy
Run Time:	35.0 Minutes	Channel Name:	Extract 275
Acquired By:	roman_roxana	Proc. Chnl. Descr.:	2998 PDA 275.0 nm (2998

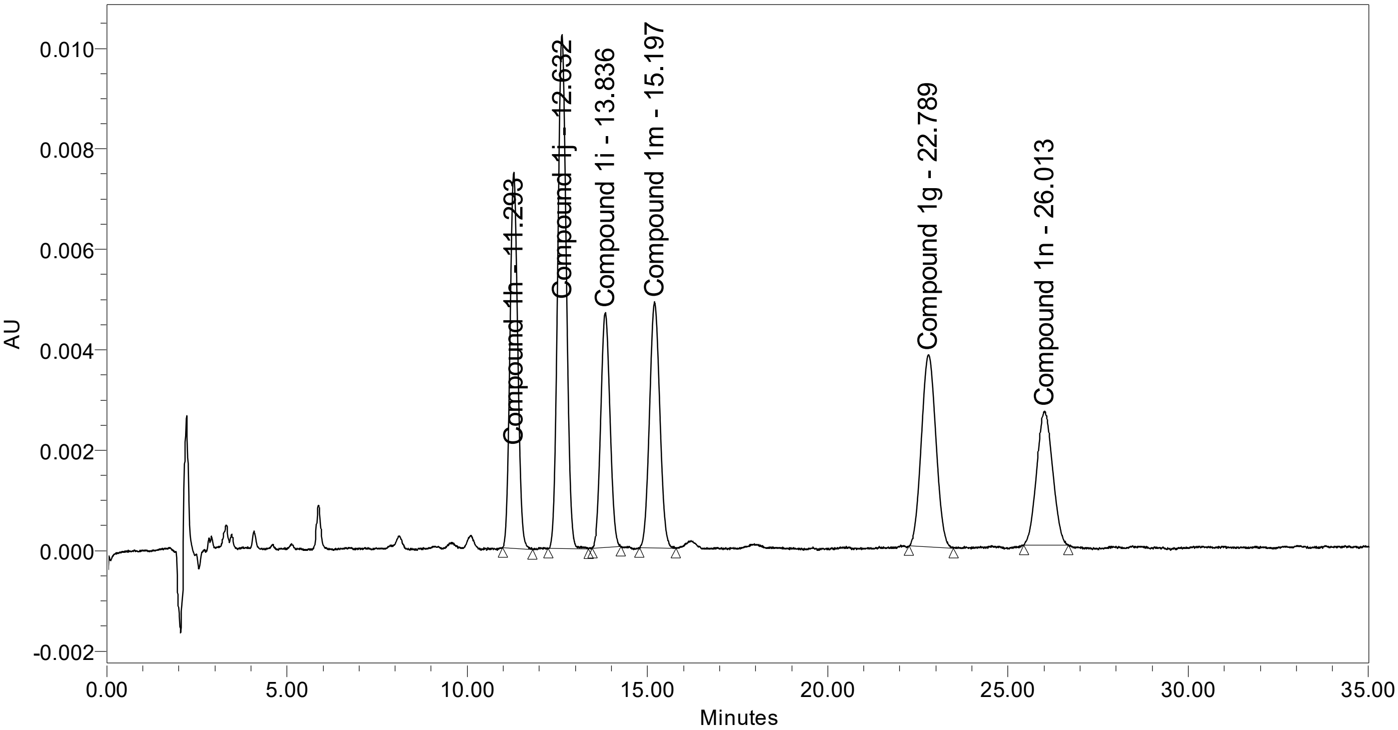

	Peak Name	RT	Area	Height (µV)	Resolution
1	Compound 1h	11.293	110417	7486	
2	Compound 1j	12.632	166835	10236	3.26
3	Compound 1i	13.836	82261	4679	2.68
4	Compound 1m	15.197	96243	4902	2.76
5	Compound 1g	22.789	106276	3822	12.03

	Peak Name	RT	Area	Height (µV)	Resolution
6	Compound 1n	26.013	83531	2673	4.06

Chloride_Ethyl_Accuracy

Reported by User: Roman Roxana (roman_roxana)
Acquisition Server Waters-cd3
Project Name: Teste\Thiazides
Sample Set Name: Seria Etil_Cl 7 93 Accuracy
Code column: Inertsil ODS-3 250*4.6 mm 5um LA140

SAMPLE INFORMATION

Sample Name:	Solution 6.0 ug/ mL		
Sample Type:	Unknown	Date Acquired:	2/12/2023 2:28:24 AM EET
Vial:	14	Acq. Method Set:	Seria Etil_Cl 7 93 Accuracy
Injection #:	1	Date Processed:	4/19/2023 1:44:01 PM EEST
Injection Volume:	10.00 ul	Processing Method:	Seria Etil_Cl 7 93 Accuracy
Run Time:	35.0 Minutes	Channel Name:	Extract 275
Acquired By:	roman_roxana	Proc. Chnl. Descr.:	2998 PDA 275.0 nm (2998

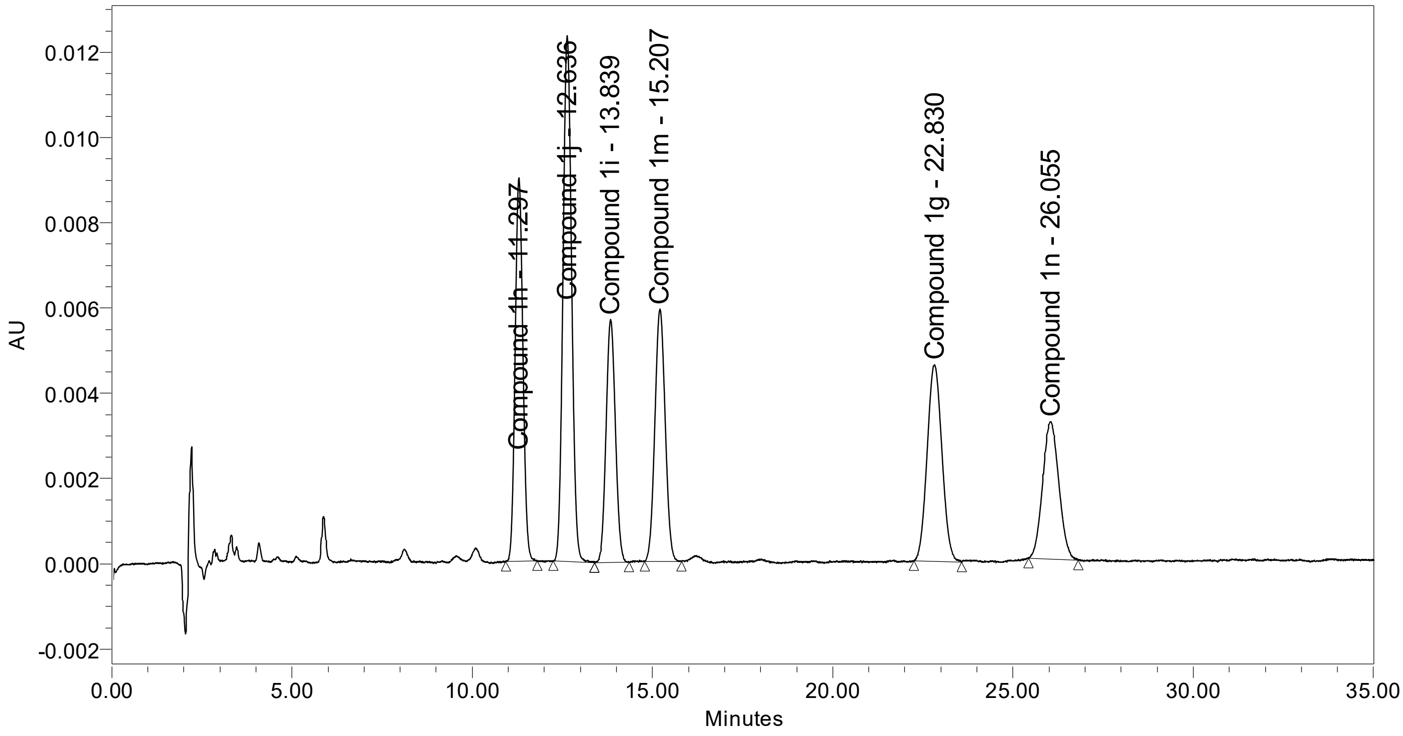

	Peak Name	RT	Area	Height (µV)	Resolution
1	Compound 1h	11.297	132813	9000	
2	Compound 1j	12.636	201924	12341	3.26
3	Compound 1i	13.839	101600	5700	2.66
4	Compound 1m	15.207	116348	5919	2.76
5	Compound 1g	22.830	129988	4614	12.02

	Peak Name	RT	Area	Height (µV)	Resolution
6	Compound 1n	26.055	102402	3229	4.05

Chloride_Ethyl_Accuracy

Reported by User: Roman Roxana (roman_roxana)
Acquisition Server Waters-cd3
Project Name: Teste\Thiazides
Sample Set Name: Seria Etil_Cl 7 93 Accuracy
Code column: Inertsil ODS-3 250*4.6 mm 5um LA140

SAMPLE INFORMATION

Sample Name:	Solution 6.0 ug/ mL		
Sample Type:	Unknown	Date Acquired:	2/12/2023 3:04:05 AM EET
Vial:	15	Acq. Method Set:	Seria Etil_Cl 7 93 Accuracy
Injection #:	1	Date Processed:	4/19/2023 2:25:25 PM EEST
Injection Volume:	10.00 ul	Processing Method:	Seria Etil_Cl 7 93 Accuracy
Run Time:	35.0 Minutes	Channel Name:	Extract 275
Acquired By:	roman_roxana	Proc. Chnl. Descr.:	2998 PDA 275.0 nm (2998

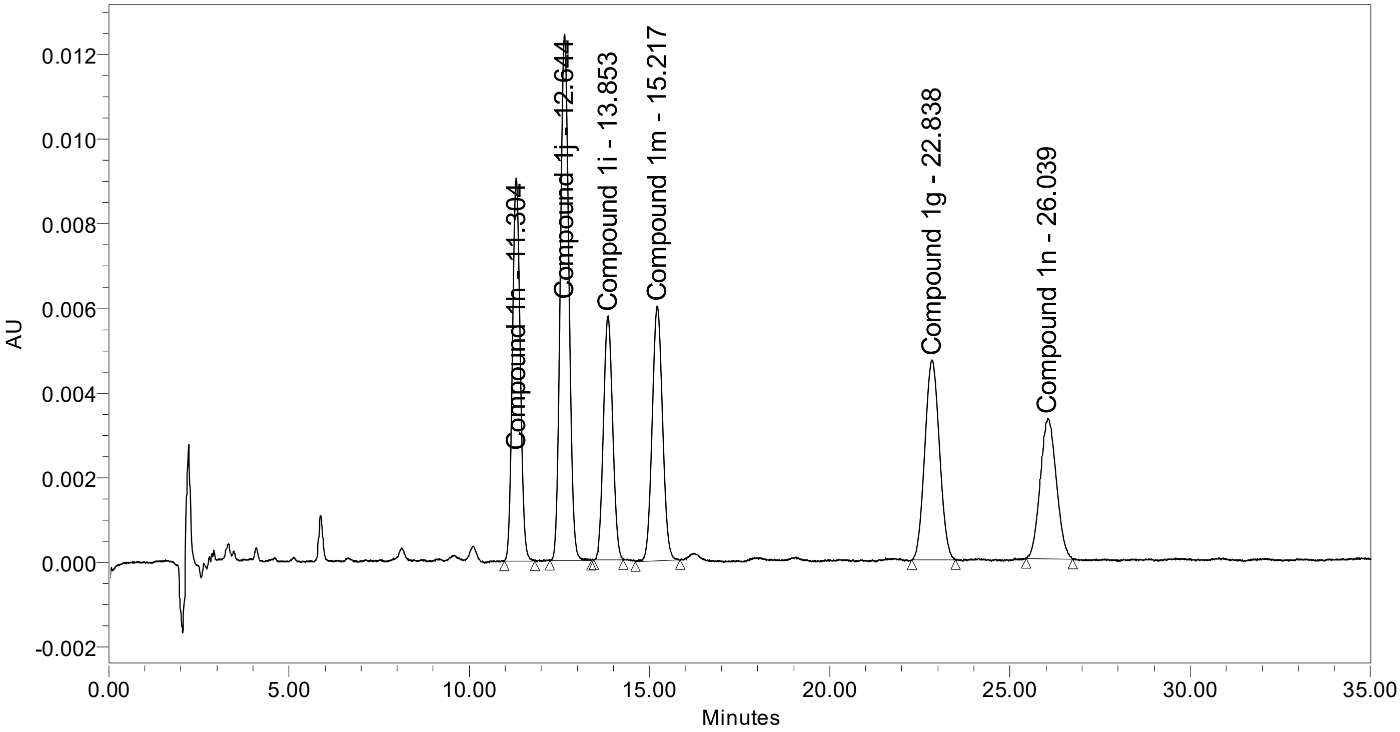

	Peak Name	RT	Area	Height (µV)	Resolution
1	Compound 1h	11.304	133659	9042	
2	Compound 1j	12.644	202900	12423	3.26
3	Compound 1i	13.853	101352	5755	2.68
4	Compound 1m	15.217	118831	6017	2.76
5	Compound 1g	22.838	131659	4717	12.06
6	Compound 1n	26.039	104769	3315	4.02

Chloride_Ethyl_Accuracy

Reported by User: Roman Roxana (roman_roxana)
Acquisition Server Waters-cd3
Project Name: Teste\Thiazides
Sample Set Name: Seria Etil_Cl 7 93 Accuracy
Code column: Inertsil ODS-3 250*4.6 mm 5um LA140

SAMPLE INFORMATION

Sample Name:	Solution 6.0 ug/ mL		
Sample Type:	Unknown	Date Acquired:	2/12/2023 3:39:46 AM EET
Vial:	16	Acq. Method Set:	Seria Etil_Cl 7 93 Accuracy
Injection #:	1	Date Processed:	4/19/2023 2:31:47 PM EEST
Injection Volume:	10.00 ul	Processing Method:	Seria Etil_Cl 7 93 Accuracy
Run Time:	35.0 Minutes	Channel Name:	Extract 275
Acquired By:	roman_roxana	Proc. Chnl. Descr.:	2998 PDA 275.0 nm (2998

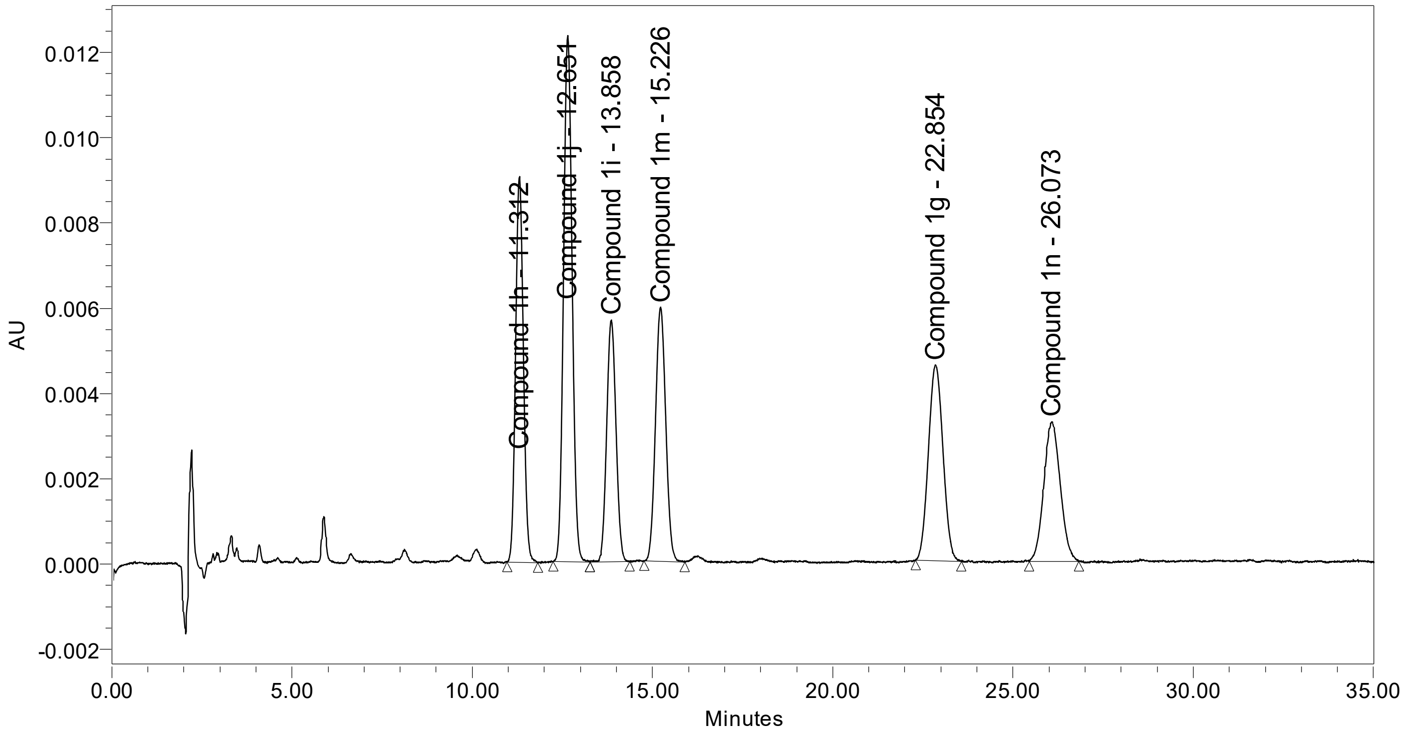

	Peak Name	RT	Area	Height (µV)	Resolution
1	Compound 1h	11.312	134236	9046	
2	Compound 1j	12.651	201415	12342	3.26
3	Compound 1i	13.858	100869	5677	2.67
4	Compound 1m	15.226	117002	5963	2.76
5	Compound 1g	22.854	127548	4591	12.12
6	Compound 1n	26.073	105123	3272	4.05

Chloride_Ethyl_Accuracy

Reported by User: Roman Roxana (roman_roxana)
Acquisition Server Waters-cd3
Project Name: Teste\Thiazides
Sample Set Name: Seria Etil_Cl 7 93 Accuracy
Code column: Inertsil ODS-3 250*4.6 mm 5um LA140

SAMPLE INFORMATION

Sample Name:	Solution 6.0 ug/ mL		
Sample Type:	Unknown	Date Acquired:	2/12/2023 4:15:26 AM EET
Vial:	17	Acq. Method Set:	Seria Etil_Cl 7 93 Accuracy
Injection #:	1	Date Processed:	4/19/2023 2:15:44 PM EEST
Injection Volume:	10.00 ul	Processing Method:	Seria Etil_Cl 7 93 Accuracy
Run Time:	35.0 Minutes	Channel Name:	Extract 275
Acquired By:	roman_roxana	Proc. Chnl. Descr.:	2998 PDA 275.0 nm (2998

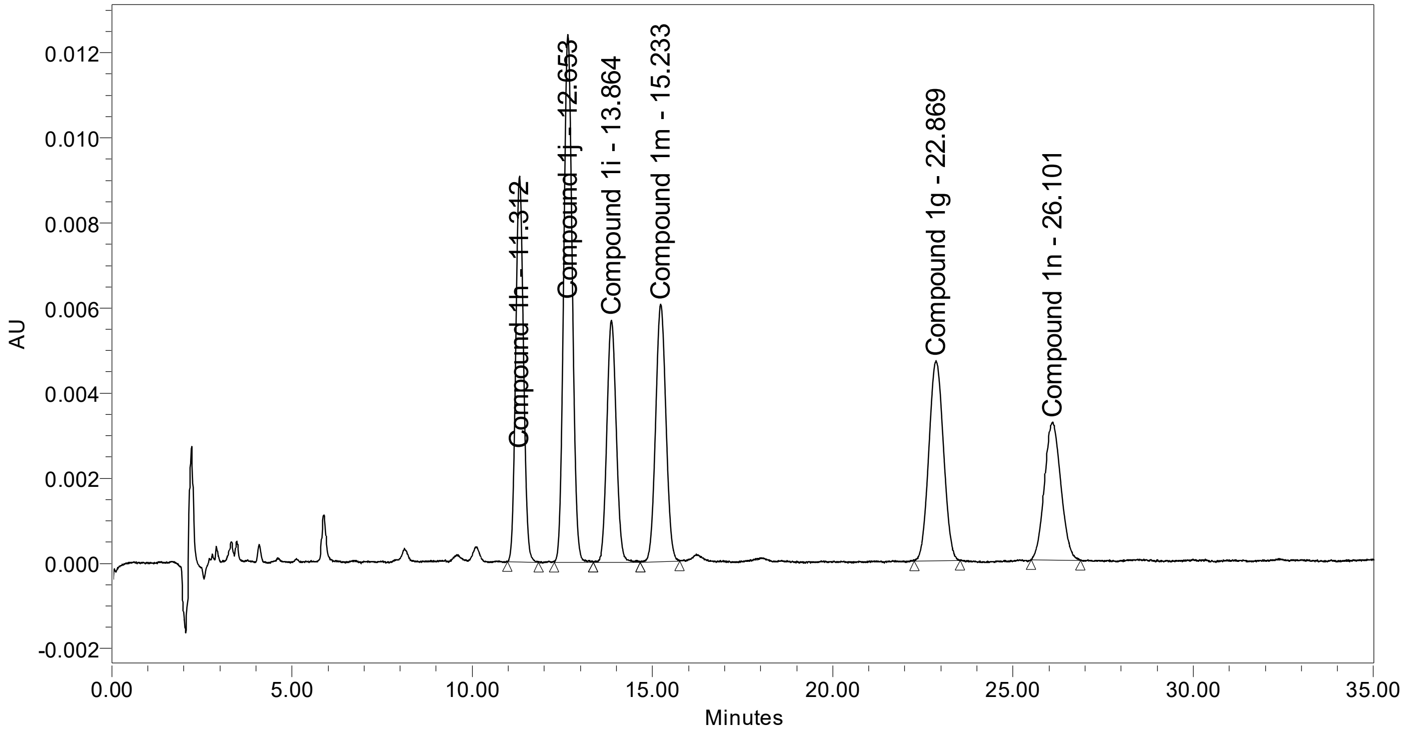

	Peak Name	RT	Area	Height (µV)	Resolution
1	Compound 1h	11.312	133529	9067	
2	Compound 1j	12.653	202073	12400	3.28
3	Compound 1i	13.864	102102	5691	2.69
4	Compound 1m	15.233	119030	6046	2.77
5	Compound 1g	22.869	131006	4702	12.10
6	Compound 1n	26.101	104052	3244	4.05

Chloride_Ethyl_Accuracy

Reported by User: Roman Roxana (roman_roxana)
Acquisition Server Waters-cd3
Project Name: Teste\Thiazides
Sample Set Name: Seria Etil_Cl 7 93 Accuracy
Code column: Inertsil ODS-3 250*4.6 mm 5um LA140

SAMPLE INFORMATION

Sample Name:	Solution 6.0 ug/ mL		
Sample Type:	Unknown	Date Acquired:	2/12/2023 4:51:06 AM EET
Vial:	18	Acq. Method Set:	Seria Etil_Cl 7 93 Accuracy
Injection #:	1	Date Processed:	4/19/2023 2:26:24 PM EEST
Injection Volume:	10.00 ul	Processing Method:	Seria Etil_Cl 7 93 Accuracy
Run Time:	35.0 Minutes	Channel Name:	Extract 275
Acquired By:	roman_roxana	Proc. Chnl. Descr.:	2998 PDA 275.0 nm (2998

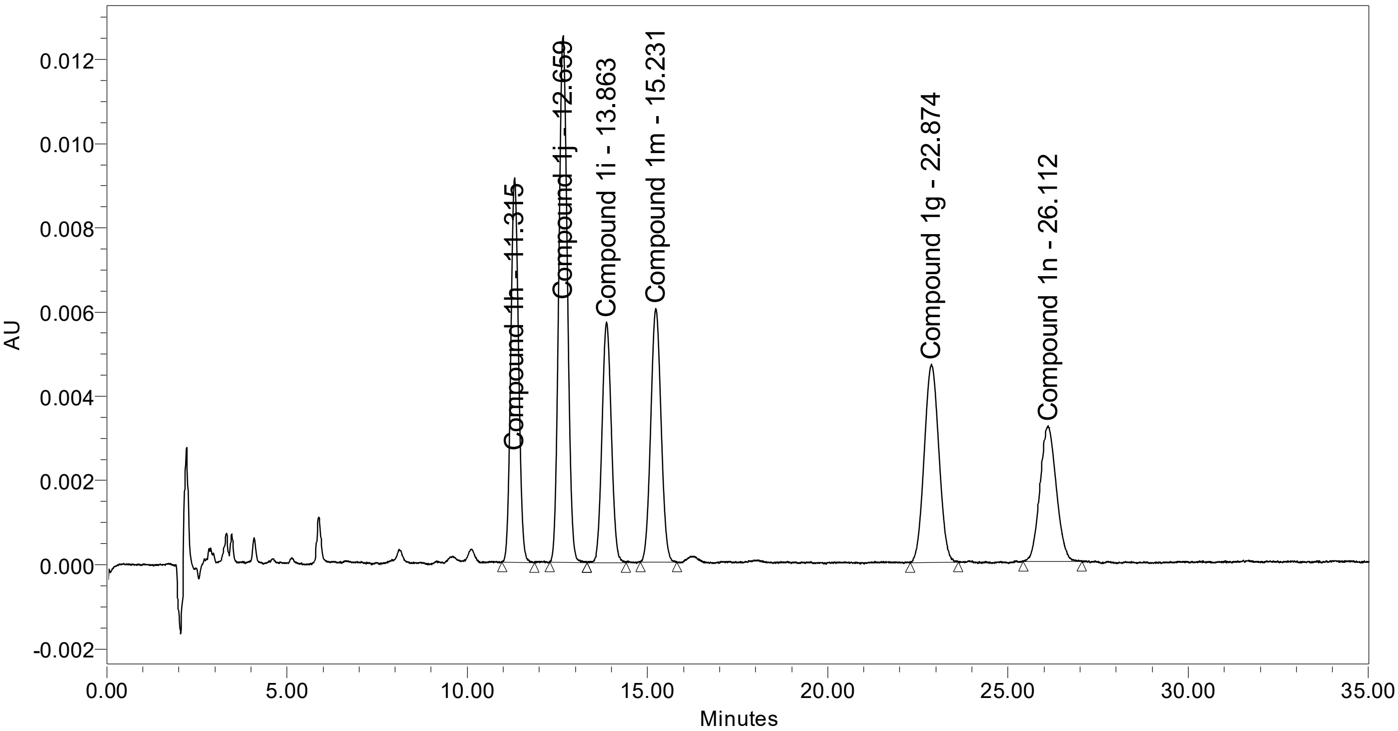

	Peak Name	RT	Area	Height (µV)	Resolution
1	Compound 1h	11.315	134527	9127	
2	Compound 1j	12.659	203088	12505	3.29
3	Compound 1i	13.863	101244	5708	2.68
4	Compound 1m	15.231	118523	6027	2.77
5	Compound 1g	22.874	131484	4707	12.10
6	Compound 1n	26.112	102701	3220	4.08

Chloride_Ethyl_Accuracy

Reported by User: Roman Roxana (roman_roxana)
Acquisition Server Waters-cd3
Project Name: Teste\Thiazides
Sample Set Name: Seria Etil_Cl 7 93 Accuracy
Code column: Inertsil ODS-3 250*4.6 mm 5um LA140

SAMPLE INFORMATION

Sample Name:	Solution 6.0 ug/ mL		
Sample Type:	Unknown	Date Acquired:	2/12/2023 5:26:47 AM EET
Vial:	19	Acq. Method Set:	Seria Etil_Cl 7 93 Accuracy
Injection #:	1	Date Processed:	4/19/2023 3:52:37 PM EEST
Injection Volume:	10.00 ul	Processing Method:	Seria Etil_Cl 7 93 Accuracy
Run Time:	35.0 Minutes	Channel Name:	Extract 275
Acquired By:	roman_roxana	Proc. Chnl. Descr.:	2998 PDA 275.0 nm (2998

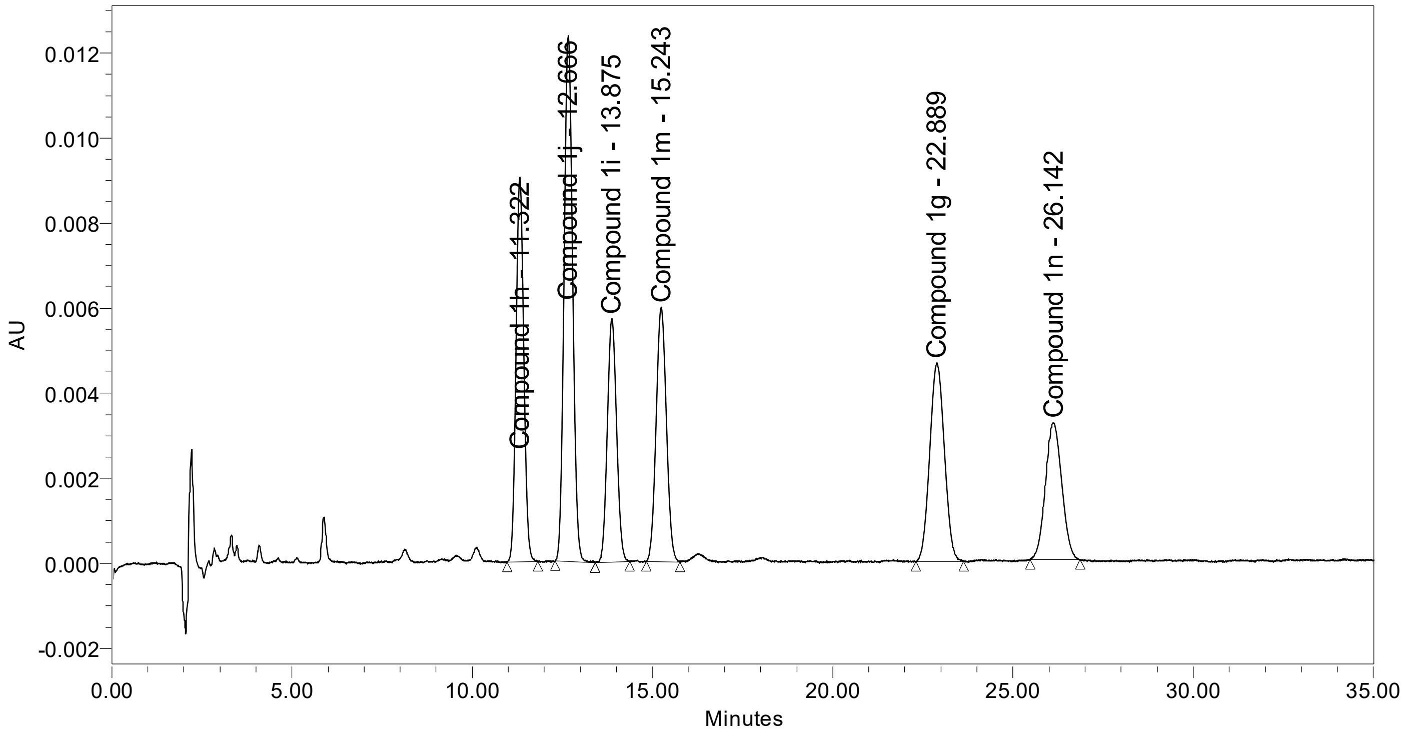

	Peak Name	RT	Area	Height (µV)	Resolution
1	Compound 1h	11.322	133149	9043	
2	Compound 1j	12.666	201432	12369	3.28
3	Compound 1i	13.875	101373	5725	2.69
4	Compound 1m	15.243	116833	5982	2.78
5	Compound 1g	22.889	130301	4663	12.13
6	Compound 1n	26.142	102703	3214	4.08

Quantitative determination of

compounds 1g, 1h, 1i, 1j, 1m, 1n

- Validation of the analytical method -

Validation parameters:

- Specificity
- Precision 1
- Precision 2
- LOD – LOQ
- Linearity
- Range
- Accuracy
- **Robustness**

Quantitative determination of

compounds 1g, 1h, 1i, 1j, 1m, 1n

- Validation of the analytical method -

Robustness

Mobile phase Solvent A: Solvent B (93: 7, V/V)

Chloride_Ethyl_Robustness

Reported by User: Roman Roxana (roman_roxana)
Acquisition Server Waters-cd3
Project Name: Teste\Thiazides
Sample Set Name: Seria Etil_Cl Robustness
Code column: Inertsil ODS-3 250*4.6 mm 5um LA140

SAMPLE INFORMATION

Sample Name:	Solution 5 ug/ mL		
Sample Type:	Standard	Date Acquired:	2/13/2023 11:59:50 AM EET
Vial:	1	Acq. Method Set:	Seria Etil_Cl 7 93
Injection #:	1	Date Processed:	4/10/2023 4:23:38 PM EEST
Injection Volume:	10.00 ul	Processing Method:	Seria Etil_Cl 7 93
Run Time:	35.0 Minutes	Channel Name:	Extract 275
Acquired By:	roman_roxana	Proc. Chnl. Descr.:	2998 PDA 275.0 nm (2998

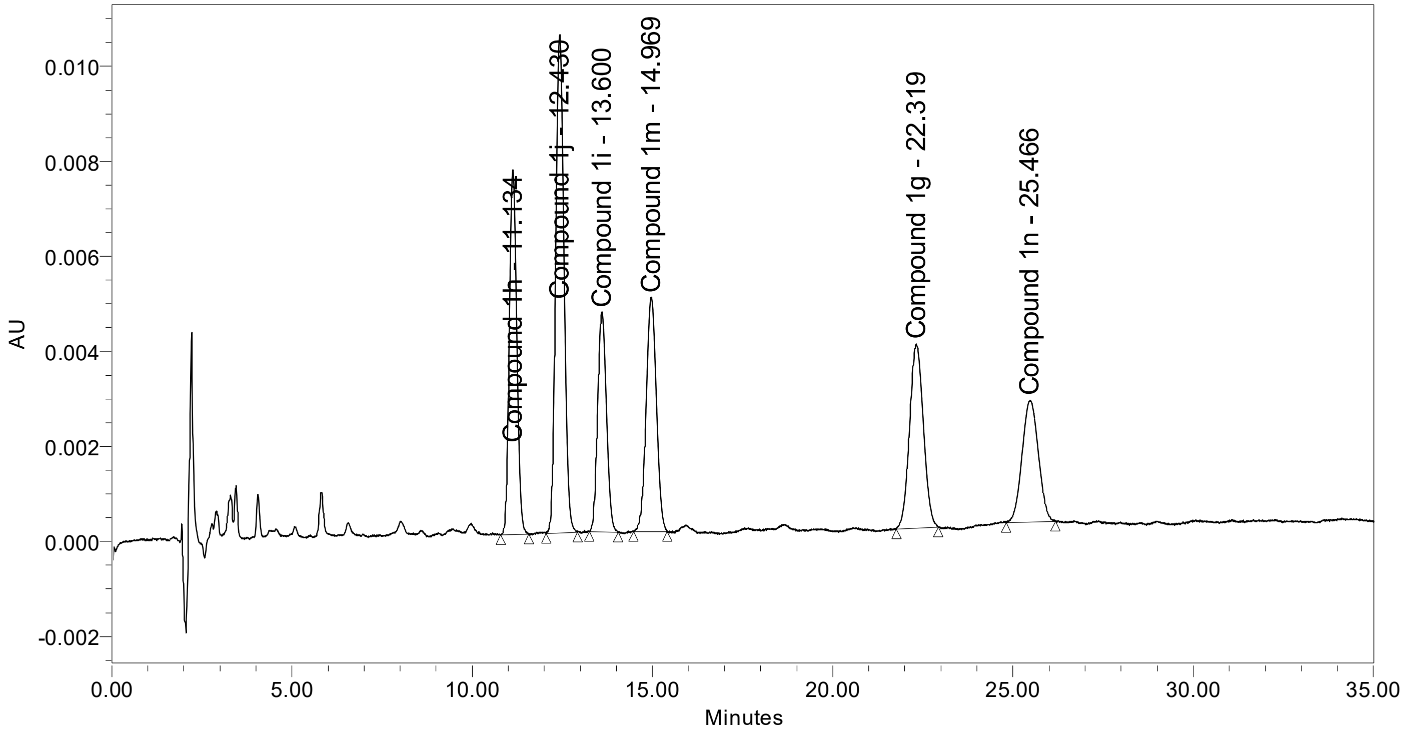

	Peak Name	RT	Area	Height (µV)	Resolution
1	Compound 1h	11.134	110465	7671	
2	Compound 1j	12.430	167189	10488	3.23
3	Compound 1i	13.600	79261	4627	2.67
4	Compound 1m	14.969	96692	4931	2.82
5	Compound 1g	22.319	104951	3874	11.88
6	Compound 1n	25.466	79517	2562	4.05

Quantitative determination of

compounds 1g, 1h, 1i, 1j, 1m, 1n

- Validation of the analytical method -

Robustness

Mobile phase

Solvent A: Solvent B (91: 9, V/V)

Chloride_Ethyl_Robustness

Reported by User: Roman Roxana (roman_roxana)
Acquisition Server Waters-cd3
Project Name: Teste\Thiazides
Sample Set Name: Seria Etil_Cl Robustness
Code column: Inertsil ODS-3 250*4.6 mm 5um LA140

SAMPLE INFORMATION

Sample Name:	Solution 5 ug/ mL		
Sample Type:	Standard	Date Acquired:	2/13/2023 3:15:14 PM EET
Vial:	1	Acq. Method Set:	Seria Etil_Cl 9 91
Injection #:	1	Date Processed:	4/10/2023 4:41:33 PM EEST
Injection Volume:	10.00 ul	Processing Method:	Seria Etil_Cl 9 91
Run Time:	35.0 Minutes	Channel Name:	Extract 275
Acquired By:	roman_roxana	Proc. Chnl. Descr.:	2998 PDA 275.0 nm (2998

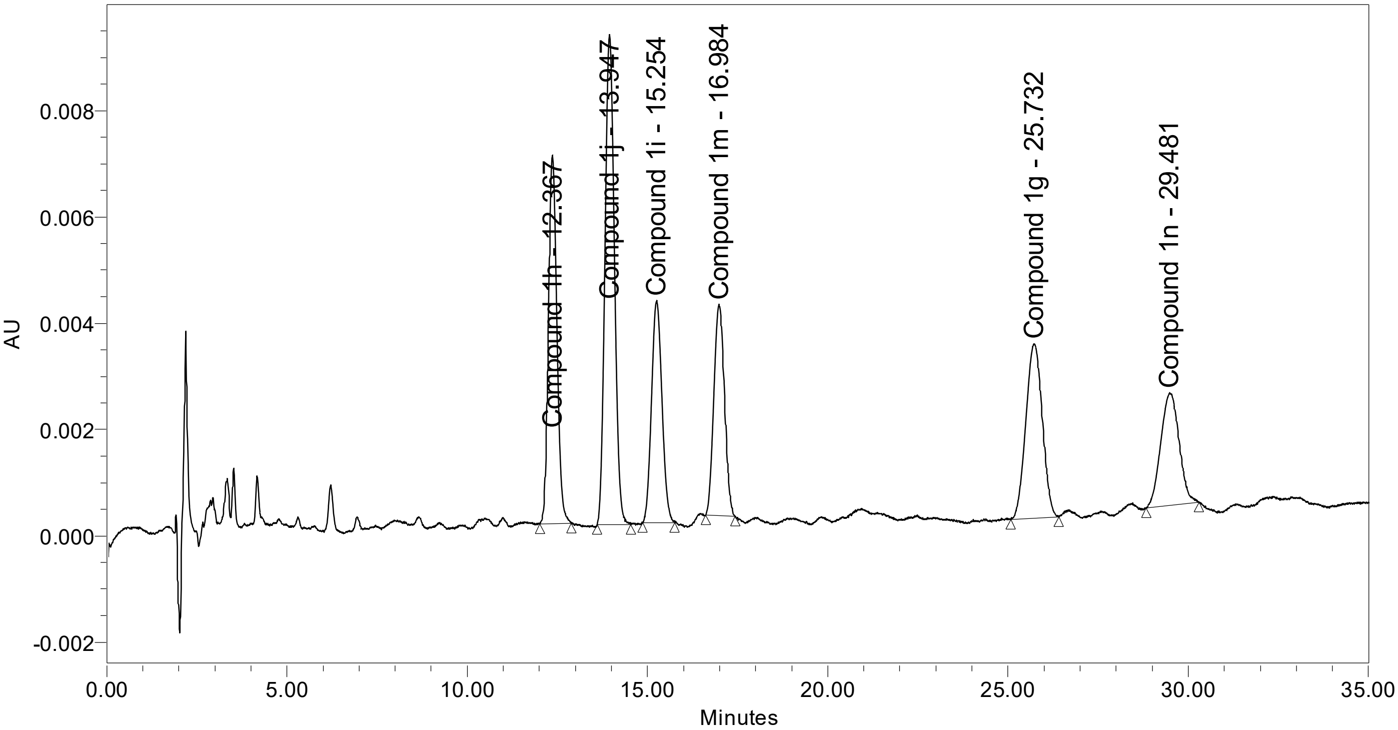

	Peak Name	RT	Area	Height (µV)	Resolution
1	Compound 1h	12.367	113647	6929	
2	Compound 1j	13.947	164894	9216	3.49
3	Compound 1i	15.254	83100	4182	2.60
4	Compound 1m	16.984	82081	3977	3.18
5	Compound 1g	25.732	104489	3277	12.37

	Peak Name	RT	Area	Height (µV)	Resolution
6	Compound 1n	29.481	75218	2123	4.16

Quantitative determination of

compounds 1g, 1h, 1i, 1j, 1m, 1n

- Validation of the analytical method -

Robustness

Mobile phase

Solvent A: Solvent B (95: 5, V/V)

Chloride_Ethyl_Robustness

Reported by User: Roman Roxana (roman_roxana)
Acquisition Server Waters-cd3
Project Name: Teste\Thiazides
Sample Set Name: Seria Etil_Cl Robustness
Code column: Inertsil ODS-3 250*4.6 mm 5um LA140

SAMPLE INFORMATION

Sample Name:	Solution 5 ug/ mL		
Sample Type:	Standard	Date Acquired:	2/13/2023 11:14:02 AM EET
Vial:	1	Acq. Method Set:	Seria Etil_Cl 5 95
Injection #:	1	Date Processed:	4/10/2023 4:17:20 PM EEST
Injection Volume:	10.00 ul	Processing Method:	Seria Etil_Cl 5 95
Run Time:	35.0 Minutes	Channel Name:	Extract 275
Acquired By:	roman_roxana	Proc. Chnl. Descr.:	2998 PDA 275.0 nm (2998

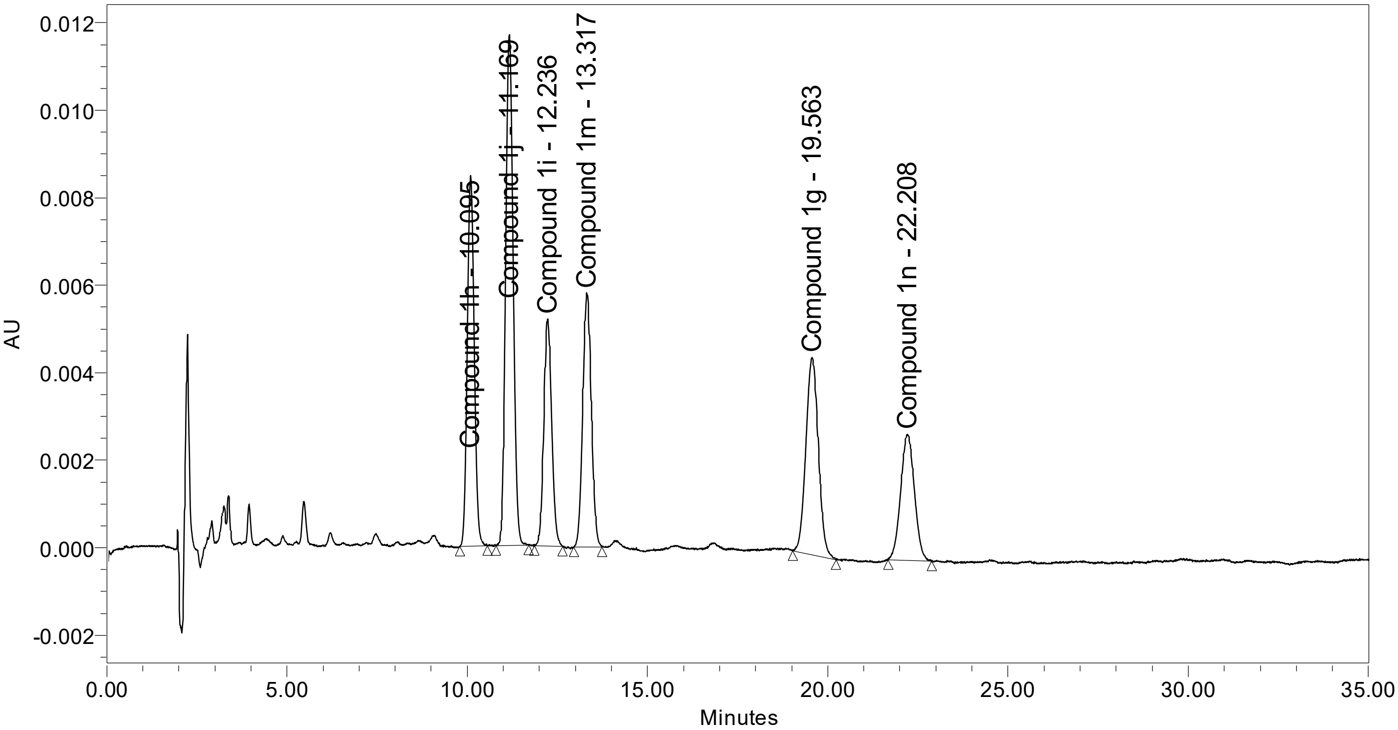

	Peak Name	RT	Area	Height (µV)	Resolution
1	Compound 1h	10.095	111974	8478	
2	Compound 1j	11.169	167336	11672	2.96
3	Compound 1i	12.236	81377	5197	2.69
4	Compound 1m	13.317	98448	5805	2.50
5	Compound 1g	19.563	110102	4512	11.42

	Peak Name	RT	Area	Height (µV)	Resolution
6	Compound 1n	22.208	77931	2888	3.87

Quantitative determination of

compounds 1g, 1h, 1i, 1j, 1m, 1n

- Validation of the analytical method -

Robustness

Flow 0.8 mL/ min.

Chloride_Ethyl_Robustness

Reported by User: Roman Roxana (roman_roxana)
Acquisition Server Waters-cd3
Project Name: Teste\Thiazides
Sample Set Name: Seria Etil_Cl Robustness
Code column: Inertsil ODS-3 250*4.6 mm 5um LA140

S A M P L E   I N F O R M A T I O N

Sample Name:	Solution 5 ug/ mL		
Sample Type:	Standard	Date Acquired:	2/13/2023 12:46:09 PM EET
Vial:	1	Acq. Method Set:	Seria Etil_Cl 7 93 flow 0 8
Injection #:	1	Date Processed:	4/10/2023 4:28:50 PM EEST
Injection Volume:	10.00 ul	Processing Method:	Seria Etil_Cl 7 93 flow 0 8
Run Time:	35.0 Minutes	Channel Name:	Extract 275
Acquired By:	roman_roxana	Proc. Chnl. Descr.:	2998 PDA 275.0 nm (2998

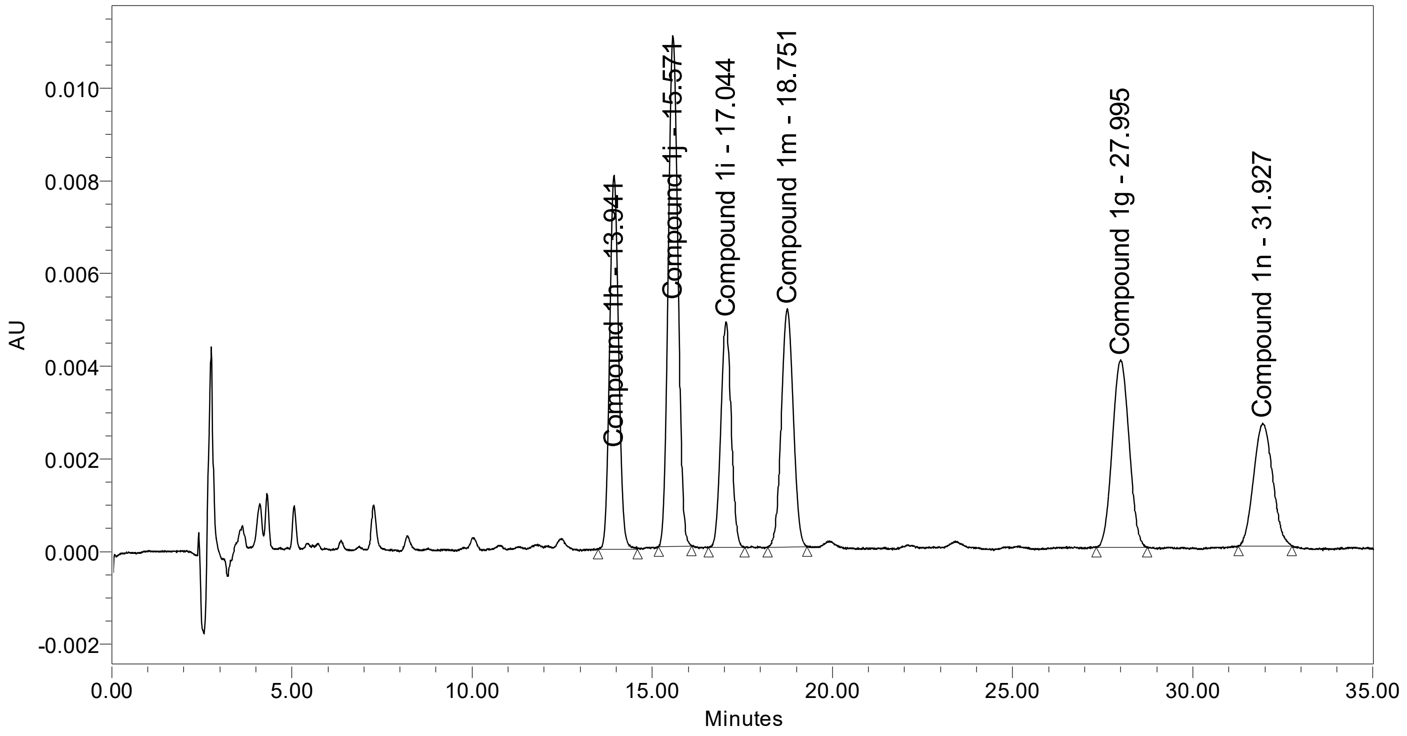

	Peak Name	RT	Area	Height (µV)	Resolution
1	Compound 1h	13.941	137904	8070	
2	Compound 1j	15.571	205283	11020	3.46
3	Compound 1i	17.044	98551	4869	2.85
4	Compound 1m	18.751	118429	5145	2.99
5	Compound 1g	27.995	129613	4049	12.66

	Peak Name	RT	Area	Height (µV)	Resolution
6	Compound 1n	31.927	97198	2656	4.28

Quantitative determination of

compounds 1g, 1h, 1i, 1j, 1m, 1n

- Validation of the analytical method -

Robustness

Flow 1.2 mL/ min.

Chloride_Ethyl_Robustness

Reported by User: Roman Roxana (roman_roxana)
Acquisition Server Waters-cd3
Project Name: Teste\Thiazides
Sample Set Name: Seria Etil_Cl Robustness
Code column: Inertsil ODS-3 250*4.6 mm 5um LA140

SAMPLE INFORMATION

Sample Name:	Solution 5 ug/ mL		
Sample Type:	Standard	Date Acquired:	2/13/2023 1:32:00 PM EET
Vial:	1	Acq. Method Set:	Seria Etil_Cl 7 93 flow 1 2
Injection #:	1	Date Processed:	4/10/2023 4:34:26 PM EEST
Injection Volume:	10.00 ul	Processing Method:	Seria Etil_Cl 7 93 flow 1 2
Run Time:	35.0 Minutes	Channel Name:	Extract 275
Acquired By:	roman_roxana	Proc. Chnl. Descr.:	2998 PDA 275.0 nm (2998

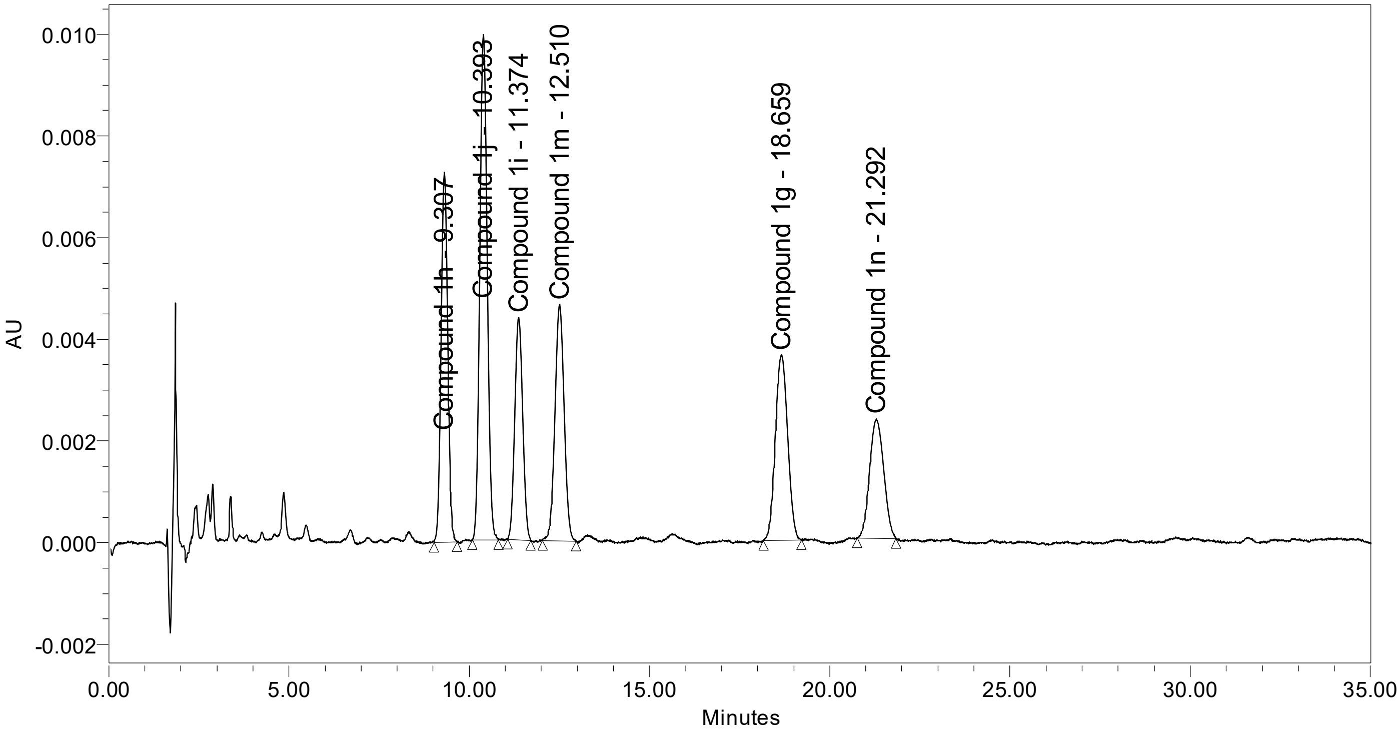

	Peak Name	RT	Area	Height (µV)	Resolution
1	Compound 1h	9.307	92549	7268	
2	Compound 1j	10.393	139910	9940	3.06
3	Compound 1i	11.374	66095	4375	2.53
4	Compound 1m	12.510	81728	4651	2.64
5	Compound 1g	18.659	86930	3646	11.21
6	Compound 1n	21.292	64028	2355	3.83

Quantitative determination of

compounds 1g, 1h, 1i, 1j, 1m, 1n

- Validation of the analytical method -

Robustness

Column temperature: 35 ºC

Chloride_Ethyl_Robustness

Reported by User: Roman Roxana (roman_roxana)
Acquisition Server Waters-cd3
Project Name: Teste\Thiazides
Sample Set Name: Seria Etil_Cl Robustness
Code column: Inertsil ODS-3 250*4.6 mm 5um LA140

SAMPLE INFORMATION

Sample Name:	Solution 5 ug/ mL		
Sample Type:	Unknown	Date Acquired:	2/13/2023 2:27:48 PM EET
Vial:	1	Acq. Method Set:	Seria Etil_Cl 7 93 temperature
Injection #:	1	Date Processed:	4/10/2023 4:38:28 PM EEST
Injection Volume:	10.00 ul	Processing Method:	Seria Etil_Cl 7 93 temperature
Run Time:	35.0 Minutes	Channel Name:	Extract 275
Acquired By:	roman_roxana	Proc. Chnl. Descr.:	2998 PDA 275.0 nm (2998

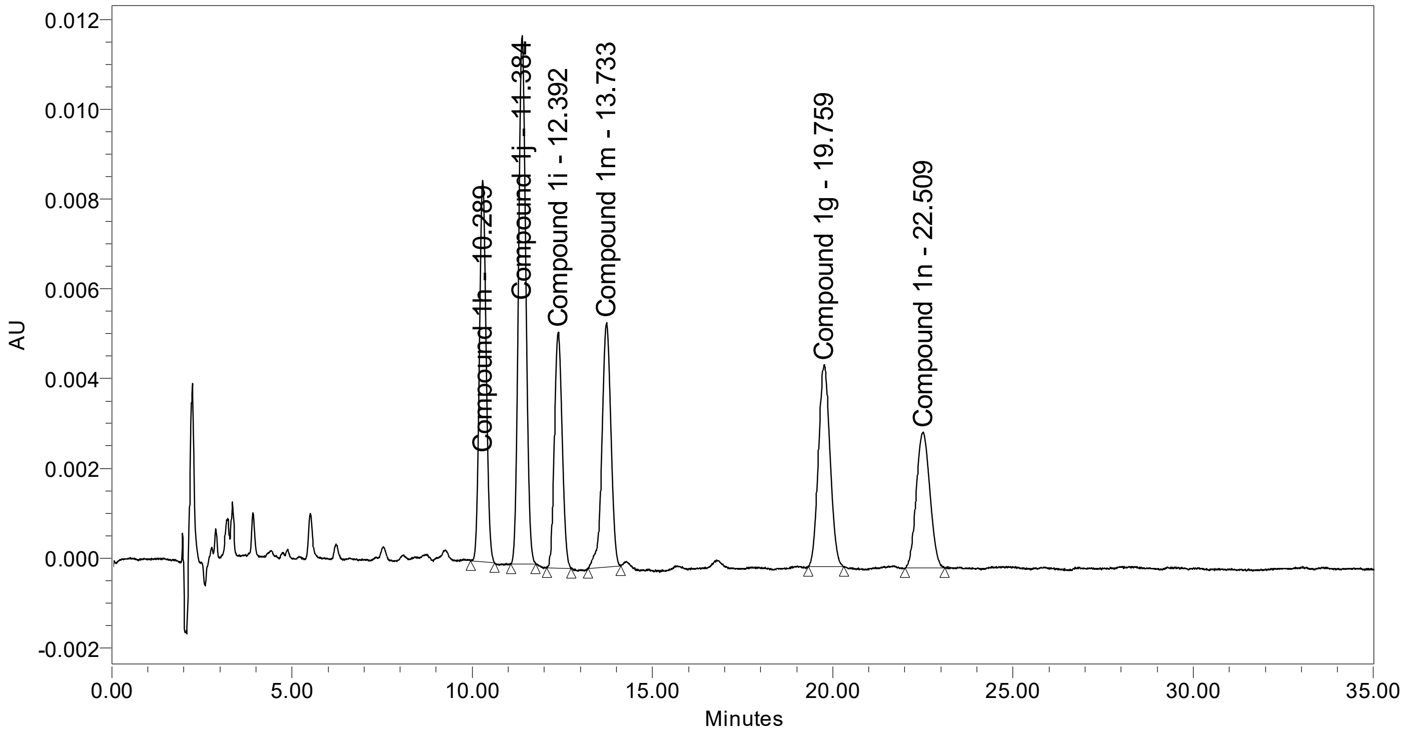

	Peak Name	RT	Area	Height (µV)	Resolution
1	Compound 1h	10.289	108303	8504	
2	Compound 1j	11.384	163318	11767	3.10
3	Compound 1i	12.392	78388	5255	2.63
4	Compound 1m	13.733	93360	5447	3.20
5	Compound 1g	19.759	102791	4493	11.42
6	Compound 1n	22.509	79335	3021	4.19

^{1}H-NMR and ^{13}C-NMR spectra for the compounds 1a – 1o

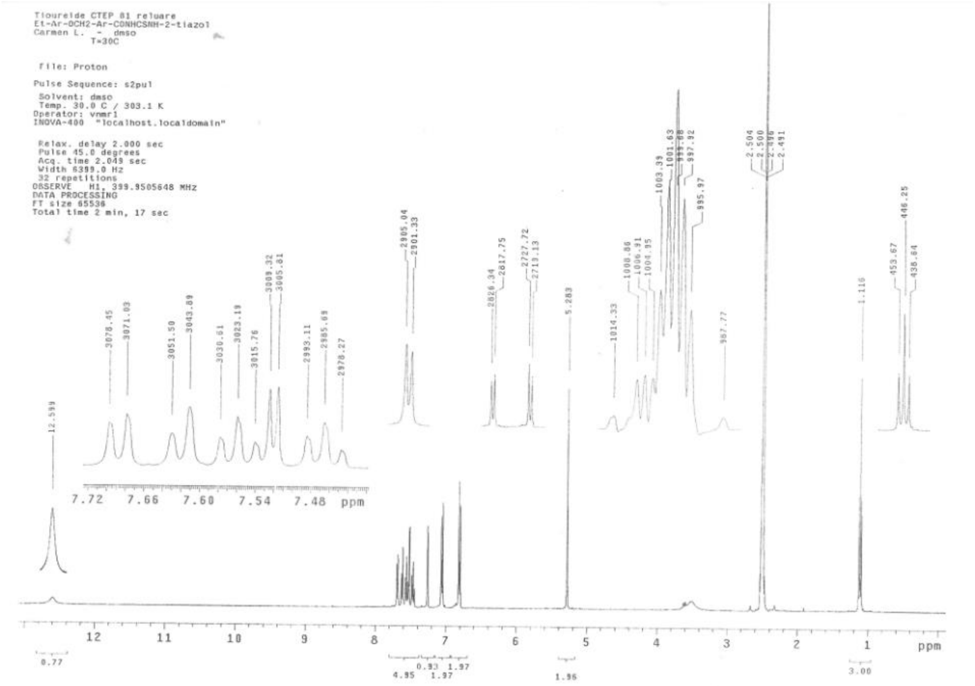

Figure S14. **^{1}H-NMR** for the compound **1a**

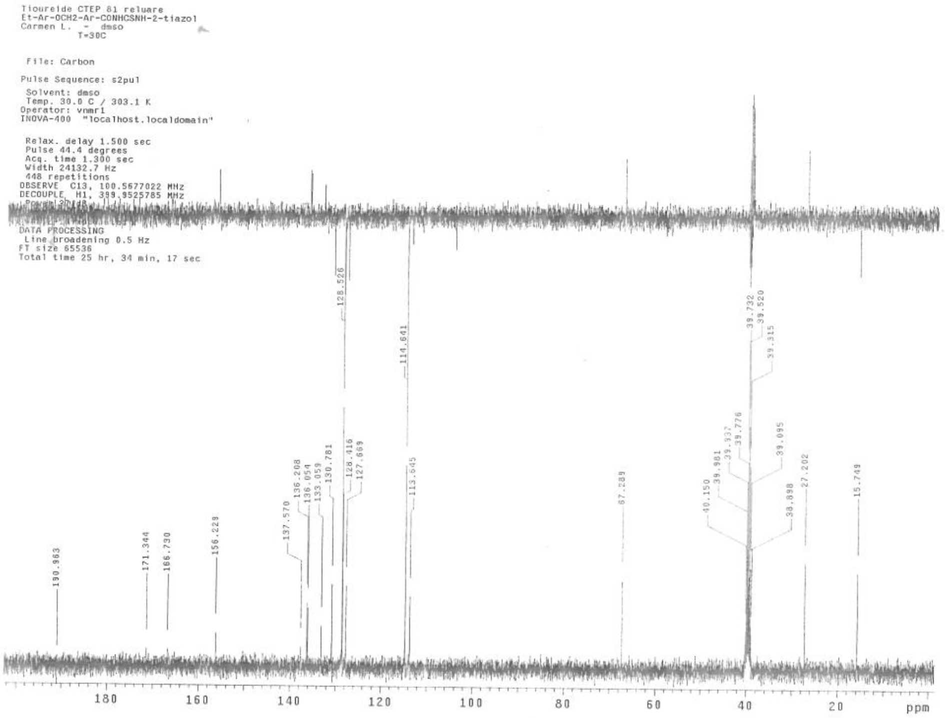

Figure S15. **^{13}C-NMR** for the compound **1a**

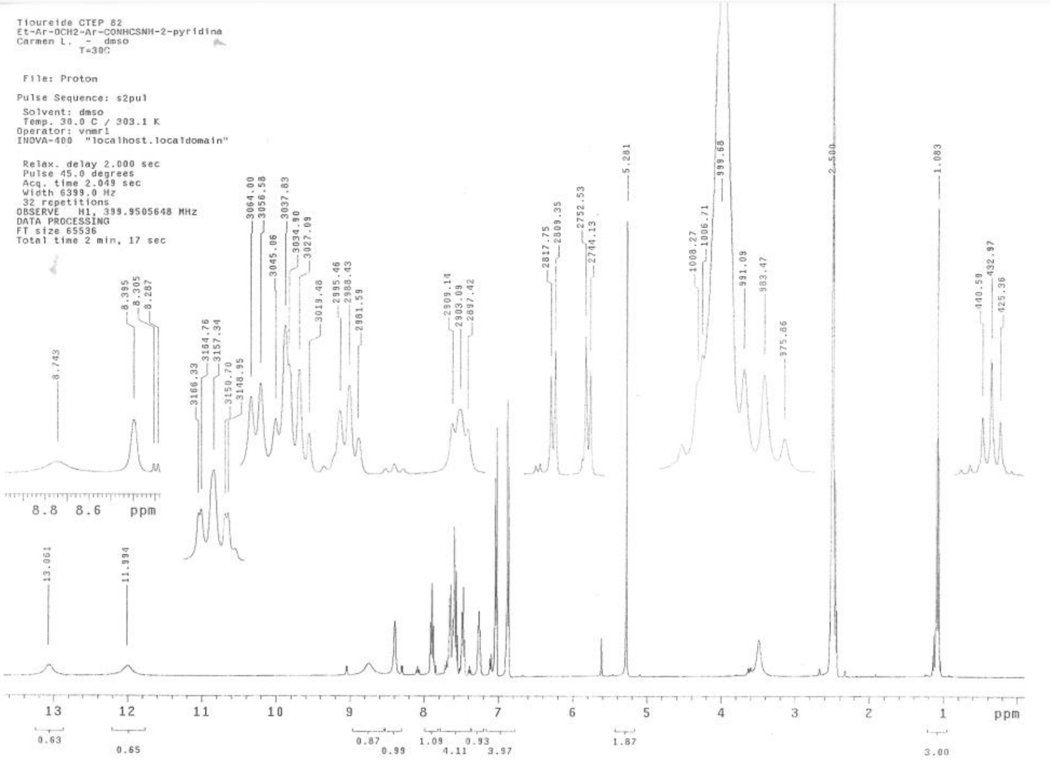

Figure S16. **^{1}H-NMR** for the compound **1b**

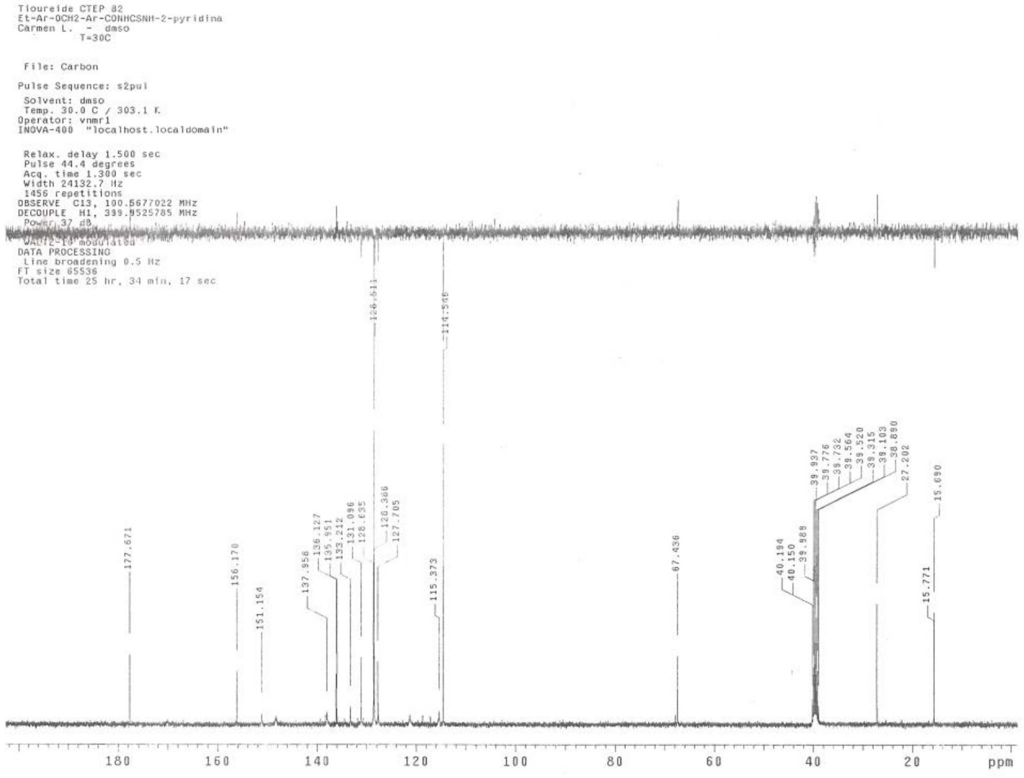

Figure S17. **^{13}C-NMR** for the compound **1b**

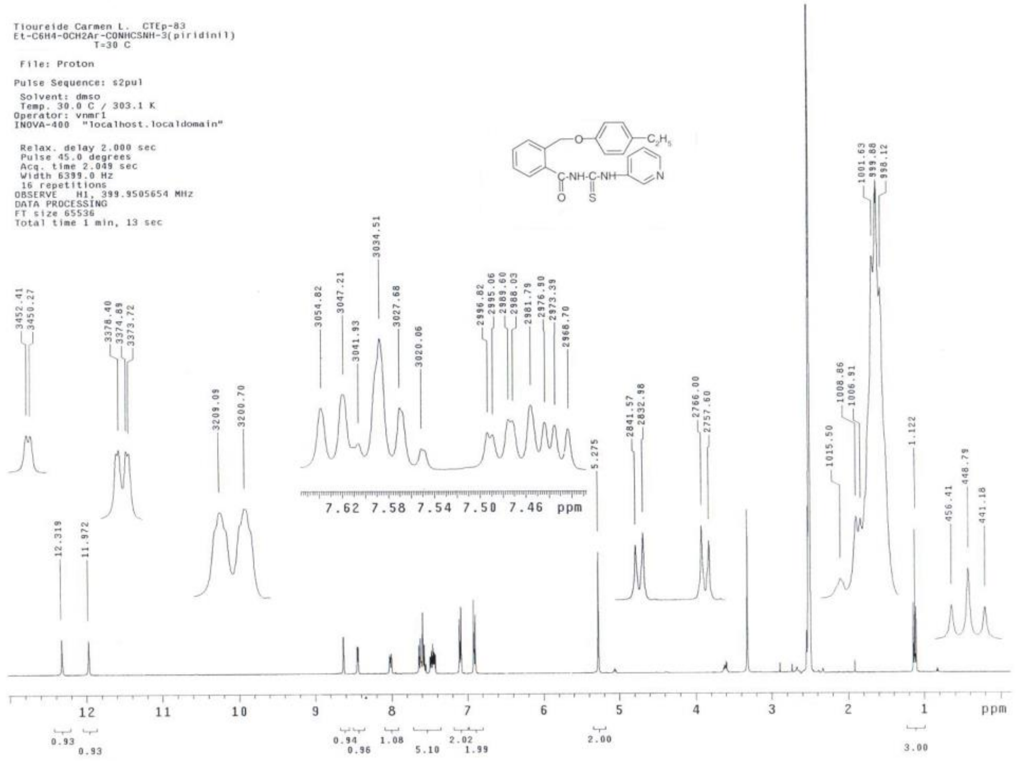

Figure S18. **^{1}H- NMR** for the compound **1c**

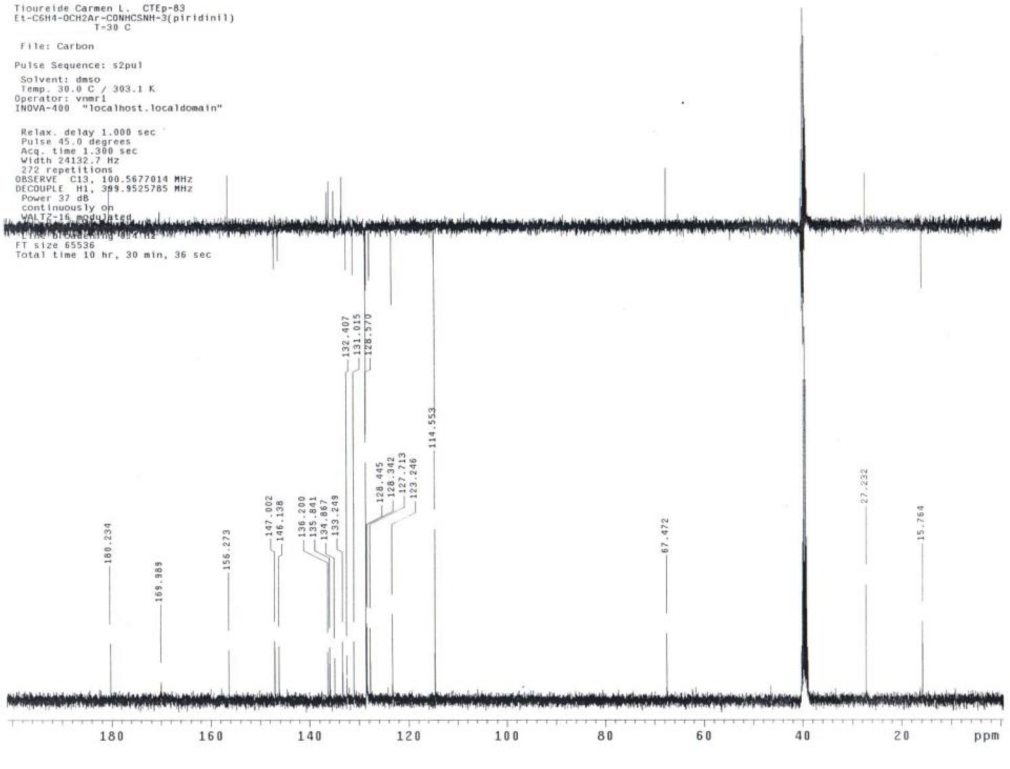

Figure S19. **^{13}C- NMR** for the compound **1c**

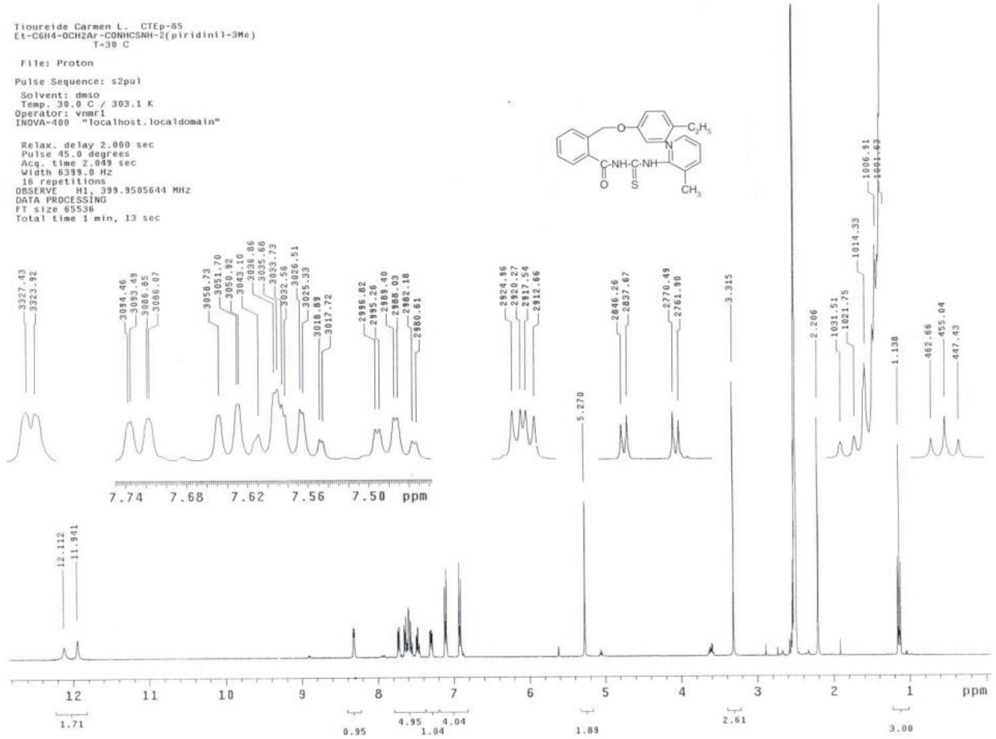

Figure S20. **^{1}H- NMR** for the compound **1d**

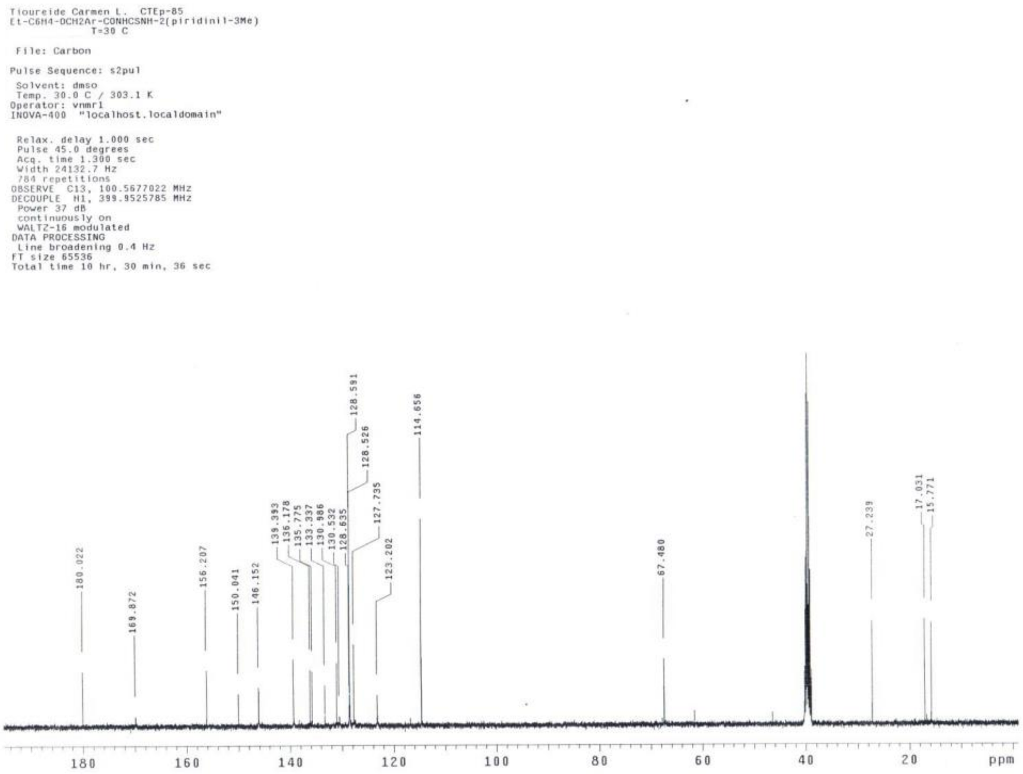

Figure S21. **^{13}C- NMR** for the compound **1d**

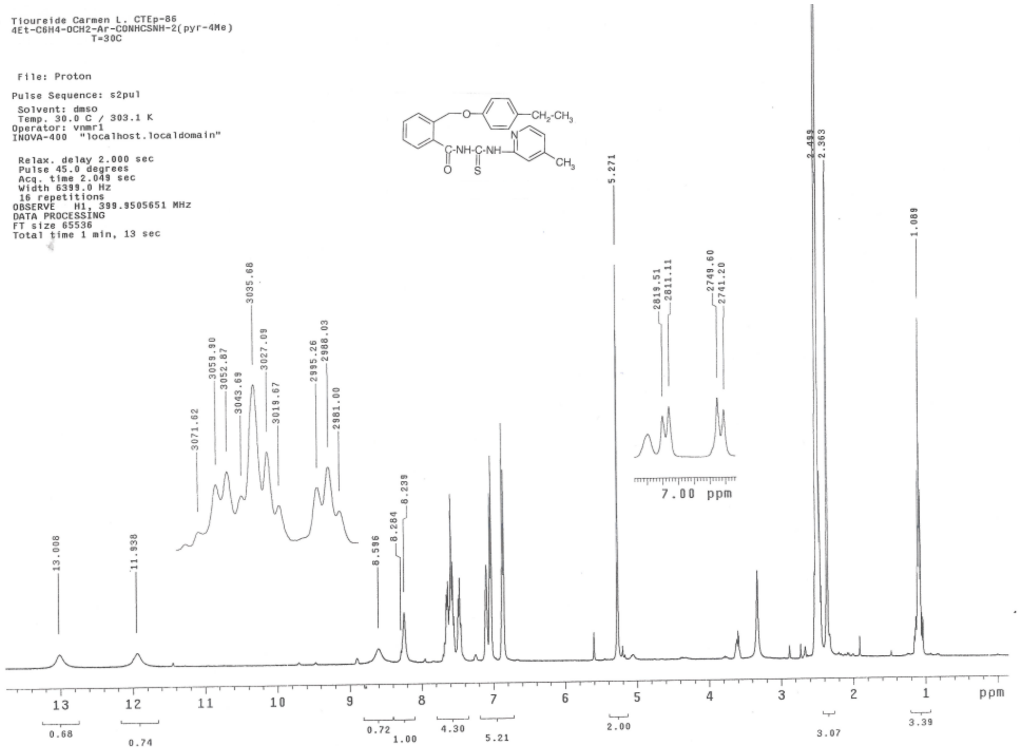

Figure S22. [1]**H- NMR** for the compound **1e**

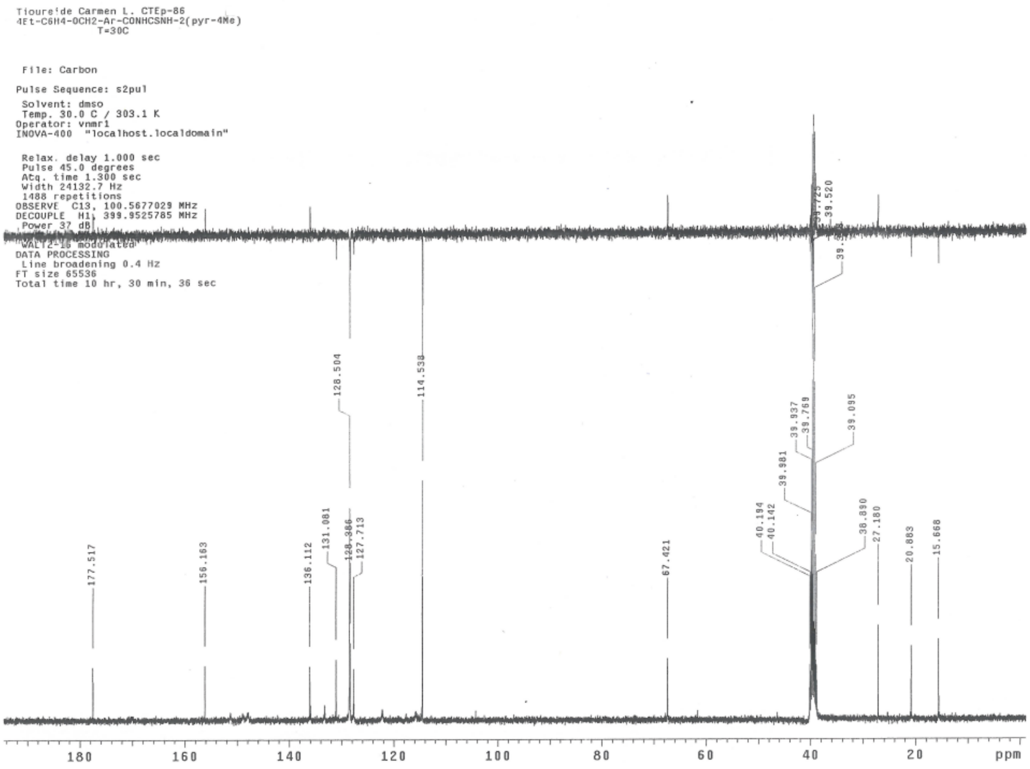

Figure S23. [13]**C- NMR** for the compound **1e**

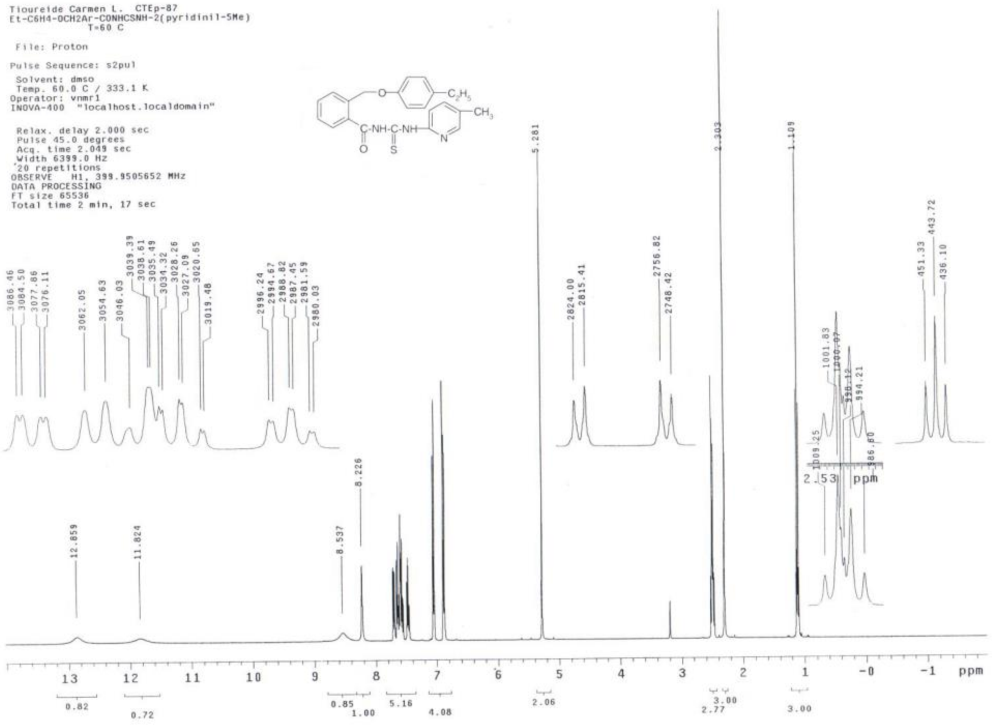

Figure S24. **^{1}H- NMR** for the compound **1f**

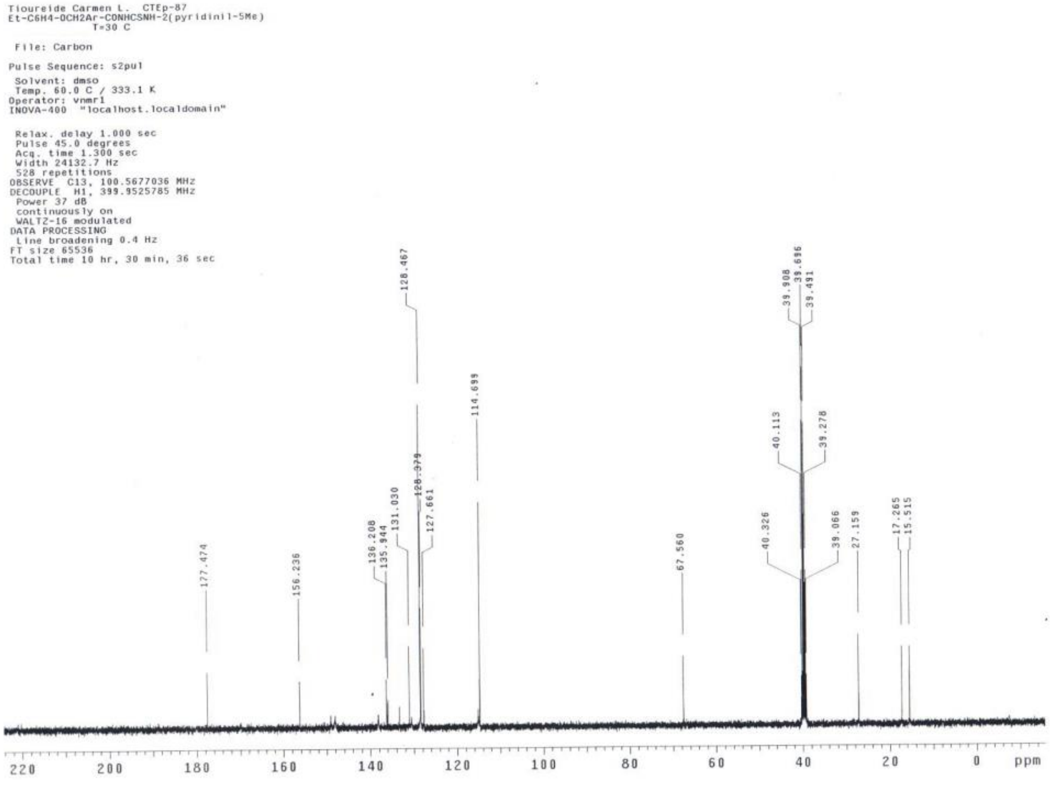

Figure S25. **^{13}C- NMR** for the compound **1f**

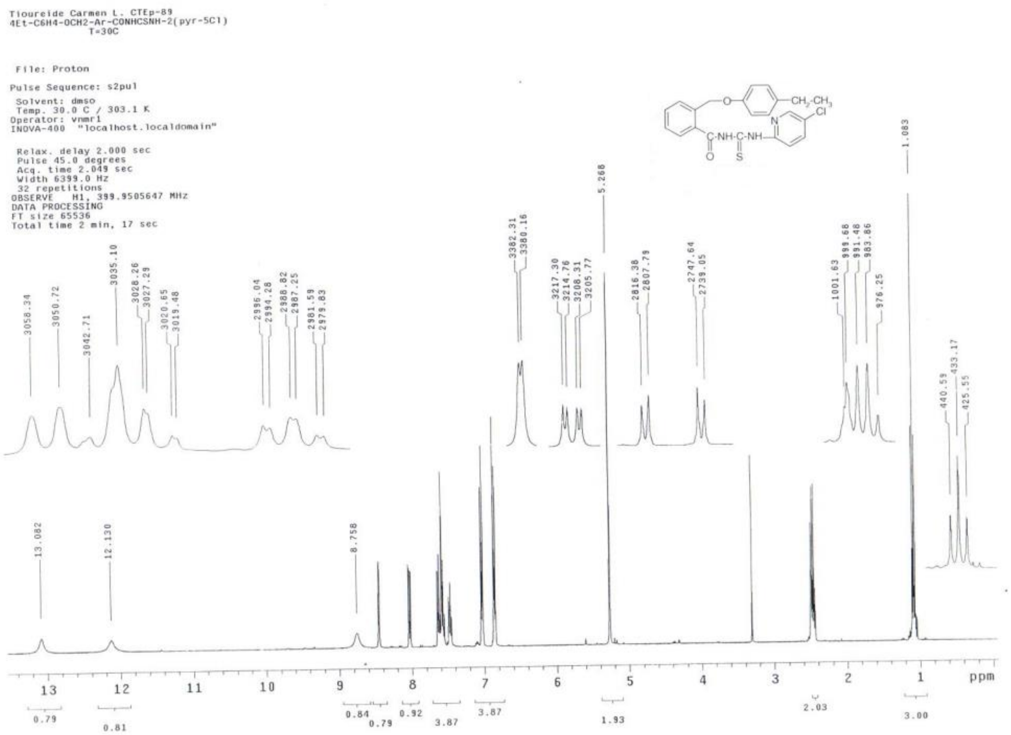

Figure S26. **^{1}H- NMR** for the compound **1g**

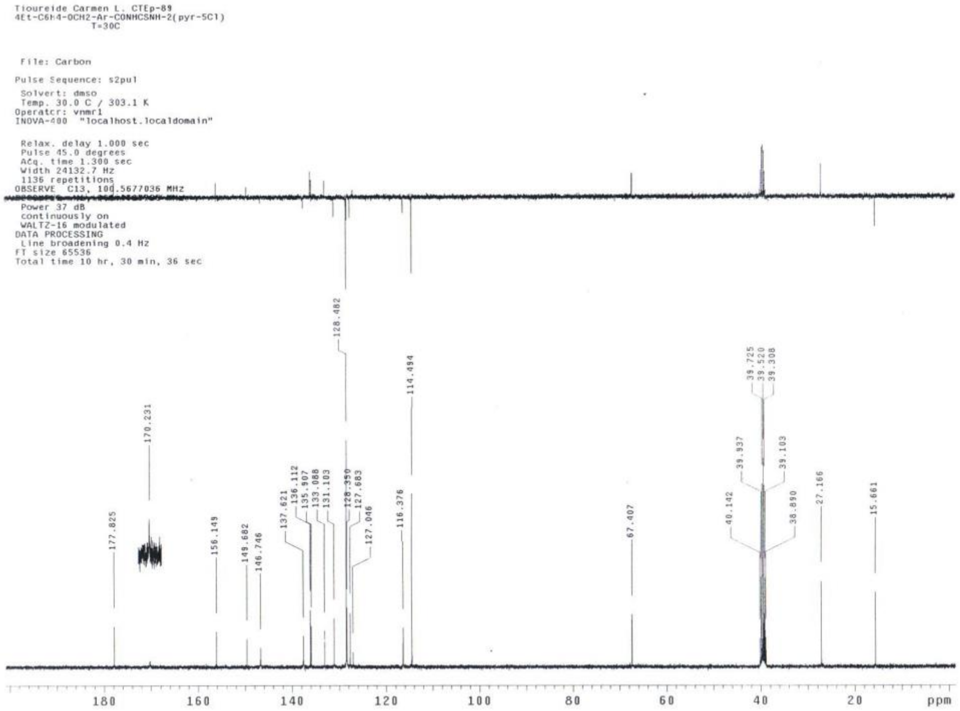

Figure S27. **^{13}C- NMR** for the compound **1g**

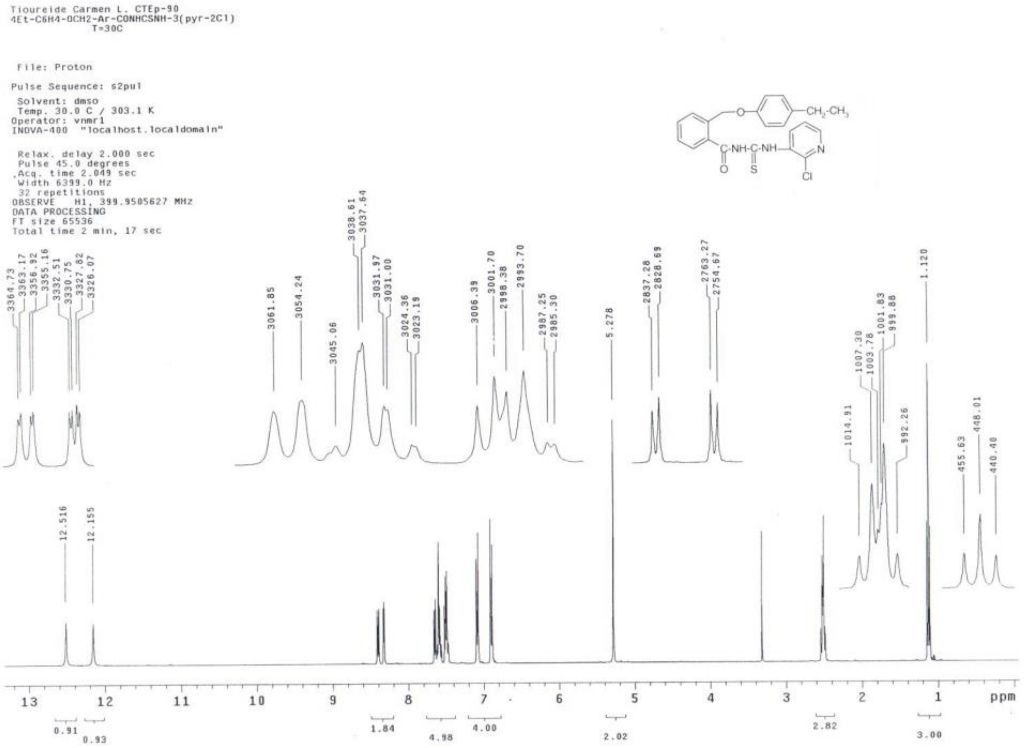

Figure S28. **^{1}H- NMR** for the compound **1h**

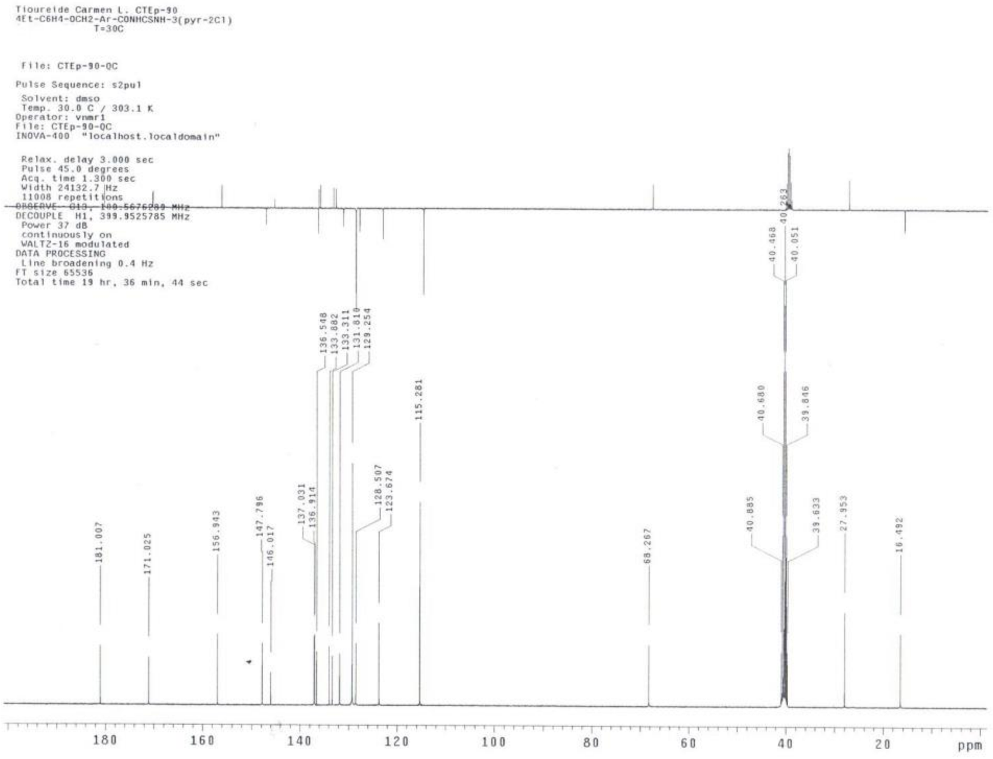

Figure S29. **^{13}C- NMR** for the compound **1h**

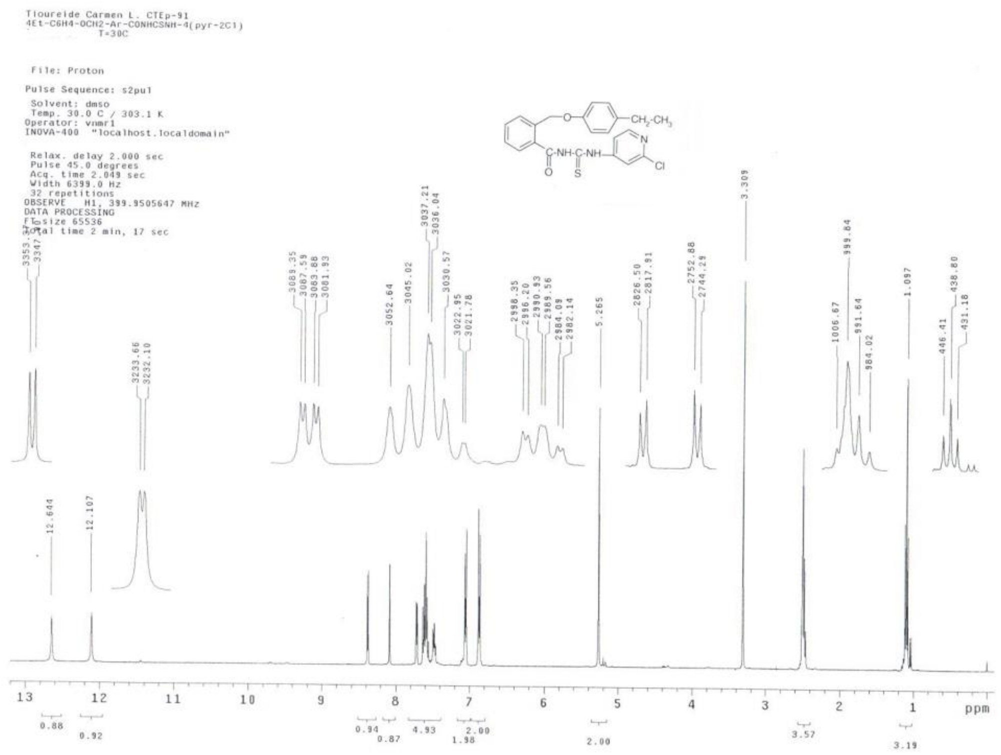

Figure S30. **^{1}H- NMR** for the compound **1i**

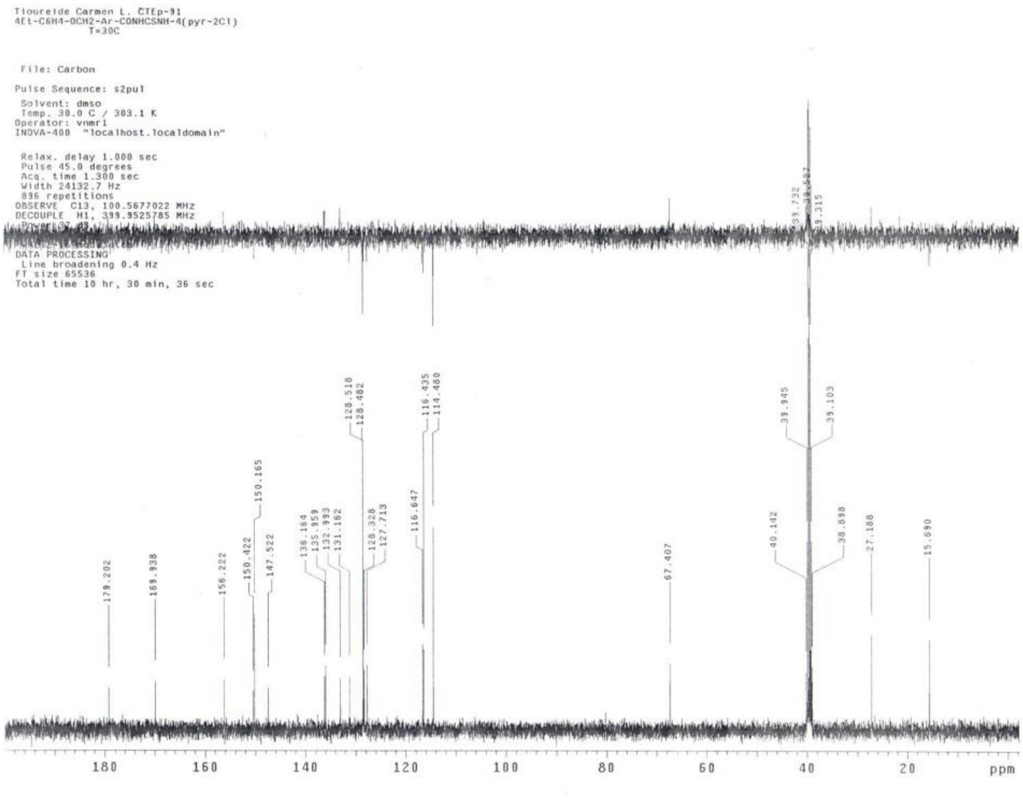

Figure S31. **^{13}C- NMR** for the compound **1i**

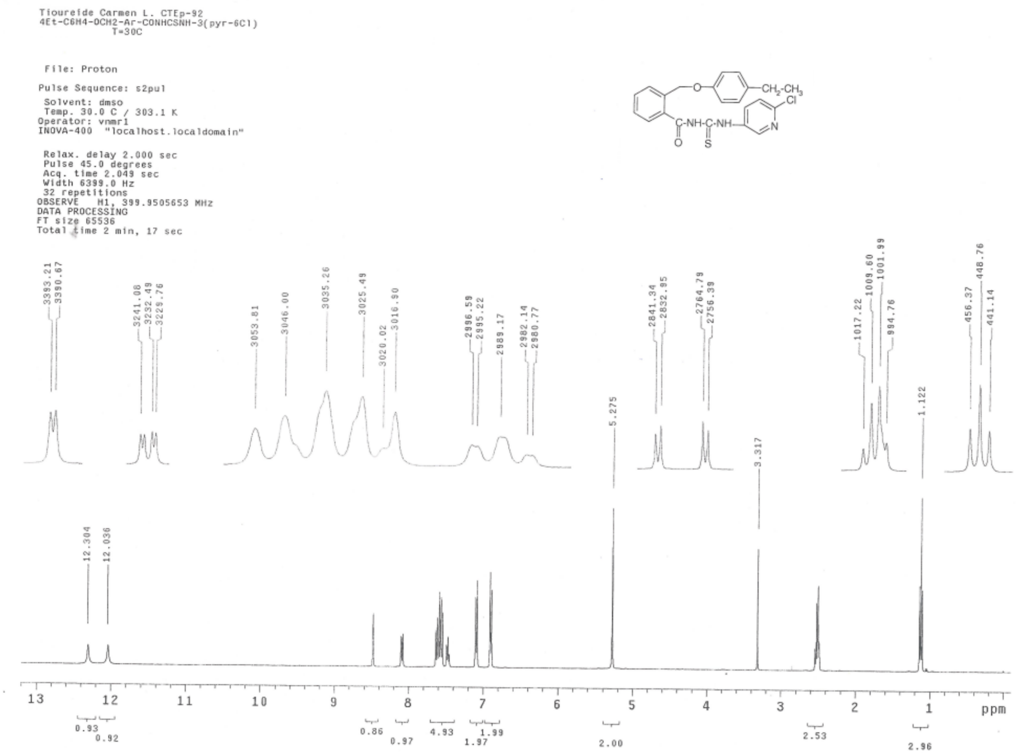

Figure S32. **^{1}H- NMR** for the compound **1j**

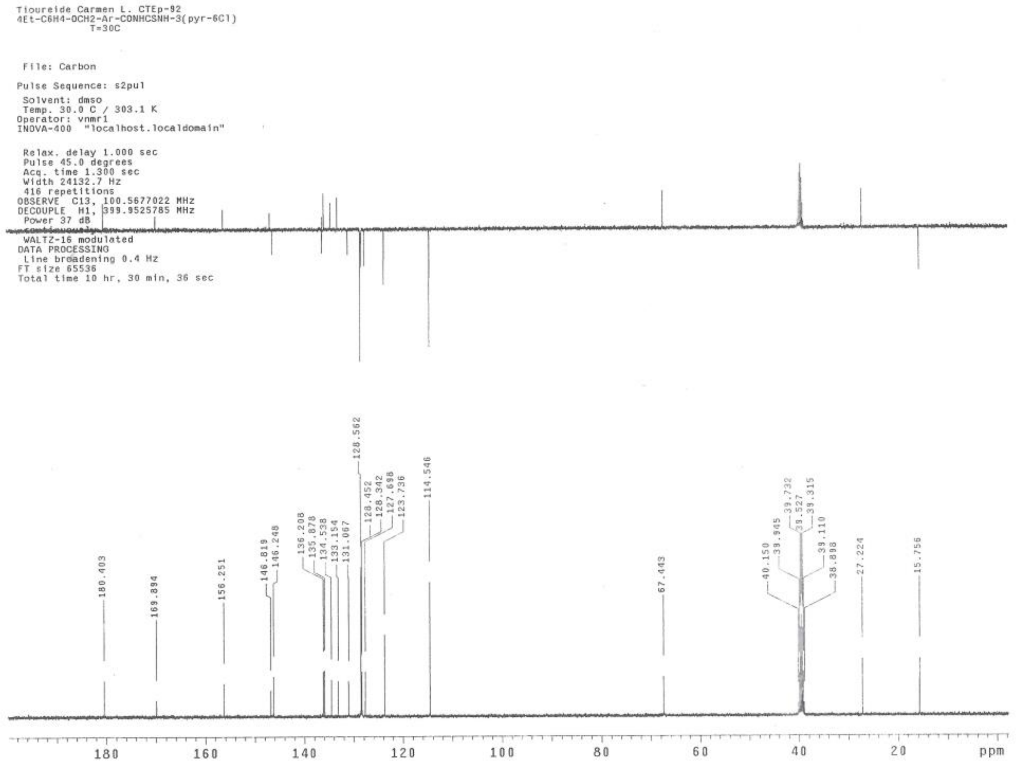

Figure S33. **^{13}C- NMR** for the compound **1j**

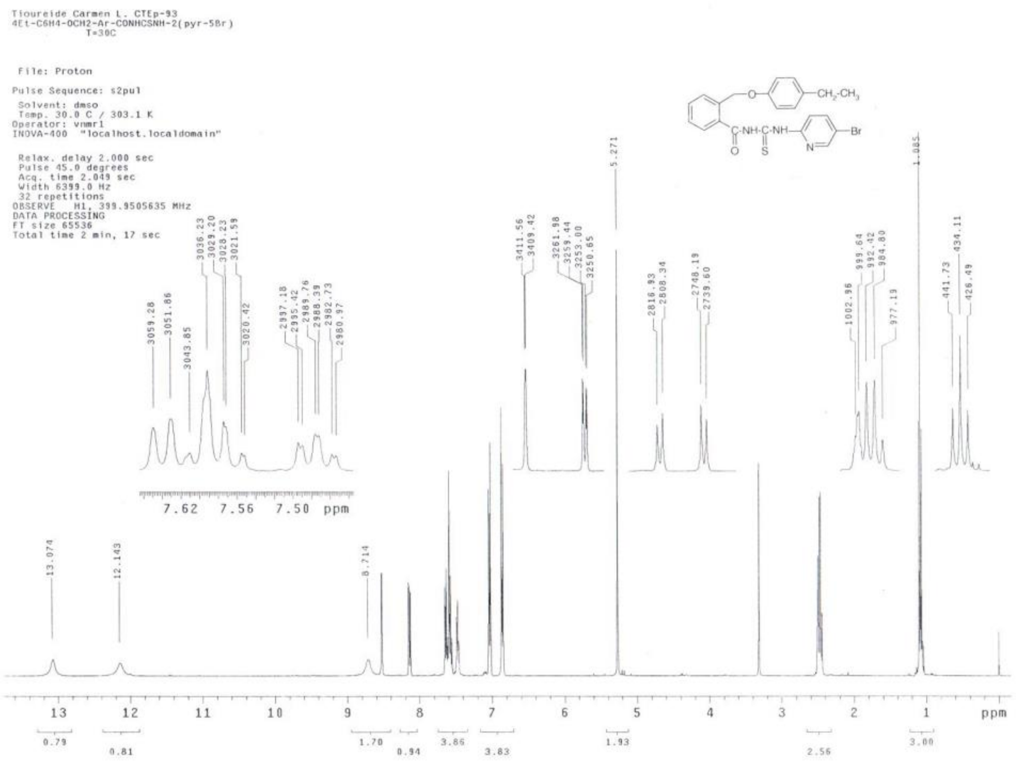

Figure S34. **^{1}H- NMR** for the compound **1k**

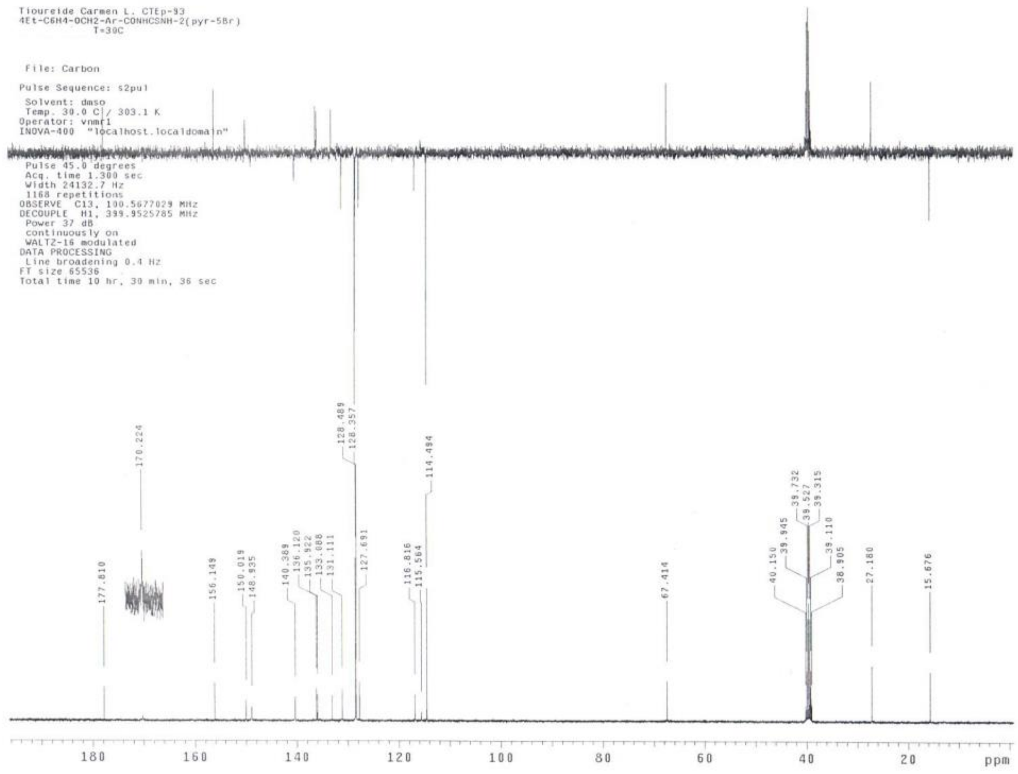

Figure S35. **^{13}C- NMR** for the compound **1k**

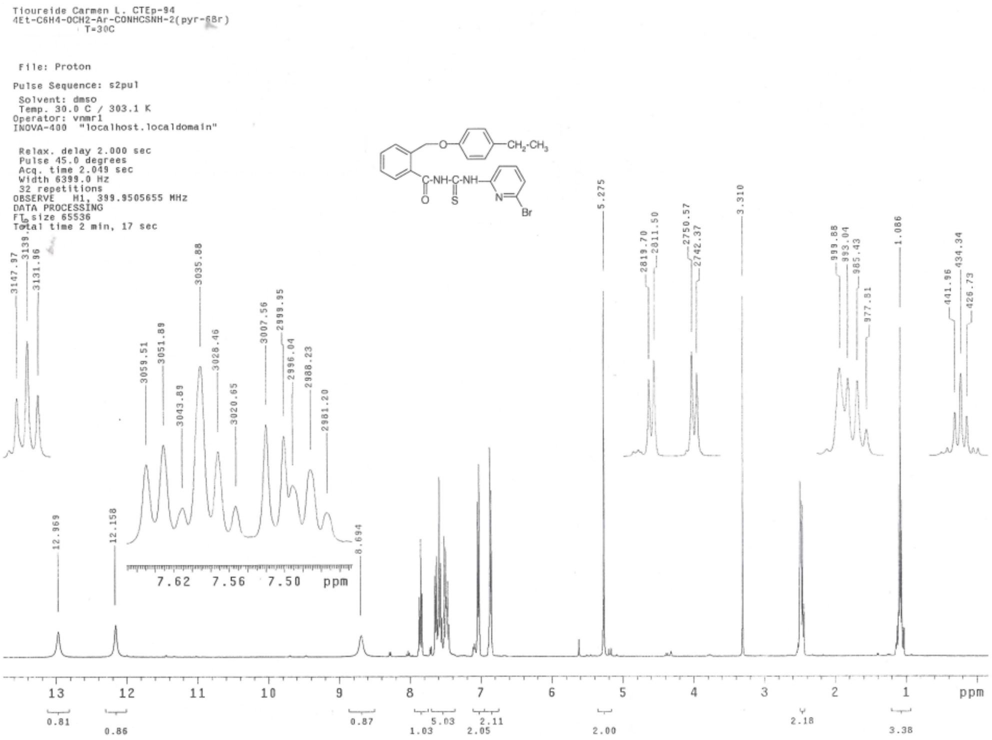

Figure S36. [1]**H- NMR** for the compound **1l**

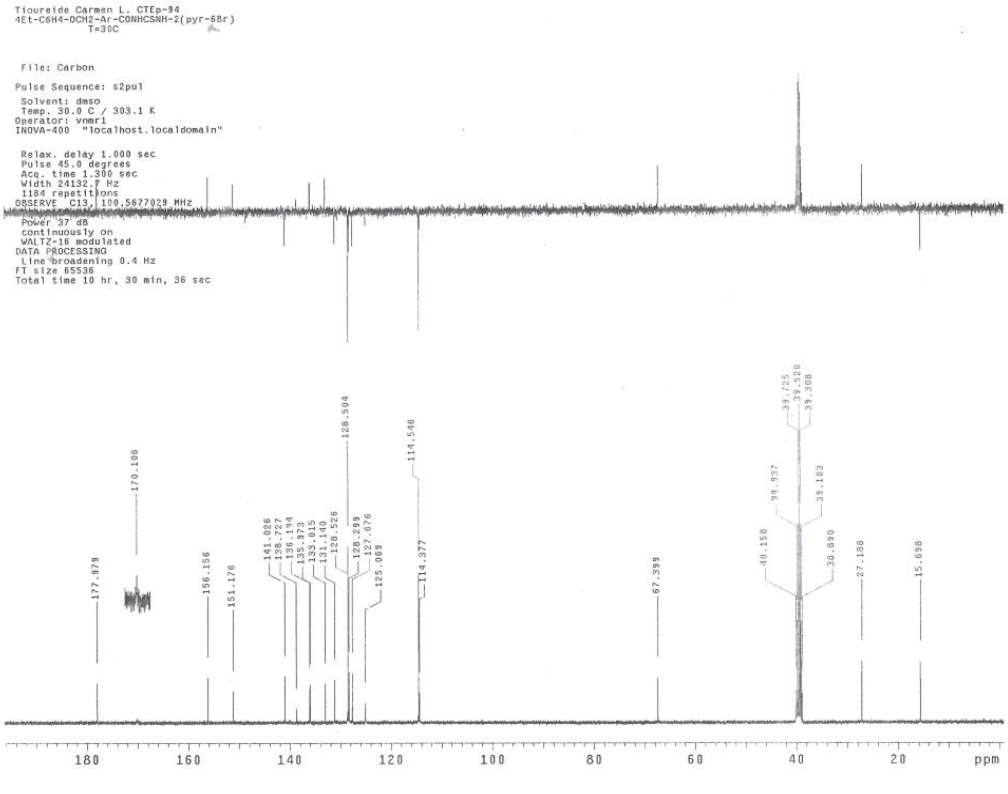

Figure S37. [13]**C- NMR** for the compound **1l**

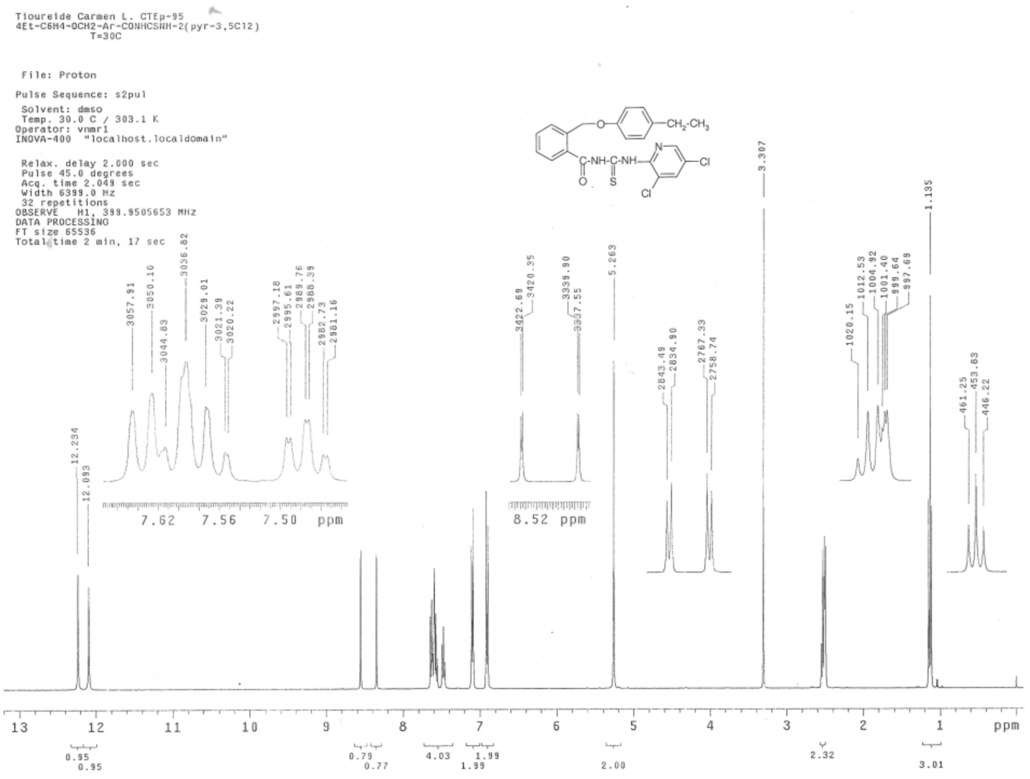

Figure S38. **^{1}H- NMR** for the compound **1m**

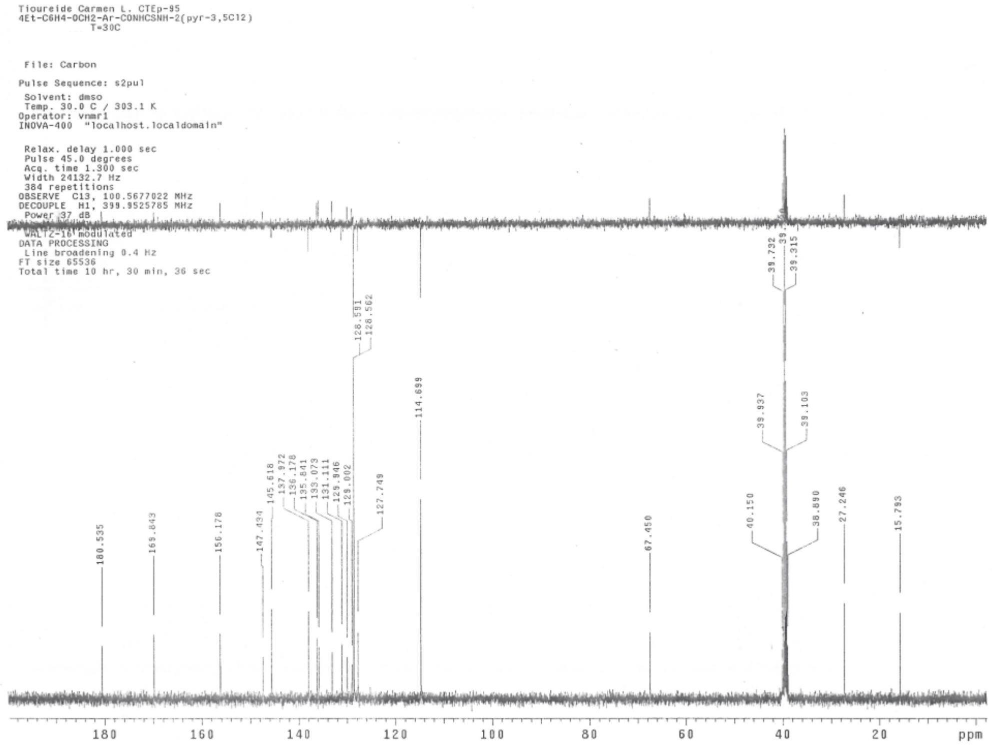

Figure S39. **^{13}C-NMR** for the compound **1m**

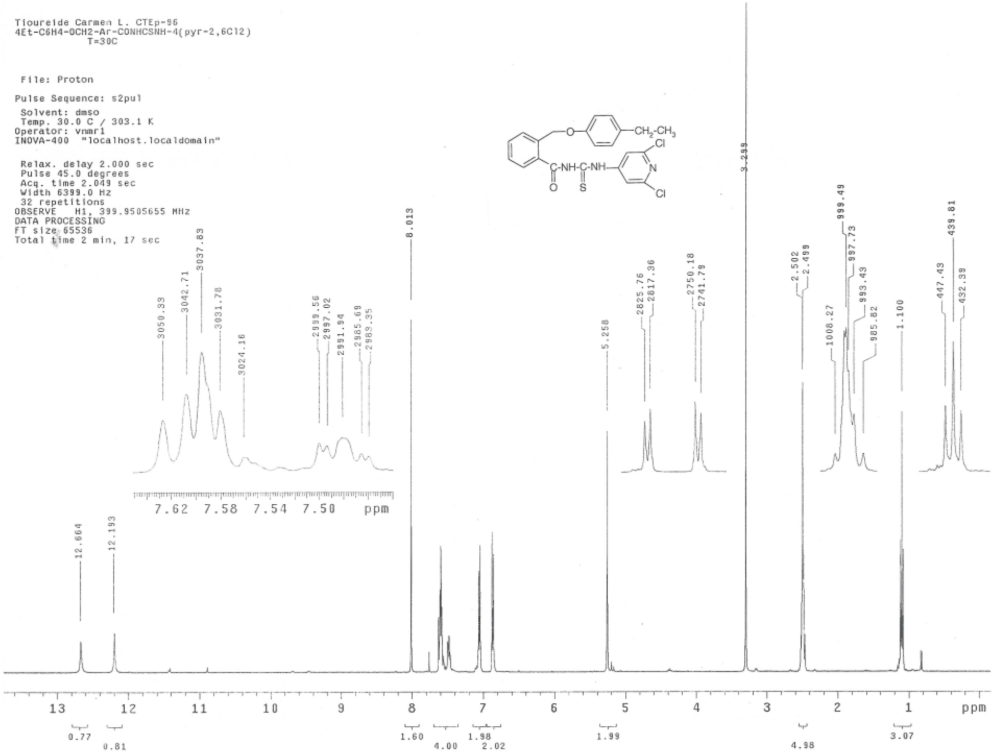

Figure S40. **^{1}H- NMR** for the compound **1n**

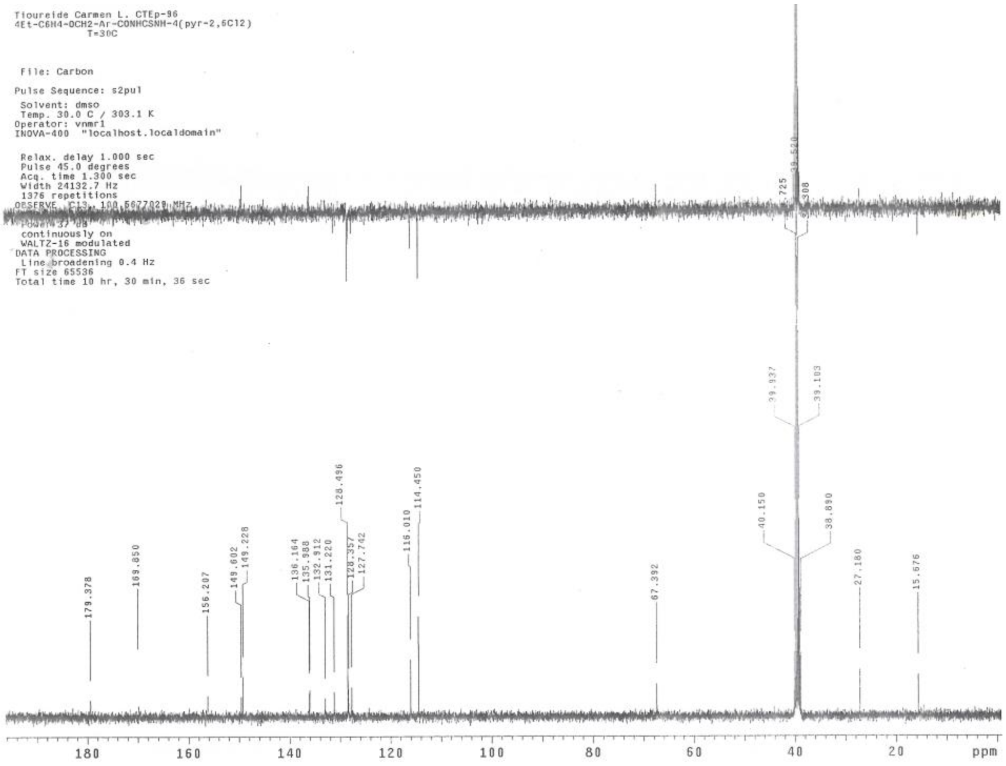

Figure S41. **^{13}C- NMR** for the compound **1n**

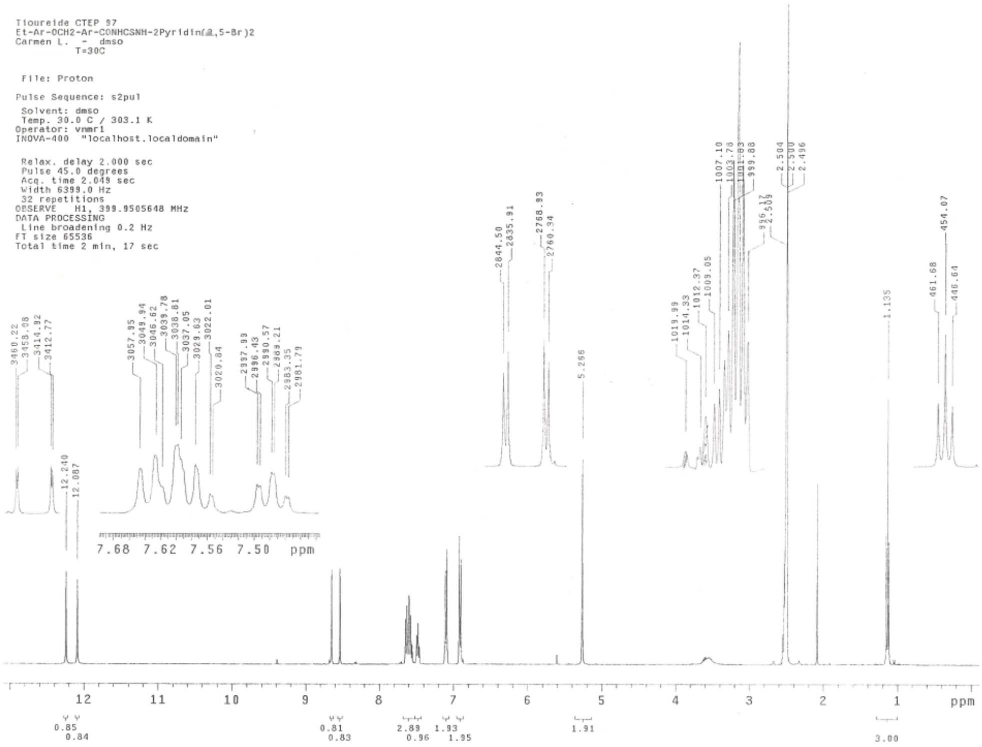

Figure S42. **^{1}H-RMN** for the compound **1o**

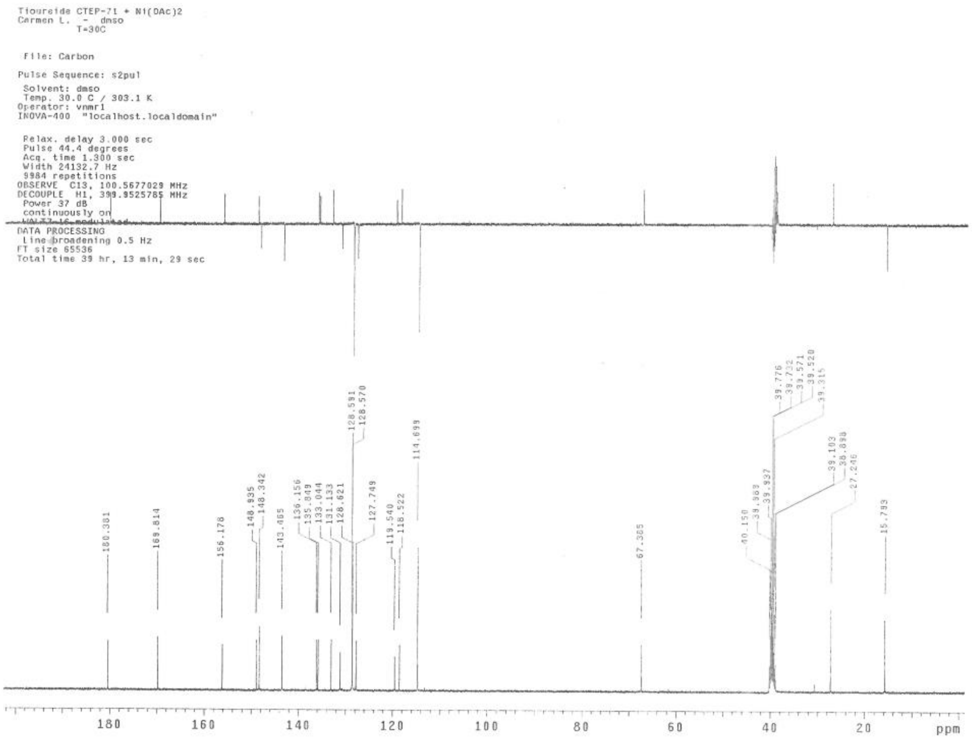

Figure S43. **^{13}C-RMN** for the compound **1o**

IR spectra for the compounds 1a – 1o

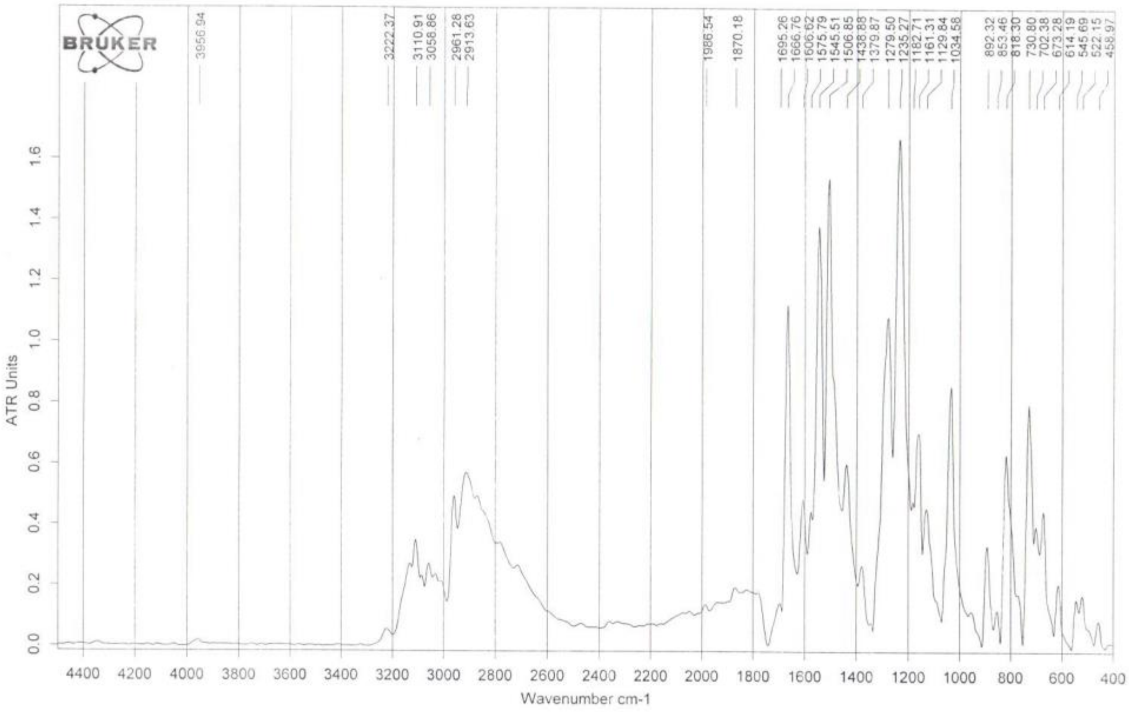

Figure S44. IR spectrum for the compound **1a**

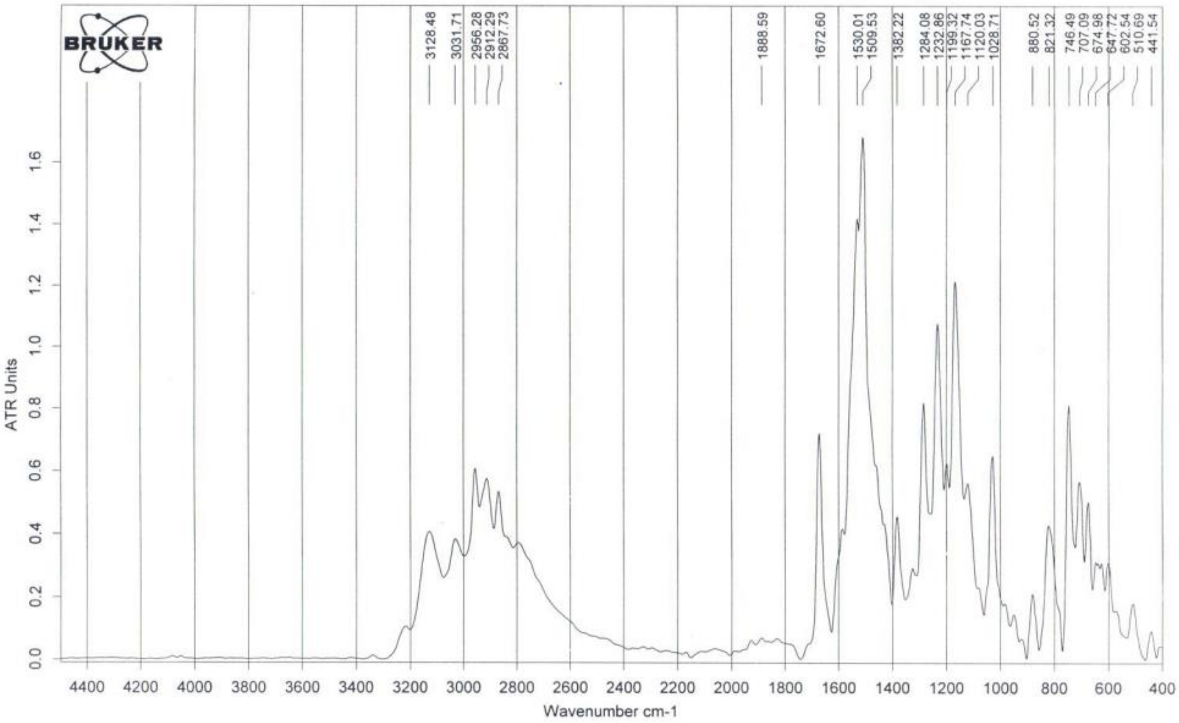

Figure S45. IR spectrum for the compound **1b**

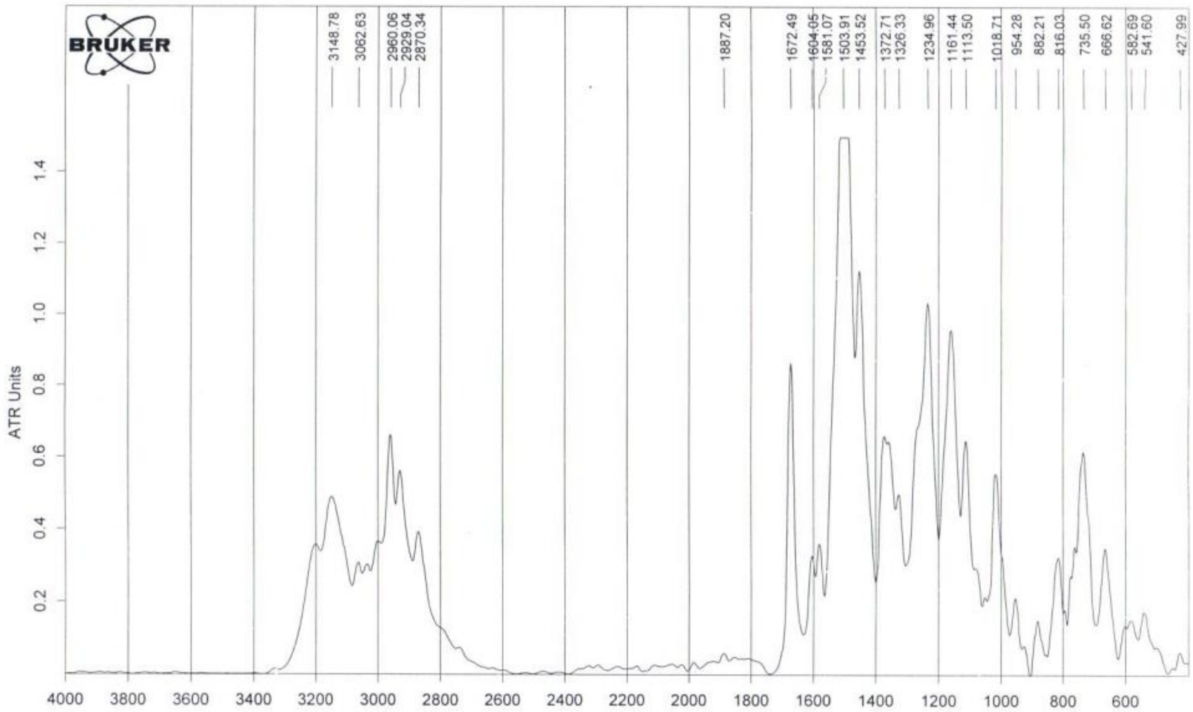

Figure S46. IR spectrum for the compound **1c**

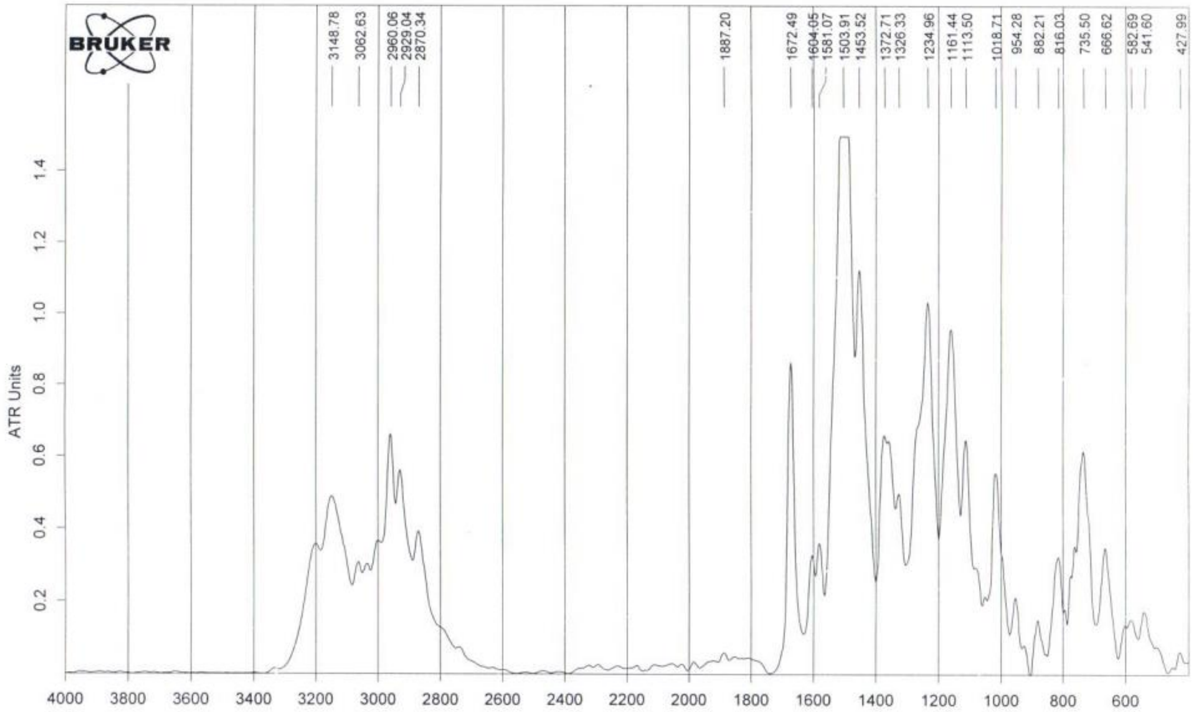

Figure S47. IR spectrum for the compound **1d**

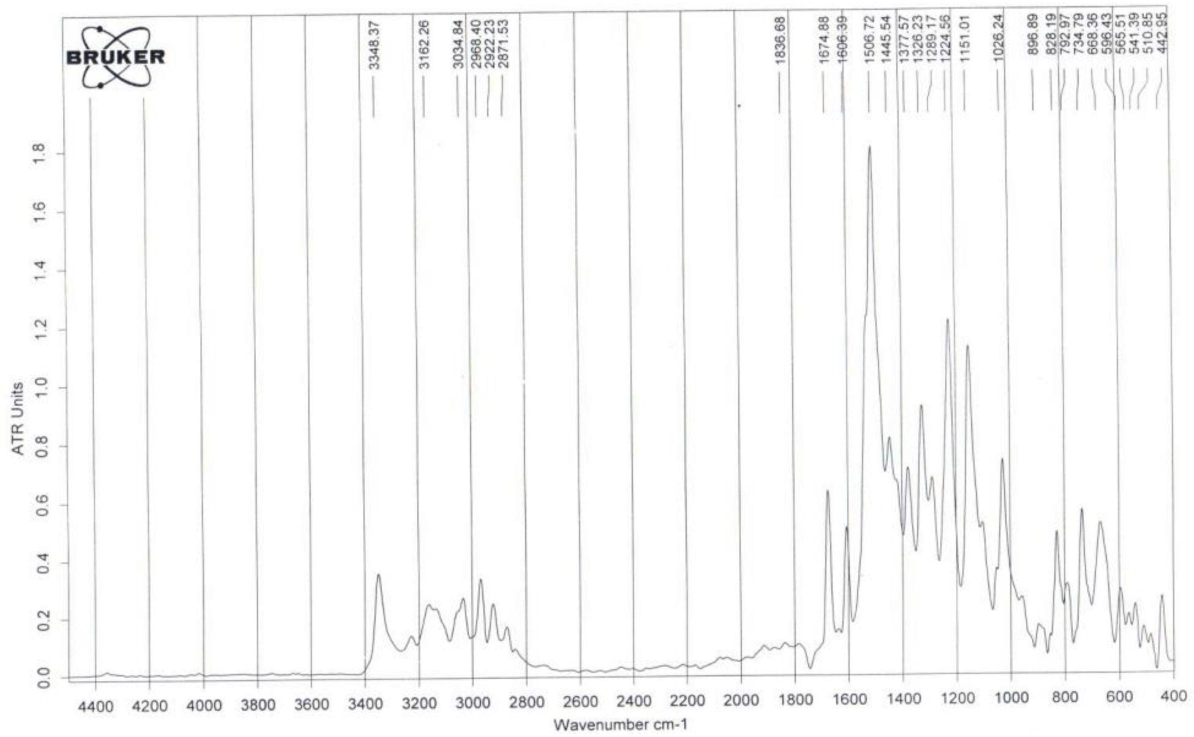

Figure S48. IR spectrum for the compound **1e**

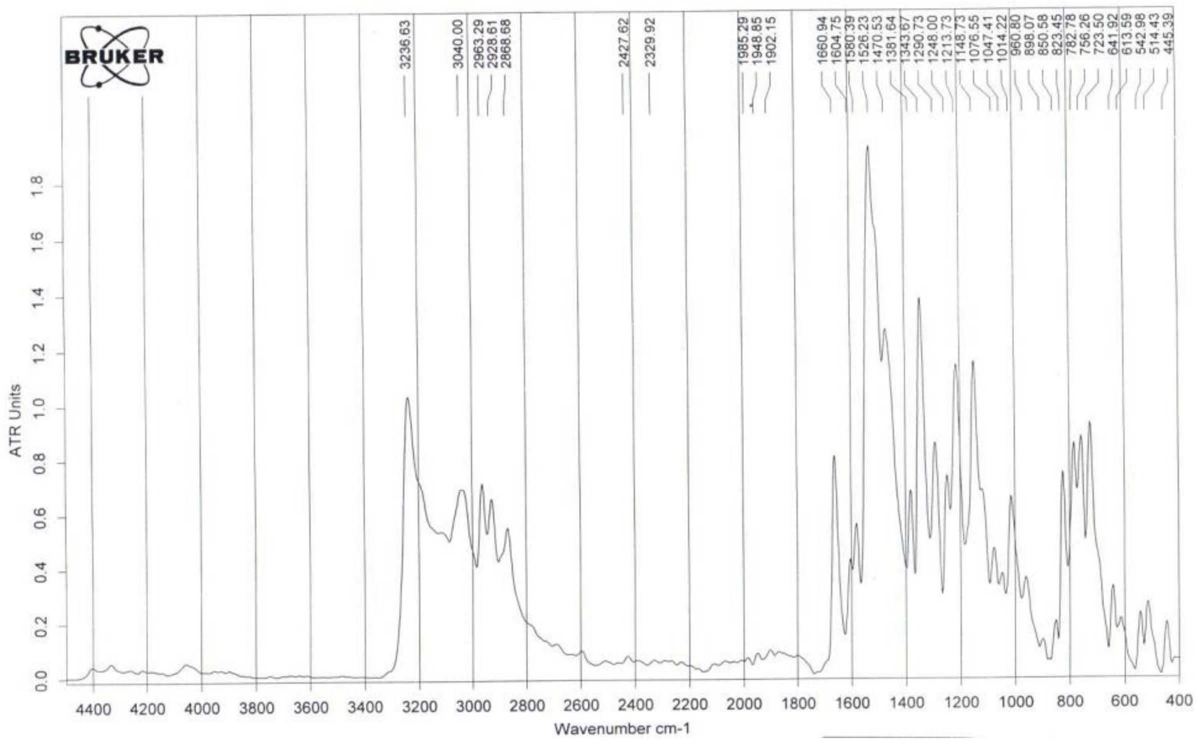

Figure S49. IR spectrum for the compound **1f**

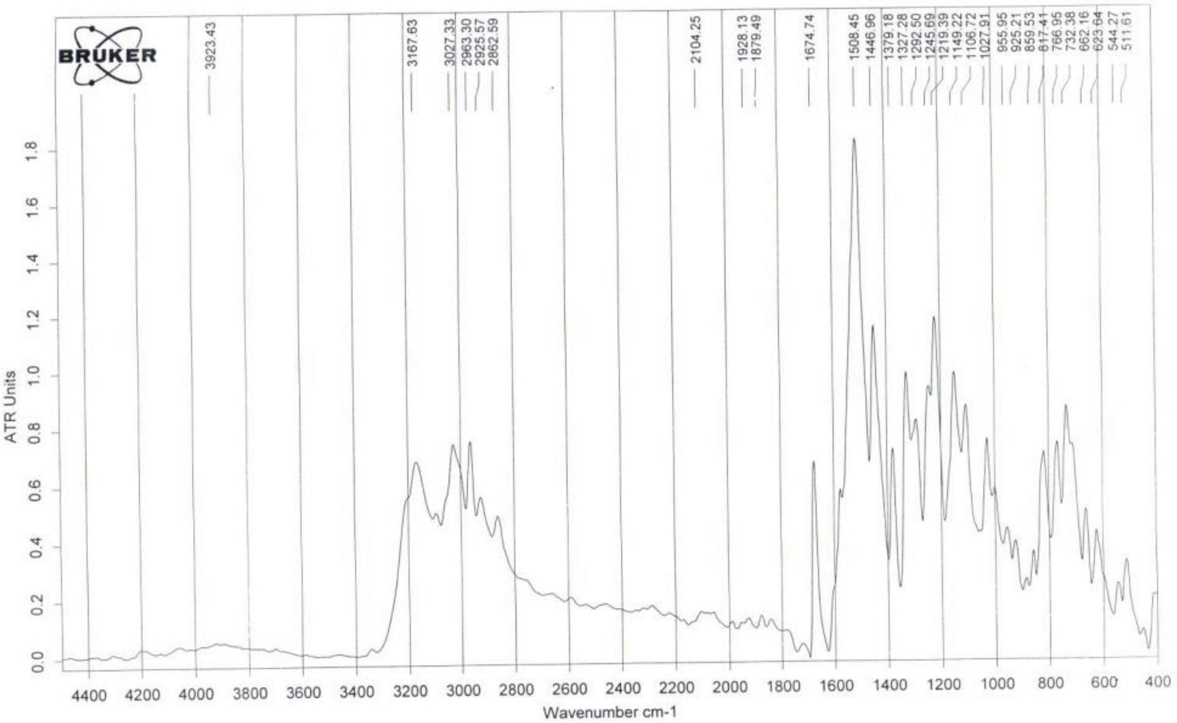

Figure S50. IR spectrum for the compound **1g**

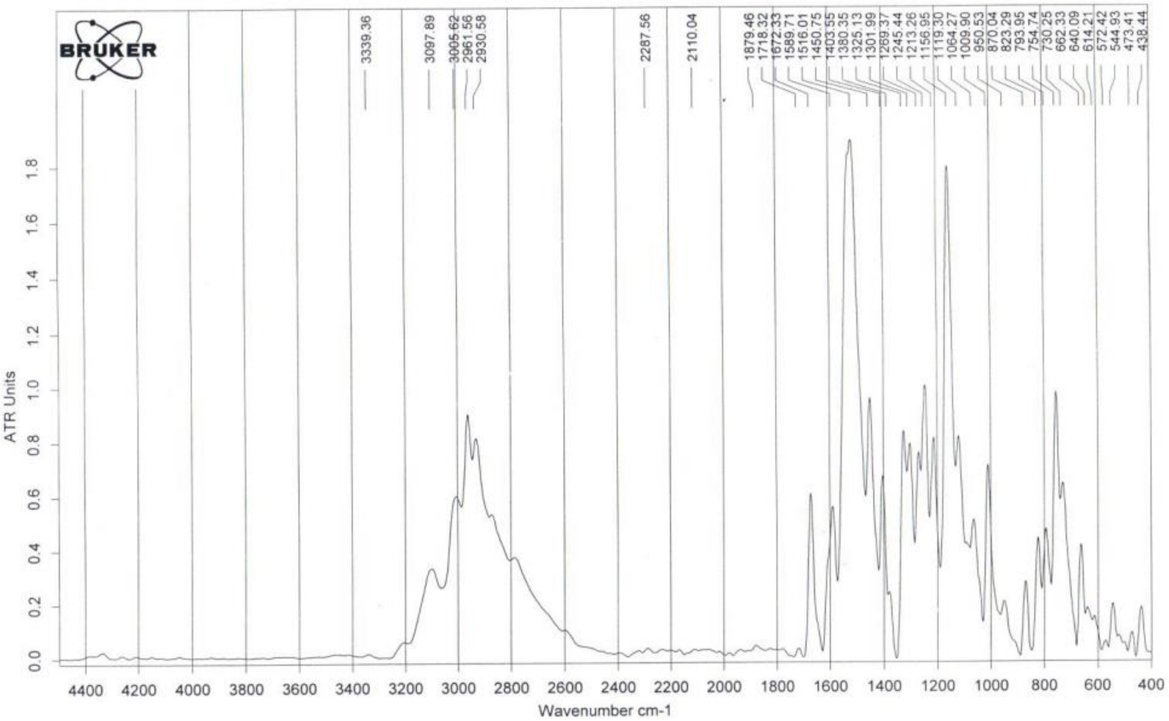

Figure S51. IR spectrum for the compound **1h**

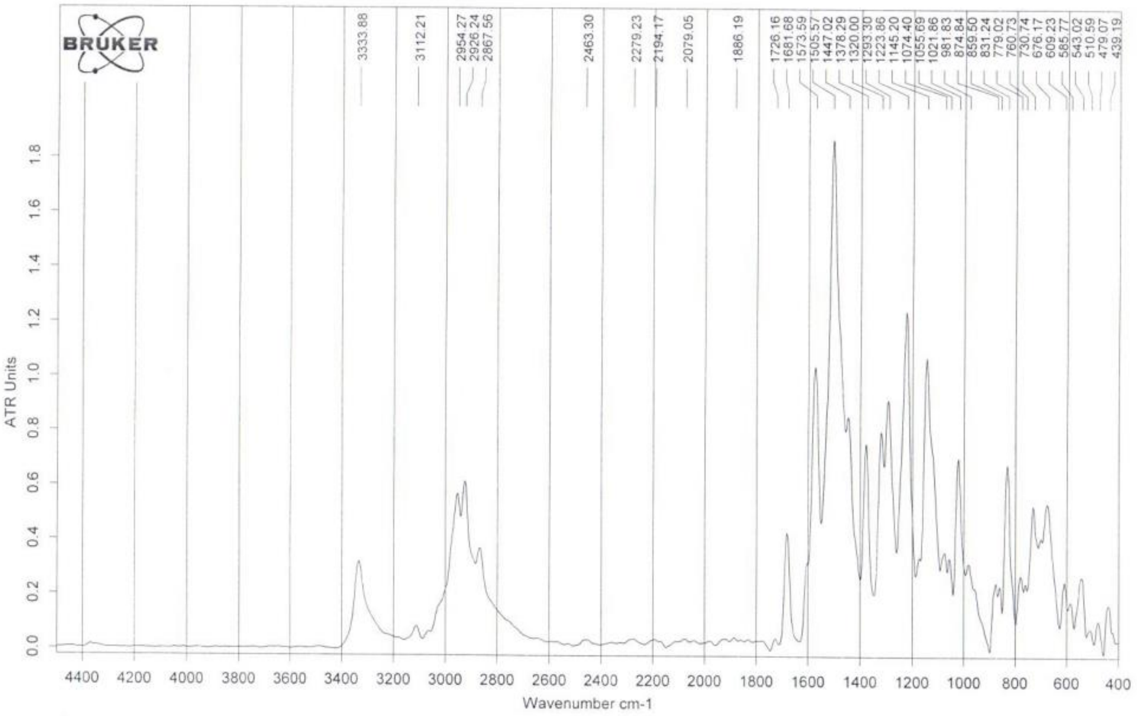

Figure S52. IR spectrum for the compound **1i**

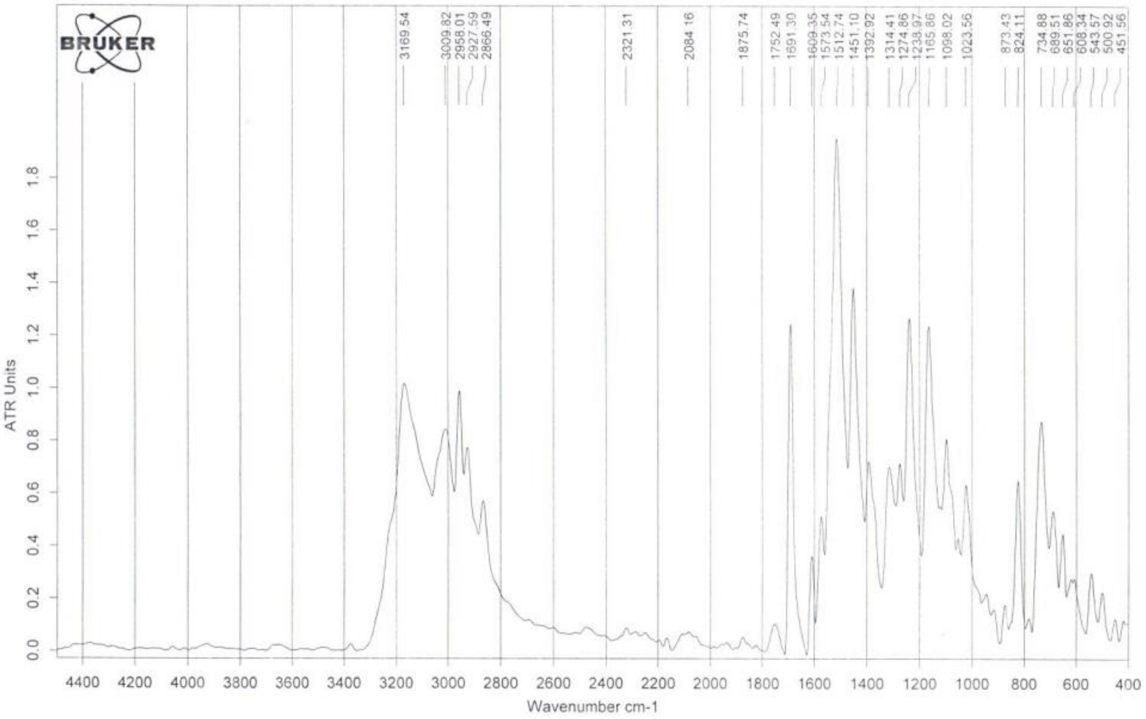

Figure S53. IR spectrum for the compound **1j**

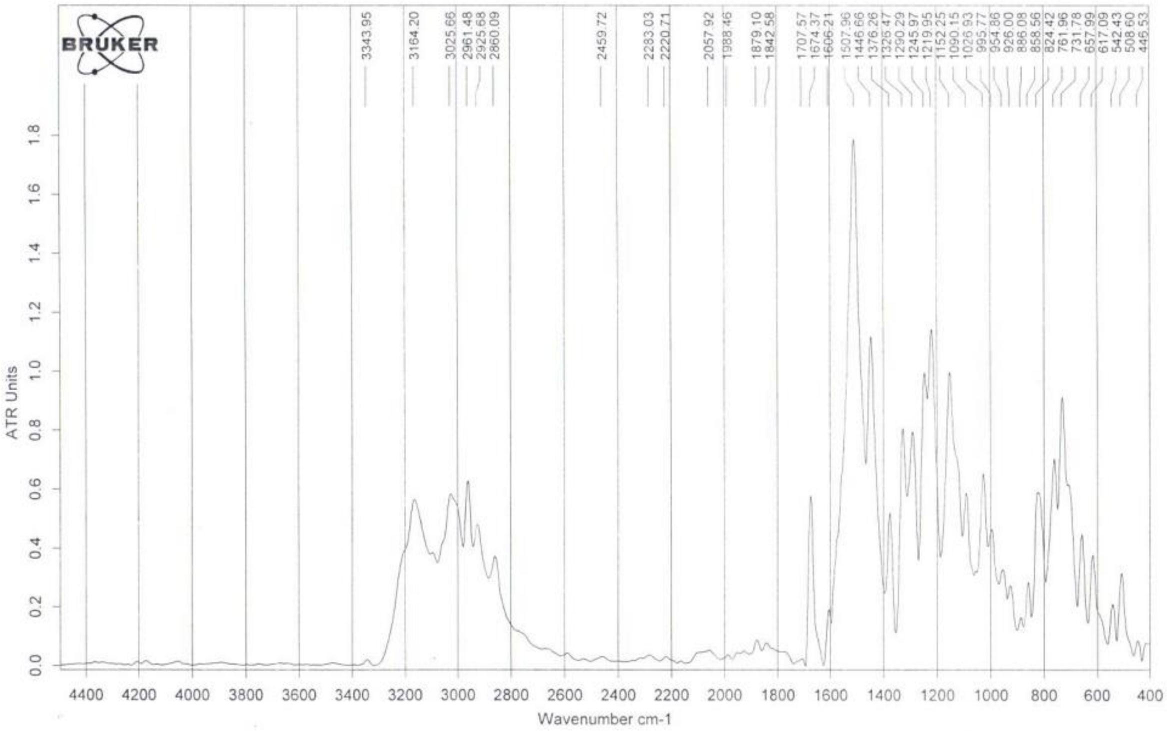

Figure S54. IR spectrum for the compound **1k**

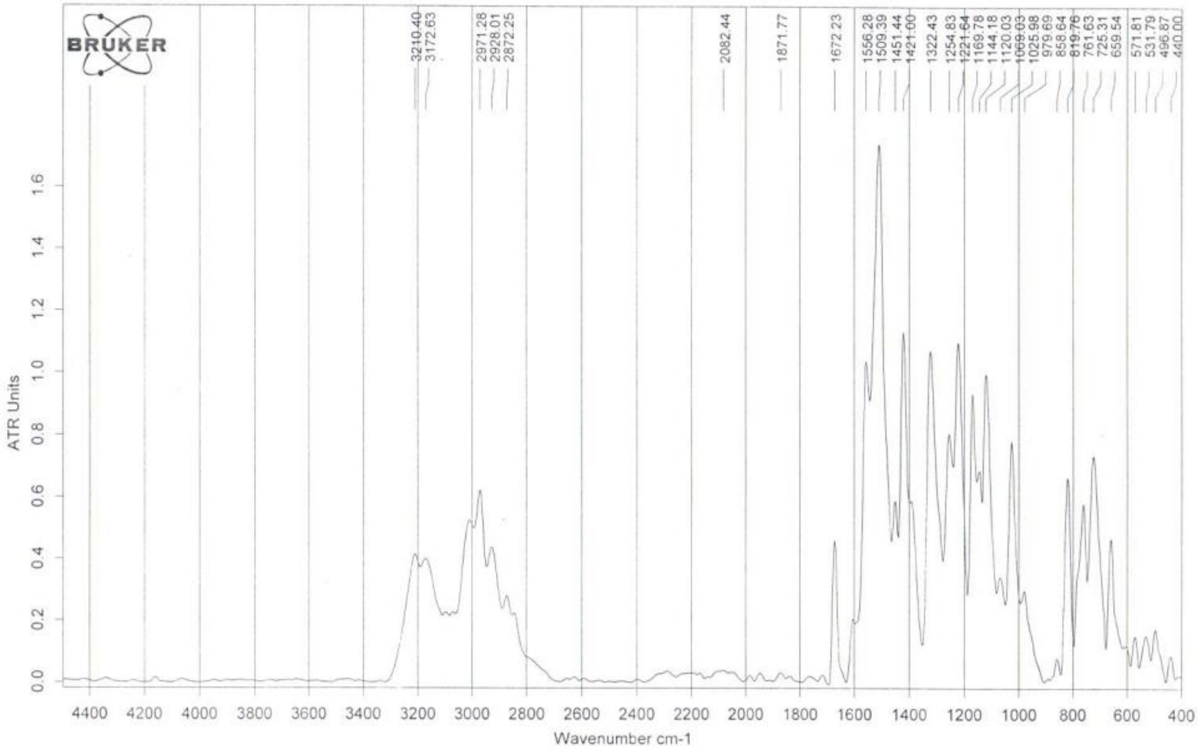

Figure S55. IR spectrum for the compound **1l**

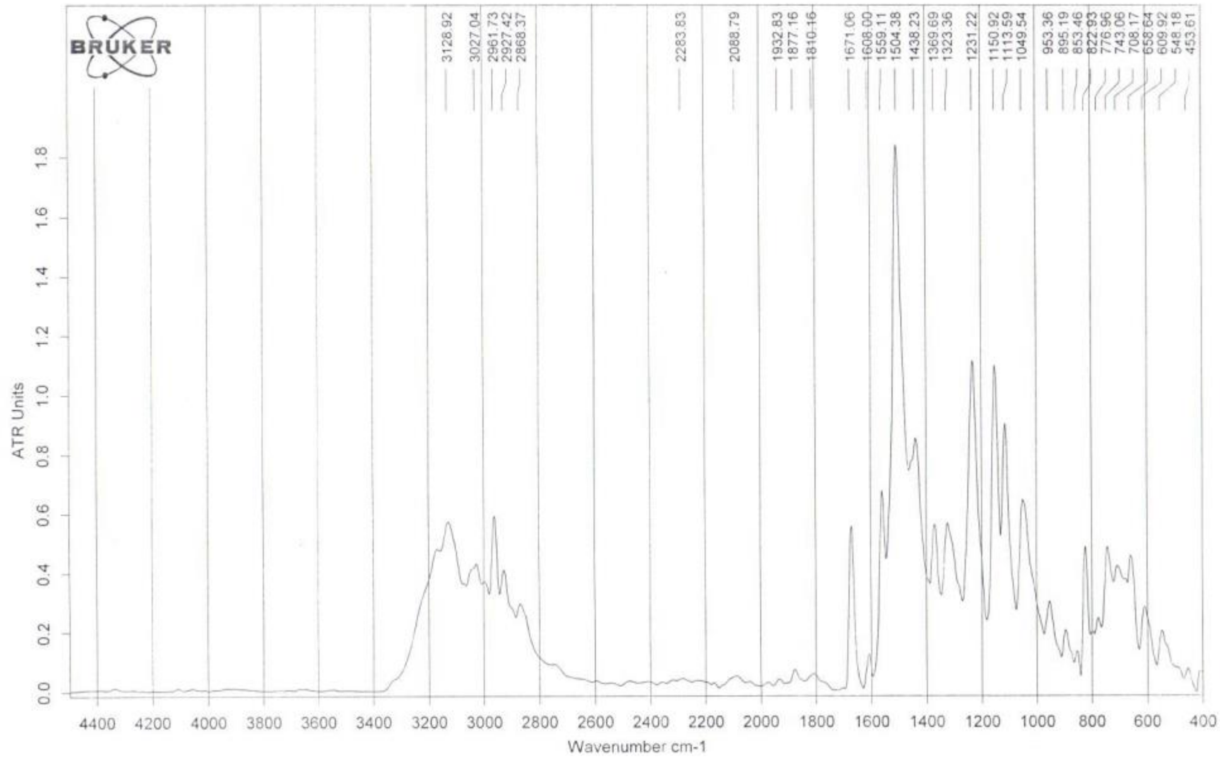

Figure S56. IR spectrum for the compound **1m**

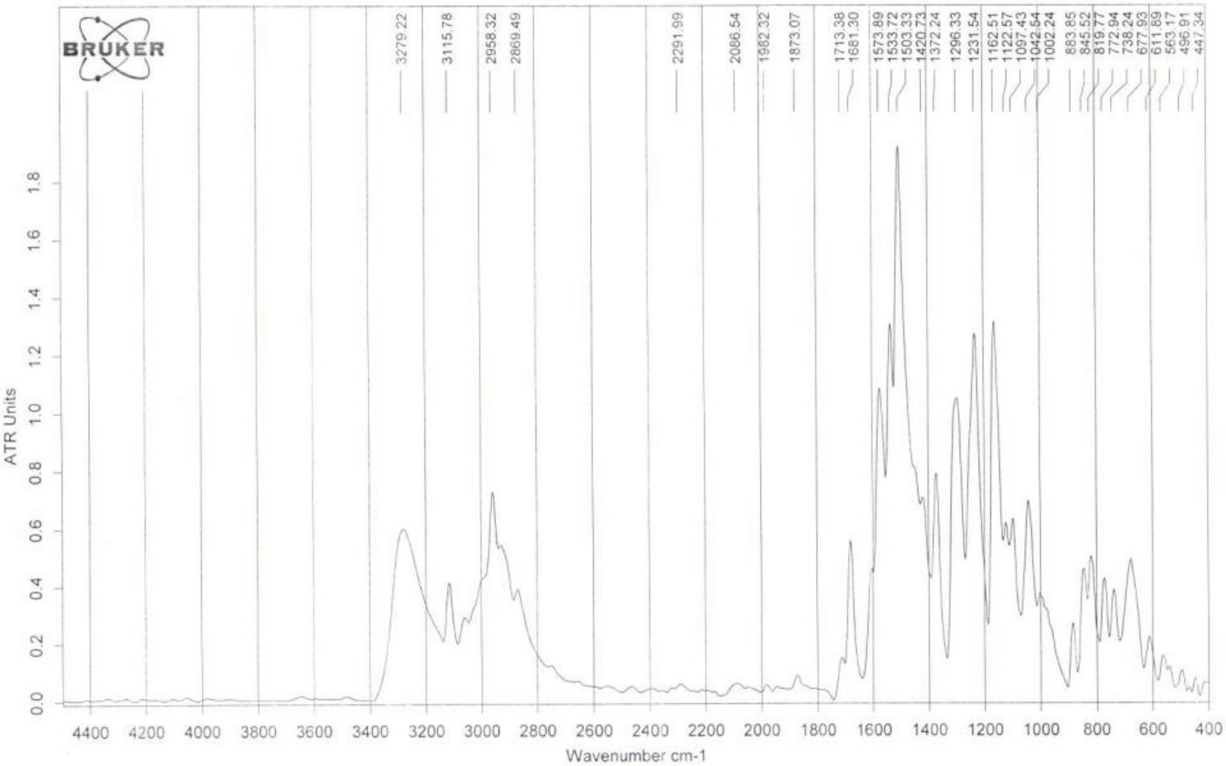

Figure S57. IR spectrum for the compound **1n**

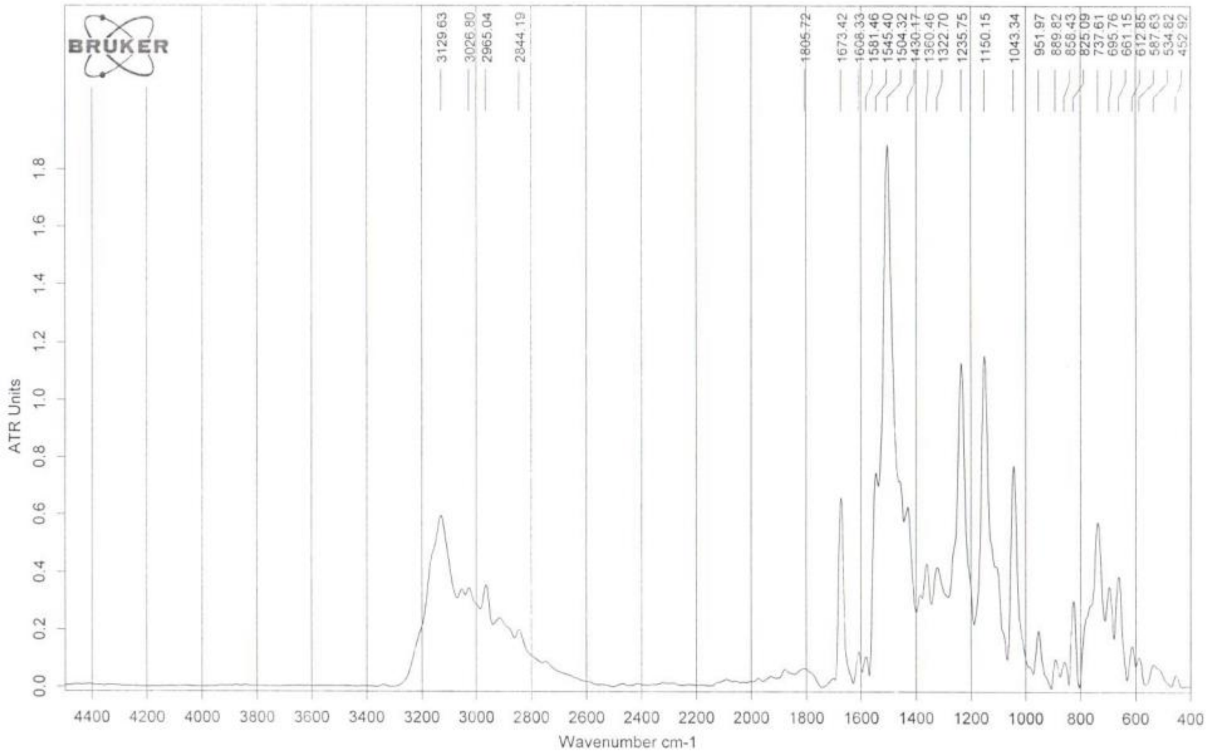

Figure S58. IR spectrum for the compound **1o**
